# Synthesis and Functional Properties of Fluorinated Phosphonate‐Substituted Porphyrins: Excellent Photosensitizers and Robust Precursors for Photoactive Langmuir–Schaefer Films

**DOI:** 10.1002/chem.71118

**Published:** 2026-05-15

**Authors:** Azhar Kechiche, Cyrille Monnereau, Chantal Andraud, Alla Bessmertnykh‐Lemeune, Yoann Rousselin, Andrey V. Cheprakov, Elizaveta V. Ermakova, Alexandra I. Zvyagina, Habib Nasri

**Affiliations:** ^1^ ENS de Lyon, UMR 5182, CNRS Laboratoire de Chimie Université Claude Bernard Lyon 1 Lyon France; ^2^ Institut de Chimie Moléculaire de l'Université Bourgogne Europe UMR CNRS 6302 Université Bourgogne Europe Dijon France; ^3^ Department of Chemistry Lomonosov Moscow State University Moscow Russia; ^4^ Frumkin Institute of Physical Chemistry and Electrochemistry Russian Academy of Sciences Moscow Russia; ^5^ Laboratory of Physical Chemistry of Materials (LR01ES19) Faculty of Sciences of Monastir University of Monastir Monastir Tunisia

**Keywords:** Langmuir–Schaefer films, porphyrinoids, phosphonate, photocatalysis, sulfide

## Abstract

Fluorinated porphyrins have attracted considerable attention in PDT, sensing and catalysis. Guided by demands for functional porphyrins with tunable electronic properties, high photostability, and controlled supramolecular organization, we report a synthetic approach to *meso*‐tetrakis[4‐(diethoxyphosphoryl)‐2,3,5,6‐tetrafluorophenyl]porphyrin (H_2_TF_4_PPP), which combines the advantages of diethyl phosphonate and fluorine substituents within a highly symmetrical porphyrin molecule. X‐ray diffraction analysis of H_2_TF_4_PPP reveals that diethyl phosphonates govern the crystal packing, while the macrocycles avoid *π–π* stacking. Zn(II) and Pd(II) complexes with H_2_TF_4_PPP were prepared in high yields. In contrast, insertion of In(III) ions exhibiting strong Lewis acidity was accompanied by partial hydrolysis of phosphonate groups. H_2_TF_4_PPP and its Zn(II) and Pd(II) complexes efficiently generate singlet oxygen displaying a good solubility in various organic solvents. The noble‐metal‐free porphyrin H_2_TF_4_PPP surpasses Pd(II) complex of non‐fluorinated analogue (PdTPPP) in photocatalytic performance, enabling sustainable oxidation of sulfides to sulfoxides with oxygen. H_2_TF_4_PPP forms a stable Langmuir monolayer which can be transferred onto solid substrates, yielding dense single‐layer films containing fiber‐shaped nanoparticles. The films thus obtained are emissive and their photostability surpasses that of H_2_TPPP films. Water‐soluble porphyrins H_2_TF_4_PPP‐A and PdTF_4_PPP‐A with phosphonic acid groups were also prepared and briefly investigated.

## Graphical abstract

This is a comprehensive study of new family of high functionalized *meso*‐tetraarylporphyrins. A synthetic approach to phosphonate‐substituted fluorinated porphyrins was developed. These compounds were investigated with respect to singlet oxygen generation and photostability. Their advantages as photocatalysts were demonstrated studying the photooxidation of sulfides by molecular oxygen. Notably, this work introduced the first family of fluorinated *meso‐*tetraarylpophyrins that can be deposited onto solid substrates using LS technique, yielding emissive and highly photostable nanoscale films.

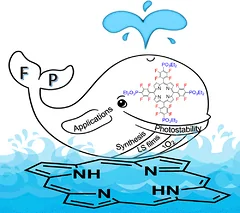

## 1 Introduction

Fluorinated porphyrins have attracted considerable attention in porphyrin chemistry. Fluorination of porphyrins enhances their oxidative stability, shifts redox potentials, modulates magnetic, and excited‐state properties, and improves their pharmacokinetics while maintaining low biotoxicity [1, 2, 3, 4, 5, 6, 7, 8, 9, 10]. The hydrophilic–lipophilic balance of fluorinated derivatives is highly sensitive to both the number and position of fluorine atoms, a feature widely exploited to optimize photosensitizers and tune the properties of functional porphyrin‐based materials [11, 12, 13].

Fluorine‐containing porphyrins have been studied in solutions [9, 14, 15], micelles, and lipid assemblies [12, 16], organogels [12], and a variety of molecular materials, including thin films [17, 18], polymers [1, 5, 6], sol–gels [19], coordination polymers [20, 21] such as MOFs [22, 23, 24], covalent organic frameworks (COFs) [25], and self‐organized organic nanoparticles [26, 27].

Specific fluorinated porphyrin derivatives have been prepared to enhance the performance of anticancer, antiviral, and antimicrobial agents in photodynamic therapy (PDT) [14, 15, 28, 29, 30, 31], as boron neutron capture therapy (BNCT) agents [32], as biocompatible redox MRI probes [33], and for theranostic platforms that combine ^19^F NMR imaging with PDT or chemotherapy for imaging‐guided cancer treatment [12, 34, 35]. These compounds have also been employed to improve perovskite solar cells [36], sensors [15, 37, 38], redox catalysts [8, 9, 19, 20, 22, 23, 25, 39, 40, 41, 42, 43, 44, 45], and, more recently, photocatalysts [46].

**FIGURE 1:**
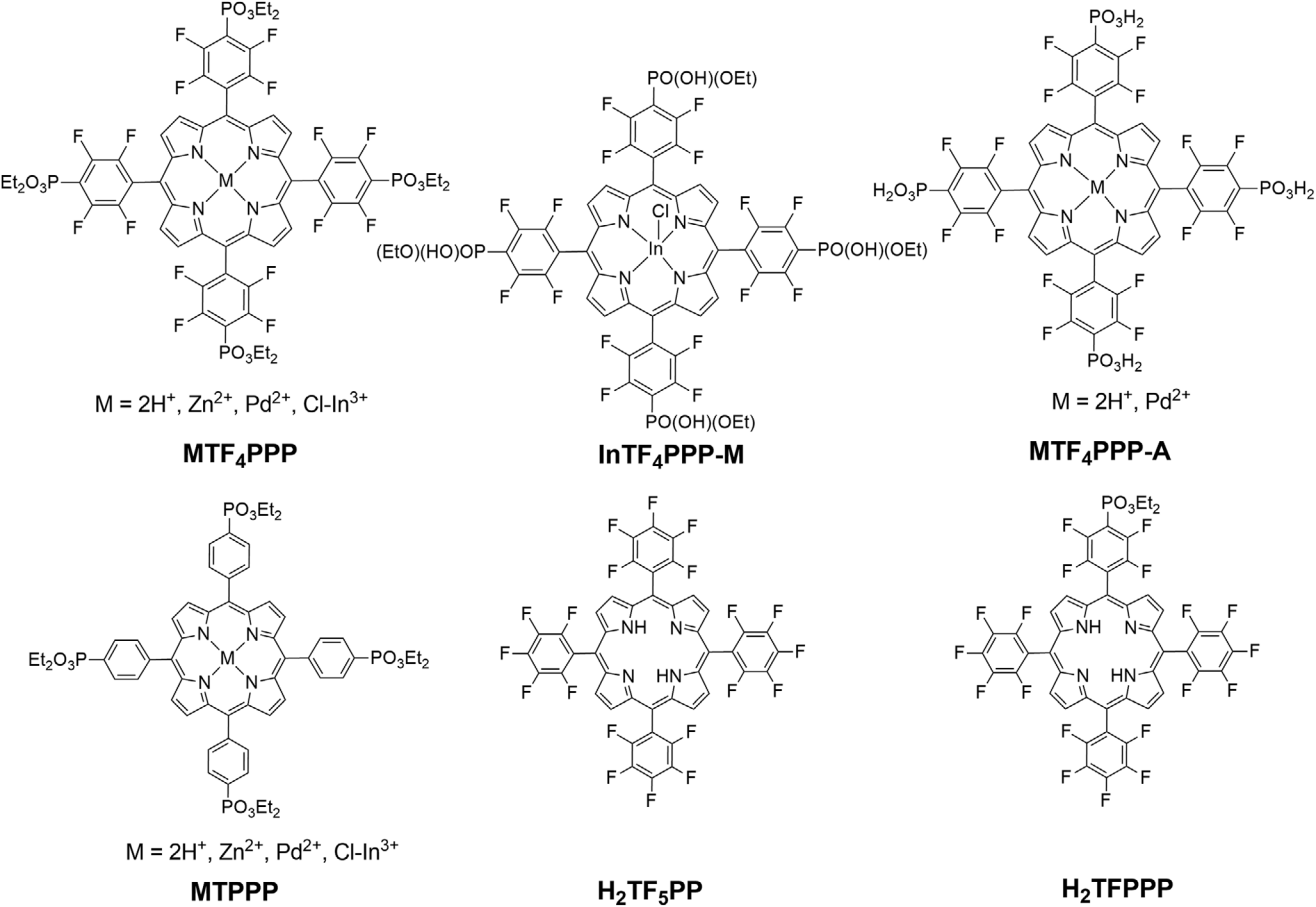
Porphyrins MTF_4_PPP, MTF_4_PPP‐A, and InTF_4_PPP‐M investigated in this work and referenced compounds.

On another front, porphyrins functionalized with phosphonate groups have attracted increasing interest due to their electron‐withdrawing properties, high solubility in both organic and aqueous media, and the propensity of the phosphonate group to form structurally organized supramolecular aggregates in bulk and on solid surfaces [47, 48, 49, 50, 51]. Porphyrinylphosphonates have demonstrated high efficiency in protein binding [52], sensing in aqueous media [49, 53, 54], and photocatalysis [51]. Diethoxyphosphoryl(PO_3_Et_2_)‐substituted derivatives are readily accessible and can be activated to serve as anchors in preparation of thin film materials [55, 56]. Polyphosphonates are also well‐known molecular precursors for porous sol–gels [57], MOFs [58], hydrogen–organic frameworks (HOFs) [59], and COFs [60]. In particular, non‐fluorinated analogues of MTF_4_PPPs, referred to here as MTPPPs (Figure 1), provide porous MOFs and HOFs with conducting [61], semiconducting [59, 62], and proton‐conducting [63] properties.

In this framework, porphyrins that combine fluorine and phosphorous substituents remain underexplored. Reported examples include phosphonium salts [64] and compounds where fluorinated aryl and phosphonate groups are separated by flexible linkers [17, 18, 65]. Despite their potential interest in catalysis and material chemistry, A_4_‐type fluorinated porphyrins MTF_4_PPP have not been described; only an unsymmetrical A_3_B‐type porphyrin (H_2_TFPPP, Figure 1) has been prepared and structurally characterized [66].

Here, we report the synthesis and characterization of *meso*‐tetraarylporphyrins MTF_4_PPPs bearing *para‐*phosphonate substituents on all fluorinated aryl substituents (Figure 1). Free‐base porphyrin H_2_TF_4_PPP and its Zn(II), Pd(II), and In(III) complexes were prepared followed by investigation of their structure and photophysical properties, photostability, singlet oxygen generation efficiency, and their photocatalytic activity studying selective photooxidation of sulfides to sulfoxides by molecular oxygen. In order to investigate their potential as water‐soluble dyes relevant to photocatalytic and biomedical applications, phosphonic acids H_2_TF_4_PPP‐A and PdTF_4_PPP‐A were prepared and briefly studied. We also demonstrated that the introduction of PO_3_Et_2_ substituents plays a decisive role in the preparation of Langmuir–Schaefer (LS) films from fluorinated porphyrins.

## 2 Results and Discussion

### 2.1 Synthesis of Porphyrin H_2_TF_4_PPP

In this work, we investigated synthetic approaches to 5,10,15,20‐tetrakis[4‐(diethoxyphosphoryl)‐2,3,5,6‐tetrafluorophenyl]porphyrin (H_2_TF_4_PPP, Figure 1).

Fluorinated *meso*‐tetraarylporphyrins are typically synthesized using the nucleophilic substitution of 5,10,15,20‐tetrakis(pentafluorophenyl)porphyrin (H_2_TF_5_PP), which regioselectively reacts with *N‐*, *S‐*, and *O‐*nucleophiles to afford, in most cases, tetra‐substituted derivatives bearing the nucleophilic residues at the *para*‐ position of the electron‐deficient C_6_F_5_ residues [67, 68, 69]. By tuning the amount of nucleophile and the reaction conditions, selective *para*‐functionalization of one or three phenyl groups can also be achieved [17, 28, 67, 70, 71]. Unfortunately, this synthetic strategy proves inefficient for introducing phosphorus‐containing groups. Heating H_2_TF_5_PP with an excess of triethyl phosphite in toluene resulted in the substitution of only one *para*‐fluorine atom by the PO_3_Et_2_ group, yielding H_2_TFPPP with a modest yield of 22% [66]. To the best of our knowledge, the introduction of phosphorous‐containing substituents in four fluorinated *meso‐*aryl residues of H_2_TF_5_PP was reported only once when this porphyrin was reacted with triethylphosphine in the presence of trimethylsilyl triflate yielding the phosphonium salt [64]. However, these reaction conditions are unsuitable for trialkyl phosphites which are required to prepare analogous phosphonated derivatives.

Two general synthetic strategies are commonly employed for the preparation of *meso*‐tetraarylporphyrins. The post‐functionalization of a preformed porphyrin macrocycle represents the first approach, in which transition‐metal‐catalyzed carbon–carbon and carbon–heteroatom (N, O, S, Se, P, B) bond‐forming reactions are particularly well suited [72, 73, 74]. Notably, the conversion of bromoporphyrins and *meso‐*tetrakis(4‐bromophenyl)porphyrins into PO_3_Et_2_‐substituted derivatives has been reported to proceed smoothly [48, 75, 76]. In the second strategy, functionalized aldehydes or *meso*‐perfluoroaryldipyrranes are prepared and introduced in the macrocyclization reaction to form porphyrinogens, which are subsequently oxidized to afford the desired porphyrins [77, 78, 79, 80, 81, 82].

In our preliminary experiments, 5,10,15,20‐tetrakis(4‐bromo‐2,3,5,6‐tetrafluorophenyl)porphyrin (H_2_TBrF_4_PP) was selected as the starting material for the preparation of the PO_3_Et_2_‐containing porphyrin H_2_TF_4_PPP, since this compound can be readily obtained from pyrrole and 4‐bromo‐2,3,5,6‐tetrafluorobenzaldehyde (**1**) in 30% yield [83].

**SCHEME 1:**
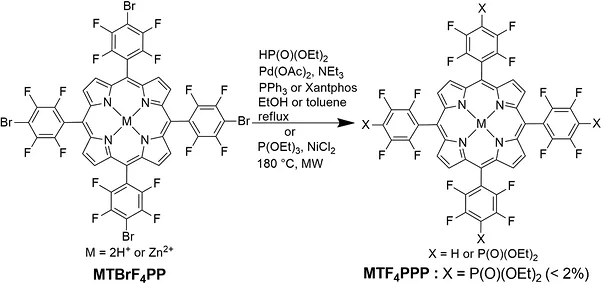
Transition‐metal‐catalyzed phosphonylation of H_2_TBrF_4_PP (M = 2H^+^, Zn(II)) with diethyl *H*‐phosphonate or triethyl phosphite.

When reactions of H_2_TBrF_4_PP or ZnTBrF_4_PP with diethyl *H*‐phosphonate were carried out under palladium‐catalyzed coupling conditions previously developed for the synthesis of the non‐fluorinated analogues H_2_TPPP and ZnTPPP (Pd(OAc)_2_/3PPh_3_ in ethanol, Scheme 1), the desired product was obtained only in trace amounts. A brief screening of reaction conditions demonstrated that the hydrodebromination side reaction is rapid even when the reaction was carried out in non‐protic toluene and in the presence of the bidentate ligands (Dppf, Xantphos) as previously observed for other sterically hindered porphyrin derivatives [76].

As shown in Scheme 1, attempts to obtain the target product by reacting H_2_TBrF_4_PP or ZnTBrF_4_PP with triethyl phosphite in the presence of NiCl_2_ under solvent‐free conditions in a microwave reactor were also unsuccessful.

**SCHEME 2:**
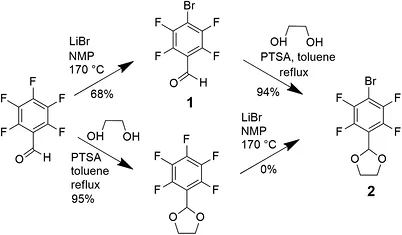
Synthesis of 4‐bromophenyl dioxolane **2**.

For these reasons, we revised the synthetic strategy and investigated the introduction of the phosphorus substituent onto the phenyl ring prior to formation of the tetrapyrrolic macrocycle. 4‐Bromo‐2,3,5,6‐tetrafluorobenzaldehyde (**1**) was obtained by reacting pentafluorobenzaldehyde with lithium bromide (LiBr) [84, 85], and protected using ethylene glycol (EG) (Scheme 2). Notably, the dioxolane **2** could not be obtained if pentafluorobenzaldehyde is first protected with EG and then reacted with LiBr in NMP at 170°C (Scheme 2).

**SCHEME 3:**
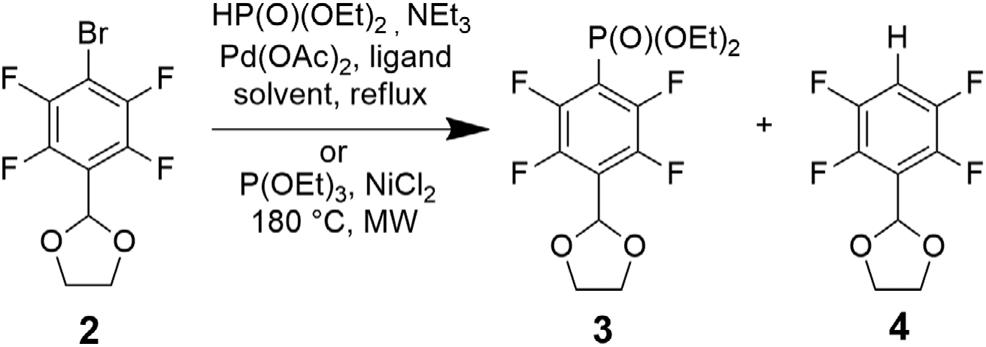
Transition‐metal‐catalyzed coupling of bromide **2** and diethyl *H*‐phosphonate or triethyl phosphite. Experimental conditions used for Pd‐catalyzed reactions are summarized in Table S1.

The phosphonylation of bromide **2** proved to be challenging. We were unable to obtain the target product **3** by heating compound **2** with triethyl phosphite and NiCl_2_ in a microwave reactor (Scheme 3).

As an alternative, the bromide **2** was reacted with diethyl *H*‐phosphonate using a palladium‐catalyzed cross‐coupling reaction, as shown in Scheme 3. Numerous attempts to optimize this reaction were unsuccessful (Table S1). Under almost all conditions studied, rapid (2–6 h) conversion of the starting bromide to the reduced compound **4** was observed. It seems that amines can serve as a hydride source [86, 87], thereby promoting hydrodebromination even in non‐protic solvents [88].

The desired product **3** was obtained in 35% yield (^19^F NMR yield) only when the reaction was performed using the Pd(OAc)_2_/Xantphos catalytic system (30 mol%) in a biphasic toluene/EG (9:1 v/v) mixture (Table S1, entry 16) [89]. However, isolation of pure phosphonate **3** by column chromatography proved challenging due to the presence of large amounts of Xantphos and phosphonated side products.

**SCHEME 4:**
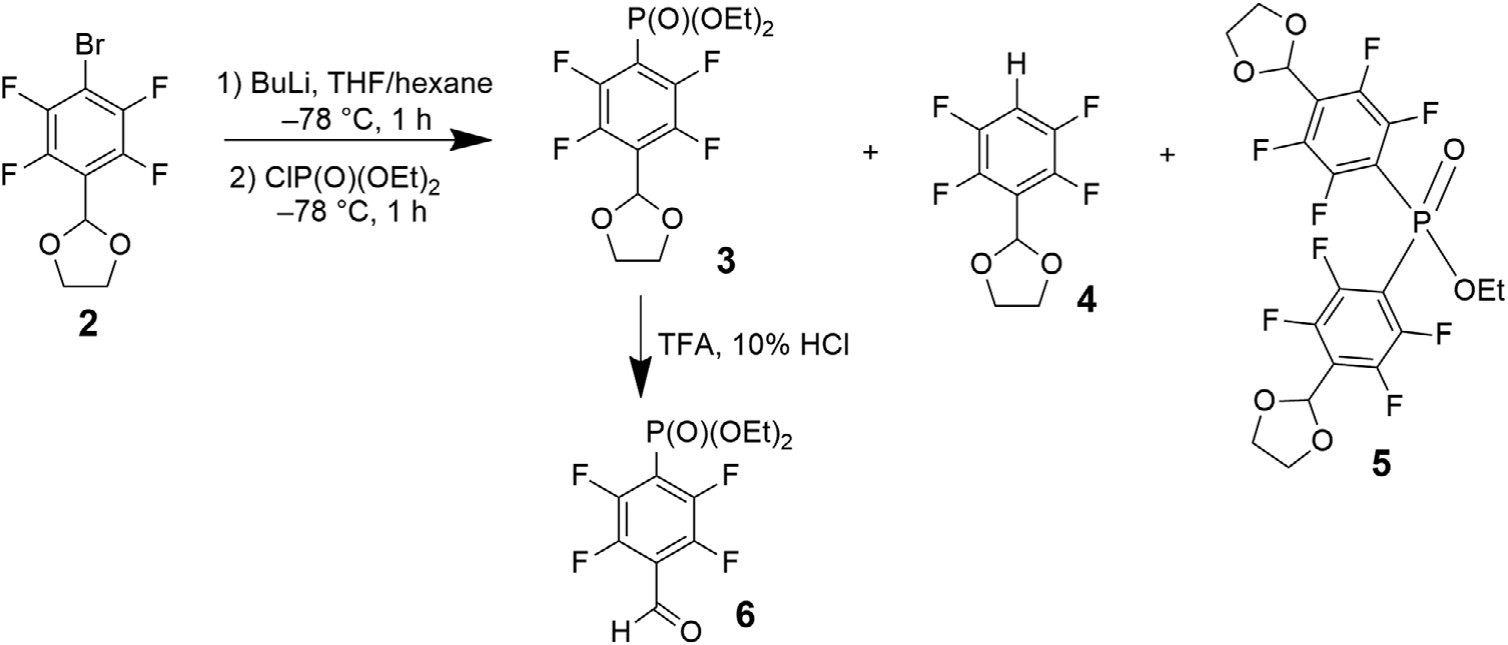
Synthesis of fluorinated arylphosphonate **6**.

Finally, we turned from catalytic methods to a classical nucleophilic substitution using the aryllithium reagent and *O,O′‐*diethyl chlorophosphonate (Scheme 4). Side reactions again proved to be a major obstacle in obtaining the target product in good yield. Along with the reduced compound **4**, the diaryl phosphinate **5** was formed in 20–30% yield due to the high reactivity of the phosphonate group in the target product bearing an electron‐withdrawing aryl substituent. Similar products resulting from multiple nucleophilic attacks of aryllithium reagents on the pentavalent phosphorus atom of the chlorophosphonate have been reported previously [90], and their formation cannot be easily controlled. Fortunately, the pure product **3** was successfully isolated in 25% yield by flash chromatography on a gram‐scale experiment. The subsequent deprotection of the aldehyde group, using the previously reported procedure [91], afforded aldehyde **6** in quantitative yield.

**SCHEME 5:**
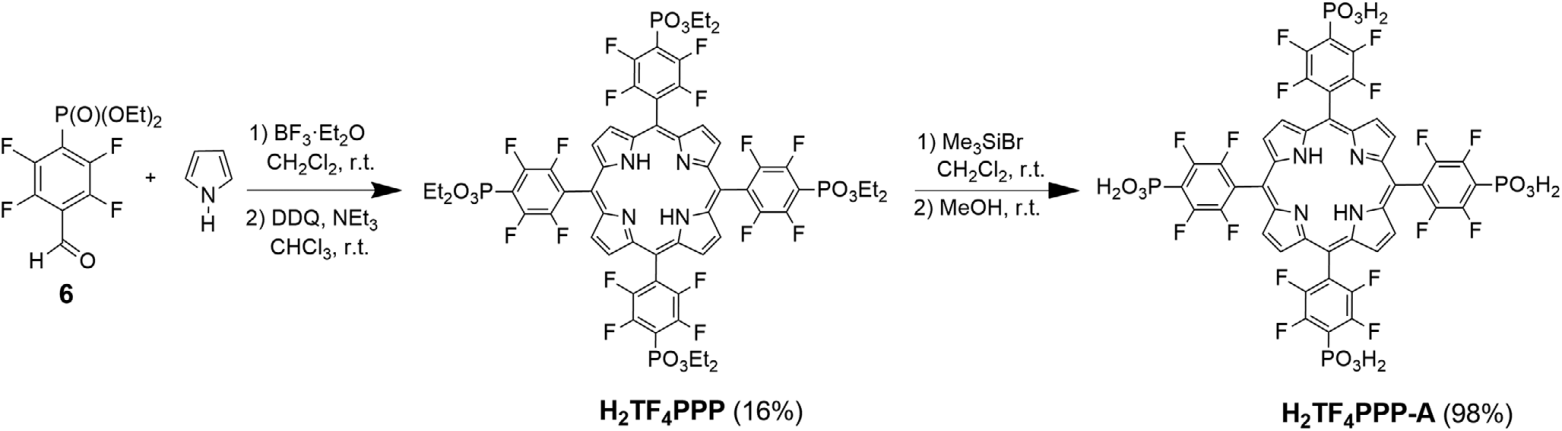
Synthesis of H_2_TF_4_PPP and H_2_TF_4_PPP‐A.

In the final step, the porphyrin H_2_TF_4_PPP was synthesized by the condensation of aldehyde **6** with pyrrole (**7**) in the presence of BF_3_·OEt_2_, followed by oxidation of the resulting porphyrinogen with DDQ (Scheme 5) [92]. A similar procedure had previously been successfully applied to the synthesis of H_2_TPPP [93]. Introduction of electron‐withdrawing fluorine substituents on the phenyl rings led to a decrease in product yield from 35–55% to 16%, likely due to the more difficult oxidation of the intermediate porphyrinogen or partial hydrolysis of the PO_3_Et_2_ groups during chromatographic purification.

The water‐soluble porphyrin H_2_TF_4_PPP‐A, containing four phosphonic acid substituents, was synthesized in quantitative yield by reacting H_2_TF_4_PPP with an excess of TMSBr in CH_2_Cl_2_ at room temperature, and subsequently treating the resulting silyl ester with methanol (Scheme 5). Notably, water‐soluble fluorinated porphyrins remain largely unexplored. Reported examples are limited to *meso*‐(4‐X‐2,3,4,5‐tetrafluorophenyl)porphyrins with carboxylate or sulfonate substituents (X) [94, 95, 96], for which detailed synthetic protocols and full spectroscopic characterization are still unavailable.

All new organic compounds obtained in this work were unambiguously characterized by ^1^H, ^13^C, ^1^ ^9^F, and ^3^ ^1^P NMR, IR, UV–vis, and HRMS analyses (Figures S30–S52). The spectroscopic data were consistent with the proposed structures. Interestingly, the phosphorus signals of compounds **3**, **5**, **6**, and H_2_TF_4_PPP were shifted upfield (Δ*δ* = 12–14 ppm) compared to those of diethyl phenylphosphonate and H_2_TPPP, despite the electron‐withdrawing nature of the fluorine substituents. This observation is in good agreement with previously reported spectral characteristics of substituted phenylphosphonic acids and their esters [97, 98]. The structure of H_2_TF_4_PPP was also investigated by single‐crystal X‐ray diffraction to elucidate the effect of fluorine substituents on the molecular geometry and packing in the crystals (see the crystallographic section).

One of the primary challenges associated with the use of porphyrins is their low solubility. The free‐base porphyrin H_2_TF_4_PPP exhibits higher solubility than H_2_TF_5_PP in chlorinated solvents (CHCl_3_, CH_2_Cl_2_). The dye also displays good solubility in tetrahydrofuran, ethyl acetate, and DMF (10^−^^3^–10^−^^2^ M), moderate solubility in acetonitrile and toluene (10^−^^4^–10^−^^3^ M), and markedly lower solubility in methanol (10^−^^7^–10^−^^6^ M), while being insoluble in DMSO.

### 2.2 Synthesis of Metalloporphyrins MTF_4_PPP, InTF_4_PPP‐M, and PdTF_4_PPP‐A

**SCHEME 6:**
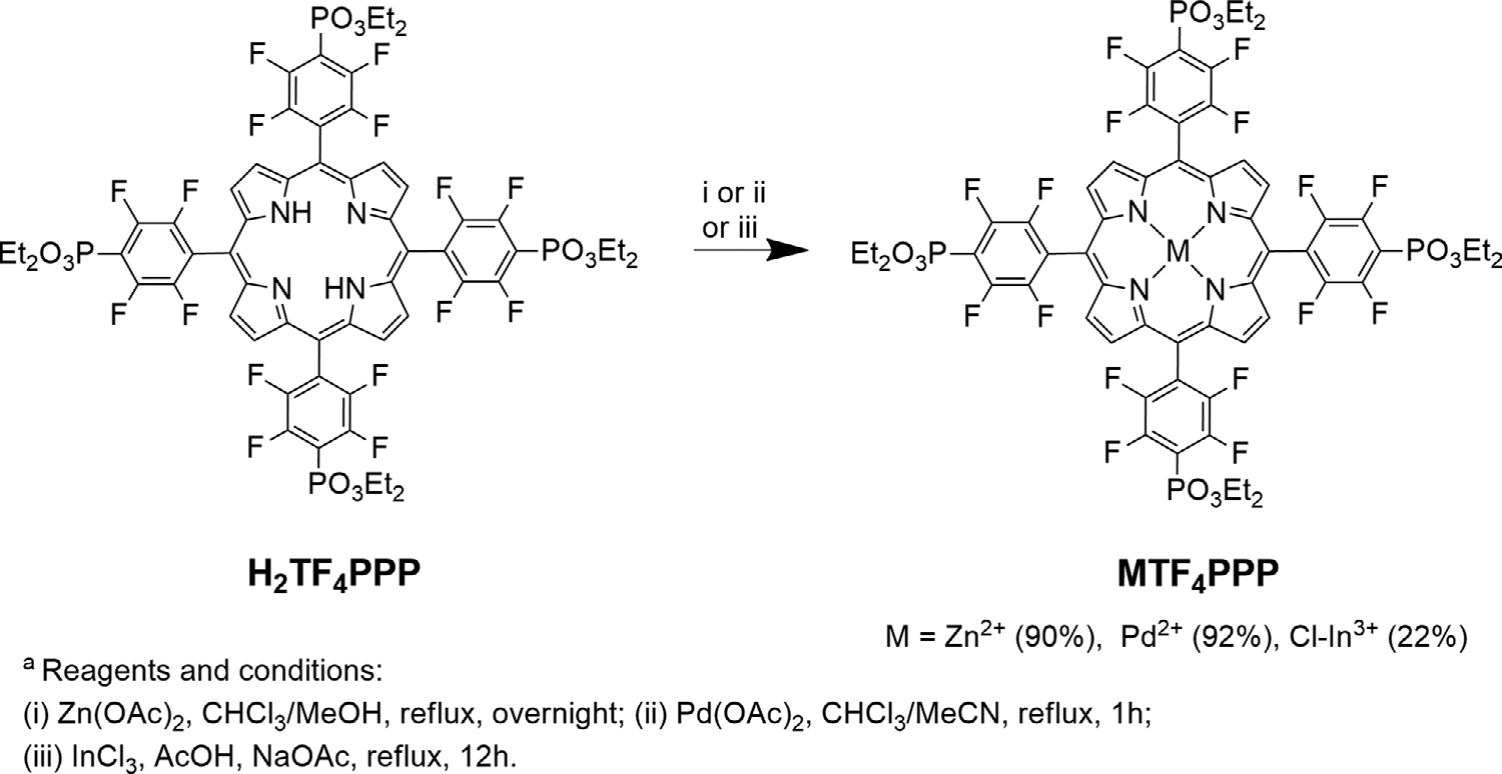
Synthesis of MTF_4_PPP.

Next, we investigated the insertion of Zn(II), Pd(II), and In(III) ions into H_2_TF_4_PPP to obtain metalloporphyrins of interest for photocatalytic applications (Scheme 6). The Zn(II) complex was prepared in 90% yield by reacting the free base porphyrin with an excess of zinc acetate dihydrate in a CHCl_3_/MeOH mixture at room temperature overnight. Insertion of Pd(II) proceeded rapidly in refluxing CHCl_3_/MeCN, affording the target complex in 92% yield when heating was stopped immediately after complete consumption of the free base porphyrin. The PdTF_4_PPP complex was quantitatively converted into its water‐soluble analogue PdTF_4_PPP‐A, bearing four phosphonic acid substituents, using the procedure described above for H_2_TF_4_PPP‐A.

The synthesis of the In(III) complex, containing a large and triply charged metal ion, proved to be considerably more challenging. The reaction of H_2_TF_4_PPP with indium(III) chloride was conducted in refluxing glacial acetic acid in the presence of sodium acetate, following a procedure previously reported for the non‐fluorinated analogue InTPPP [51]. While H_2_TPPP was reacted within 2 h under these conditions, the more electron‐deficient and sterically hindered H_2_TF_4_PPP showed significantly lower reactivity, attaining only ∼20–30% conversion. Complete conversion was observed after 12 h of reflux and the target complex was isolated by column chromatography on NEt_3_‐deactivated silica gel, though with a modest yield (22%). Upon increasing the polarity of the eluent (CH_2_Cl_2_/MeOH/NEt_3_), four colored fractions were collected. Their analysis by NMR spectroscopy revealed that those correspond to stepwise transformation of the diethyl phosphonate groups to the corresponding monoesters (PO(OEt)(OH)). This side reaction is likely promoted by the strong Lewis acidity of indium(III) chloride. When the reaction time was extended to 48 h, increasing the amount of inorganic salts, only the complex InTF_4_PPP‐M containing four phosphonate monoester groups was obtained in 21% yield after chromatographic purification of the reaction mixture. To our knowledge, this is the first example of Lewis acid–mediated transformation of phosphonate diesters to monoesters, a reaction that typically requires hydrolysis in the presence of strong aqueous base [99].

The structures of all synthesized complexes were confirmed by ^1^H, ^13^C, ^19^F, and ^31^P NMR, IR, UV–vis spectroscopies and HRMS analyses (Figures S53–S88). Interestingly, the ^19^F NMR spectrum of InTF_4_PPP in chloroform‐*d_1_* was typical for a metalloporphyrin with *C_4v_* symmetry showing four signals corresponding to non‐equivalent fluorine atoms (Figure S66). In contrast, only two signals were observed in the ^19^F NMR spectrum of this complex recorded in methanol‐*d_4_* (Figure S69). These spectral features indicated a rapid (in the NMR time) scale exchange of the axial chloride ion in this coordinating solvent.

**FIGURE 2:**
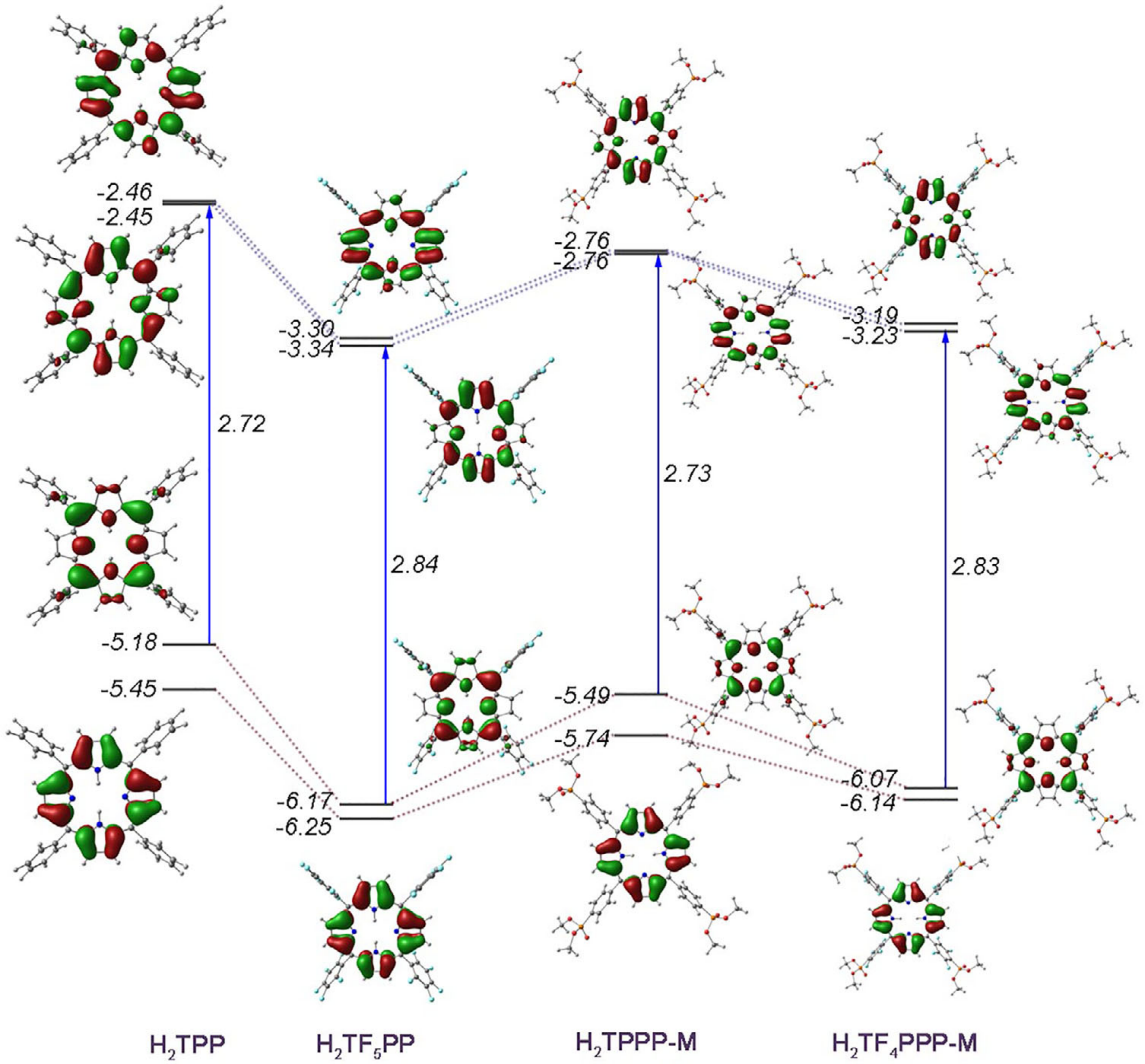
The isodensity plot of HOMO and LUMO orbitals for model chromophores H_2_TF_4_PPP‐M and related compounds obtained from DFT calculations.

DFT computations on H_2_TF_4_PPP and ZnTF_4_PPP were performed using B3LYP/6‐311G(d,p) (M = 2H^+^) or B3LYP/6‐31(d,p) (M = Zn(II)) level. To simplify the calculations, the ethyl substituents of the phosphonate groups were replaced by methyl groups; the corresponding model compounds were denoted H_2_TF_4_PPP‐M. According to these calculations, these molecules possesses a planar porphyrin core, while the peripheral phenyl rings are rotated significantly out of the macrocyclic plane, thereby preventing substantial *π*‐conjugation between the core and the phenyl substituents (Figures S1,S2, and Table 2). The frontier molecular orbitals exhibit the characteristic features of the porphyrin compounds, consistent with the four‐orbital Gouterman model. The isodensity plot of HOMO and LUMO orbitals of H_2_TF_4_PPP‐M, H_2_TPP, H_2_TF_5_PP, and model chromophore H_2_TPPP‐M are shown in Figure 2. The electron‐withdrawing fluorine and phosphonate substituents predictably decrease the energy of these orbitals. Notably, the replacement of *para*‐fluorine atom withPO_3_Me_2_ group has a small influence on the electronic structure. Consequently, the redox and photophysical properties of H_2_TF_5_PP and H_2_TF_4_PPP are expected to be similar and to resemble those of typical MTPP derivatives. The photophysical behavior and photostability of porphyrin MTF_4_PPP were subsequently investigated experimentally to evaluate their potential as photosensitizers (PS) and optical sensors.

### 2.3 Solid State Structure of H_2_TF_4_PPP

Dark‐violet single crystals of H_2_TF_4_PPP were obtained by slow diffusion of *n*‐hexane into a toluene/chloroform (1:1 v/v) solution of the compound. The complex crystallized as a toluene solvate. Two PO_3_Et_2_ groups and the toluene molecules exhibited partial disorder (50.0(2)%). The geometrical parameters of these disordered components were restrained during refinement using the standard approach (see ESI†). Crystallographic data are summarized in Tables S3–S6.

**FIGURE 3:**
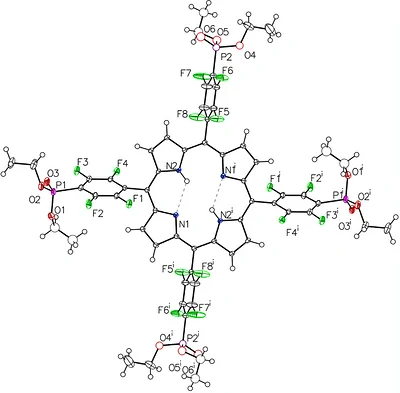
ORTEP view of H_2_TF_4_PPP. Thermal ellipsoids are drawn at 25% probability plot for clarity. Minor disordered parts and solvent molecules are omitted for clarity.

The porphyrin H_2_TF_4_PPP crystallizes in the triclinic *P*‐1 space group, as the non‐fluorinated analogue H_2_TPPP, previously reported by Venkatramaiah et al. [53]. Although H_2_TPPP was investigated as a water solvate, the overall packing of the two compounds is remarkably similar. The asymmetric unit contains half of the porphyrin molecule (Figure S4). Four nitrogen atoms of the tetrapyrrolic macrocycle lie in the same plane, and the macrocycle is essentially planar (Figure 3), with the maximum deviation of *β*‐carbon atoms from the N_4_‐mean plane not exceeding 0.250 Å, and those of the *meso*‐carbons below 0.146 Å. The C─C and C─N bond length lies in the typical range of porphyrins.

**FIGURE 4:**
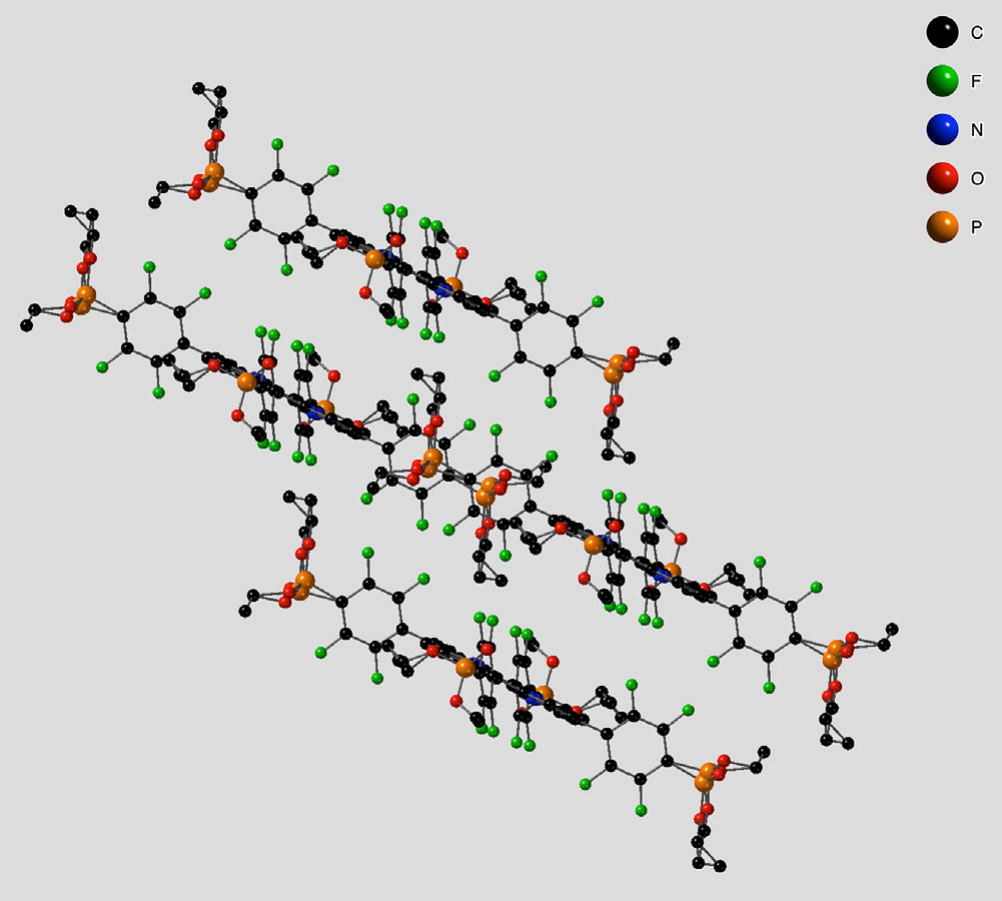
Schematic presentation of molecular packing in the crystals of H_2_TF_4_PPP. Minor disordered parts, solvent molecules, and hydrogen atoms are omitted for clarity.

The phenyl substituents at the *meso‐*positions display varying twist angles (56.86°–81.80°; Table S5) relative to the N_4_ plane (Figures 4 and S5). These values closely match those found in the crystal structure of H_2_TPPP (51.11°–87.74°).

As expected, the P─O bond lengths differ between P═O (1.419–1.449 Å) and P─OEt (1.559–1.597 Å) groups (Table S4). While the C─PO_3_ bond lengths (1.815–1.866 Å) fall within the expected range for arylphosphonates, a notable displacement of the two phosphorus atoms from the N_4_ plane is observed (1.095 Å). A similar distortion (1.97 Å) was reported for H_2_TPPP, likely suggesting that the phosphonate substituents contribute significantly to the crystal packing in both solids.

The molecules of H_2_TF_4_PPP are organized in columns along the *a*‐axis, with small spherical voids located above and below the tetrapyrrolic macrocycle (Figures 4 and S5). The accessible void fraction, as determined by Mercury software using a 1.2 Å probe, amounts to ∼17% of the unit cell volume (≈300 Å^3^). In the crystal structure of H_2_TF_4_PPP, the tetrapyrrolic macrocycles of adjacent molecules do not engage in *π–π* stacking interactions, as they are separated by 5.166 Å. In contrast, the corresponding distance in H_2_TPPP crystals is 3.436 Å, which falls within the conventional range for *π–π* stacking [100].

Interestingly, only one fluorine atom from each phenyl substituent is involved in weak F···H─C contacts. In contrast, hydrogen atoms of non‐fluorinated porphyrin H_2_TPPP forms numerous short intramolecular contacts in the crystal. The role of halogen contacts in crystal packing is a hot topic in crystal engineering, and many fluorinated compounds have been synthesized to investigate intermolecular interactions involving fluorine atoms [101, 102]. Four main types of such interactions have been reported: phenyl–perfluorophenyl stacking, C─F···H, F···F, and C─F···*π*(F) contacts [103]. Among them, only the phenyl–perfluorophenyl stacking provides a notable stabilizing energy of approximately 30 kJ mol^−1^, while other observed contacts are generally weak (< 5–7 kJ mol^−1^) [103, 104, 105]. Moreover, these interactions appear to be less common than the corresponding hydrogen atom interaction modes [103, 104]. The structural parameters of H_2_TF_4_PPP studied in this work are consistent with these general trends.

### 2.4 Optical Properties

Electronic absorption spectra of MTF_4_PPP were recorded in CH_2_Cl_2_ and/or MeCN solutions (unless mentioned otherwise). The obtained data are summarized in Table 1 and compared to those of the corresponding non‐fluorinated MTPPP and the parent MTPP porphyrins, which was previously reported in the literature.

**TABLE 1:** Selected photophysical parameters for MTF_4_PPP, MTPPP, and MTPP.

Compound	Solvent	Absorption, *λ*_abs_ (nm) (*ε*×10^−3^ (M^−1^ cm^−1^))	Fluorescence^a^	
			*λ*_f_ (nm) (Q(0,0), Q(0,1)	*Φ*_f_ ^b^
H_2_TF_4_PPP	CH_2_Cl_2_	414 (285), 507 (21), 540 (3), 584 (6), 637 (1)		
	MeCN	409 (275), 505 (19), 540 (2), 580 (6), 635 (0.9)	640, 704	0.03
H_2_TPPP^c^	CH_2_Cl_2_	417, 513, 550, 590, 645		
	MeCN	357 (15), 416 (340), 482 (3), 513 (15), 546 (6), 587 (4), 642 (3)	646, 713	0.09
H_2_TPP^d^	MeCN	417 (575)^e^, 513 (14), 549 (5), 592 (5), 646 (4)	654, 720	0.09^f^
ZnTF_4_PPP	CH_2_Cl_2_	416 (314), 548 (17), 580 (3)		
	MeCN	398 (48), 413 (404), 510 (4), 552 (20), 588 (2)	600, 652	0.02
ZnTPPP^c^	CH_2_Cl_2_	423 (380), 550 (17), 592 (sh)		
	MeCN		604, 657	0.04
ZnTPP^d^	MeCN	420 (661)^g^, 556 (20), 596 (7)	604, 652	0.03^h^
PdTF_4_PPP	CH_2_Cl_2_	409 (162), 519 (14), 553 (10)		
	MeCN	405 (164), 517 (13), 551 (9)	598, 650	<0.01
PdTPPP^c^	CH_2_Cl_2_	415 (275), 522 (25), 554 (3)		
	MeCN	413 (253), 523 (22), 552 (3)	604, 665	<0.01
PdTPP^i^	toluene	417 (257.04), 485 (2.04), 523 (25.70), 554 (1.48)	560, 607	<0.01
InTF_4_PPP	CH_2_Cl_2_	399 (38), 421 (365), 512 (2), 553 (18), 588 (2)		
	MeCN		597, 649	<0.01
InTPPP^c^	CH_2_Cl_2_	404 (40), 426 (617), 518 (3), 559 (21), 598 (8)	603, 657	
	MeCN	403 (36), 423 (1080), 518 (3), 559 (21), 598 (8)	606, 661	0.064
InTPP^c^	CH_2_Cl_2_	403 (35.49), 425 (1176.05), 520 (3.00), 560 (18.83), 599 (8.31)	604, 660	
	ethanol			0.01^j^
H_2_TF_4_PPP‐A	MeCN/H_2_O (1:1 v/v)	409 (291), 505 (17), 537 (2), 579 (6), 632 (1)		
	water^k^	408, 508, 574	636, 697	0.07
H_2_TPPP‐A^c^	MeCN/H_2_O (1:1 v/v)	416 (346), 482 (3), 516 (13), 552 (8), 589 (4), 644 (4)	649, 714	0.11
PdTF_4_PPP‐A	MeCN/H_2_O (1:1 v/v)	405, 516, 551		
	water^k^	403, 517, 551	602, 652	<0.01
PdTPPP‐A^c^	MeCN/H_2_O (1:1 v/v)	415 (213), 463 (6), 523 (17), 556 (2)	564, 607	<0.001
InTF_4_PPP‐M	MeOH	393 (57), 414 (532), 509 (5), 548 (26), 583 (3)	593, 644	<0.01

^a^Emission was excited at 547 nm for all compounds studied in this work.

^b^Fluorescence quantum yields were measured at ambient temperature relative to a solution of ZnTPP in MeCN as a standard.

^c^Ref. [51].

^d^Absorption and emission properties from ref. [106].

^e^Average value of 417 (575) was found from all data previously reported [107], limited to consistent values.

^f^Ref. [108]. Average value 0.14 was calculated from all previously reported data [107], limited to consistent values.

^g^Average value of 420 (661) was found from all previously reported data [107] limited to consistent values.

^h^Ref. [109].

^i^Ref. [108] and [110].

^j^Ref. [111].

^k^A one drop of 0.1 M NaOH was added to solubilize the porphyrin.

All MTF_4_PPP porphyrins display similar absorption profiles, with a Soret band in the range of 400–430 nm and Q bands spanning the 500–650 nm range, in agreement with previously reported spectra of MTPPP and MTPP (Figure S6). Notably, the molar extinction coefficient in the maximum of the Soret band was significantly lower for H_2_TF_4_PPP and PdTF_4_PPP than for their non‐fluorinated analogues H_2_TPPP and PdTPPP. This was a result of a significant band broadening (Figures S13 and S14). These observations suggest that neither fluorination nor phosphonate substitution at the *meso*‐phenyl groups significantly affects the electronic transitions of the MTPP core.

All newly investigated compounds show negligible solvatochromism, a classical feature of symmetric porphyrins [111], and maintain linear evolution in absorption with concentration within the studied range (Figures S7–S9). This spectral behavior indicates the absence of aggregation *via* an axial coordination of the PO_3_Et_2_ groups to the central metal ions under these conditions.

H_2_TF_4_PPP‐A and PdTF_4_PPP‐A were soluble in slightly basified aqueous solution (pH > 8) and their light absorption was examined under these conditions. No evidence of aggregation was again detected: absorption energy maxima remained constant, and a good linear relationship between absorption and concentration was maintained across the accessible concentration range (up to 10^−^^4^ M) (Figures S11 and S12). Finally, a similar behavior was observed for the partially hydrolyzed complex InTF_4_PPP‐M bearing ethyl phosphonate groups, studied in MeOH solution.

Emission properties of MTF_4_PPP were examined in aerated MeCN performing excitation in the Q‐bands. All compounds showed the S_1_→S_0_ fluorescence with two distinct bands (Q(0,0) and Q(0,1)) typical of MTPP derivatives (Figure S15). Metal insertion significantly decreased the fluorescence quantum yields, consistent with an enhancement of spin–orbit coupling and thus intersystem crossing (ISC) efficiency through so‐called “the heavy‐atom effect”. It appears that fluorination of MTPPP has a negligible impact on this process, as the *Φ*_f_ values of MTF_4_PPP closely match those of the MTPPP analogues. Hydrolysis of the diethyl phosphonate groups in MTF_4_PPP also has little effect on the emission properties, even though a straightforward comparison has little relevance since spectra of MTF_4_PPP‐A and MTF_4_PPP were recorded in different solvents. The porphyrin H_2_TF_4_PPP‐A exhibits *Φ*_f_ as high as 0.07 in aqueous solutions, even though water‐soluble porphyrins typically display low emissivity in this solvent due to aggregate‐induced quenching.

Next, H_2_TF_4_PPP, PdTF_4_PPP, and ZnTF_4_PPP were evaluated as PS by monitoring the intensity of photoinduced ^1^O_2_ phosphorescence signal at 1270 nm in chloroform upon photoexcitation of each compound in their Soret band. These experimental conditions allow for evaluation of singlet oxygen quantum yields (*φ*_Δ_) in comparison with the reference compound phenalen‐1‐one (**PH**) (*φ*_Δ_ = 0.95 [112]) (Figure S16). Moreover, corresponding MTPPP compounds were studied under the same conditions to assess the effect of fluorine substitution on PS efficiency. Data obtained in these studies are gathered in Table 2.

**TABLE 2:** Singlet oxygen quantum yields of MTF_4_PPP and MTPPP.^a^

Compound	Solvent	*φ*_Δ_	Reference
H_2_TF_4_PPP	CHCl_3_	0.83	This work
H_2_TPPP	CHCl_3_	0.82	This work
	CD_3_CN/D_2_O (10:1)	0.72	[51]
PdTF_4_PPP	CHCl_3_	0.97	This work
PdTPPP	CHCl_3_	1.1	This work
	CD_3_CN/D_2_O (10:1)	0.94	[51]
ZnTF_4_PPP	CHCl_3_	0.9	This work

^a^Comparative studies by singlet oxygen phosphorescence measurements in chloroform using **PH** as a standard. Quantum yield values are given with 10% error.

As a first remark, all studied compounds, regardless of the presence of metal ions or peripheral phenyl substitution pattern are efficient PS with *φ*_Δ_ systematically exceeding 0.7. Within this series of compounds, the non‐fluorinated Pd(II) complex PdTPPP exhibited the highest *φ*_Δ_, with a calculated value even slightly exceeding the theoretical maximum of 1, yet within the 10% range of measurement error generally associated with singlet oxygen measurements.^1^ Fluorination of PdTPPP slightly lowered *φ*_Δ_ to 0.97, while the noble‐metal‐free complex ZnTF_4_PPP was only slightly less efficient (*φ*_Δ_ = 0.90). The free‐base porphyrins H_2_TPPP and H_2_TF_4_PPP showed lower *φ*_Δ_ values (0.82 and 0.83, respectively) compared to Pd(II) and Zn(II) complexes, highlighting the role of the heavy‐atom effect in enhancing ISC. Nevertheless, their *φ*_Δ_ values were still substantially higher than those reported for most free‐base porphyrins (*φ*_Δ_ = 0.50–0.60 for H_2_TPP [113]).

### 2.5 Photostability in Solution

The photostability of free‐base porphyrin H_2_TF_4_PPP and its Zn(II) and Pd(II) complexes was investigated in CH_2_Cl_2_, MeCN, and MeOH, and compared to that of the corresponding non‐fluorinated MTPPP analogues. As discussed in our previous work [114], comparative studies of the dyes' photostability must be interpreted with caution, as experimental conditions cannot be made fully identical for dyes under consideration. Both the solution concentration and the amount of absorbed light (determined by the molar extinction coefficient of each dye within the emission range of the selected LED) strongly influence the apparent rate of dye photobleaching. These parameters cannot be simultaneously matched across the dyes studied. Additionally, the photodegradation of porphyrins is a complex process involving radical intermediates and multiple competing pathways, each of which is strongly influenced by the nature of the peripheral substituents and probably by the light intensity. Consequently, a comprehensive evaluation of porphyrin photostability requires systematic spectroscopic monitoring until the complete disappearance of the dye across a broad range of solvents, concentrations, and illumination conditions.

In this work, photostability was evaluated under a few representative conditions using solutions of porphyrins in common organic solvents and with identical absorbance at the Soret band maximum. The first series of experiments was conducted using a blue LED (450 nm) with a power of 3 W, commonly used in photocatalysis. Solutions of MTF_4_PPP and MTPPP in CH_2_Cl_2_, MeOH and MeCN were stirred for several days and monitored periodically by UV–vis spectroscopy. As shown in Figures S17–S19, the free‐base porphyrin and the Pd(II) complex of the MTF_4_PPP series exhibited remarkable photostability in all solvents, with only minor decreases (less than 2%) in absorbance after 3 d of irradiation. Analogous compounds of the MTPPP series also displayed high stability under these conditions, showing less than 10% change in absorbance. The improvement in photostability after fluorination was particularly pronounced for the Zn(II) complexes, as ZnTPPP was considerably less stable than the free‐base porphyrin H_2_TPPP and its Pd(II) complex (likely due to its high tendency for axial coordination of solvent molecules). A relatively slow degradation of ZnTPPP was observed in MeOH only, with absorbance decreasing by approximately 25% after 72 h of irradiation. ZnTF_4_PPP was more stable under the same conditions, showing a 3% decrease in absorbance. This difference in photostability was even more pronounced in MeCN and CH_2_Cl_2_. While ZnTPPP was completely decomposed after 24 h of irradiation in CH_2_Cl_2_, ZnTF_4_PPP retained 60% of its absorbance after 72 h. In MeCN, ZnTF_4_PPP was more stable, showing only a 20% decrease in absorbance, whereas 90% of ZnTPPP had decomposed after 24 h.

**FIGURE 5:**
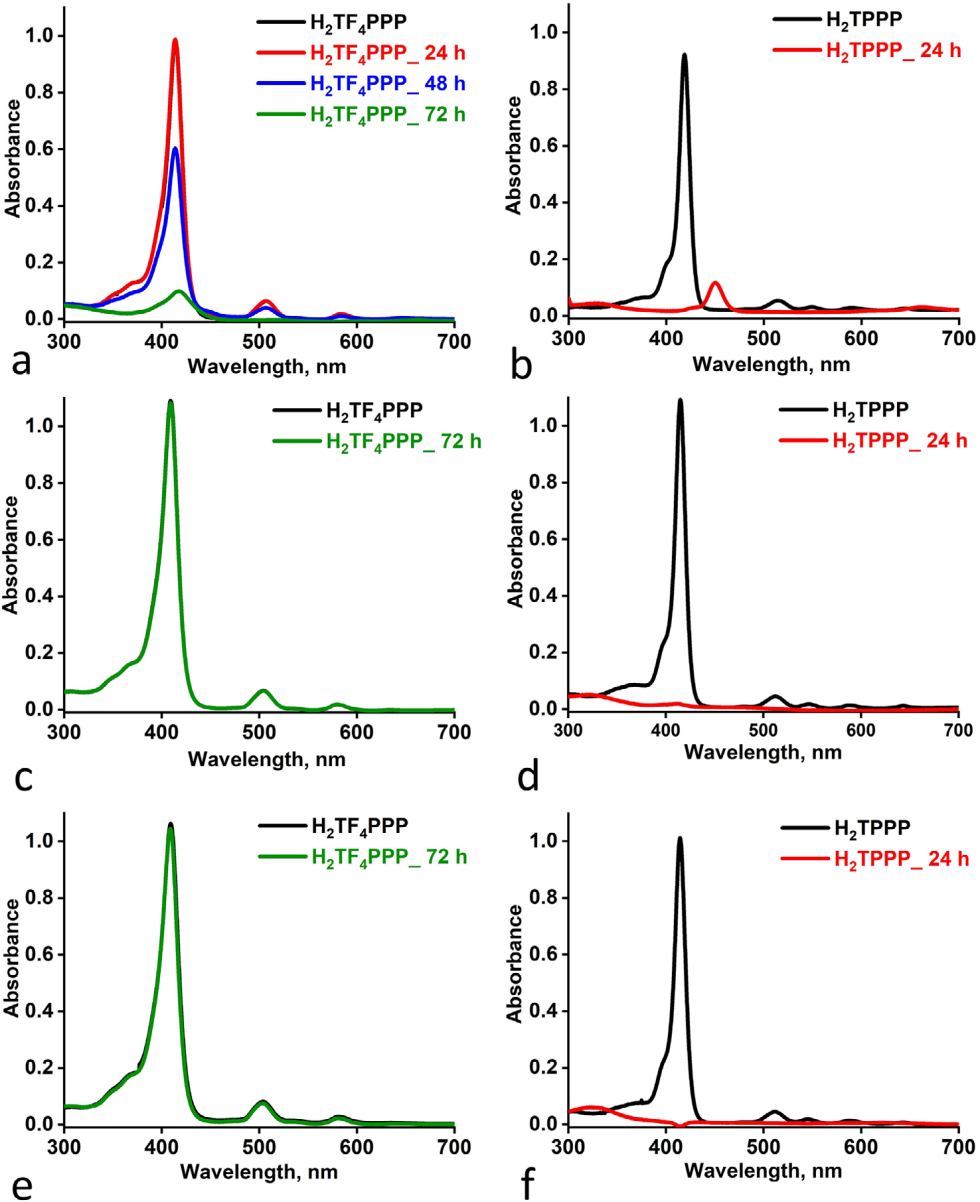
Comparative UV–vis studies of photostability of H_2_TF_4_PPP and H_2_TPPP under irradiation with blue LED (425 nm, 18 W) in CH_2_Cl_2_ (a and b, respectively), MeCN (c and d, respectively), and MeOH (e and f, respectively). The bathochromic shifts of the Soret bands upon irradiation in CH_2_Cl_2_ are likely induced by protonation of the studied compounds [117].

The most striking result was obtained when free‐base porphyrins were irradiated with a more powerful blue LED (425 nm, 18 W) included in the standard EvoluChem Photoredox setup. As shown in Figure 5, non‐fluorinated free base porphyrin H_2_TPPP exhibited poor resistance to irradiation in all solvents studied, showing a complete decomposition within 24 h. In contrast, H_2_TF_4_PPP displayed less than 2% loss of Soret band absorbance after 72 h of irradiation in MeCN and MeOH. In dichloromethane, a solvent known for its low photostability and tendency to generate free radicals that may accelerate chromophore photobleaching, H_2_TF_4_PPP was stable during the first 24 h and underwent complete degradation only within 72 h.

The positive effect of *ortho*‐halogen substituents on the chemical stability of porphyrin catalysts in oxidation reactions has been widely discussed [115]. In contrast, studies on the influence of these substituents on photostability remain limited. Recently, the benefits of *ortho*‐halogen substitution were reported for bacteriochlorins [116], and our work corroborates these findings. While the photostability of ZnTPPP remains relatively low even after fluorination, the free‐base porphyrin H_2_TF_4_PPP and PdTF_4_PPP demonstrate exceptionally high photostability.

### 2.6 Photocatalysis

Given the high photostability and excellent singlet oxygen generation efficiency, the MTF_4_PPP porphyrins rank among the most effective PS and hold strong potential for practical applications. In line with our research interests, we demonstrated their performance in photocatalysis. In particular, we explored the replacement of PdTPPP which was previously identified by our group [51] as a highly efficient photocatalyst (PC) for the aerobic oxidation of sulfides to sulfoxides by the noble‐metal‐free analogue H_2_TF_4_PPP. This reaction is valuable for organic synthesis, especially for late‐stage functionalization of pharmaceuticals [118, 119]. It also provides important insight into PC performance in the reactions proceeding through energy transfer (EnT) or electron transfer (ET, photoredox) mechanisms. Depending on the structure of the sulfide, oxidation may proceed mainly through ET, where the PC transfers energy to molecular oxygen to generate singlet oxygen, or through electron transfer involving the ET to either sulfide or molecular oxygen [120, 121, 122, 123, 124]. As a result, PC efficiency can vary greatly among different sulfides, and a comparison of their reactivity provides a useful insight into PC performance in both types of photocatalytic processes.

This study was focused on the selectivity and oxidation kinetics of dibutyl sulfide and three electronically different aryl methyl sulfides, namely thioanisole, 4‐chloro‐ and 4‐nitrothioanisole, using H_2_TF_4_PPP as a PC. These substrates were selected due to their tendency to undergo oxidation *via* distinct mechanistic pathways: dibutyl sulfide reacts predominantly through an EnT pathway, whereas aryl methyl sulfides undergo oxidation through a combination of EnT and ET processes.

In a typical experiment, a solution of selected sulfide and PC (0.005–0.01 mol%) in MeCN/H_2_O (10:1 v/v) was stirred while gently bubbling oxygen through the solution irradiated with a blue LED (450 nm, 3 W). Performing the reactions under these conditions afforded direct comparison with our previously reported results, which were obtained using PdTPPP and H_2_TPPP as PC [51]. The results of these studies, together with previously reported data for PdTPPP, are summarized in Table 3. As shown in this Table and Figure S21, PdTPPP and H_2_TF_4_PPP exhibited comparable efficiency in the oxidation of dibutyl sulfide (entries 1 and 2) and thioanisole (entries 3 and 4). All reactions proceeded with high selectivity, and replacing PdTPPP by H_2_TF_4_PPP completely suppressed sulfone formation in the oxidation of thioanisol. In contrast, the free‐base porphyrin H_2_TF_4_PPP proved more efficient in the sulfoxidation of electron‐deficient aryl methyl sulfides (entries 5–8), with the largest difference observed for 4‐nitrothioanisole, a substrate known for its low reactivity toward singlet oxygen. Notably, the catalyst replacement did not compromise the selectivity of these reactions, and both sulfoxides were obtained in nearly quantitative yield. Considering that the efficiency of PdTPPP in the studied reactions is higher than that of the fluorinated complex PdTF_5_PP, the free‐base porphyrin H_2_TF_4_PPP can be regarded as a cost‐effective and sustainable alternative to noble‐metal porphyrin complexes.

**TABLE 3:** Photooxidation of sulfides in the presence of H_2_TF_4_PPP and PdTPPP.^a^

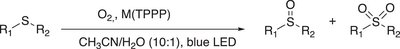							
Entry	Sulfide	Photocatalyst (mol%)	Time (h)	Conversion^b^ (%)	Yield^b^ (%)		Reference
					Sulfoxide	Sulfone	
1	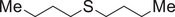	H_2_TF_4_PPP					This work
		(0.005)	4	100	99	1	
2		PdTPPP					[51]
		(0.005)	4	100	100	0	
3	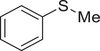	H_2_TF_4_PPP					[51]
		(0.05)	3	100	100	0	
4		PdTPPP					
		(0.05)	3	100	98	2	
5	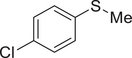	H_2_TF_4_PPP					This work
		(0.05)	2.5	100	100	0	
		(0.025)	3.5	100	100	0	
6		PdTPPP					[51]
		(0.025)	4.5	100	100	0	
7	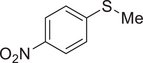	H_2_TF_4_PPP					This work
		(0.05)	8	100	99	1	
8		PdTPPP					This work
		(0.05)	8	45	100	0	
9	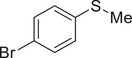	H_2_TF_4_PPP					This work
		(0.025)	4	100	99.5	0.5	
10	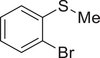	H_2_TF_4_PPP					This work
		(0.05)	7	100	98	2	
11	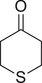	H_2_TF_4_PPP					This work
		(0.01)	4	100	100	0	
12	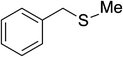	H_2_TF_4_PPP					This work
		(0.005)	6	100	100	0	
13^c^	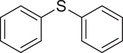	H_2_TF_4_PPP					This work
		(0.05)	4	98	100	0	

^a^Reaction conditions: 0.5 mmol of sulfide and PC were stirred and irradiated by blue LED (450 nm, 3 W) in MeCN/H_2_O mixture (2.75 mL, 10:1 v/v) bubbling slowly molecular oxygen at room temperature.

^b^Conversion and selectivity were determined by ^1^H NMR analysis of reaction mixtures using biphenyl as an internal standard.

^c^The reaction was conducted in an EvoluChem Photoredox box using a blue LED (425 nm, 18 W) under an oxygen atmosphere (1 L balloon).

Next, we explored the substrate scope using H_2_TF_4_PPP as a PC. All studied sulfides were selectively and nearly quantitatively converted to the corresponding sulfoxides, although the required reaction times varied significantly (entries 9–13). While 4‐bromothioanisole was fully converted within 4 h, the more sterically hindered 2‐bromothioanisole required 7 h of irradiation (entries 9 and 10). The cyclic thian‐4‐one showed lower reactivity than aryl methyl sulfides. Nevertheless, complete oxidation was achieved within 4 h when the PC loading was increased to 0.01 mol% (entry 11). Benzyl methyl sulfide, which is prone to side‐product formation under photocatalytic oxygenation, was also oxidized to the corresponding sulfoxide in nearly quantitative yield (entry 12). Finally, diphenylsulfide, which is known for its high photostability due to its steric hindrance and low nucleophilicity, was selectively oxidized in 4 h using a more efficient LED (425 nm, 18 W) (entry 13).

Interestingly, the free‐base H_2_TF_4_PPP exhibited slightly higher efficiency in these reactions compared to the non‐fluorinated analogue H_2_TPPP (Table S7), despite their comparable singlet oxygen generation capabilities and high photostability in acetonitrile under irradiation with a 3 W blue LED (Figure S18). To better account for the results, we compared the photostability of these PC under conditions simulating the photocatalytic reactions, namely in MeCN/H_2_O (10:1 v/v) at equal concentrations. As shown in Figure S20, the photostability of H_2_TF_4_PPP in this solvent mixture exceeded that of the non‐fluorinated PC. This enhanced photostability may account for its slightly higher efficiency in the reaction studied in this work.

Thus, the free‐base H_2_TF_4_PPP can be regarded as one of the most efficient metal‐free PC known [119, 125] for the oxidation of sulfides to sulfoxides using molecular oxygen as the terminal oxidant. This compound is also promising for the optimization of other photocatalytic reactions proceeding either through EnT or ET.

### 2.7 Behavior of H_2_TF_4_PPP at the Interfaces

Fluorinated porphyrins are widely studied as functional components of molecular materials. The Langmuir–Blodgett (LB) /Langmuir–Schaefer (LS) technique is useful for the preparation of functional films, offering precise control over molecular orientation and thickness through the stepwise transfer of ordered monolayers from the air–water interface to solid substrates. However, the use of fluorinated *meso*‐tetraarylporphyrins in LB film fabrication remains limited. These compounds typically exhibit a marked tendency to aggregate at the air–water interface rather than form stable Langmuir monolayers. To our knowledge, among fluorinated porphyrins, only chromophores containing long perfluoroalkyl chains have been reported to form monolayers at the interface [126], whereas *meso‐*tetrakis(pentafluorophenyl)porphyrin fails to produce a Langmuir monolayers [127]. Numerous studies on mixed Langmuir monolayers of such porphyrins with fatty acids, long‐chain alkylamines, or phospholipids [17, 127, 128, 129] further highlight the challenge of obtaining stable monolayers. Promising results have been achieved only when the LB technique was combined with a self‐assembly methodology based on the formation of covalent Zr─O(P) bonds [17, 18]. In contrast, LS films of fluorinated phthalocyanines have already found applications as sensors [130].

Recent studies have shown that the introduction of hydrophilic substituents into *meso*‐arylporphyrins markedly facilitates the formation of stable Langmuir monolayers from these molecules [131, 132, 133, 134, 135, 136, 137]. We hypothesized that the incorporation of PO_3_Et_2_ groups into fluorinated *meso*‐tetraarylporphyrins would stabilize their Langmuir monolayers and investigated behavior of free base porphyrin H_2_TF_4_PPP at the air–water interface and on solid substrates.

**FIGURE 6:**
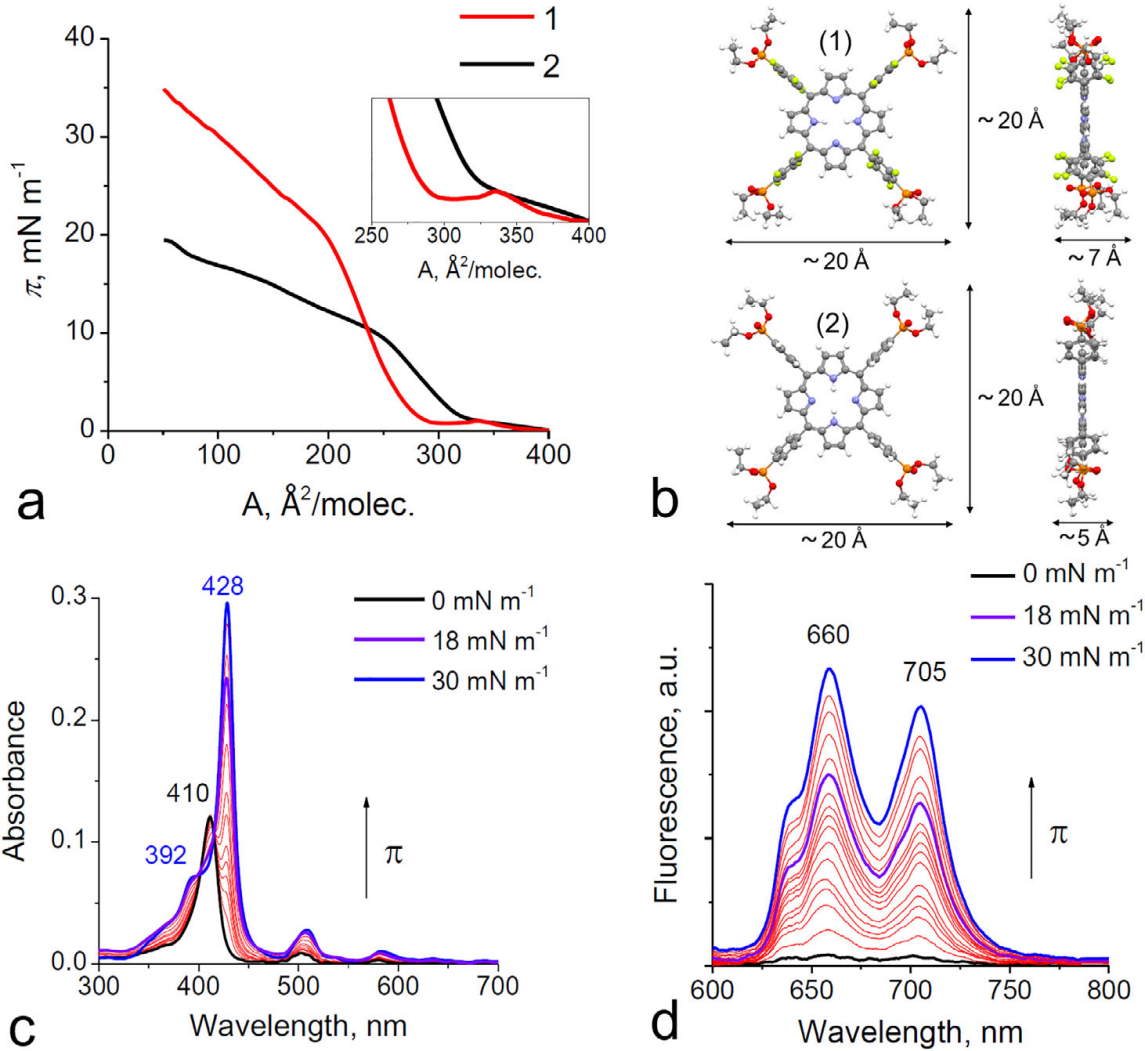
Surface pressure–area isotherms of H_2_TF_4_PPP (a, *1*) and H_2_TPPP (a, *2*) monolayers. Spacefill model of H_2_TF_4_PPP (b, *1*) and H_2_TPPP (b, *2*) molecules optimized by the semi‐empirical PM6 method [138]. In situ UV–vis (c) and fluorescence (*λ*_ex_ = 420 nm) (d) spectra of H_2_TF_4_PPP monolayer.

In preliminary experiments, we confirmed that H_2_TF_5_PP does not form a stable Langmuir monolayer under experimental conditions used in this work (Figure S22), in agreement with previous reports [126]. In contrast, H_2_TF_4_PPP yielded such monolayer, similar to its non‐fluorinated analogue H_2_TPPP [54]. The surface pressure–area (*π*–*A*) isotherms shown in Figure 6a indicate that, at low pressures, the molecules of both H_2_TF_4_PPP and H_2_TPPP adopt a face‐on orientation at the water surface, occupying approximately 400 Å^2^ per molecule. This value is in good agreement with computational estimates obtained using the Spartan package [138] (Figure 6b). Notably, limiting molecular areas of H_2_TF_4_PPP and H_2_TPPP significantly exceeded those of non‐phosphonated *meso*‐tetraarylporphyrins (55–125 Å^2^ per molecule) [134, 135, 139, 140, 141] indicating low density of the molecular packing in the studied monolayers. The compression isotherms of both porphyrins exhibit distinct regions, suggesting structural reorganization within the monolayers upon compression (Figure 6a). As the surface pressure increases, the porphyrin molecules change their orientation, becoming tilted relative to the water surface and/or forming discrete molecular aggregates. The monolayer of H_2_TF_4_PPP shows a higher compressibility modulus (*C*_s_ *^−1^*) than that of H_2_TPPP (Figure S23), revealing a greater degree of molecular organization after the introduction of fluorine atoms. Additionally, an increase in collapse pressure is observed upon this functionalization, indicating an enhanced stability of the fluorinated monolayer. These results are somewhat unexpected, as fluorine atoms do not engage in strong intermolecular interactions in the crystals of H_2_TF_4_PPP and *π–π* stacking of the tetrapyrrolic macrocycles is not observed in these crystals, as discussed above. It appears that the structural organization of the H_2_TPPP monolayer, as its crystal packing, is dominated by intermolecular interactions in which PO_3_Et_2_ groups and hydrogen atoms of the phenyl rings are involved. In contrast, *π–π* stacking plays a more significant role in stabilizing the H_2_TF_4_PPP monolayer, despite the larger size of fluorine substituents compared to hydrogen atoms and absence of such interactions in the crystals.

To gain deeper insight into the structure of the H_2_TF_4_PPP monolayer, *in situ* UV–vis spectra were recorded during monolayer compression. Immediately after chloroform evaporation (*π* = 0 mN m^−^^1^), a Soret band was observed at 410 nm. This value agrees well with that measured for H_2_TF_4_PPP in ethanol solution (Figure S24), indicating that the porphyrin molecules do not form aggregates under these initial conditions. Upon increasing the surface pressure, a new band emerges at 428 nm, indicating formation of *J*‐aggregates (Figure 6c).

Despite observed aggregation in the studied monolayer, the porphyrin molecules remained emissive (Figure 6d). The fluorescence spectrum displayed three bands with maxima at 648, 660, and 705 nm, whereas the spectrum of H_2_TF_4_PPP in ethanol showed the typical porphyrin pattern with only two bands (648 and 712 nm). Studies of the emission properties of H_2_TF_4_PPP in ethanol/water solvent mixture (1:9 v/v), where the compound exhibited limited solubility, revealed that the band at 660 nm corresponds to electronic transitions associated with *J*‐aggregates (excimers) (Figure S24).

In accordance with these data, a compression–expansion cycle of the H_2_TF_4_PPP monolayer displays a pronounced hysteresis (Figure S25a). In situ UV–vis spectra recorded during monolayer expansion show that H_2_TF_4_PPP does not fully revert to its monomeric form at *π* = 0 mN m^−^^1^ since the band at 428 nm is still observed, albeit being of low intensity (Figure S25b). When the monolayer was compressed again, the resulting isotherm differed from that obtained in the first cycle, although no porphyrin molecules were detected in the water subphase (Figures S25a and S26).

**FIGURE 7:**
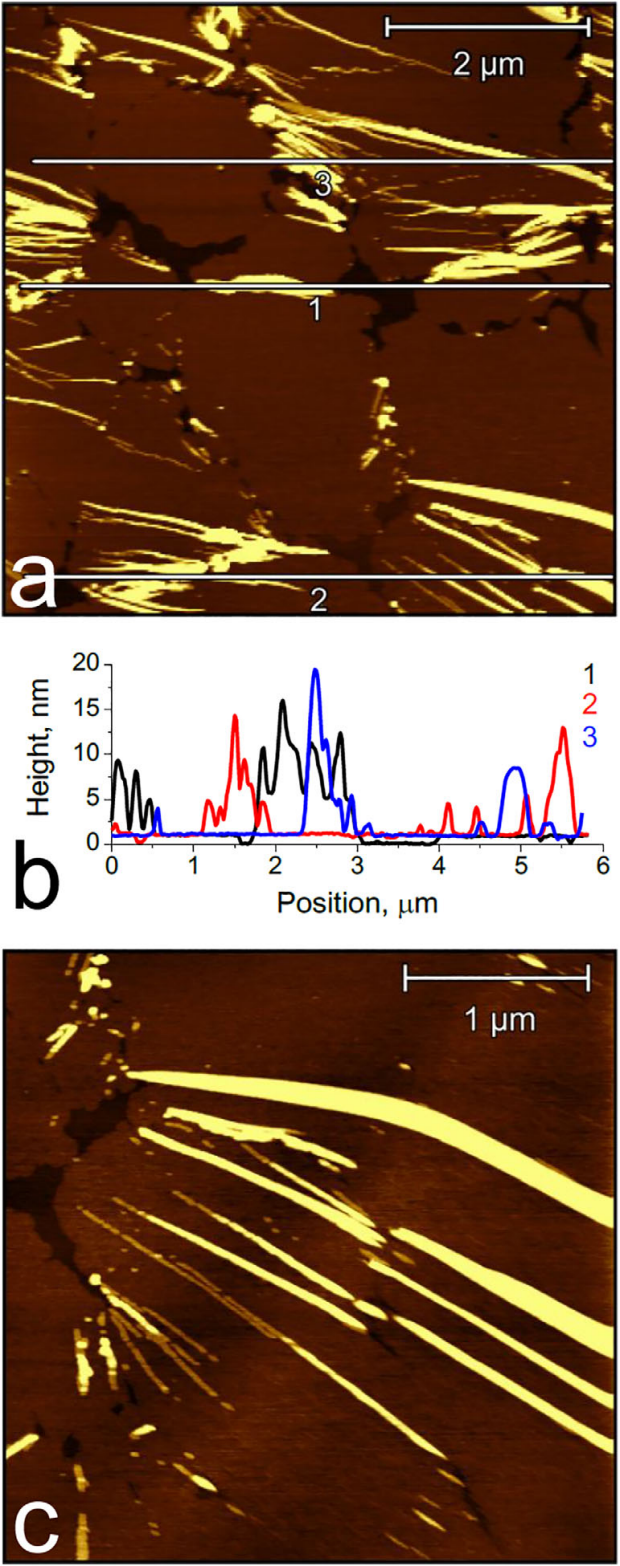
AFM images of single‐layer LS film of H_2_TF_4_PPP (a, c) deposited onto a mica substrate at a surface pressure of 18 mN m^−1^ and corresponding height profiles (b).

To further evaluate the practical utility of these Langmuir monolayers, we studied their transfer onto hydrophilic (mica) and hydrophobic (poly(vinyl chloride), PVC) solid substrates using the horizontal dipping (LS technique) at a surface pressure of 18 mN m**^−^** ^1^. The morphology of single‐layer film transferred onto a mica surface was examined by atomic force microscopy (AFM) (Figure 7). The studied surface was uniformly covered by a porphyrin monolayer with a height not exceeding 1 nm and large porphyrin nanofibers with heights of 10–20 nm.

The optical properties of the single‐layer LS film were studied after transfer of the Langmuir monolayer onto a transparent PVC substrate. The position of the Soret band (Figure S27a) closely matched that observed for the monolayer at the water surface (Figure 6c). In the fluorescence spectrum, the overall band shape was also preserved, although a red shift of 5–10 nm was observed for the band maxima, likely due to changes in the surrounding environment (Figure S27b). These results revealed that the removal of water does not significantly alter the supramolecular interactions within this stable single‐layer film.

**FIGURE 8:**
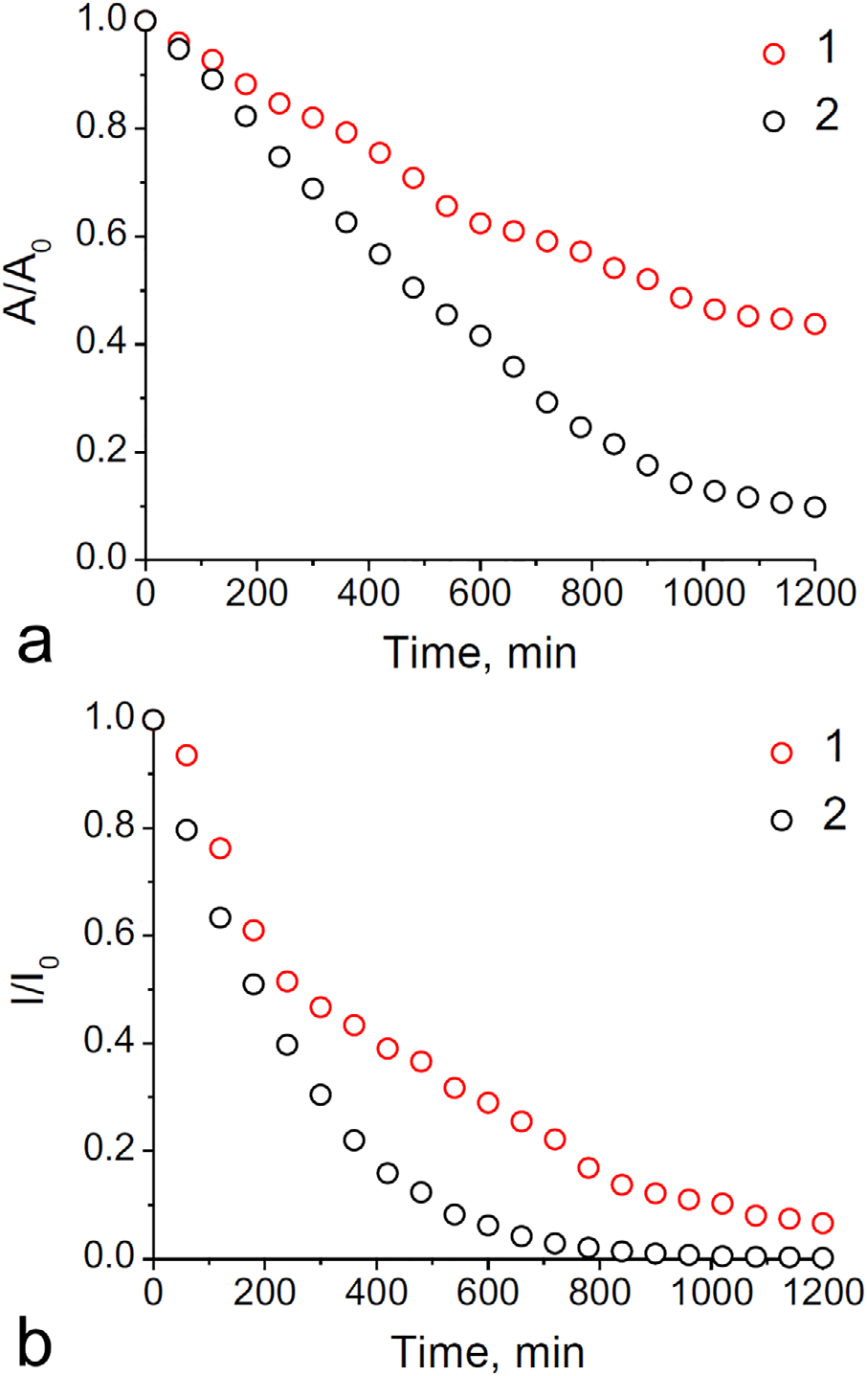
Photodegradation of single‐layer LS films of H_2_TF_4_PPP (*1*) and H_2_TPPP (*2*) deposited onto a PVC at 18 mN m**^−^** ^1^ under irradiation using LED (365 nm, 3 W): a) the absorbance (A) changes over time (H_2_TF_4_PPP (428 nm) and H_2_TPPP (430 nm)); b) the fluorescence (I) changes (H_2_TF_4_PPP (710 nm) and H_2_TPPP (720 nm)) over time (*λ*_ex_ = 420 nm).

The photobleaching of H_2_TF_4_PPP and H_2_TPPP films deposited on PVC substrates was investigated by irradiating the films with a 365 nm LED (3 W) and regularly recording their UV–vis and fluorescence spectra. The absorption and emission band shapes changed in parallel with a decrease in band intensities (Figure S28). These changes in optical properties can be explained by different rates of photobleaching of non‐uniform porphyrin aggregates or a structural reorganization of the studied films under irradiation. As shown in Figure 8, the results obtained by both spectroscopic techniques are consistent and reveal that the H_2_TPPP monolayer decomposes significantly faster than the fluorinated LS film.

To further confirm the superior photostability of the H_2_TF_4_PPP film, two studied films were irradiated using the xenon lamp (150 W, excitation slits opening of 5 nm) in the spectrofluorometer. Continuous irradiation at 430 nm was performed for 60 min, with regular monitoring of the Q(0,1) band intensities (710 and 720 nm for H_2_TF_4_PPP and H_2_TPPP, respectively) (Figure S29). Consistent with the LED‐irradiation experiments, the H_2_TF_4_PPP film exhibited greater photostability than non‐fluorinated thin film.

Thus, the results of these studies demonstrate that fluorination of H_2_TPPP plays a key role in the structural organization of Langmuir monolayers of phosphonated porphyrins. The thin films thus obtained are highly emissive, and their photostability surpasses that of H_2_TPPP films.

## 3 Conclusion

This work was aimed at the synthesis of *meso*‐tetraarylporphyrins bearing both hydrophilic phosphonate groups and strongly electron‐withdrawing fluorine substituents at the phenyl substituents of the tetrapyrrolic macrocycle. We developed a synthetic approach to *meso*‐tetra[4‐(diethoxyphosphoryl)‐2,3,5,6‐tetraflouorophenyl]porphyrins and briefly investigated their coordination, optical, and catalytic properties. Our studies of the preparation of photoactive complexes have demonstrated that the presence of fluorine substituents may alter the reactivity of these compounds toward metal salts. Zn(II) and Pd(II) complexes were prepared in high yields under mild reaction conditions. However, insertion of metal ions exhibiting strong Lewis acidity, such as In(III) ions, was accompanied by partial hydrolysis of phosphonate groups. This limitation may, in certain cases, be beneficial, facilitating the synthesis of water‐soluble porphyrins, as exemplified here by the synthesis of InTF_4_PPP‐M. The free‐base porphyrin H_2_TF_4_PPP and its Zn(II) and Pd(II) complexes efficiently generate singlet oxygen and exhibit good solubility in common organic solvents. The noble‐metal‐free porphyrin H_2_TF_4_PPP surpasses PdTPPP in photocatalytic performance, enabling more sustainable selective oxidation of sulfides to sulfoxides with molecular oxygen in MeCN/H_2_O solvent mixture.

The structural features of these molecules (such as their high symmetry, electron‐deficient character, and the presence of four phosphonate groups) make them valuable for optimizing sensors, catalysts and PS, as well as for developing stable functional materials, including porous sol–gels based on metal oxides, MOFs, and structured thin films on solid supports. In this work, we have demonstrated their unique role in the formation of LS films, an important class of functional materials for sensing and photonic applications. To our knowledge, this is the first family of *fluorinated meso‐*tetraarylporphyrins that forms stable Langmuir monolayers at the water surface, which can be transferred onto solid supports, yielding highly photostable thin films.

Thus, compounds developed in this work hold promise for advancing porphyrin chemistry, with potential contributions to sustainable photocatalysis, sensing technologies, PDT and so forth.

## Conflicts of Interest

The authors declare no conflicts of interest.

## Supporting information



Detailed experimental materials and methods as well as theoretical data can be found in the Supporting Information. The authors have cited additional references within the Supporting Information [142, 143, 144, 145, 146, 147, 148, 149, 150]. CCDC 2520463 contains the supplementary crystallographic data for this paper. These data can be obtained free of charge from The Cambridge Crystallographic Data Centre via www.ccdc.cam.ac.uk/structures.

## Data Availability

The data that supports the findings of this study are available in the supplementary material of this article

## Footnotes

1 Errors are generally exacerbated in porphyrin dyes, due to the sharp profile of the very intensive Soret band associated to the wide opening of the excitation slits required to maximize singlet oxygen emission intensity leading to possible misestimation of the real absorbance.
