# Supplementary material for: Synthesis and Functional Properties of Fluorinated Phosphonate‐Substituted Porphyrins: Excellent Photosensitizers and Robust Precursors for Photoactive Langmuir–Schaefer Films
Source: Chemistry. 2026 May 15;32(34):e71118. doi: 10.1002/chem.71118 (PMC13569938; doi:10.1002/chem.71118)
Supplement: Supplementary file 1 [file CHEM-32-e71118-s001.pdf]

Supporting Information

1. Materials and methods S2
2. Synthesis of porphyrin H_2TF_4PPP S3
3. Synthesis of metalloporphyrin MTF_4PPP and $InTF_4PPP$-M S7
4. Synthesis of porphyrins MTF_4PPP-A S9
5. DFT calculations S11
6. X-ray crystallography of H_2TF_4PPP S14
7. Optical properties S21
8. Studies on the single oxygen generation S26
9. Photostability in solution S28
10. Photocatalysis S32
11. Interfacial behavior of H_2TF_4PPP S34
12. Spectral characterization of newly synthesized compounds S40

1. Materials and methods

Materials

All reagents and solvents were acquired from commercial unless otherwise noted. Deionized high-purity water (18.2 MΩ cm) was obtained from a Purelab Option (USF Elga) water purification system. Pyrrole was dried over Na_2SO_4 and distilled under Ar. *N*-methyl-2-pyrrolidone (NMP) was dried with molecular sieves (4 Å).

5,10,15,20-Tetrakis(4-bromo-2,3,5,6-tetrafluorophenyl)porphyrin (H_2TBrF_4PP) and [5,10,15,20-tetrakis(4-bromo-2,3,5,6-tetrafluorophenyl)porphyrinato]zinc ($ZnTBrF_4PP$) were prepared using previously published procedures [83].

Methods

Analytical thin-layer chromatography (TLC) was performed using Merck silica gel 60 F-254 plates (precoated sheets, 0.2 mm thick, with fluorescence indicator F254). The spots were visualized either directly or through illumination with a UV lamp (λ = 254/365 nm). Column chromatography purification was carried out on silica gel (silica gel for flash chromatography, 40–63 μm, VWR).

Spectroscopic measurements

^{1}H, ^{19}F, ^{31}P, and ^{13}C NMR spectra were acquired on a Bruker Avance III 300 MHz spectrometer at room temperature (at 300, 282, 121, and 75 MHz, respectively). Chemical shifts are given in parts per million (ppm), referenced on the δ scale by using residual non-deuterated solvent signals as internal standard for ^{1}H and ^{13}C NMR spectroscopies; ^{31}P and ^{19}F NMR spectra were calibrated using referencing *via* direct measurement of the absolute frequency lock signal, provided by the ^{2}H resonance of deuterated solvent. The coupling constants are expressed in units of frequency (Hz). In the description of the ^{13}C NMR spectra of porphyrins, the carbon atoms of the phenyl residue attached to the *meso*- position of the macrocycle are labelled as first, while the opposite carbon atoms of this residue are labelled as fourth. This numbering scheme was also used in the description of the ^{19}F NMR spectra. FT-IR spectra were registered on FT-IR Perkin Elmer Spectrum 65 spectrophotometers using an ATR accessory. The UV–vis spectra were recorded using a Jasco V-550 spectrophotometer in rectangular quartz cells (Hellma, 100-QS, 45 × 12.5 × 12.5 mm, light path 10 mm, chamber volume 3.5 mL). Fluorescence spectroscopic studies were performed using a HORIBA Jobin Yvon Fluorolog spectrophotometer (software FluorEssence) with a standard fluorometer cell (Labbox, LBQ, 10 mm). Emission spectra were recorded after excitation at the corresponding wavelength (shutter: Auto Open, excitation slit = 3 nm, and emission slit = 3 nm). All fluorescence spectra were corrected.

Mass spectrometry

Accurate (HR) mass measurements were recorded on a MicrOTOFQ II (Bruker) apparatus equipped with an electrospray ionization (ESI) source. A $CHCl_3$/MeOH (1:1 v/v) solution was used for the analysis, except MTF_4PPP-A, which was analysed in MeOH.

ChatGPT was used to improve the English language in this manuscript.

2. Synthesis of porphyrin H_2TF_4PPP

4-Bromo-2,3,5,6-tetrafluorobenzaldehyde (1) [84, 85]. A dried 250 mL two-necked flask equipped with a magnetic stirrer and a back-flow condenser was charged under Ar with pentafluorobenzaldehyde (**1**) (29.00 g, 149 mmol), lithium bromide (18.00 g, 207 mmol) and dried NMP (150 mL). Subsequently, the reaction mixture was stirred and heated at 170 °C (oil bath) for 4 h. After cooling, the reaction mixture was puured under stirring into crushed ice (500 g). The precipitate was filtered off and washed with water (150 mL). The resulting brown solid was dissolved in CH_2Cl_2 (300 mL) and the solution was dried over anhydrous Na_2SO_4. After evaporation of solvent under reduced pressure, the residue was purified by column chromatography (short column) using CH_2Cl_2/pentane (3:1 v/v) as an eluent. The target compound was obtained as a yellow solid in 68% yield (26.00 g).

The spectroscopic data of the obtained product were in agreement with those reported in the literature [84, 85].^{1}H NMR (300 MHz, $CDCl_3$, 298 K, ppm): δ_H = 10.3 (s, 1 H; CHO) ppm. $^{13}C\{^1H\}$ NMR (75 MHz, $CDCl_3$, 298 K, ppm): δ_C = 181.8 (s,1C; CHO), 146.9 (d, $J_{C,F}$ = 265 Hz, 2C; 2-C_{Ph}), 145.1 (d, $J_{C\text{-}F}$ = 256 Hz, 2C; 3-C_{Ph}), 114.4 (s, 1C; 1-C_{Ph}), 107.3 (s, 1C; 4-C_{Ph}) ppm.

2-(4-Bromo-2,3,5,6-tetrafluorophenyl)-1,3-dioxolane (2). Ethylene glycol (40.07 g, 646 mmol, 6.4 equiv.) and *p*-toluenesulfonic acid (0.385 g, 2.2 mmol, 0.02 equiv.) were added to a solution of **1** (26.00 g, 101 mmol) in toluene (350 mL). The mixture was heated at reflux, and water was removed using a Dean–Stark apparatus. After stirring overnight, the reaction mixture was cooled to room temperature, washed with saturated $NaHCO_3$ solution (2 × 50 mL) and water (1 × 50 mL), dried over anhydrous $MgSO_4$, and concentrated under reduced pressure to afford the title compound in 94% yield (28.57 g) as a white solid. M. p. 95–97 °C (EtOH). ^{1}H NMR (300 MHz, $CDCl_3$, 298 K): δ_H = 4.02–4.12 (m, 2H, CH_2), 4.16–4.27 (m, 2H, CH_2), 6.40 (s, 1H, CH) ppm. $^{13}C\{^1H\}$ NMR (75 MHz, $CDCl_3$, 298 K): δ_C = 66.1 (s, 2C, OCH_2), 96.7 (m, 1C, CHO_2), 101.1 (tt, $^2J_{C,F}$ = 14.3 Hz, $^3J_{C,F}$ = 2.2 Hz, 1C, 4-C_{Ph}), 116.7 (t, $^2J_{C,F}$ = 13.5 Hz, 1C, 1-C_{Ph}), 144.9 (dm, $^2J_{C,F}$ = 247.1 Hz, 2C, 3-C_{Ph}), 145.3 (dm, $^2J_{C,F}$ = 253.5 Hz, 2C, 2-C_{Ph}) ppm. ^{19}F NMR (282 MHz, $CDCl_3$, 298 K, ppm): δ_F = −142.9 to −142.8 (m, AA' part of AA'BB' system, 2F, 2-F), −133.3 to −133.2 (m, BB' part of AA'BB' system, 2F, 3-F) ppm. IR (neat): $\tilde{\nu}$ = 2975

(w), 2908 (w), 1642 (m), 1485 (vs), 1475 (vs), 1399 (s), 1278 (m), 1262 (m), 1212 (m), 1148 (m), 1132 (m), 1111 (s), 1023 (m), 992 (s), 971 (vs), 948 (vs), 925 (vs), 765 (s), 729 (m), 641 (s), 617 (w) cm^{-1}. HRMS (ESI^+): *m/z* calcd for $C_9H_6BrF_4O_2$: 302.9462 $[M+H]^+$; found: 302.9463.

Diethyl [4-(1,3-dioxolan-2-yl)-2,3,5,6-tetrafluorophenyl]phosphonate (3). In a 500 mL two-necked flask equipped with a magnetic stirrer, a solution of bromide **2** (14.15 g, 47 mmol, 1.0 equiv.) in anhydrous THF (130 mL) was cooled to –78 °C under Ar. Then *n*-BuLi (1.6 M in *n*-hexane, 35.2 mL, 56 mmol, 1.2 equiv.) was added dropwise to this solution, keeping the reaction mixture at –78 to –70 °C. Then the reaction mixture was stirred at –78 °C for 1 h. A solution of diethylphosphite chloride (6.8 mL, 47 mmol, 1 equiv.) in anhydrous THF (55 mL) was prepared and cooled to –78°C under Ar. This solution was cannulated into the reaction vessel, and the reaction mixture was stirred for an additional 2 h before being warmed to room temperature. The reaction mixture was quenched with a saturated aqueous solution of ammonium chloride (200 mL) and extracted with diethyl ether (3 × 100 mL). The combined organic layers were dried over anhydrous $MgSO_4$ and concentrated under reduced pressure. The crude product was purified by column chromatography using pentane/ethyl acetate (1:1 v/v). The target product was obtained in 25% yield (4.3 g) as a yellow oil. ^1H NMR (300 MHz, $CDCl_3$, 298 K): δ_H = 1.32 (dt, $^3J_{H,H}$ = 7.0 Hz, $^4J_{H,P}$ = 0.6 Hz, 6H, CH_3), 3.95–4.04 (m, 2H, OCH_2), 4.07–4.17 (m, 2H, OCH_2), 4.17–4.28 (m, 4H, $POCH_2$), 6.15 (s,1H, CH) ppm. $^{13}C\{^1H\}$ NMR (75 MHz, $CDCl_3$, 298 K): δ_C = 16.1 (d, $^3J_{C,P}$ = 6.7 Hz, 2C, CH_3), 62.5 (d, $^2J_{C,P}$ = 6.0 Hz, 2C, $POCH_2$), 66.1 (s, 2C, OCH_2), 96.4 (m, 1C, CH), 109.9 (dt, $^1J_{C,P}$ = 180.3 Hz, $^2J_{C,F}$ = 19.2 Hz, 1C, 1-C_{Ph}), 121.5 (td, $^2J_{C,F}$ = 12.8 Hz, $^4J_{C,P}$ = 2.4 Hz, 1C, 4-C_{Ph}), 145.1 (dm, $^1J_{C,F}$ = 253.3 Hz, 2C, 3-C_{Ph}), 146.7 (dm, $^2J_{C,F}$ = 253.8 Hz, 2C, 2-C_{Ph}) ppm. ^{19}F NMR (282 MHz, $CDCl_3$, 298 K): δ_F = −142.7 to −142.5 (m, 2F, 3-F), −132.0 to −131.8 (m, 2F, 2-F) ppm. $^{31}P\{^1H\}$ NMR (121 MHz, $CDCl_3$, 298 K): δ_P = 4.4 (br s) ppm. IR (neat): ṽ = 2985 (w), 2906 (w), 1469 (vs), 1395 (m), 1282 (s, P=O), 1262 (m, P=O), 1144 (w), 1116 (w), 1048 (s), 1015 (vs, P–O), 996 (s), 952 (s), 931 (m), 853 (w), 799 (w), 752 w), 642 (w), 577 (m, P–C) cm^{-1}. HRMS (ESI^+): *m/z* calcd for $C_{13}H_{16}F_4O_5P$: 359.0666 $[M+H]^+$; found: 359.0664; calcd for $C_{13}H_{15}F_4NaO_5P$: 381.0485 $[M+Na]^+$; found: 381.0482; calcd for $C_{26}H_{30}F_8NaO_{10}P_2$: 739.1079 $[2M+Na]^+$; found: 739.1075.

Table S1. Palladium-catalysed coupling of bromide **2** with diethyl *H*-phosphonate [a]

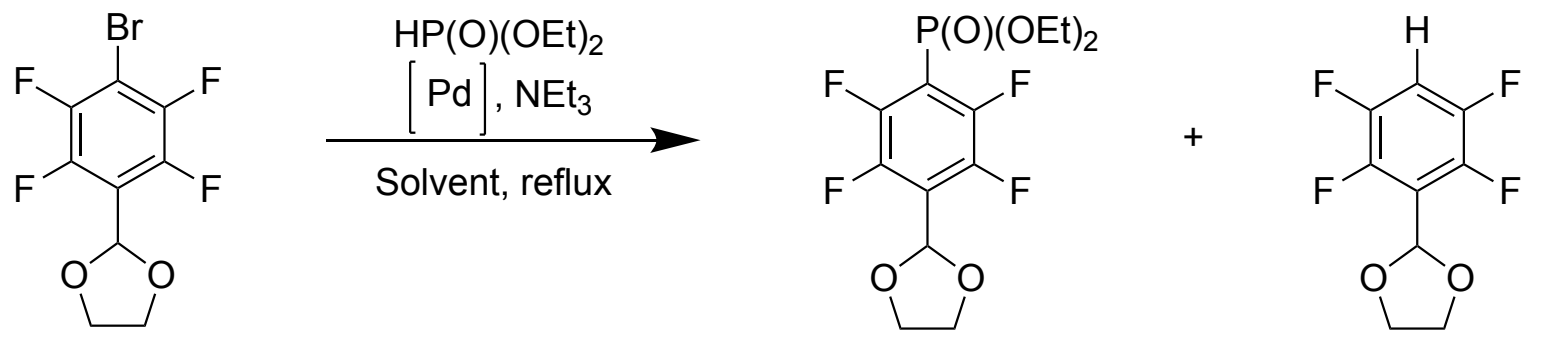

Entry	Pd source (mol%)	Ligand[b] (mol%)	Solvent[c]	Time (h)	Yield[d] **3**	**4**
1	$Pd(PPh_3)_4$ (5)	-	Tol	3		100
2	$Pd(PPh_3)_4$ (5)	-	Tol : EtOH (1 :1)	3		100
3	$Pd(OAc)_2$(10)	PPh_3(30)	Tol	3		100
4	$Pd(OAc)_2$ (10)	Dppf(10)	Tol	3		100
5	$Pd(OAc)_2$(10)	PPh_3(30)	Tol : EtOH (1 :1)	3		100
6	$Pd(OAc)_2$ (10)	Dppf (10)	Tol : EtOH (1 :1)	3		100
7	$Pd(OAc)_2$(15)	PPh_3(30)	DME	24		100
8	$Pd(OAc)_2$ (15)	Dppf (15)	DME	24		100
9	$Pd(OAc)_2$ (15)	Xantphos (15)	DME	24		100
10	$Pd(OAc)_2$ (15)	Xantphos (15)	MeCN	16		100
11	$Pd(OAc)_2$ (15)	Xantphos (15)	DMF	6		100
12	$Pd(OAc)_2$ (15)	Xantphos (15)	Tol	3		100
13	$Pd(OAc)_2$ (15)	Xantphos (15)	Tol/EG (9 :1)	2	26	55[e]
14	$Pd(OAc)_2$ (5)	Xantphos (5)	Tol/EG (9 :1)	4		100
15[f]	$Pd(OAc)_2$ (5)	Xantphos (5)	Tol/EG (9 :1)	4		100
16	$Pd(OAc)_2$ (30)	Xantphos (30)	Tol/EG (9 :1)	2	35	50[e]
17[g]	$Pd(OAc)_2$ (15)	Xantphos (15)	Tol/EG (9 :1)	2	25	65[e]
18	$Pd(OAc)_2$ (15)	DPEphos (15)	Tol/EtOH (1 :1)	2		100
19	$Pd(OAc)_2$ (15)	DPEphos (15)	Tol/EG (9 :1)	2		100

[a] Reaction conditions: bromide **1** (1 equiv), $HP(O)(OEt)_2$ (1.2 equiv), Et_3N (1.5 equiv), solvent (1–3 mL), reflux under N_2. [b] Dppf = 1,1’-bis(diphenylphosphino)ferrocene; Xantphos = 1,1'-(9,9-dimethyl-9*H*-xanthene-4,5-diyl)bis(1,1-diphenylphosphine); DPEphos = bis[(2-diphenylphosphino)phenyl] ether. [c] Tol = toluene; DME = 1,2-dimethoxyethane; EG = ethyleneglycol. [d] The yield was determined by NMR spectroscopic analysis. [e] Another side product was observed in the reaction mixture. [f] 5 equiv. of $HP(O)(OEt)_2$ and 3 equiv. of NEt_3 were used. [g] Tiethylamine was replaced by *N,N*-diisoprophylethylamine.

Diethyl (4-formyl-2,3,5,6-tetrafluorophenyl)phosphonate (6). In a 500 mL two-necked flask equipped with a reflux condenser, dioxolane **3** (7.75 g, 21.6 mmol) was added to a mixture of TFA (75 mL), concentrated HCl (30 mL), and H_2O (75 mL) under an argon atmosphere. The reaction mixture was then stirred for 3 h at 60 °C. After cooling the reaction mixture to room temperature, CH_2Cl_2 (150 mL) was added to the flask. Then the organic layer was separated and washed with a saturated sodium

bicarbonate solution (100 mL) and water (2 × 100 mL). After drying over anhydrous $MgSO_4$, this solution was evaporated under reduced pressure to afford the target phosphonate **6** in 91% yield (6.15 g) as a white solid. M. p. 102–104 °C. ^{1}H NMR (300 MHz, $CDCl_3$, 298 K): δ_H = 1.38 (t, $^3J_{H,H}$ = 7.3 Hz, 6H, CH_3), 4.36–4.20 (m, 4H, OCH_2), 10.30 (s, 1H, CHO) ppm. ^{13}C{^{1}H} NMR (75 MHz, $CDCl_3$, 298 K): δ_C = 16.1 (d, $^3J_{C,P}$ = 6.7 Hz, 2C, CH_3), 63.9 (d, $^3J_{C,P}$ = 6.1 Hz, 2C, OCH_2), 114.87 (dt, $^1J_{C,P}$ = 180.8 Hz, $^2J_{C,F}$ = 18.0 Hz, 1C, 1-C_{Ph}), 117.8 (td, $^2J_{C,F}$ = 9.8 Hz, $^4J_{C,P}$ = 2.3 Hz, 1C, 4-C_{Ph}), 146.2 (dm, $^2J_{C,F}$ = 263.3 Hz, 2C, 2-C_{Ph}), 146.7 (dm, $^2J_{C,F}$ = 255.0 Hz, 2C, 3-C_{Ph}), 181.9 (m, 1C, CH(O)) ppm. ^{19}F NMR (282 MHz, $CDCl_3$, 298 K): δ_F = −135.3 to −138.7 (m, 2F, 3-F), −131.6 to −131.7 (m, 2F, 2-F) ppm. ^{31}P{^{1}H} NMR (121 MHz, $CDCl_3$, 298 K): δ_P = 3.19 (br s) ppm. IR (neat): ṽ = 2988 (w), 2911 (w), 1790 (w), 1716 (m, C=O), 1466 (vs), 1396 (w), 1287 (s, P=O), 1258 (m, P=O), 1226 (w), 1164 (w), 1013 (vs, P–O), 961 (s), 848 (m), 801 (w), 569 (m, P–C) cm^{-1}. HRMS (ESI$^+$): *m/z* calcd for $C_{11}H_{12}F_4O_4P$: 315.0404 $[M+H]^+$; found: 315.0408; calcd for $C_{11}H_{11}F_4NaO_4P$: 337.0223 $[M+Na]^+$; found: 337.0227; calcd for $C_{12}H_{16}F_4O_5P$: 347.0666 $[M+CH_3OH+H]^+$; found: 347.0670; calcd for $C_{12}H_{15}F_4NaO_5P$: 369.0485 $[M+CH_3OH+Na]^+$; found: 369.0489.

5,10,15,20-Tetrakis[(4-(diethoxyphosphoryl)-2,3,5,6-tetrafluorophenyl]porphyrin (H_2TF_4PPP). A 250 mL two-necked flask equipped with a magnetic stirrer and a back-flow condenser was charged with compound **6** (679 mg, 2.16 mmol, 1.0 equiv.) in dry CH_2Cl_2 (150 mL). The solution was stirred for 30 min while bubbling N_2. Then freshly distilled pyrrole (145 mg, 151 µL, 2.16 mmol, 1.0 equiv.) and $BF_3{\cdot}OEt_2$ (796 µL, 6.44 mmol, 2.98 equiv.) were added to the solution, and the reaction mixture was stirred at room temperature for 1 h. Then pyridine (2 mL) and DDQ (881 mg, 3.88 mmol, 1.8 equiv.) was added and the reaction mixture was stirred for 1 h. The solvent was removed under reduced pressure, and the residue was purified by column chromatography using CH_2Cl_2/MeOH (97:3 v/v) as an eluent. The porphyrin H_2TF_4PPP was isolated as a brown solid in 16% yield (123 mg). ^{1}H NMR (300 MHz, $CDCl_3$, 298 K): δ_H = 1.62 (t, ^3J = 8.9 Hz, 24H, CH_3), 4.57–4.49 (m, 16H, OCH_2), 8.97 (s, 8 H, β-H) ppm. ^{13}C{^{1}H} NMR (75 MHz, $CDCl_3$, 298 K): δ_C = 16.5 (d, $^3J_{C,P}$ = 6.7 Hz, 8C, CH_3), 64.1 (d, $^3J_{C,P}$ = 6.0 Hz, 8C; OCH_2), 104.2 (s, 4C, *meso*-C), 111.2 (dt, $^1J_{C,P}$ = 182.2 Hz, $^2J_{C,F}$ = 19.5 Hz, 4C, 4-C_{Ph}), 124.7 (t, $^2J_{C,F}$ = 17.2 Hz, 4C, 1-C_{Ph}), 131.3 (br s, 8C, β-C), 144.45–144.98 (m, 8C) and 147.95–148.65 (m, 8C) (3-C_{Ph} and 2-C_{Ph},) ppm; the signal of α-C is not observed. ^{19}F NMR (282 MHz, $CDCl_3$, 298 K): δ_F = −135.4 to −135.3 (m, 8F, 2-F), −131.9 to −131.4 (m, 8F, 3-F) ppm. ^{31}P{^{1}H} NMR (121 MHz, $CDCl_3$, 298 K): δ_P = 5.1 (br s) ppm. UV–vis (CH_2Cl_2): λ_{max} [ε (M^{-1} cm^{-1})] = 414 (285), 507 (21), 540 (3), 584 (6), 637 (1) nm. IR (neat): ṽ = 3321 (w, N–H), 3097 (w, N–H), 2984 (w), 2907 (w), 1646 (w), 1563 (w), 1455 (vs), 1393 (m), 1343 (w), 1286 (m, P=O), 1258 (s, P=O), 1161 (m), 1118 (m), 1045 (m), 1014 (vs, P–O), 969 (vs), 960 (vs), 934 (vs), 803 (m), 748 (m), 727 (m), 637 (w), 575

(s, P–C) cm^{-1}. HRMS (ESI$^+$): *m/z* calcd for ½($C_{60}H_{50}F_{16}N_4Na_2O_{12}P_4$): 746.0952 [M+2Na]$^{2+}$; found: 746.0969; calcd for $C_{60}H_{51}F_{16}N_4O_{12}P_4$: 1447.2193 [M+H]$^+$; found: 1447.2204.

3. Synthesis of metalloporphyrin MTF$_4$PPP and InTF$_4$PPP-M

{5,10,15,20-Tetrakis[(4-(diethoxyphosphoryl)-2,3,5,6-tetrafluorophenyl]porphyrinato}zinc(II) (ZnTF$_4$PPP). A solution of $Zn(OAc)_2\cdot 2H_2O$ (62 mg, 0.28 mmol, 40 equiv.) in methanol (8 mL) was added to a stirred solution of H_2TF$_4$PPP (10 mg, 0.007 mmol) in chloroform (16 mL). The reaction mixture was stirred overnight at room temperature. The crude product was purified by column chromatography using a mixture of $CHCl_3$/MeOH (96:4 v/v) to give ZnTF$_4$PPP in 90% yield (9.5 mg) as a violet solid. ^{1}H NMR (300 MHz, $CDCl_3$, 298 K): δ_H = 1.31 (t, $^3J_{H,H}$ = 7.1 Hz, 24H, CH_3), 4.36–4.16 (m, 16 H, OCH_2), 8.72 (s, 8 H, β–H) ppm. ^{13}C{^{1}H} NMR (75 MHz, $CDCl_3$, 298 K): δ_C = 16.0 (d, $^3J_{C,P}$ = 6.7 Hz, 8C, CH_3), 64.3 (d, $^3J_{C,P}$ = 6.0 Hz, 8C, OCH_2), 104.0 (s, 4C, *meso*-C), 109.5 (dt, $^1J_{C,P}$ = 185.2 Hz, $^2J_{C,F}$ = 18.7 Hz, 4C, 4-C_{Ph}), 126.9 (m, 4C, 1-C_{Ph}), 131.7 (s, 8C, β-C), 144.6–145.1 (m, 8C) and 148.2–148.5 (m, 8C) (3-C_{Ph} and 2-C_{Ph}, respectively), 149.6 (s, 8C, α-C) ppm. ^{19}F NMR (282 MHz, $CDCl_3$, 298 K): δ_F = −136.3 to −136.0 (m, 8F, 2-F), −133.1 to −132.9 (m, 8F, 3-F) ppm. ^{31}P{^{1}H} NMR (121 MHz, $CDCl_3$, 298 K): δ_p = 5.50 (br s) ppm. UV–vis (CH_2Cl_2): λ_{max} [ε (M^{-1} cm^{-1})] = 416 (314), 548 (17), 580 (3) nm. IR (neat): ṽ = 2988 (w), 2924 (w), 2854 (w), 1644 (w), 1454 (vs), 1393 (w), 1335 (w), 1279 (m, P=O), 1253 (m, P=O), 1162 (w), 1132 (m), 1044 (m), 1012 (vs, P–O), 961 (vs), 836 (w), 798 (m), 749 (m), 575 (m, P–C) cm^{-1}. HRMS (ESI$^+$): *m/z* calcd for ½($C_{60}H_{48}F_{16}N_4Na_2O_{12}P_4Zn$): 777.0520 [M+2Na]$^{2+}$; found: 777.0520; calcd for $C_{60}H_{48}F_{16}N_4NaO_{12}P_4Zn$: 1533.1145 [M+Na]$^+$; found: 1533.1141.

{5,10,15,20-Tetrakis[(4-(diethoxyphosphoryl)-2,3,5,6-tetrafluorophenyl]porphyrinato}-palladium(II) (PdTF$_4$PPP). A solution of palladium acetate (58.60 mg, 0.26 mmol, 7.4 equiv.) in acetonitrile (40 mL) was added to a solution of porphyrin H_2TF$_4$PPP (51 mg, 0.034 mmol) in chloroform (10 mL). The reaction mixture was stirred at reflux for 1 h, and then the volatiles were removed under reduced pressure. The residue was purified by column chromatography using a mixture of CH_2Cl_2/MeOH (97:3 v/v) as an eluent. The complex PdTF$_4$PPP was obtained as a red solid in 92% yield (48 mg). ^{1}H NMR (300 MHz, $CDCl_3$, 298 K): δ_H = 1.61 (t, $^3J_{H,H}$ = 7.1 Hz, 24H, CH_3), 4.47–4.66 (m, 16H, OCH_2), 8.93 (s, 8H, β–H) ppm. ^{13}C{^{1}H} NMR (75 MHz, $CDCl_3$, 298 K): δ_C = 16.5 (d, $^3J_{C,P}$ = 6.7 Hz, 8C, CH_3), 64.1 (d, $^3J_{C,P}$ = 6.0 Hz, 8C; OCH_2), 106.0 (s, 4C, *meso*-C), 111.2 (dt, $^1J_{C,P}$ = 183.1 Hz, $^2J_{C,F}$ = 18.7 Hz, 4C, 4-C_{Ph}), 124.4 (t, $^2J_{C,F}$ = 17.2 Hz, 4C, 1-C_{Ph}), 131.4 (s, 8C, β-C), 141.3 (s, 8C, α-C), 144.1–144.5 and 148.0 –148.3 (dm, 16C, 3-C_{Ph} and 2-C_{Ph},) ppm. ^{19}F NMR (282 MHz, $CDCl_3$,

298 K): δ_F = −135.6 to −135.0 (m, 8F, 2-F_{Ph}), −131.9 to −131.3 (m, 8F, 3-F_{Ph}) ppm. $^{31}P\{^1H\}$ NMR (21 MHz, $CDCl_3$, 298 K): δ_p = 5.1 (br s) ppm. UV–vis (CH_2Cl_2): λ_{max} [ε (M^{-1} cm^{-1})]] = 409 (162), 519 (14), 553 (10) nm. IR (neat): ṽ= 2924 (w), 2853 (w), 1646 (w), 1568 (w),1460 (s), 1395 (w), 1348 (w), 1287 (s, P=O), 1258 (s, P=O), 1145 (m), 1046 (m), 1016 (vs, P–O), 970 (vs), 838 (s), 751 (s), 703 (w), 574 (m, P–C) cm^{-1}. HRMS (ESI^+): *m/z* calcd for ½($C_{60}H_{48}F_{16}N_4Na_2O_{12}P_4Pd$): 798.0402 $[M+2Na]^{2+}$; found: 798.0409.

{5,10,15,20-Tetrakis[(4-(diethoxyphosphoryl)-2,3,5,6-tetrafluorophenyl]porphyrinato}-indium(III) chloride ($InTF_4PPP$). A solution of H_2TF_4PPP (10 mg, 0.007 mmol), indium(III) chloride (6 mg, 0.027 mmol, 3 equiv.), and sodium acetate (21 mg, 0.26 mmol) in glacial acetic acid (10 mL) was refluxed for 12 h. Complete conversion of starting porphyrin was achieved according to the UV–vis analysis of the reaction mixture. The volatiles were removed under reduced pressure, and the solid residue was purified by column chromatography using a mixture of CH_2Cl_2/MeOH (gradient 9:1 → 2:1 v/v) as an eluent. The colored fraction obtained was a mixture of $InTF_4PPP$ and H_2TF_4PPP in the 45:65 ratio. The silica gel was treated with a CH_2Cl_2/MeOH/NEt_3 (9:1:0.05 v/v/v) solvent mixture, and the chromatographic separation was repeated using this solvent mixture as an eluent. Two colored fractions were obtained, $InTF_4PPP$ being in the last one. The fraction was diluted with CH_2Cl_2 (20 mL) and treated with a concentrated solution of NaCl (2 × 5 mL), and washed with water (1 × 5 mL) in a decantation ampoule. The solution thus obtained was evaporated and dried under reduced pressure. The complex $InTF_4PPP$ was obtained as a rose-violet solid. Yield: 22% (2.4 mg). ^1H NMR (300 MHz, $CDCl_3$, 298 K): δ_H = 1.63 (t, $^3J_{H,H}$ = 7.1 Hz, 24H, CH_3), 4.5–4.6 (m, 16H, OCH_2), 8.9 (s, 8H, *β*–H) ppm. ^{19}F NMR (282 MHz, $CDCl_3$, 298 K): δ_F = −135.9 to −135.7 and −134.3 to −134.1 (dm, 8F, 2-F_{ph}), −131.6 to −131.4 and −131.1 to −129.9 (dm, 8F, 3-F_{ph}) ppm. $^{31}P\{^1H\}$ NMR (121 MHz, $CDCl_3$, 298 K): δ_p = −4.9 (br s) ppm. UV–vis (CH_2Cl_2): λ_{max} [ε (M^{-1} cm^{-1})] = 399 (38), 421 (365), 512 (2), 553 (18), 588 (2) nm. IR (neat): ṽ= 3027 (w), 3060 (w), 3028 (w), 2922 (w), 2849 (w), 1687 (s), 1599 (m), 1583 (m), 1493 (m), 1450 (w), 1317 (w), 1264 (m, P=O), 1215 (m), 1178 (m), 1022 (m, P–O), 1002 (w), 974 (w), 838 (s), 753 (m), 696 (m), 574 (m, P–C) cm^{-1}. HRMS (ESI^+): *m/z* calcd for ½($C_{60}H_{48}F_{16}InN_4Na_2O_{12}P_4$): 791.0445 $[M\text{-}Cl+Na]^{2+}$; found: 791.0461; ½($C_{60}H_{50}F_{16}InN_4NaO_{13}P_4$): 800.0497 $[M\text{-}Cl+H_2O+Na]^{2+}$; found: 800.0509; ½($C_{62}H_{51}F_{16}InN_4O_{14}P_4$): 809.0562 $[M\text{-}Cl+MeCO_2]^{2+}$; found: 809.0555;calcd for $C_{60}H_{48}F_{16}InN_4O_{12}P_4$: 1559.1000 $[M\text{-}Cl]^+$; found: 1559.1027; calcd for $C_{60}H_{49}F_{16}InN_4O_{12}P_4$: 1560.1080 $[M\text{-}Cl+H]^+$; found: 1560.1056.

A rapid (on the NMR time scale) exchange of the axial chloride ion is observed in methanol-d_4, as previously reported for In(III) complexes with other porphyrins substituted with electron-withdrawing groups [99].

^{19}F NMR (282 MHz, CD_3OD, 298 K): δ_F = −138.7 to −135.2 (dm, 8F, 2-F_{ph}), −133.8 to −133.7 (dm, 8F, 3-F_{ph}) ppm.

{5,10,15,20-Tetrakis{[4-[ethoxy)(hydroxy)phosphoryl]-2,3,5,6-tetrafluorophenyl}porphyrinato}indium(III) chloride (InTF$_4$PPP-M). A solution of H_2TF$_4$PPP (10 mg, 0.007 mmol), indium(III) chloride (9 mg, 0.039 mmol, 5.6 equiv.), and sodium acetate (42 mg, 0.52 mmol) in glacial acetic acid (10 mL) was refluxed for 48 h. The volatiles were removed under reduced pressure, and the solid residue was purified by column chromatography using a mixture of CH_2Cl_2/MeOH/NEt_3 (gradient 10:1:0.05→ 2:1:0.05 v/v/v) as an eluent. The coloured solution thus obtained was evaporated and dried under reduced pressure. The complex InTF$_4$PPP-M was obtained as a rose-violet solid. Yield: 21% (2.2 mg). ^{1}H NMR (300 MHz, CD_3OD, 298 K): δ_H = 1.4 (s, 12H, CH_3), 4.2 (s, 8H, OCH_2), 9.5 (s, 8H, β–H) ppm. ^{13}C{^{1}H} NMR (75 MHz, CD_3OD, 298 K): δ_C = 15.6 (s, 4C, CH_3), 61.1 (s, 4C; OCH_2), 106.3 (s, 4C, *meso*-C), 120.9 (s, 4C, 1-C_{Ph}), 133.4 (s, 8C, β-C), 146.9 (br d, $J_{C,P}$ = 257.5 Hz, 16C, 2-C_{Ph} and 3-C_{Ph}), 149.0 (s, 8C, α-C) ppm; the signal of 4-C_{Ph} is not observed. ^{19}F NMR (282 MHz, CD_3OD, 298 K): δ_F = −140.8 (s, 8F, 2-F_{ph}), −134.4 (s, 8F, 3-F_{Ph}) ppm. ^{31}P{^{1}H} NMR (121 MHz, CD_3OD, 298 K): δ_p = −1.1 (br s) ppm. UV–vis (CH_3OH): λ_{max} [ε (M^{-1} cm^{-1})] = 393 (57), 414 (532), 509 (5), 548 (26), 583 (3) nm. IR (neat): ṽ = 3366 (br w, O–H), 3028 (m), 2922 (w), 1681 (w), 1448(w), 1277 (s, P=O), 1232 (br m, P=O), 1134 (m), 1080 (w), 1045 (w), 964 (vs), 797 (w), 754 (m), 699 (m), 571 (w, P–C) cm^{-1}. HRMS (ESI$^-$): *m/z* calcd for ⅓($C_{52}H_{29}F_{16}InN_4O_{12}P_4$): 480.9842 $[M–Cl–3H]^{3-}$; found: 480.9842; calcd for ⅓($C_{52}H_{29}F_{16}InN_4O_{12}P_4$): 492.9763 $[M–3H]^{3-}$; found: 492.9740; calcd for ⅓ ($C_{53}ClH_{31}F_{16}InN_4O_{14}P_4$): 496.3193 $[M–Cl+HCO_2H–3H]^{2-}$; found 496.3170.

4. Synthesis of porphyrins MTF$_4$PPP -A

5,10,15,20-Tetrakis[(4-(dihydroxyphosphoryl)-2,3,5,6-tetrafluorophenyl]porphyrin (H_2TF$_4$PPP-A). A Schlenk tube was charged with the porphyrin H_2TF$_4$PPP (50 mg, 0.035 mmol), and the evacuation/inert gas (Ar) refill cycle was repeated three times. Anhydrous CH_2Cl_2 (4 mL) and bromotrimethylsilane (131.6 mg, 113 µL, 0.86 mmol, 24.6 equiv.) were added by syringe, and the reaction mixture was stirred for 48 h at room temperature. The volatiles were distilled under reduced pressure, MeOH (2 mL) was added, and the solution was stirred for 1 h at room temperature. Evaporation of the volatiles under reduced pressure afforded H_2TF$_4$PPP-A as a crystalline hydroscopic powder in quantitative yield. A low solubility of the acid in organic solvents has prevented its purification by column chromatography. ^{1}H NMR (300 MHz, D_2O + NaOD (40% in D_2O, 1 drop), 298 K): δ_H = 9.26 (s, β–H) ppm. ^{19}F NMR (300 MHz, D_2O + NaOD (40% in D_2O, 1 drop), 298 K): δ_F = −140.9 to −140.5 (s, 8F, 2-F), −135.7 to −135.2 (s, 8F, 3-F) ppm. ^{31}P{^{1}H} NMR (121 MHz, D_2O +

NaOD (40% in D_2O, 1 drop), 298 K): $\delta_P = -0.2$ (br s) ppm. UV–vis (MeCN/ H_2O (1:1 v/v) + 0.1 M NaOH (1 drop), nm): λ_{max} [ε (M^{-1} cm^{-1})] = 409 (291), 505 (17), 537 (2), 579 (6), 632 (1) nm. IR (neat): ṽ = 3371 (br w, O–H), 3152 (w), 3086 (w), 3023 (m), 2995 (w), 1641 (w), 1499 (w), 1452 (vs), 1306 (w),1272 (s, P=O), 1200 (br m, P=O), 1122 (m), 1014 (w), 998 (w), 956 (vs), 843 (w), 806 (m), 749 (m), 570 (w, P–C) cm^{-1}. HRMS (ESI^-): *m/z* calcd for ⅓($C_{44}H_{13}F_{16}N_4O_{12}P_4$): 406.3133 $[M–3H]^{3–}$; found: 406.3135; calcd for ⅓($C_{44}H_{12}F_{16}N_4NaO_{12}P_4$): 413.6406 $[M–4H+Na]^{3–}$; found: 413.6404; calcd for ½($C_{44}H_{14}F_{16}N_4O_{12}P_4$): 609.9731 $[M–2H]^{2–}$; found 609.9735; calcd for ½($C_{44}H_{13}F_{16}N_4NaO_{12}P_4$): 620.9644 $[M–3H+Na]^{2–}$; found: 620.9645; calcd for ½($C_{44}H_{12}F_{16}N_4Na_2O_{12}P_4$) 631.9555 $[M–4H+2Na]^{2–}$; found: 631.9556.

{5,10,15,20-tetrakis[(4-(dihydroxyphosphoryl)-2,3,5,6-tetrafluorophenyl]porphyrinato}-palladium (PdTF$_4$PPP-A). A Schlenk tube was charged with complex PdTF$_4$PPP (23 mg, 0.015 mmol), and the evacuation/inert gas refill cycle was repeated three times. Anhydrous CH_2Cl_2 (3 mL) and bromotrimethylsilane (58.1 mg, 50 µL, 0.38 mmol, 25 equiv.) were added by syringe, and the reaction mixture was stirred for 48 h at room temperature. The volatiles were distilled under reduced pressure, MeOH (2 mL) was added, and the solution was stirred for 1 h at room temperature. Evaporation of the volatiles under reduced pressure afforded PdTF$_4$PPP-A as a crystalline hydroscopic powder in quantitative yield. A low solubility of the acid in organic solvents has prevented its purification by column chromatography.

^1H NMR (300 MHz, D_2O + NaOD (40% in D_2O, 1 drop), 298 K): δ_H 9.34 (s, 8H, β–H) ppm. ^{19}F NMR (282 MHz, D_2O + NaOD (40% in D_2O, 1 drop), 298 K): $\delta_F = -140.8$ to -140.7 (m, 8F, 2-F_{Ph}), -134.8 to -134.7 (m, 8F, 3-F_{Ph}) ppm. $^{31}P\{^1H\}$ NMR (121 MHz, CD_3OD), 298 K): $\delta_P = -1.0$ (br s) ppm. UV–vis (H_2O + 0.1 M NaOH (1drop), nm): λ_{max} = 403, 517, 551 nm. IR (neat): ṽ = 3288 (br m, O–H), 2963 (w), 1643 (m), 1455 (m, 1352 (w), 1260 (w, P=O), 1130 (m), 1049 (s, P–O), 1013 (m), 963 (vs), 836 (w), 795 (m), 732 (w), 579 (m, P–C) cm^{-1}. HRMS (ESI^-): *m/z* calcd for ⅓($C_{44}H_{13}F_{16}N_4O_{12}P_4Pd$) 440.9431 $[M–3H]^{3–}$; found: 440.9442; calcd for ½($C_{44}H_{14}F_{16}N_4O_{12}P_4Pd$) 661.9182 $[M–2H]^{2–}$, found: 661.9196.

5. DFT calculations

To simplify the calculations, ethyl substituents of phosphonate groups were replaced by methyl groups. The structure of MTF_4PPP-M was modeled by DFT calculations using the Firefly quantum chemistry package [142], which is partially based on the GAMESS (US) [143] source code. The calculations were performed using B3LYP/6-311G(d,p) ($M = 2H^+$) or B3LYP/6-31(d,p) ($M = Zn^{2+}$), at each step, full optimization of geometry was achieved, and the minima were confirmed by computation of vibration frequencies.

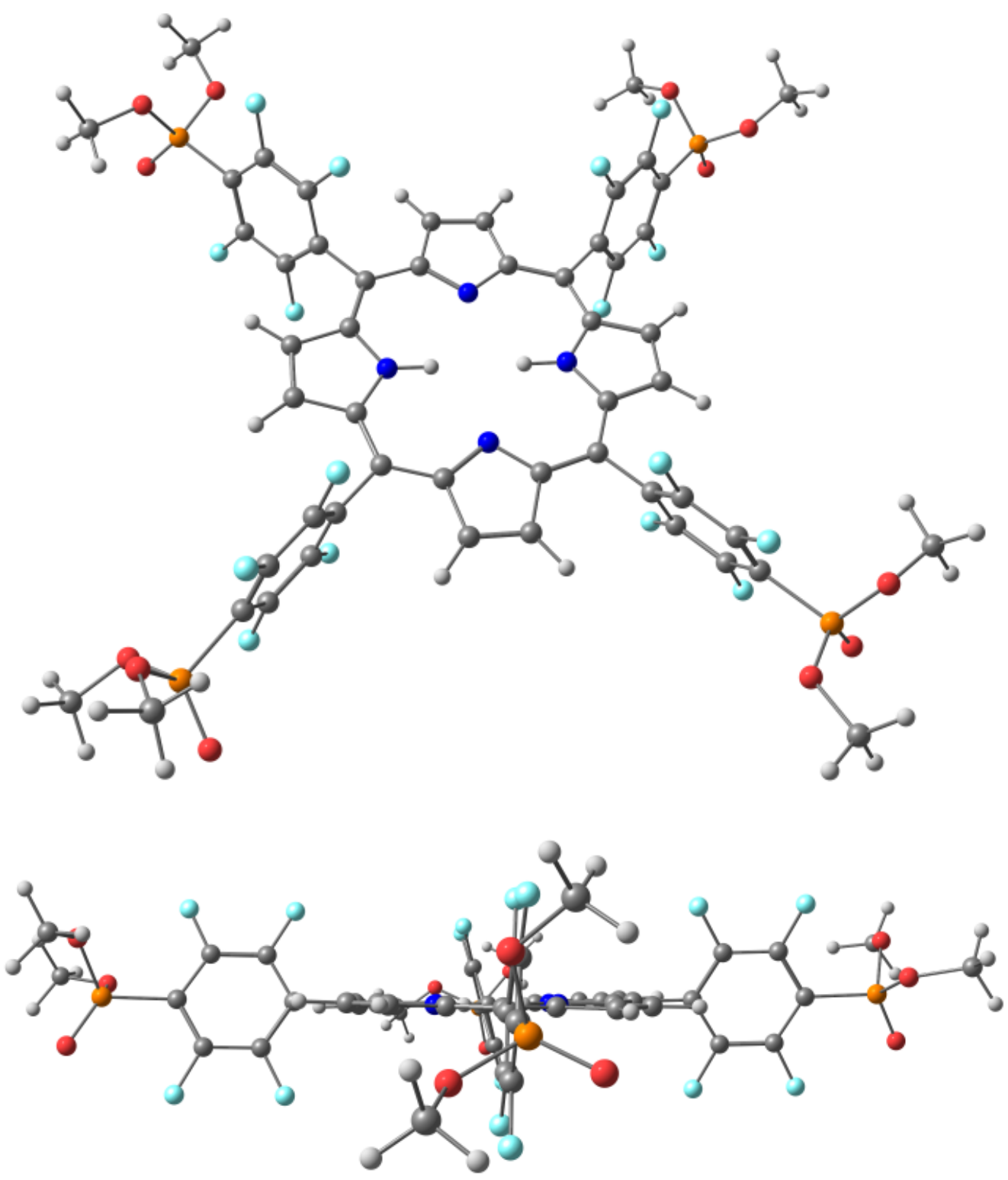

Figure S1. The structure of H_2TF_4PPP-M complex obtained by full geometry optimization at B3LYP/6-311G(d,p) level.

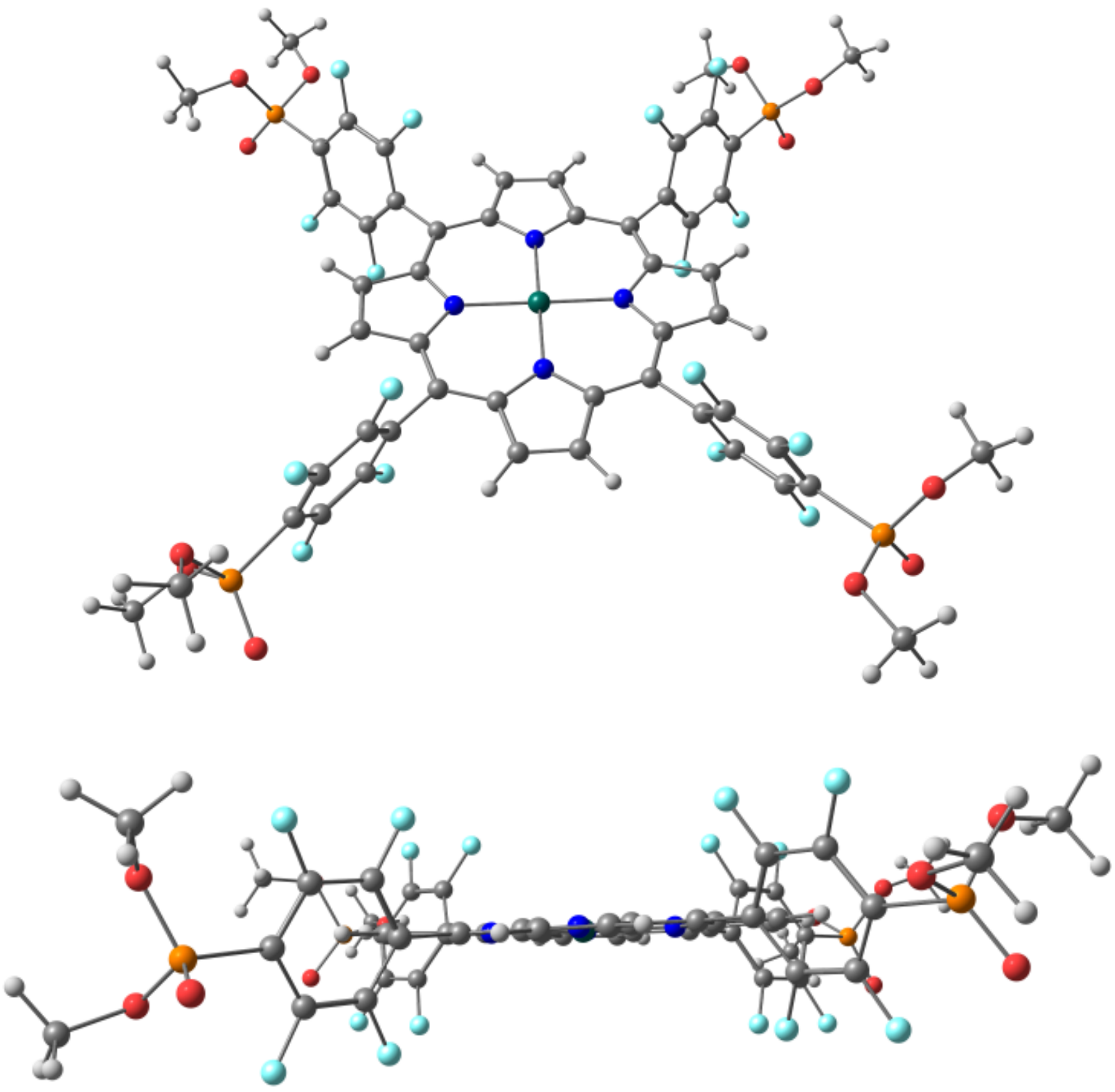

Figure S2. The structure of $ZnTF_4PPP$-M complex obtained by full geometry optimization at B3LYP/6-31(d,p) level.

Table S2. Selected bonds lengths (Å) and angles (°) in MTF_4PPP-M (M = $2H^+$, Zn^{2+}) obtained by full geometry optimization at B3LYP/6-311G(d,p) (M = $2H^+$) or B3LYP/6-31(d,p) (M = Zn^{2+}) level.

Compound	M–N	P=O	P–O	P–C	C–C(Ph)	N4–M[a]	N4/Ph dihedral angle
H_2TF_4PPP-M	-	1.48	1.599–1.614	1.827	1.387-1.398	-	73.70
							75.84
							75.21
							86..91
$ZnTF_4PPP$-M	2.04	1.48	1.606–1.620	1.823	1.391–1.400	0	69.96
							71.86
							72.02
							77.50

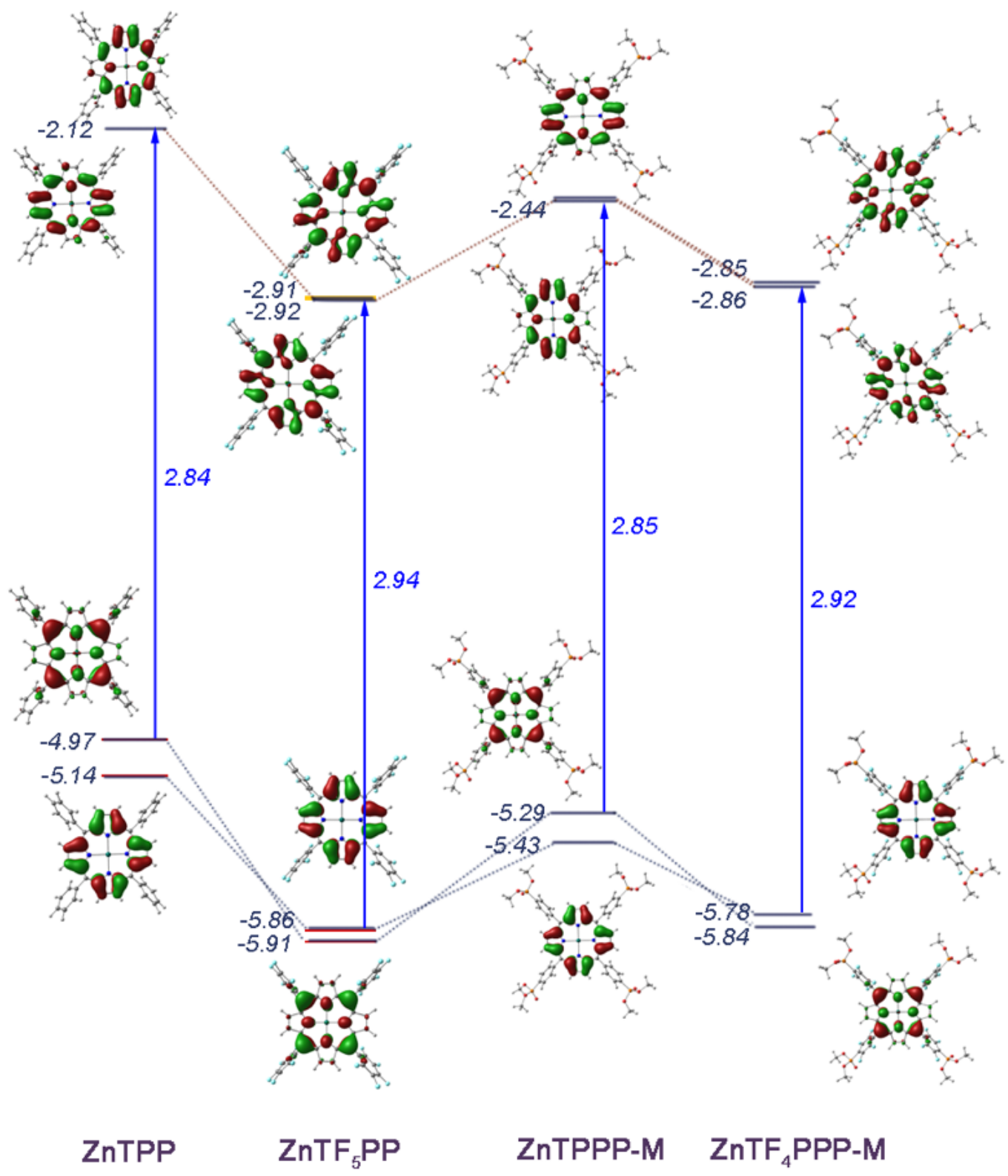

Figure S3. The isodensity plot of HOMO and LUMO orbitals for model chromophores $ZnTF_4PPP$-M and related compounds obtained from DFT calculations.

6. X-ray crystallography of H_2TF_4PPP

Single clear dark violet prism-shaped crystals of H_2TF_4PPP were obtained by slow diffusion of *n*-hexane in a solution of this compound in toluene/chloroform solvent mixture (1:3 v/v). A suitable crystal with dimensions 0.188 × 0.253 × 0.298 mm^3 was selected and mounted on a MiTeGen micromount with perfluoroether oil. Data were collected from a shock-cooled single crystal at 110.0(1) K on a Nonius Kappa Apex II *κ*-geometry diffractometer with a X-ray tube using a graphite as monochromator and an APEX2 detector. The diffractometer was equipped with an Oxford Cryostream 600 low temperature device and used Mo *Kα1* radiation (*λ* = 0.71073 Å). All data were integrated with SAINT V8.40B and a multi-scan absorption correction using SADABS 2016/2 was applied [145, 146]. The structure was solved by dual methods with SHELXT and refined by full-matrix least-squares methods against F^2 using SHELXL 2019/3 [146, 147]. All non-hydrogen atoms were refined with anisotropic displacement parameters, except for minor disordered parts. The hydrogen atoms were refined isotropically on calculated positions using a riding model with their *U*iso values constrained to 1.5 times the *U*eq of their pivot atoms for terminal sp^3 carbon atoms and 1.2 times for all other carbon atoms. Crystallographic data for the structures reported in this paper have been deposited with the Cambridge Crystallographic Data Centre [148].

*Crystal Data***.** $C_{63.50}H_{54}F_{16}N_4O_{12}P_4$, M_r = 1492.98, triclinic, *P*-1 (No. 2), *a* = 9.971(3) Å, *b* = 11.913(4) Å, c = 15.326(5) Å, α = 91.476(13)°, β = 96.358(12) °, γ = 103.052(13)°, *V* = 1760.2(11) Å^3, *T* = 110.0(1) K, *Z* = 1, μ(Mo $K_{\alpha 1}$) = 0.210, 40161 reflections measured, 6204 unique (R_{int} = 0.0592) which were used in all calculations. The final wR_2 was 0.2544 (all data) and R_{sigma} was 0.0403. Additional structural data are given in Figures S4 and S5 and Tables S3–S5.

CCDC 2520463 contain the supplementary crystallographic data for this paper. These data can be obtained free of charge from The Cambridge Crystallographic Data Centre via www.ccdc.cam.ac.uk/structures.

Table S3. Crystal data and structure refinement for H_2TF_4PPP.

CCDC number	
Empirical formula	$C_{63.50}H_{54}F_{16}N_4O_{12}P_4$
Formula weight	1492.98
Temperature [K]	110.0(1)
Crystal system	triclinic
Space group (number)	$P\bar{1}$ (2)
a [Å]	9.971(3)
b [Å]	11.913(4)
c [Å]	15.326(5)
α [°]	91.476(13)
β [°]	96.358(12)
γ [°]	103.052(13)
Volume [Å^3]	1760.2(11)
Z	1
ρ_{calc} [gcm^{-3}]	1.408
μ [mm^{-1}]	0.210
F(000)	763
Crystal size [mm^3]	0.188×0.253×0.298
Crystal colour	clear dark violet
Crystal shape	prism
Radiation	Mo K_{α} (λ=0.71073 Å)
2θ range [°]	3.51 to 50.00 (0.84 Å)
Index ranges	$-11 \le h \le 11$<br>$-14 \le k \le 14$<br>$-18 \le l \le 18$
Reflections collected	40161
Independent reflections	6204<br>R_{int} = 0.0592<br>R_{sigma} = 0.0403
Completeness to θ = 25.000°	99.9
Data / Restraints / Parameters	6204 / 385 / 445
Goodness-of-fit on F^2	1.060
Final *R* indexes [$I \ge 2\sigma(I)$]	R_1 = 0.0834<br>wR_2 = 0.2321
Final *R* indexes [all data]	R_1 = 0.1047<br>wR_2 = 0.2544
Largest peak/hole [eÅ^{-3}]	1.12/−1.37

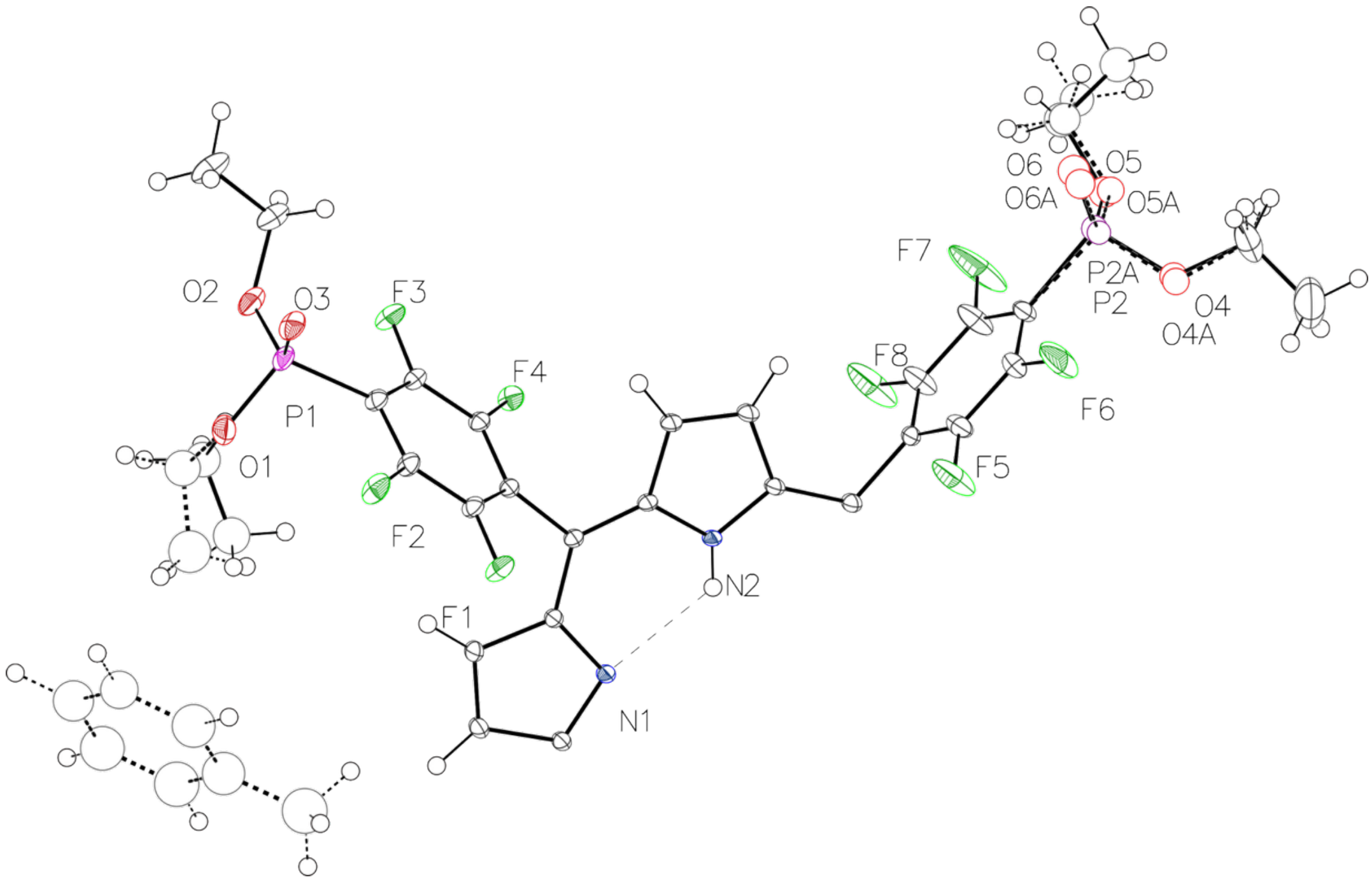

Figure S4. ORTEP view of the asymmetric unit of H_2TF_4PPP. Thermal ellipsoids are drawn at a 25% probability plot. Minor disordered parts and solvent are omitted for clarity.

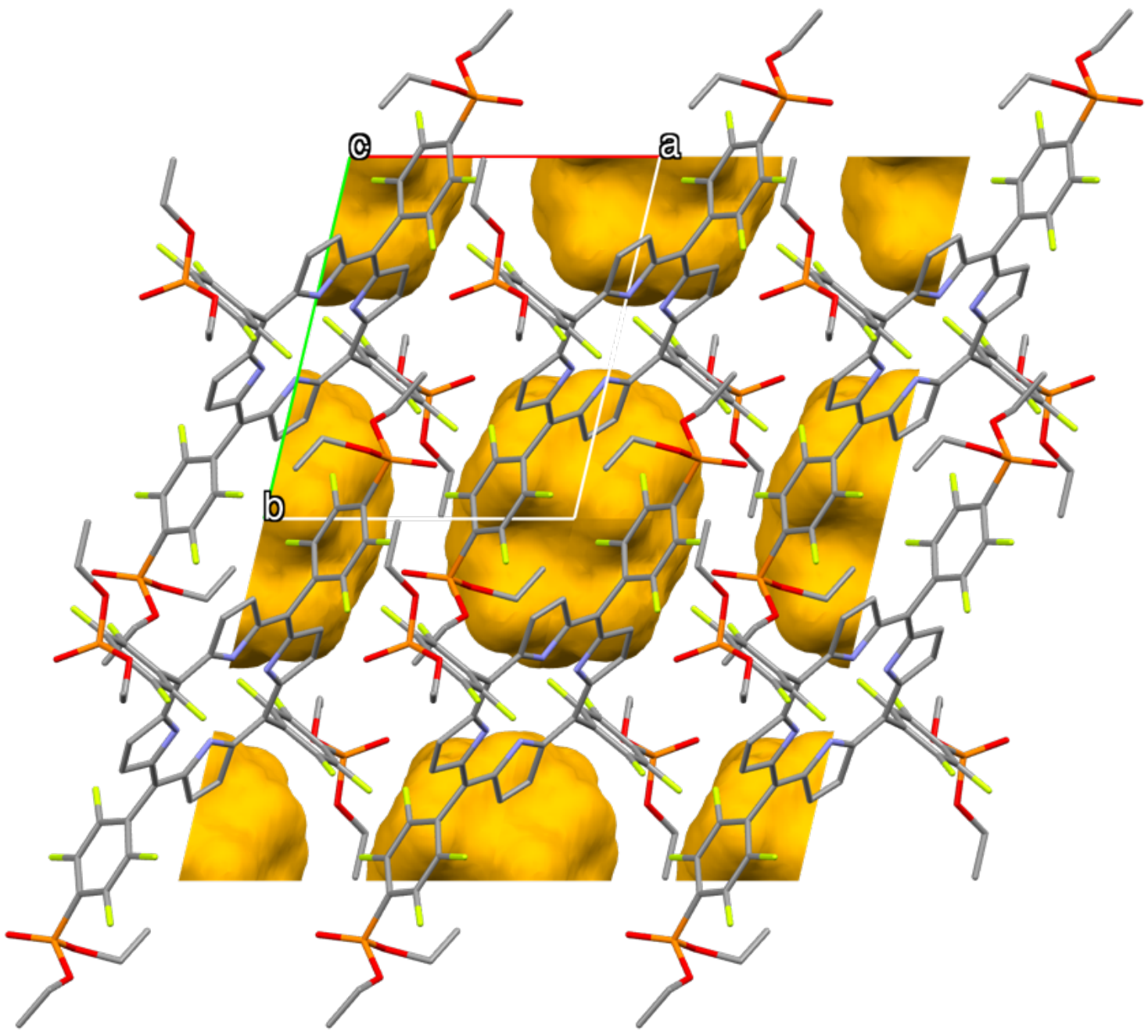

Figure S5. Schematic presentation of molecular packing in the crystals of H_2TF_4PPP (view along the [0 0 1] crystallographic direction). Voids are shown as yellow balls.

Table S4. Bond lengths and angles for H_2TF_4PPP.

Atom–Atom	Length [Å]
P1–O1	1.562(4)
P1–O2	1.565(3)
P1–O3	1.449(3)
P1–C24	1.815(4)
P2–O4	1.559(7)
P2–O5	1.594(8)
P2–O6	1.455(8)
P2–C14	1.817(5)
P2A–O4A	1.531(7)
P2A–O5A	1.597(7)
P2A–O6A	1.419(7)
P2A–C14	1.866(5)
F1–C22	1.340(4)
F2–C23	1.345(4)
F3–C25	1.341(5)
F4–C26	1.350(4)
F5–C16	1.336(6)
F6–C15	1.342(6)
F7–C13	1.332(7)
F8–C12	1.351(6)
O1–C27	1.529(10)
O1–C27A	1.421(11)
O2–C29	1.463(6)
O4–C19	1.534(8)
O4A–C19	1.406(8)
O5–C17	1.469(11)
O5A–C17A	1.467(10)
N1–C1	1.360(5)
N1–C4	1.363(4)
N2–C6	1.368(4)
N2–C9	1.363(5)
C1–C2	1.456(5)
C1–C10[#1]	1.399(5)
C2–C3	1.339(6)
C3–C4	1.452(5)
C4–C5	1.412(5)
C5–C6	1.396(5)
C5–C21	1.486(5)
C6–C7	1.431(5)
C7–C8	1.358(6)
C8–C9	1.424(5)
C9–C10	1.393(5)
C10–C11	1.502(5)
C11–C12	1.363(6)
C11–C16	1.383(6)
C12–C13	1.383(7)
C13–C14	1.383(8)
C14–C15	1.364(7)
C15–C16	1.374(6)
C17–C18	1.425(12)
C17A–C18A	1.468(12)
C19–C20	1.460(10)
C21–C22	1.393(5)
C21–C26	1.380(5)
C22–C23	1.376(5)
C23–C24	1.390(6)
C24–C25	1.384(6)
C25–C26	1.377(5)
C27–C28	1.390(12)
C27A–C28A	1.404(13)
C29–C30	1.491(6)
C1A–C1B	1.4993
C1B–C1C	1.3921
C1B–C1G	1.3922
C1C–C1D	1.3868
C1D–C1E	1.3869
C1E–C1F	1.3869
C1F–C1G	1.3868

Atom–Atom–Atom	Angle [°]
O1–P1–O2	103.1(2)
O1–P1–C24	103.77(18)
O2–P1–C24	106.44(18)
O3–P1–O1	112.6(2)
O3–P1–O2	117.74(18)
O3–P1–C24	111.93(18)
O4–P2–O5	102.1(4)
O4–P2–C14	98.8(3)
O5–P2–C14	112.7(4)
O6–P2–O4	117.8(5)
O6–P2–O5	114.9(5)
O6–P2–C14	109.3(4)
O4A–P2A–O5A	101.9(4)
O4A–P2A–C14	101.8(3)
O5A–P2A–C14	100.9(3)
O6A–P2A–O4A	115.4(5)
O6A–P2A–O5A	118.1(4)
O6A–P2A–C14	116.2(4)
C27–O1–P1	115.2(6)
C27A–O1–P1	132.9(7)
C29–O2–P1	121.6(3)
C19–O4–P2	123.0(5)
C19–O4A–P2A	119.1(6)
C17–O5–P2	121.8(8)
C17A–O5A–P2A	120.8(6)
C1–N1–C4	104.7(3)
C9–N2–C6	110.1(3)
N1–C1–C2	111.3(3)
N1–C1–C10#1	125.8(3)
C10#1–C1–C2	123.0(3)
C3–C2–C1	106.3(3)
C2–C3–C4	106.3(3)
N1–C4–C3	111.4(3)
N1–C4–C5	124.7(3)
C5–C4–C3	123.9(3)
C4–C5–C21	116.7(3)
C6–C5–C4	125.6(3)
C6–C5–C21	117.7(3)
N2–C6–C5	125.8(3)
N2–C6–C7	106.8(3)
C5–C6–C7	127.4(3)
C8–C7–C6	107.9(3)
C7–C8–C9	108.1(3)
N2–C9–C8	107.2(3)
N2–C9–C10	126.6(3)
C10–C9–C8	126.2(3)
C1#1–C10–C11	117.1(3)
C9–C10–C1#1	126.5(3)
C9–C10–C11	116.4(3)
C12–C11–C10	122.6(4)
C12–C11–C16	116.0(4)
C16–C11–C10	121.5(4)
F8–C12–C11	118.9(4)
F8–C12–C13	118.6(5)
C11–C12–C13	122.5(5)
F7–C13–C12	117.0(5)
F7–C13–C14	122.1(4)
C12–C13–C14	121.0(5)
C13–C14–P2	115.9(4)
C13–C14–P2A	130.9(4)
C15–C14–P2	127.4(4)
C15–C14–P2A	112.2(4)
C15–C14–C13	116.7(4)
F6–C15–C14	120.0(4)
F6–C15–C16	118.1(4)
C14–C15–C16	121.9(5)
F5–C16–C11	119.2(4)
F5–C16–C15	118.8(4)
C15–C16–C11	122.0(4)
C18–C17–O5	108.6(9)
O5A–C17A–C18A	109.9(9)
O4A–C19–C20	106.8(6)
C20–C19–O4	110.8(7)
C22–C21–C5	121.7(3)
C26–C21–C5	122.5(3)
C26–C21–C22	115.8(3)
F1–C22–C21	120.0(3)
F1–C22–C23	118.3(3)
C23–C22–C21	121.7(4)
F2–C23–C22	117.6(3)
F2–C23–C24	120.1(3)
C22–C23–C24	122.3(4)
C23–C24–P1	117.7(3)
C25–C24–P1	126.3(3)
C25–C24–C23	115.7(3)
F3–C25–C24	121.0(3)
F3–C25–C26	117.0(3)
C26–C25–C24	122.0(4)
F4–C26–C21	120.1(3)
F4–C26–C25	117.5(3)
C25–C26–C21	122.4(3)
C28–C27–O1	113.8(10)
C28A–C27A–O1	117.1(12)
O2–C29–C30	110.4(5)
C1C–C1B–C1A	120.9
C1C–C1B–C1G	118.1
C1G–C1B–C1A	120.9
C1D–C1C–C1B	121.1
C1C–C1D–C1E	120.1
C1D–C1E–C1F	119.4
C1G–C1F–C1E	120.1
C1F–C1G–C1B	121.1

Bonds and angles to hydrogen atoms were omitted.
Symmetry transformations used to generate equivalent atoms:
#1: -X, 1-Y, -Z;

Table S5. Torsion angles for H_2TF_4PPP.

Atom–Atom–Atom–Atom	Torsion Angle [°]
P1–O1–C27–C28	112.8(12)
P1–O1–C27A–C28A	128.2(13)
P1–O2–C29–C30	−95.0(4)
P1–C24–C25–F3	7.7(6)
P1–C24–C25–C26	−170.8(3)
P2–O4–C19–C20	172.5(7)
P2–O5–C17–C18	128.2(11)
P2–C14–C15–F6	1.0(7)
P2–C14–C15–C16	−179.3(4)
P2A–O4A–C19–C20	175.8(7)
P2A–O5A–C17A–C18A	−171.1(8)
P2A–C14–C15–F6	−2.8(6)
P2A–C14–C15–C16	176.9(4)
F1–C22–C23–F2	−1.0(6)
F1–C22–C23–C24	−179.5(4)
F2–C23–C24–P1	−6.9(5)
F2–C23–C24–C25	178.9(4)
F3–C25–C26–F4	−1.2(5)
F3–C25–C26–C21	−178.7(3)
F6–C15–C16–F5	−1.0(7)
F6–C15–C16–C11	179.3(5)
F7–C13–C14–P2	−2.0(9)
F7–C13–C14–P2A	2.9(10)
F7–C13–C14–C15	177.7(6)
F8–C12–C13–F7	0.0(10)
F8–C12–C13–C14	178.4(6)
O1–P1–O2–C29	160.6(3)
O1–P1–C24–C23	−58.9(4)
O1–P1–C24–C25	114.7(4)
O2–P1–O1–C27	43.8(6)
O2–P1–O1–C27A	35.1(9)
O2–P1–C24–C23	−167.2(3)
O2–P1–C24–C25	6.3(4)
O3–P1–O1–C27	171.8(6)
O3–P1–O1–C27A	163.0(9)
O3–P1–O2–C29	36.0(4)
O3–P1–C24–C23	62.8(4)
O3–P1–C24–C25	−123.6(4)
O4–P2–O5–C17	−156.4(8)
O4–P2–C14–C13	−122.0(5)
O4–P2–C14–C15	58.4(6)
O4A–P2A–O5A–C17A	−160.6(8)
O4A–P2A–C14–C13	−120.6(6)
O4A–P2A–C14–C15	64.4(5)
O5–P2–O4–C19	93.7(7)
O5–P2–C14–C13	−14.8(6)
O5–P2–C14–C15	165.6(5)
O5A–P2A–O4A–C19	70.8(7)
O5A–P2A–C14–C13	−15.8(6)
O5A–P2A–C14–C15	169.2(4)
O6–P2–O4–C19	−33.3(9)
O6–P2–O5–C17	−27.6(10)
O6–P2–C14–C13	114.3(6)
O6–P2–C14–C15	−65.3(6)
O6A–P2A–O4A–C19	−58.5(8)
O6A–P2A–O5A–C17A	−33.0(9)
O6A–P2A–C14–C13	113.2(6)
O6A–P2A–C14–C15	−61.8(5)
N1–C1–C2–C3	0.2(5)
N1–C4–C5–C6	4.9(6)
N1–C4–C5–C21	−176.8(3)
N2–C6–C7–C8	−0.1(5)
N2–C9–C10–C1#1	−0.6(7)
N2–C9–C10–C11	−178.5(4)
C1–N1–C4–C3	−0.6(4)
C1–N1–C4–C5	−179.4(4)
C1–C2–C3–C4	−0.5(5)
C1#1–C10–C11–C12	94.4(5)
C1#1–C10–C11–C16	−86.1(5)
C2–C3–C4–N1	0.7(5)

C2–C3–C4–C5	179.6(4)
C3–C4–C5–C6	−173.8(4)
C3–C4–C5–C21	4.4(6)
C4–N1–C1–C2	0.2(4)
C4–N1–C1–C10$^{\#1}$	178.9(4)
C4–C5–C6–N2	1.9(6)
C4–C5–C6–C7	−177.7(4)
C4–C5–C21–C22	60.3(5)
C4–C5–C21–C26	−116.6(4)
C5–C6–C7–C8	179.6(4)
C5–C21–C22–F1	5.1(6)
C5–C21–C22–C23	−174.0(4)
C5–C21–C26–F4	−3.2(5)
C5–C21–C26–C25	174.2(4)
C6–N2–C9–C8	−1.6(4)
C6–N2–C9–C10	176.0(4)
C6–C5–C21–C22	−121.2(4)
C6–C5–C21–C26	61.8(5)
C6–C7–C8–C9	−0.9(5)
C7–C8–C9–N2	1.5(5)
C7–C8–C9–C10	−176.0(4)
C8–C9–C10–C1$^{\#1}$	176.4(4)
C8–C9–C10–C11	−1.4(6)
C9–N2–C6–C5	−178.6(4)
C9–N2–C6–C7	1.0(4)
C9–C10–C11–C12	−87.5(5)
C9–C10–C11–C16	92.0(5)
C10$^{\#1}$–C1–C2–C3	−178.5(4)
C10–C11–C12–F8	1.8(8)
C10–C11–C12–C13	−179.3(5)
C10–C11–C16–F5	0.0(7)
C10–C11–C16–C15	179.7(4)
C11–C12–C13–F7	−179.0(6)
C11–C12–C13–C14	−0.5(10)
C12–C11–C16–F5	179.5(5)
C12–C11–C16–C15	−0.8(7)
C12–C13–C14–P2	179.7(5)
C12–C13–C14–P2A	−175.5(5)
C12–C13–C14–C15	−0.7(8)
C13–C14–C15–F6	−178.5(5)
C13–C14–C15–C16	1.1(7)
C14–P2–O4–C19	−150.7(6)
C14–P2–O5–C17	98.5(8)
C14–P2A–O4A–C19	174.8(6)
C14–P2A–O5A–C17A	94.8(8)
C14–C15–C16–F5	179.3(5)
C14–C15–C16–C11	−0.4(8)
C16–C11–C12–F8	−177.7(5)
C16–C11–C12–C13	1.2(8)
C21–C5–C6–N2	−176.3(3)
C21–C5–C6–C7	4.1(6)
C21–C22–C23–F2	178.1(4)
C21–C22–C23–C24	−0.4(6)
C22–C21–C26–F4	179.6(3)
C22–C21–C26–C25	−2.9(6)
C22–C23–C24–P1	171.6(3)
C22–C23–C24–C25	−2.6(6)
C23–C24–C25–F3	−178.6(4)
C23–C24–C25–C26	2.9(6)
C24–P1–O1–C27	−67.0(6)
C24–P1–O1–C27A	−75.8(9)
C24–P1–O2–C29	−90.5(3)
C24–C25–C26–F4	177.4(3)
C24–C25–C26–C21	−0.1(6)
C26–C21–C22–F1	−177.8(3)
C26–C21–C22–C23	3.2(6)
C1A–C1B–C1C–C1D	−178.5
C1A–C1B–C1G–C1F	178.5
C1B–C1C–C1D–C1E	−0.1
C1C–C1B–C1G–C1F	−0.3
C1C–C1D–C1E–C1F	−0.1
C1D–C1E–C1F–C1G	0.1
C1E–C1F–C1G–C1B	0.1
C1G–C1B–C1C–C1D	0.3

Bonds and angles to hydrogen atoms were omitted.
Symmetry transformations used to generate equivalent atoms:
#1: -X, 1-Y, -Z;

Table S6. Hydrogen bonds for H_2TF_4PPP.

D–H···A [Å]	d(D–H) [Å]	d(H···A) [Å]	d(D···A) [Å]	<(DHA) [°]
N2–H2···N1	0.88	2.33	2.886(4)	121.1

7. Optical properties

UV–vis spectra were recorded in solutions using a PerkinElmer Lambda 900 UV/VIS/NIR spectrometer (1 cm path length quartz cell). Emission spectra were measured using a Horiba Jobin Yvon Fluorolog-3 spectrometer. Emission quantum yields of all compounds were measured relative to the ZnTPP in acetonitrile (Φ_f = 0.033 [149]) and calculated using a standard procedure [150].

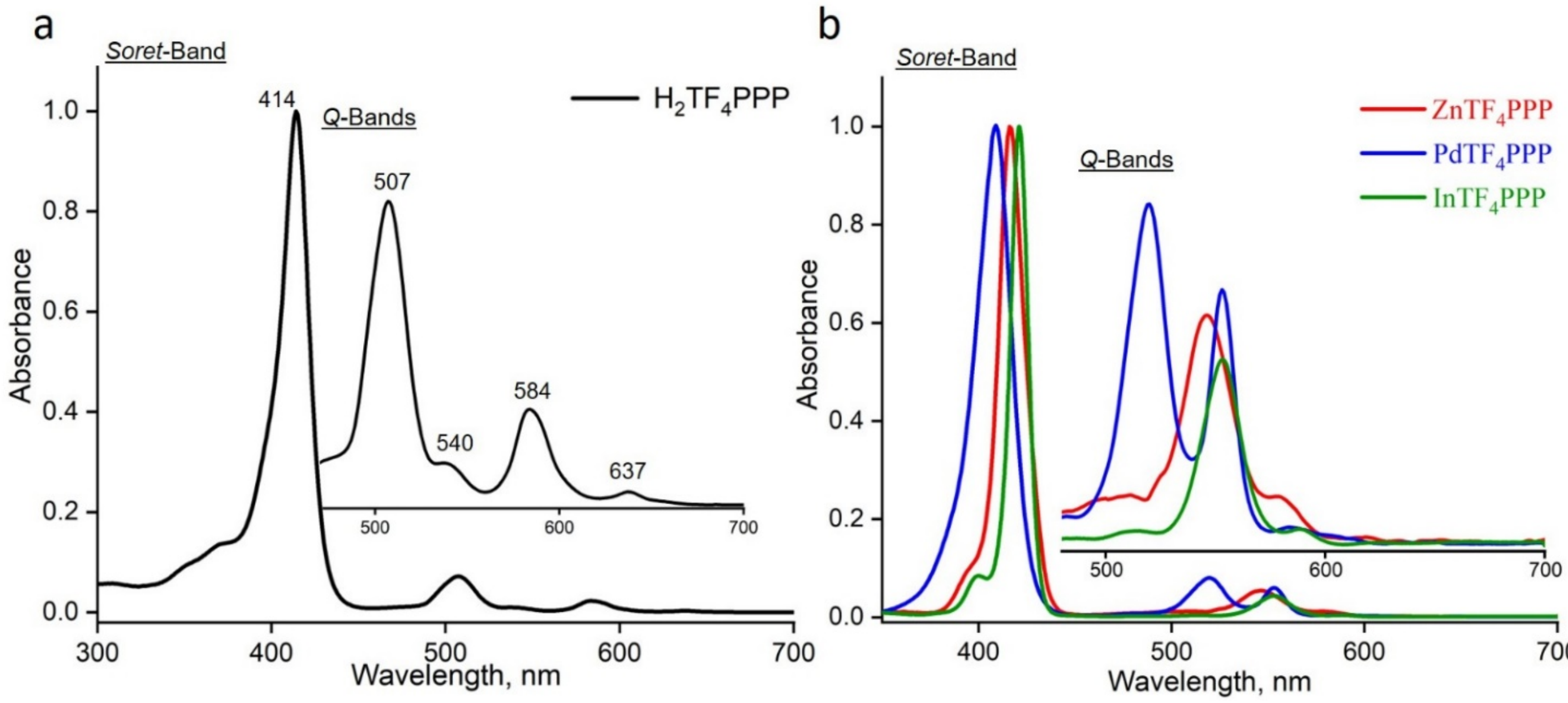

Figure S6. Normalized UV–vis spectra of H_2TF_4PPP (a) and MTF_4PPP (M = Zn(II), Pd(II), In(III)) (b) in dichloromethane at room temperature.

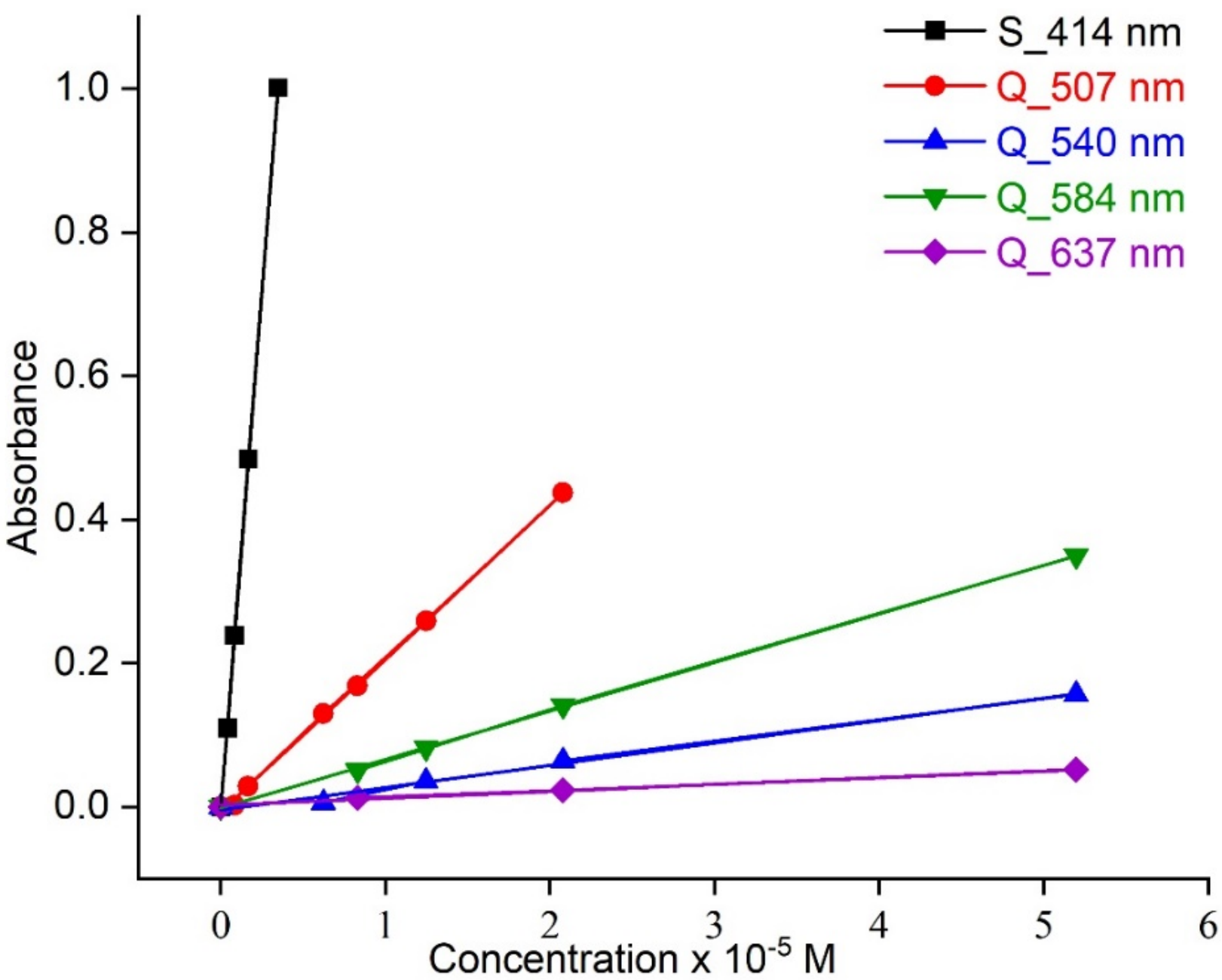

Figure S7. Beer-Lambert's law plots for H_2TF_4PPP in CH_2Cl_2 at room temperature for different absorption bands. The corresponding λ_{max} values are indicated in the legend.

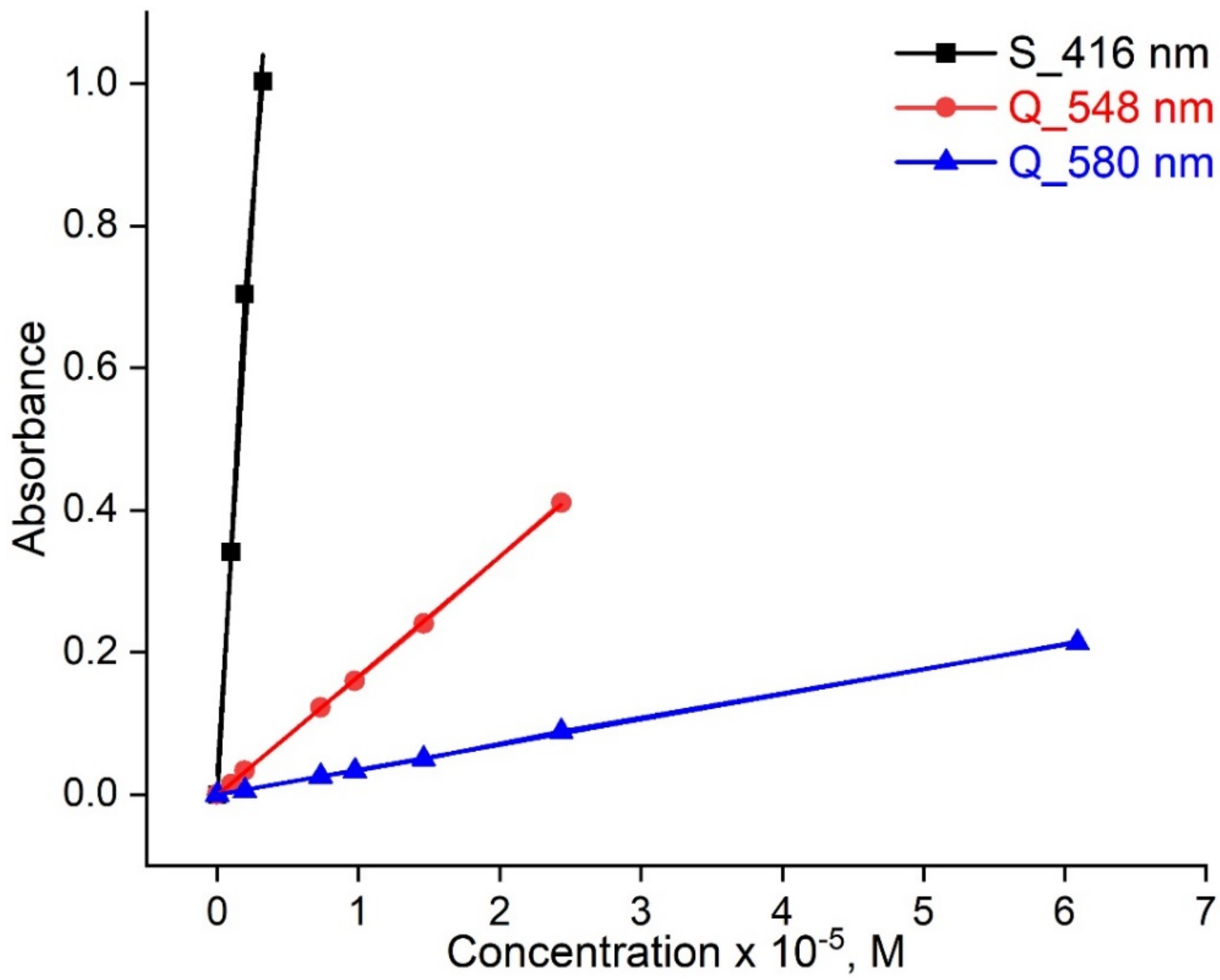

Figure S8. Beer-Lambert's law plots for $ZnTF_4PPP$ in CH_2Cl_2 at room temperature for different absorption bands. The corresponding λ_{max} values are indicated in the legend.

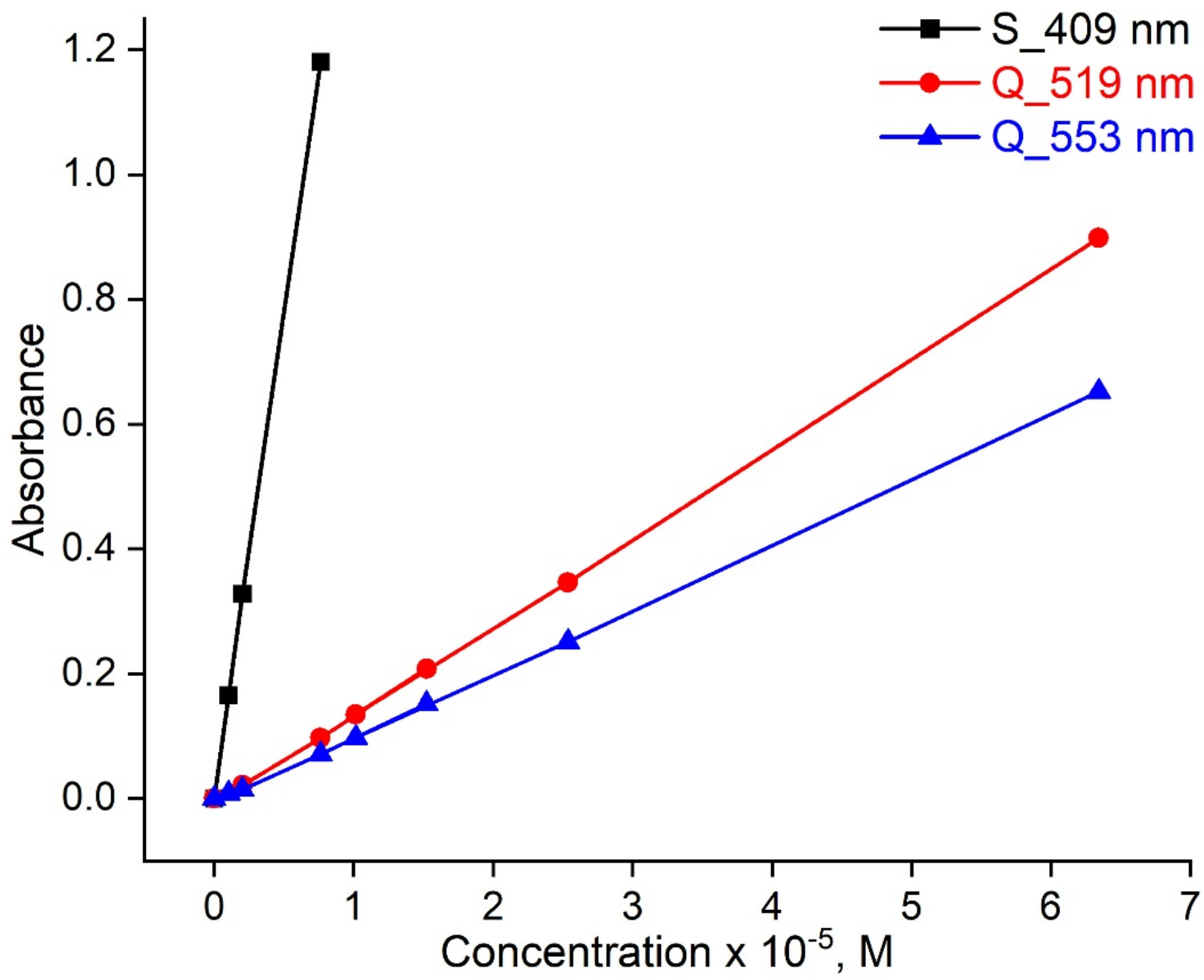

Figure S9. Beer-Lambert's law plots for $PdTF_4PPP$ in CH_2Cl_2 at room temperature for different absorption bands. The corresponding λ_{max} values are indicated in the legend.

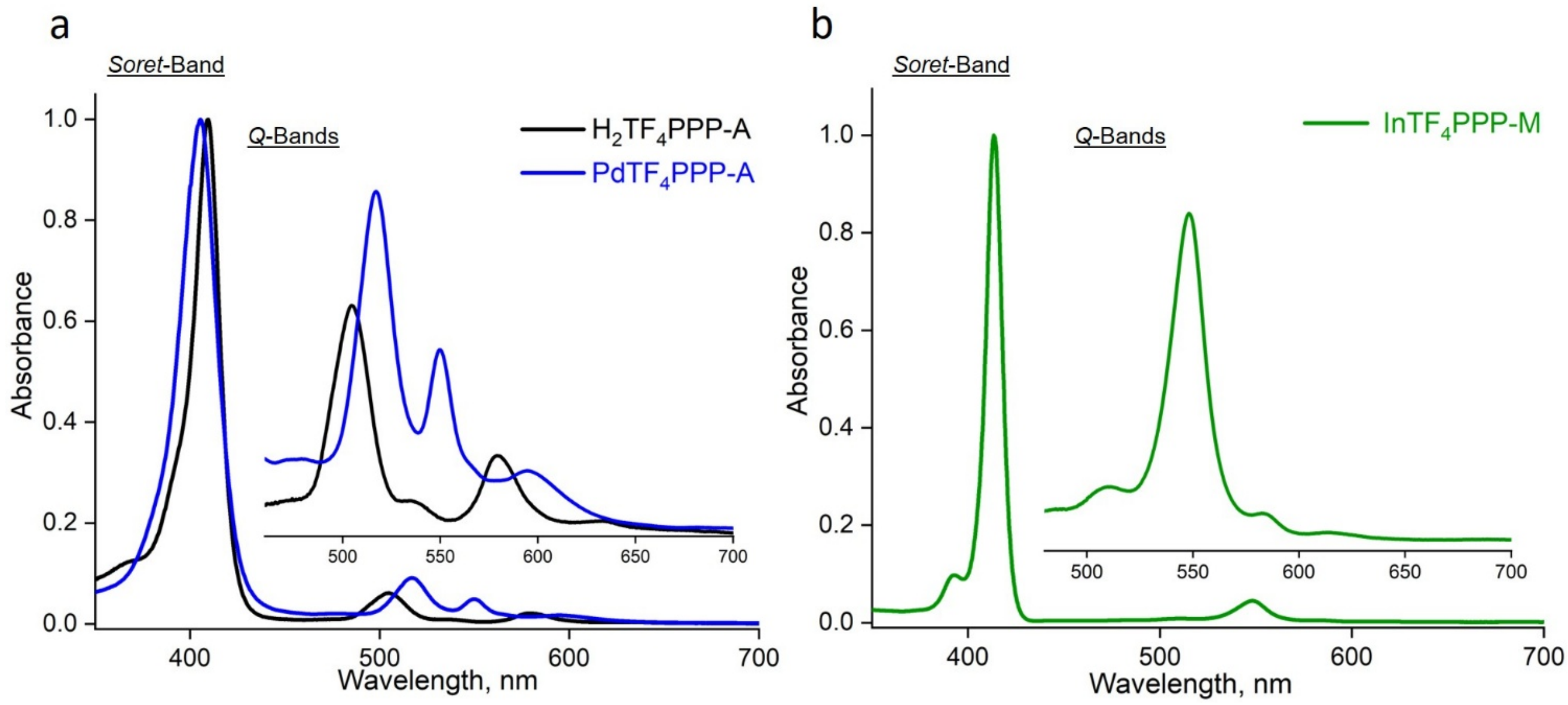

Figure S10. Normalized UV–vis spectra of MTF_4PPP-A (M = $2H^+$, Pd(II)) in slightly basified MeCN/H_2O (1:1 v/v) (a); $InTF_4PPP$-M in MeOH (b).

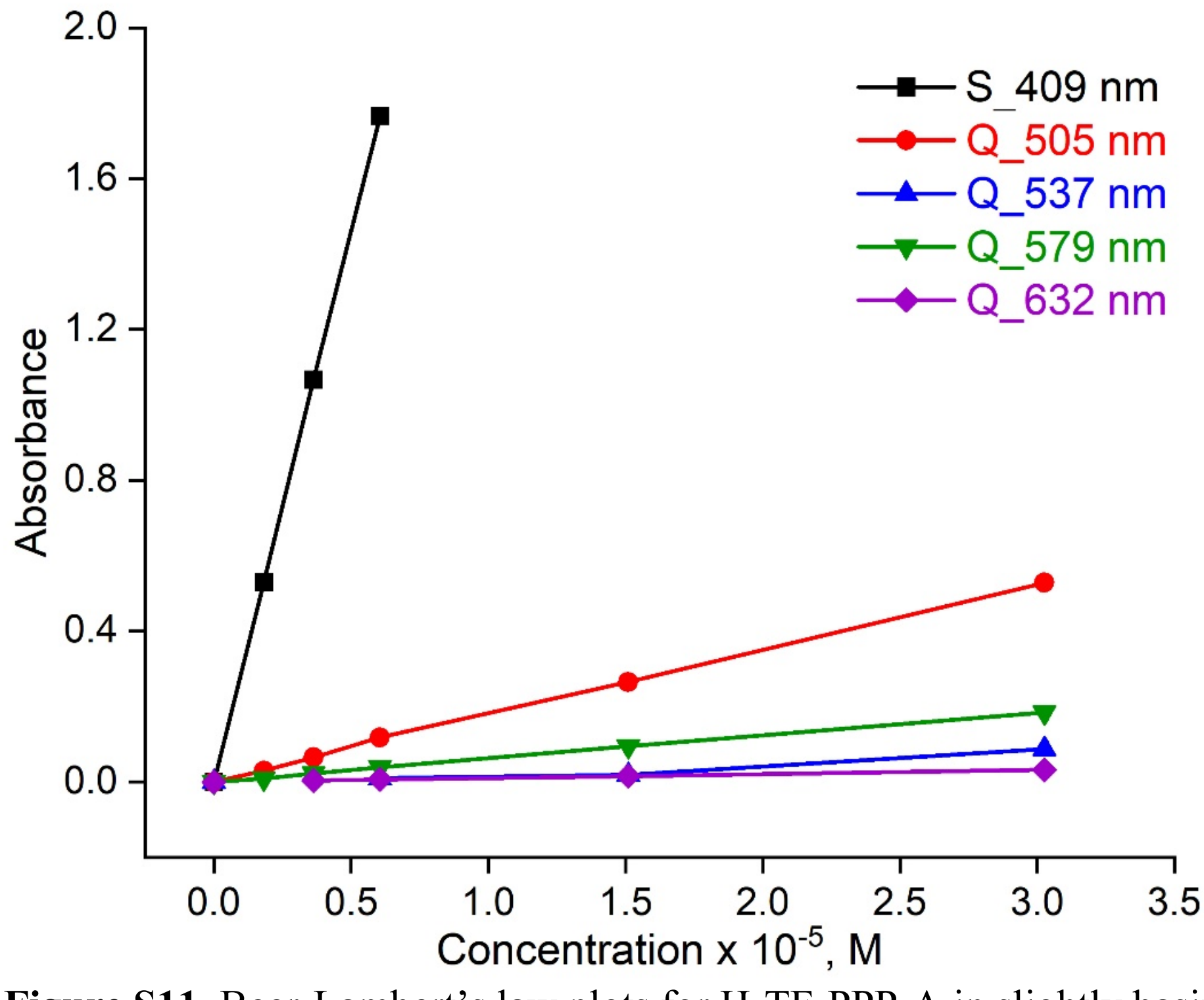

Figure S11. Beer-Lambert's law plots for H_2TF_4PPP-A in slightly basified MeCN/H_2O (1:1 v/v) for different absorption bands. The corresponding λ_{max} values are indicated in the legend.

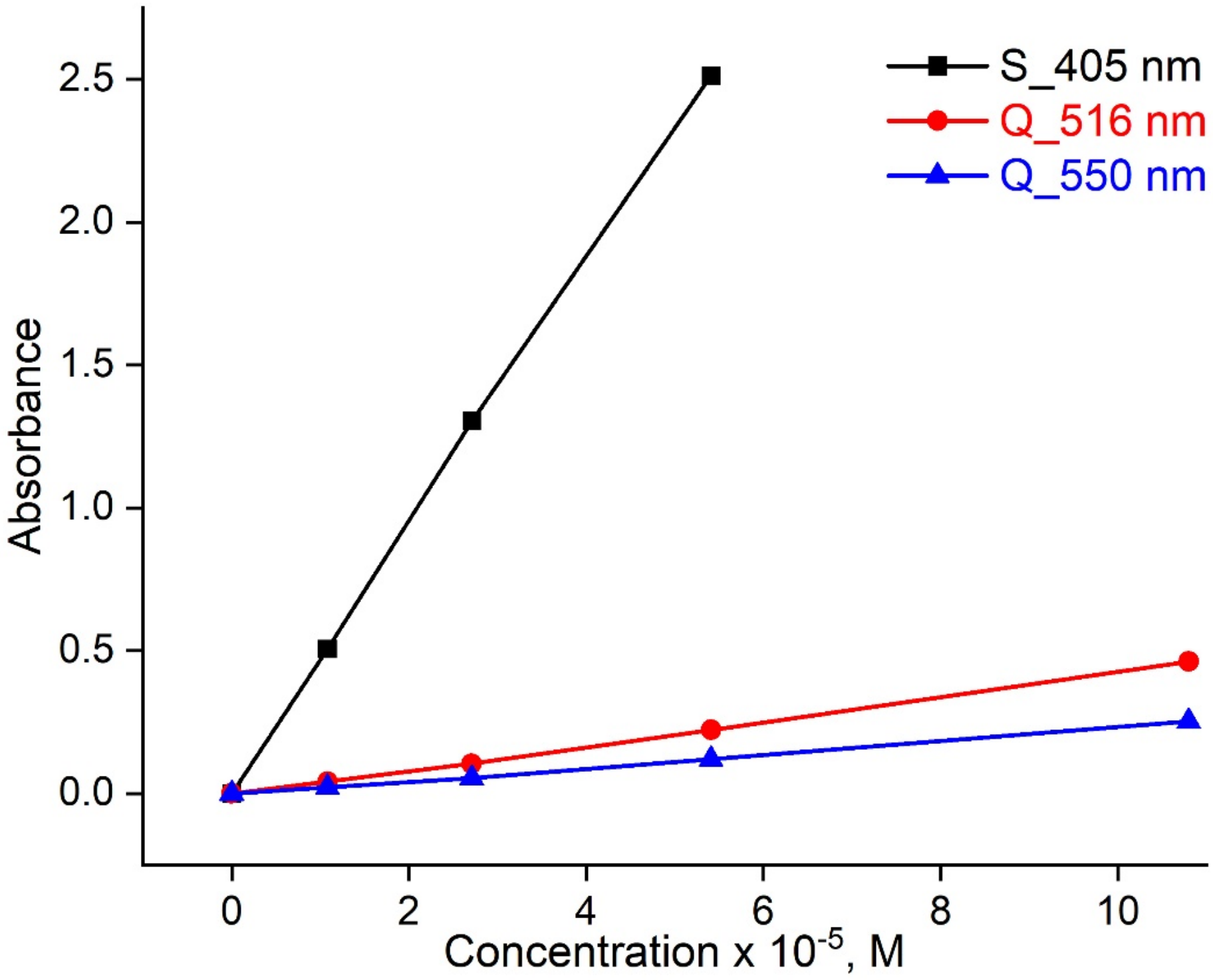

Figure S12. Beer-Lambert's law plots for $PdTF_4PPP$-A in slightly basified $MeCN/H_2O$ (1:1 v/v) at room temperature for different absorption bands. The corresponding λ_{max} values are indicated in the legend.

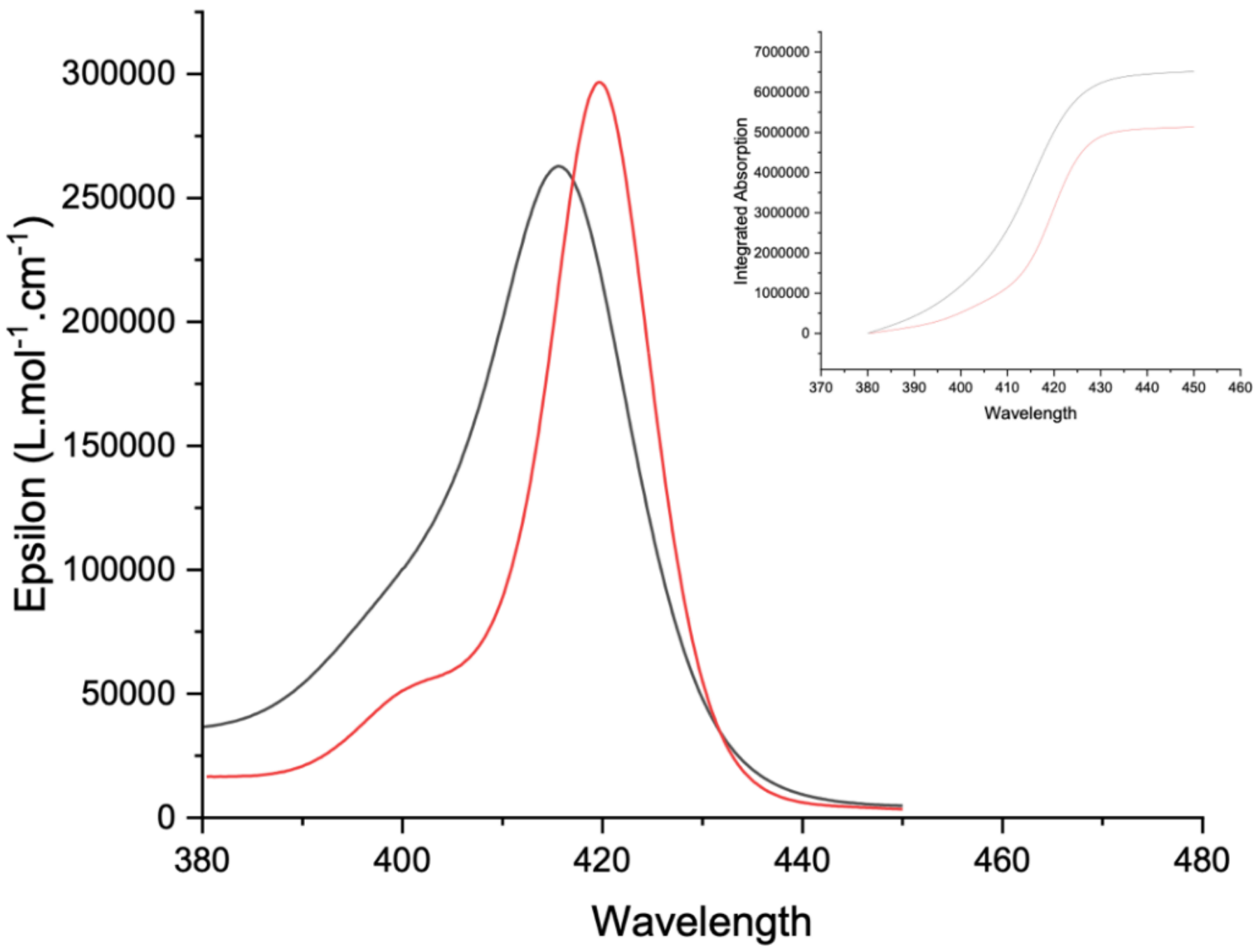

Figure S13. UV–vis spectra of H_2TPPP (red line) and H_2TF_4PPP (black line) in chloroform. Insert: Absorbance integrated over the Soret region.

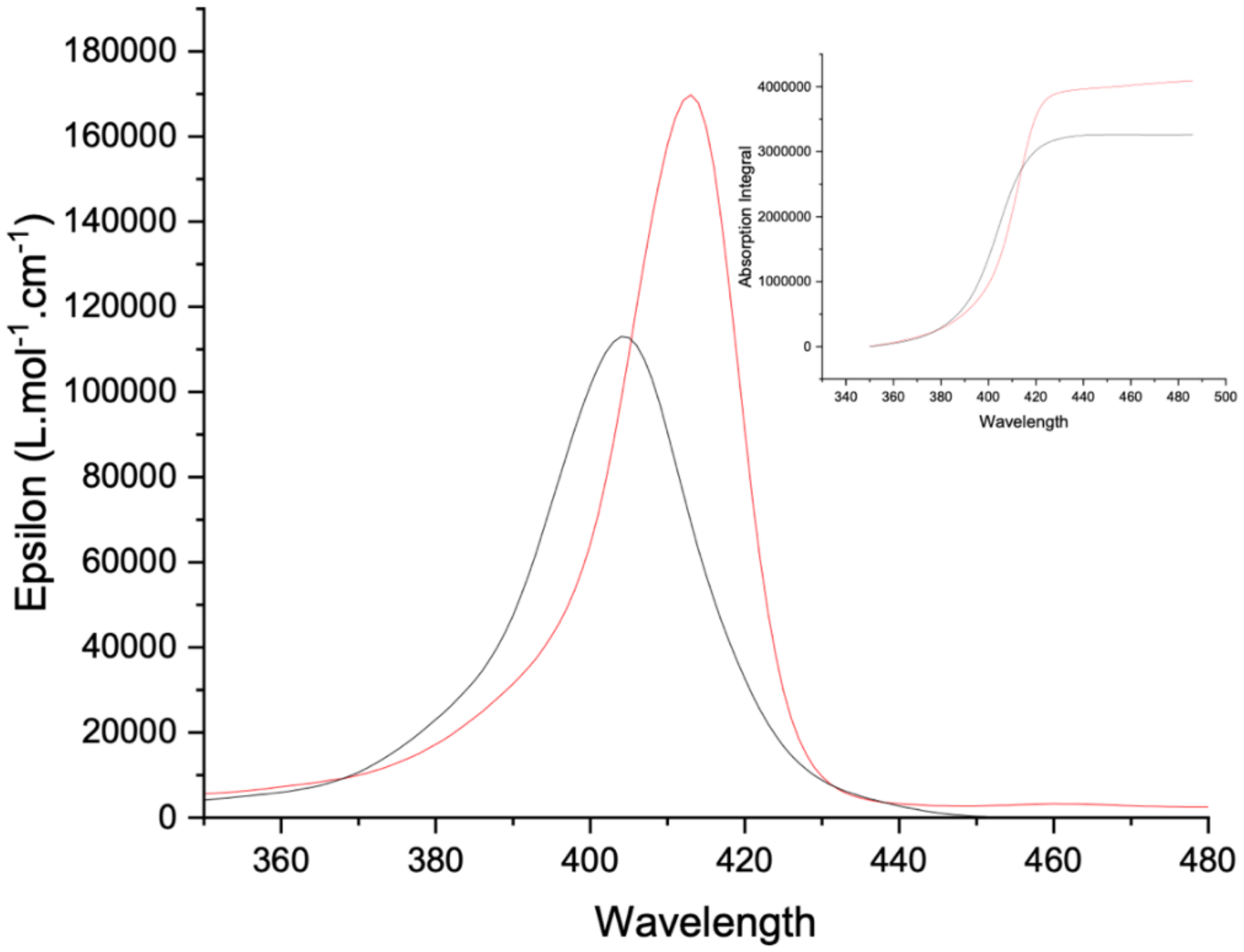

Figure S14. UV–vis spectra of PdTPPP (red line) and PdTF$_4$PPP (black line) in chloroform. Insert: Absorbance integrated over the Soret region.

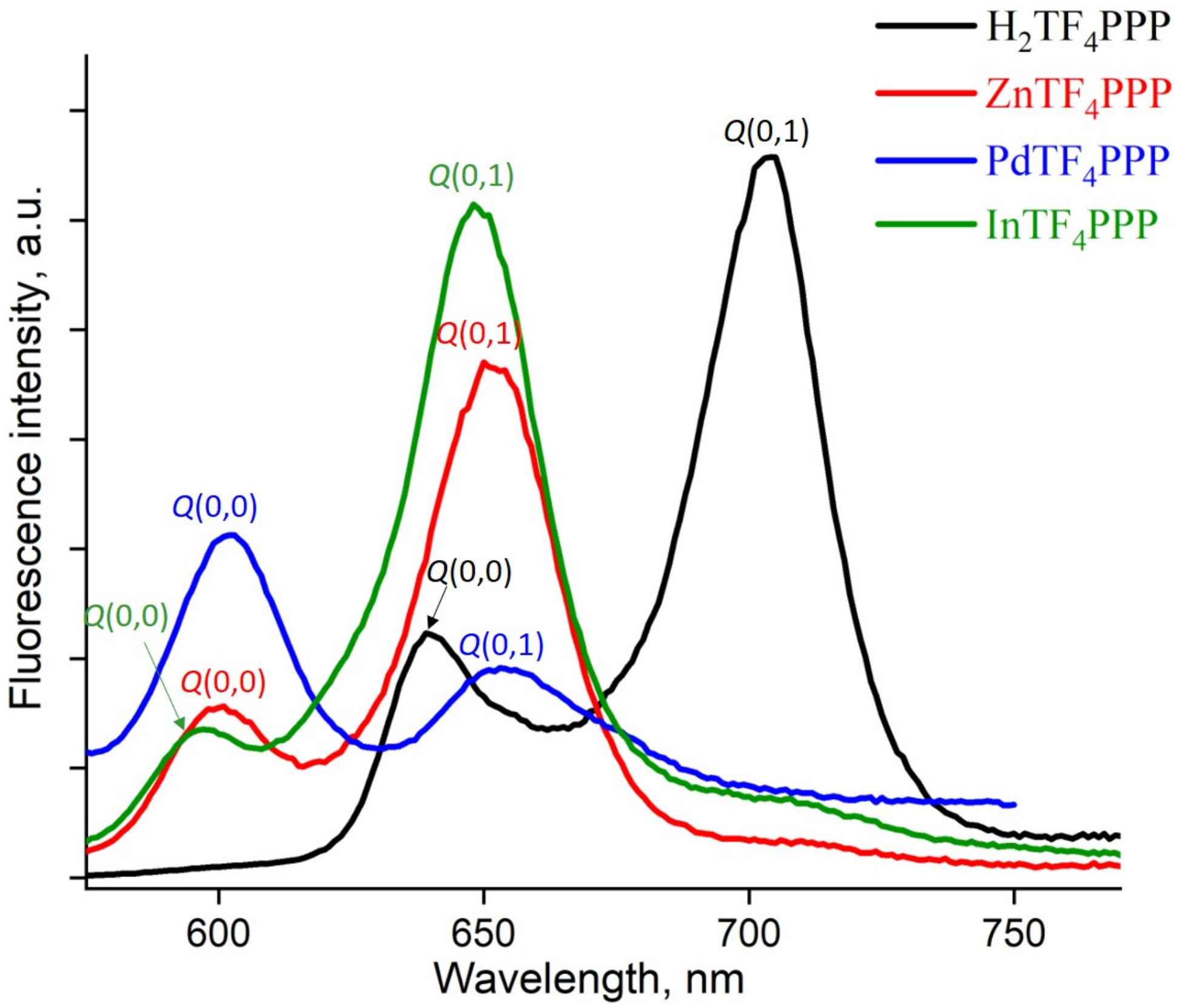

Figure S15. Emission spectra of MTF$_4$PPP (M = 2H$^+$, Zn(II), Pd(II), In(III)) in acetonitrile (c = 1 μM and λ_{ex} = 547 nm) at room temperature and in air.

8. Studies on the single oxygen generation

The photophysical measurements were carried out in chloroform by direct detection of the singlet oxygen phosphorescence signal at 1270 nm. Singlet oxygen emission spectra were measured using a Horiba Jobin Yvon Fluorolog-3 fluorimeter, equipped with a three-slit double-grating excitation and emission monochromator with a dispersion of 2.1 nm mm^{-1} (1200 grooves mm^{-1}). The steady-state luminescence was excited by unpolarized light from a 450 W xenon continuous wave (CW) lamp at the desired excitation wavelength, and with excitation slits opening of 10 nm. It was detected at an angle of 90° for measurements of dilute solutions (10 mm quartz cuvette, 15 nm emission slits opening, integration time = 1s) using a liquid nitrogen cooled solid indium/gallium/arsenide (InGaAs) detector (850–1600 nm). Spectra were corrected for both excitation source light-intensity variation and emission spectral responses (signal "T_{1c}/R_{1c}").

Singlet oxygen quantum yield (φ_Δ) measurements for MTF_4PPP and MTPPP were achieved using a relative methodology based on the comparison of $^1O_2$ phosphorescence intensity of diluted chloroform solutions (optical density (OD) < 0.1) of studied samples against that of phenalen-1-one (**PH**) in the same solvent (φ_Δ = 0.95 [112]) used here as a reference compound. Singlet oxygen luminescence quantum yields were calculated using the following equation

$$\varphi_\Delta^x = \varphi_\Delta^r \left[\frac{A_r(\lambda_r)}{A_x(\lambda_x)}\right]\left[\frac{I_x}{I_r}\right]$$

where $A(\lambda)$ is the absorbance (or optical density) at the excitation wavelength and I is the corrected integrated luminescence intensity. The subscripts r and x stand for reference and sample, respectively. The reported results are the average of 3–4 independent measurements at various absorbances (comprised between 0.01 and 0.1) for both sample and reference. The plot of the integrated luminescence intensity *vs* absorbance gives straight line with excellent correlation coefficients and the associated slopes can be determined for both sample and reference, allowing straightforward determination of the sample $^1O_2$ generation efficiency (Figure S16).

Chloroform was chosen here, as it ensures that the singlet oxygen lifetime is long enough to enable detection and quantitative evaluation of its phosphorescence signal with enough accuracy and a high signal-to-noise ratio. Measurements were performed upon irradiation in the Soret band of porphyrins (at *ca* 415 nm), first to match the conditions of the catalytic experiments, all performed under blue light irradiation, and second to enable a systematic comparison with phenalen-1-one (perinaphthenone, **PH**), which was used as a standard.

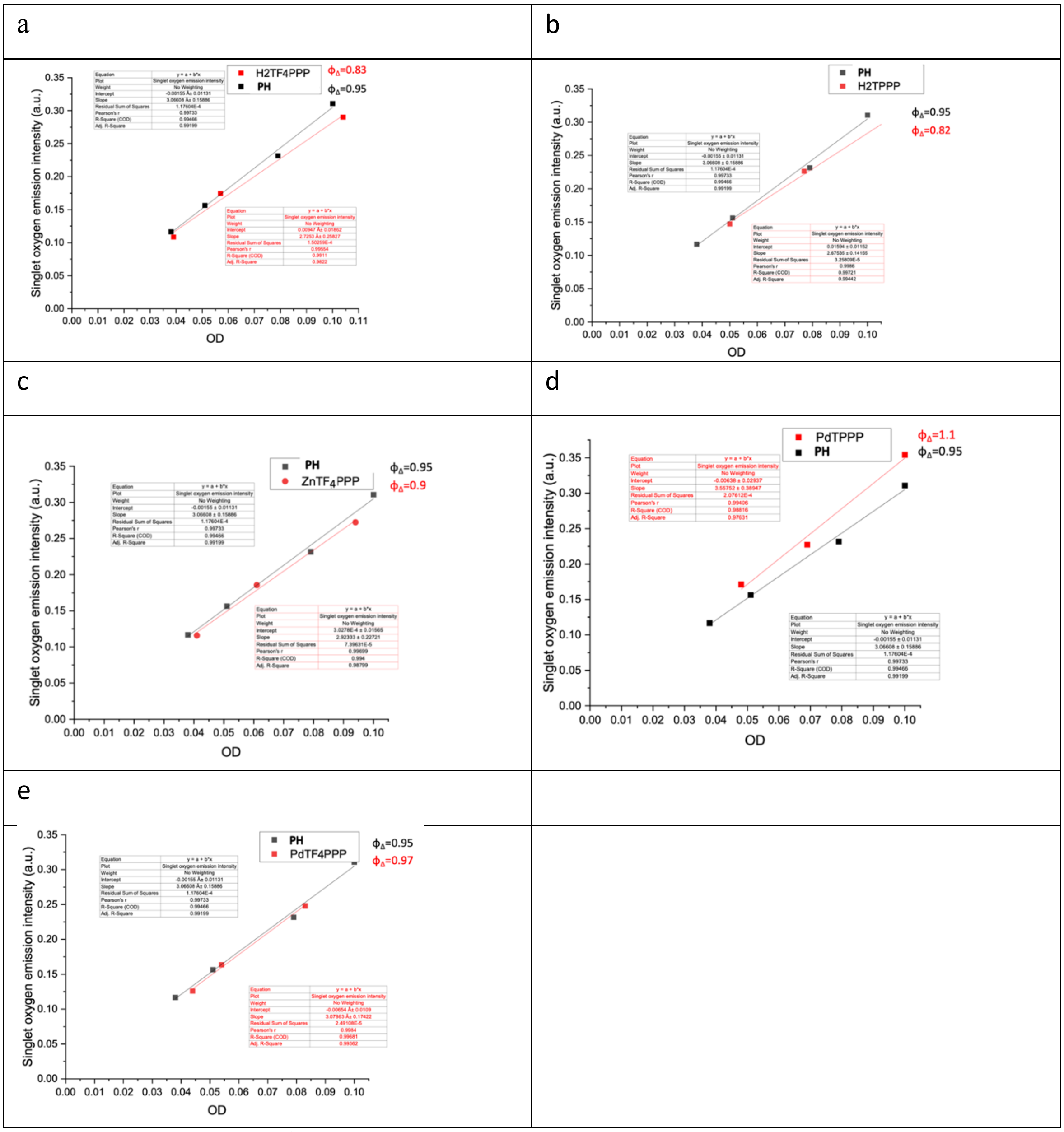

Figure S16. Plot of the $^1O_2$ emission intensity vs absorbance of $CHCl_3$ solutions for phenalen-1-one (**PH**) (black squares) and porphyrins (red circles): H_2TPPP (a), H_2TF_4PPP (b), PdTPPP (c), $PdTF_4PPP$ (d), $ZnTF_4PPP$ (e). Linear fits, with the respective color code. **PH** taken as a reference ($\varphi_\Delta = 0.95$ in chloroform) gives values for H_2TF_4PPP $\varphi_\Delta = 0.83$; $PdTF_4PPP$ $\varphi_\Delta = 0.97$; $ZnTF_4PPP$ $\varphi_\Delta = 0.90$; for H_2TPPP $\varphi_\Delta = 0.82$ PdTPPP $\varphi_\Delta = 1.1$).

9. Photostability in solution

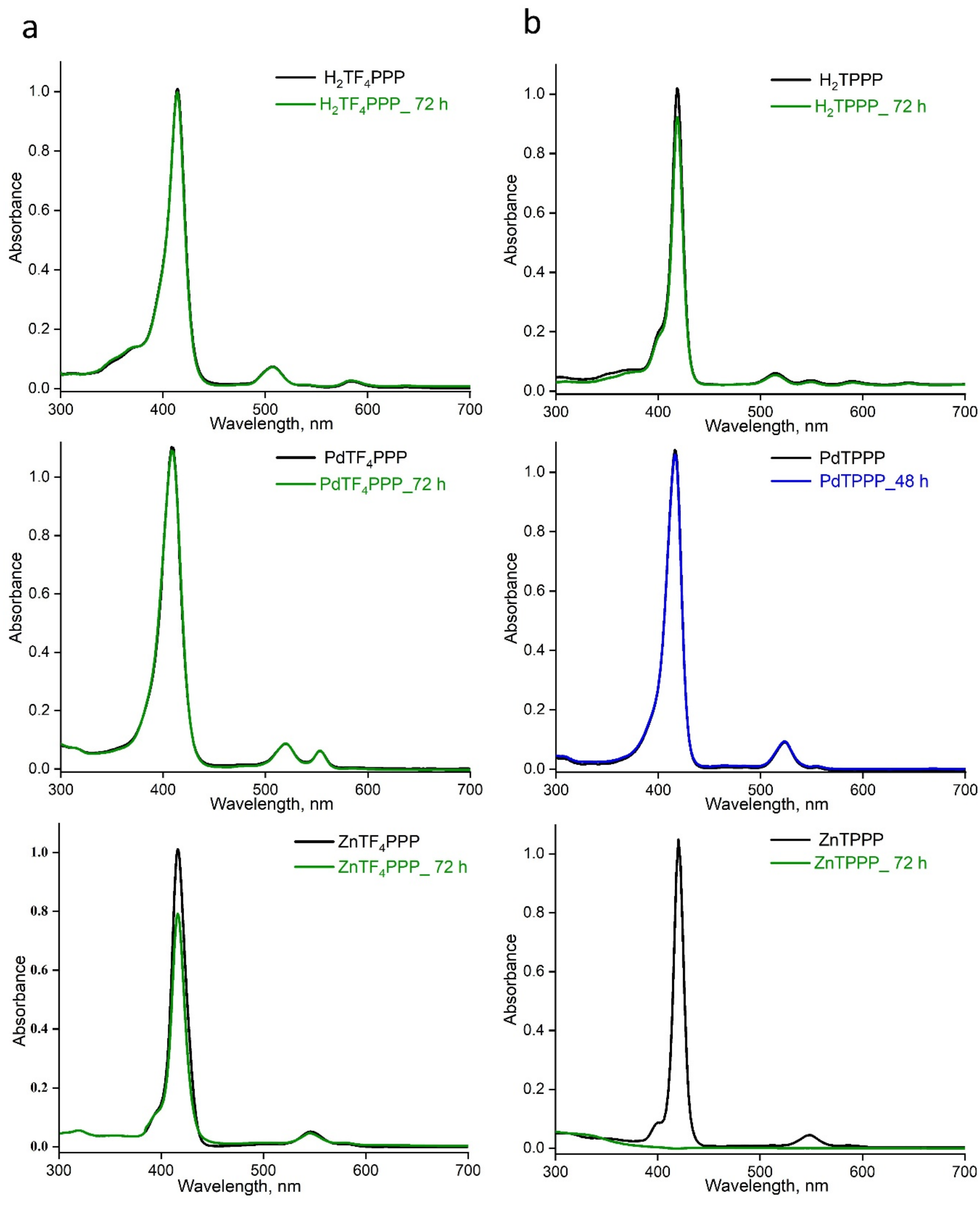

Figure S17. Evolution of the UV-vis spectra of MTF_4PPP (a), MTPPP (b) (M = *2*H^+, Pd(II)[5], Zn(II)[5]) in dichloromethane under irradiation with a blue LED (450 nm, 3 W).

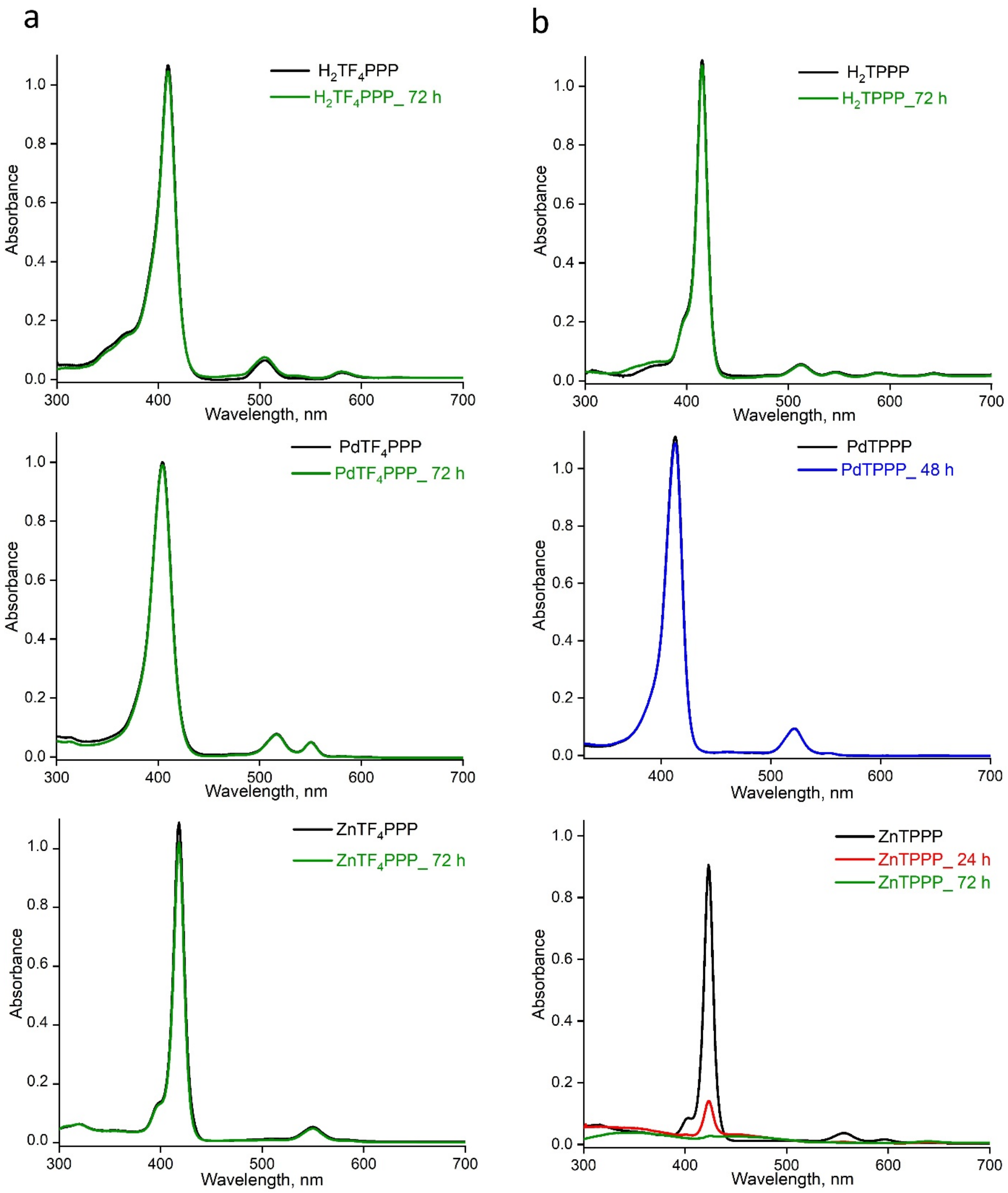

Figure S18. Evolution of the UV-vis spectra of MTF_4PPP (a), MTPPP (b) ($M = 2H^+$, Pd(II)[5], Zn(II)[5]) in MeCN under irradiation with a blue LED (450 nm, 3 W).

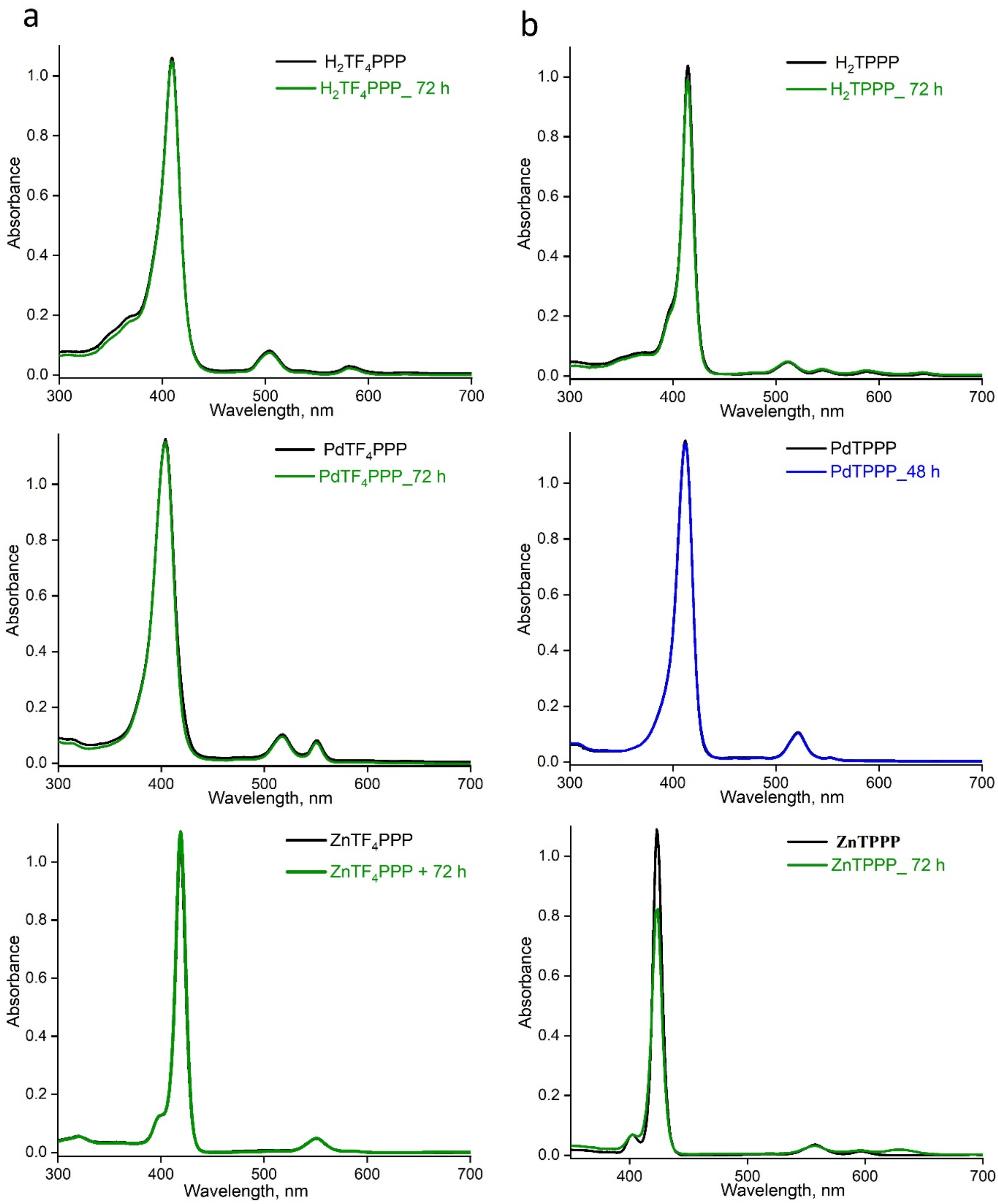

Figure S19. Evolution of the UV-vis spectra of MTF4PPP (a), MTPPP (b) (M = $2H^+$, Pd(II)[5], Zn(II)[5]) in MeOH under irradiation with a blue LED (450 nm, 3 W,).

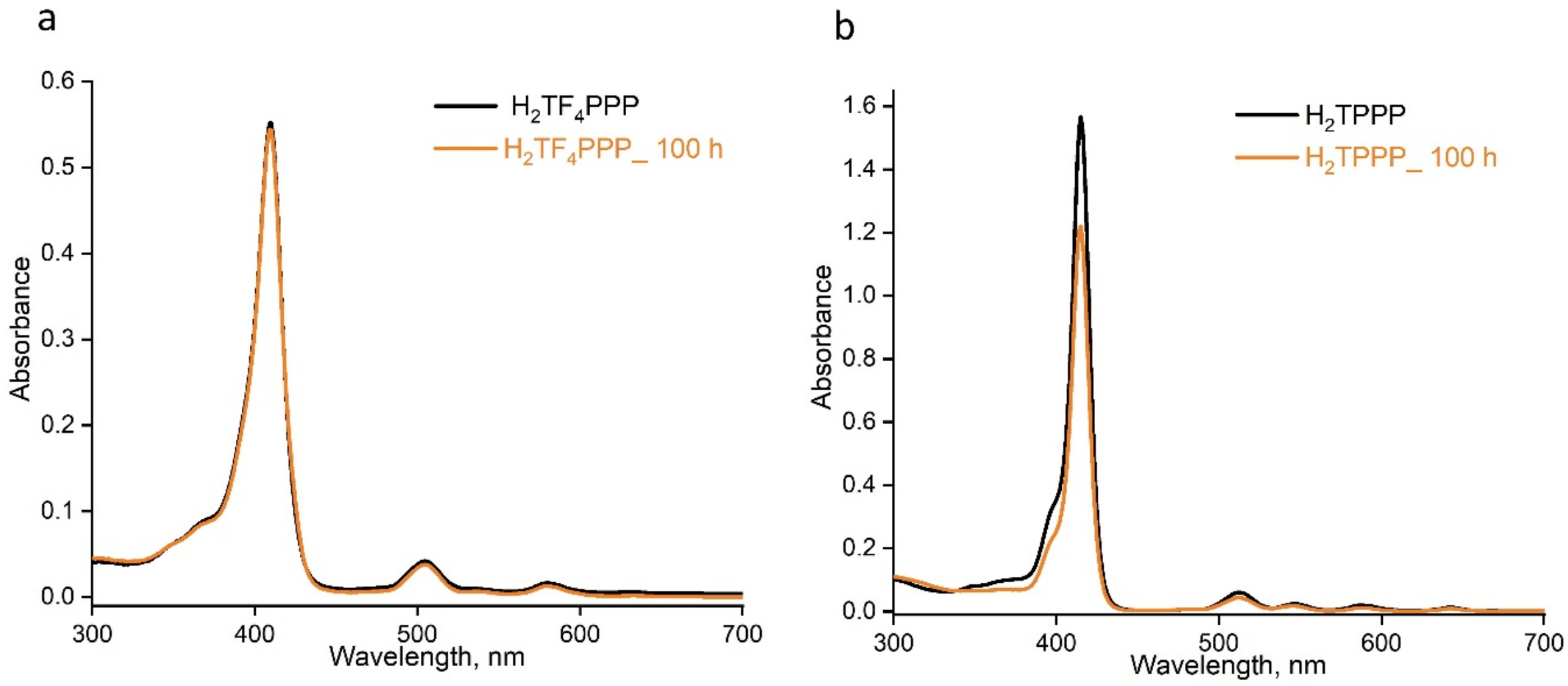

Figure S20. Evolution of the UV-vis spectra of H_2TF_4PPP (a), H_2TPPP (b) in MeCN/H_2O (1:1 v/v) mixture under irradiation with a blue LED (450 nm, 3 W).

10. Photocatalysis

Standard 0.01 M solutions of H_2TF_4PPP in MeCN were prepared by dissolving 0.05 mmol of the porphyrin in 5 mL volumetric flasks.

General procedure of sulfoxidation in the presence of M(TPPP). A glass vial equipped with a magnetic stir bar was charged with 0.05 mmol of sulfide and the calculated amount of standard solution of the photocatalyst. Then acetonitrile and water were added to give an MeCN/water reaction mixture (2.75 mL, 10:1 v/v). The reaction was irradiated with a blue LED while gently bubbling oxygen. When the reaction was complete (^{1}H NMR analysis), the mixture was diluted with 7 mL of water and extracted with dichloromethane (3 × 5 mL). The combined extracts were dried over sodium sulfate and evaporated under reduced pressure at room temperature. The yield and purity of the products were determined by ^{1}H NMR using biphenyl as an internal standard.

The catalyst loading and the reaction time are indicated in Table 3 and S7.

This catalysts loading was also used in kinetic studies (Figure S21) which were performed by using the same procedure. The reactions were periodically monitored by NMR spectroscopy after withdrawing aliquot samples.

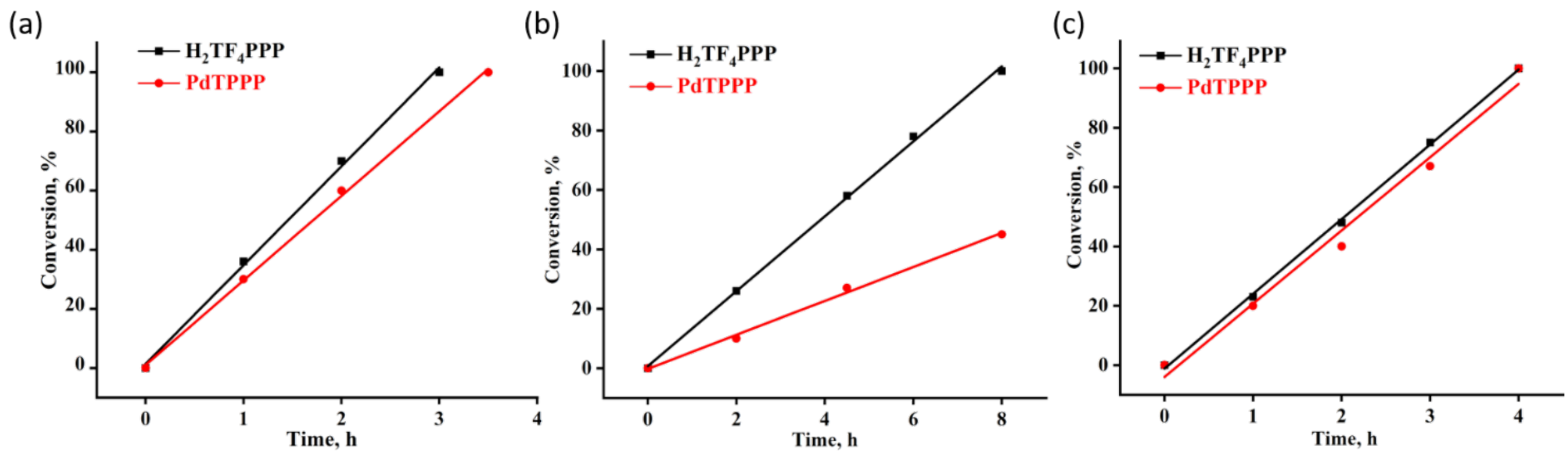

Figure S21. Comparative studies of catalytic efficiency of H_2TF_4PPP and PdTPPP in the oxidation of (a) thioanisole, (b) 4-nitrothionisole, and (c) dibutyl sulfide.

Table S7. Photooxidation of sulfides in the presence of H_2TF_4PPP and H_2TPPP.[a]

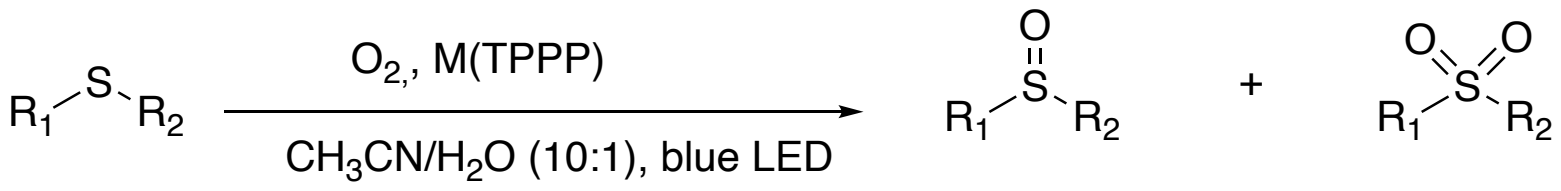

Entry	Sulfide	Photocatalyst (mol%)	Time (h)	Conversion[b] (%)	Yield[b] (%) Sulfoxide	Sulfone	Reference
1		H_2TF_4PPP					
	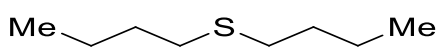	(0.005)	4	100	99	1	
2		H_2TPPP		40			[51]
		(0.005)	4	100	100	0	
3		H_2TF_4PPP					
4	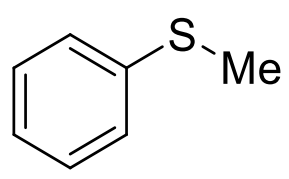	(0.05)	3	100	100	0	
		H_2TPPP					
		(0.05)	2.5	100	98	2	[51]
		H_2TF_4PPP					
	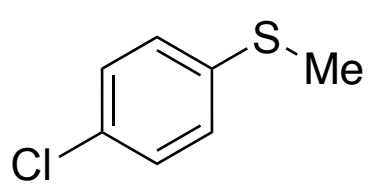	(0.05)	2.5	100	100	0	
		(0.025)	3.5	100	100	0	
		H_2TPPP					
		(0.025)	4.5	100	99	1	[51]
8		H_2TF_4PPP					
	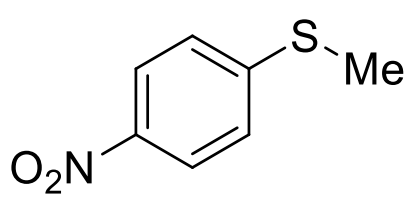	(0.05)	8	100	99	1	
9		H_2TPPP					
		(0.05)	8	45	100	0	[51]
10		H_2TF_4PPP					
		(0.025)	4	100	99.5	0.5	
	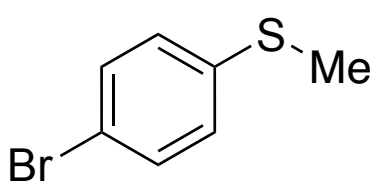	H_2TPPP					
		(0.05)	4.5	100	99	1	[51]
11		H_2TF_4PPP					
	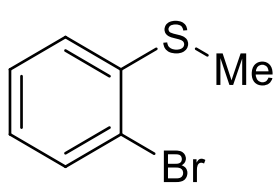	(0.05)	7	100	98	2	
		H_2TPPP					
		(0.05)	7	100	98	2	[51]
12	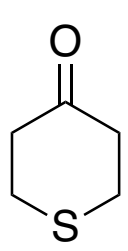	H_2TF_4PPP					
		(0.01) H_2TPPP	4	100	100	0	
		(0.1)					
			4	100	98	2	[51]
13		H_2TF_4PPP					
	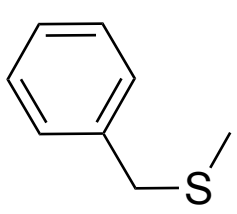	(0.005)	6	100	100	0	
		H_2TPPP					
		(0.005)	7	100	99	1	[51]

[a] Reaction conditions: 1 mmol of sulfide and PC were stirred and irradiated by blue LED (450 nm, 3 W) in MeCN/H_2O mixture (2.75 mL, 10:1, v/v), bubbling slowly with molecular oxygen at room temperature. [b] Conversion and selectivity were determined by ^1H NMR analysis of reaction mixtures using biphenyl as an internal standard.

11. Interfacial behavior of H_2TF_4PPP

Fabrication of Langmuir monolayers and LS films

A 1000-2 KSV Minitrough ($l \times w$ = 36.4 × 7.5 cm) model (KSV Instrument Ltd., Helsinki, Finland) equipped with a platinum Wilhelmy plate was used for the fabrication of Langmuir monolayers. The trough and barriers were made of Teflon®. Deionized water (18.2 MΩ cm, pH ~5.5) produced by a Vodoley cartridge purificator (SPE Himelektronika, Russia) was used as a subphase. Monolayers were formed by spreading a freshly prepared 0.05 mM chloroform solution of the porphyrins onto a water surface. The spreading solution was deposited by means of a Gilson "Distriman" micropipette in 2.5 µL portions. The overall solution volume deposited at the air–water interface was 200 µL and the delivered drops were uniformly distributed over the available surface of water. All measurements were carried out at room temperature (23 ± 1 °C).

After the sample had been spread, the solvent was allowed to evaporate for 15 min. The monolayer was compressed at a rate of 5 mm min^{-1}. Each compression isotherm was recorded at least three times to ensure the reproducibility of the results. Limiting molecular areas (A_0), were determined by interpolation of the steep rise region of the isotherm to zero $mN{\cdot}m^{-1}$.

The compressibility modulus was calculated using equation (1)

$$C_s^{-1} = -A(d\pi/dA) \quad (1)$$

where A is the surface area per molecule at a given surface pressure π.

LS films were deposited onto PVC and mica substrates. The transfer of Langmuir monolayers was performed at a constant surface pressure of 18 mN m^{-1} by a horizontal deposition onto a solid substrate. The transfer ratio for the LS films was no less than 0.95 for both substrates.

UV–vis and fluorescence spectroscopies studies

In situ UV–vis spectra of Langmuir monolayers at the air–water interface were recorded in the wavelength range of 200–750 nm using an AvaSpec–2048 FT–SPU (Avantes) fiber-optic spectrophotometer equipped with a 75 W DH-2000 deuterium-halogen light source and a CCD array detector. The UV–vis reflectometric probe with a fiber-optic diameter of 400 µm, combined with a 6 fibers-irradiating bundle, was placed perpendicular to the studied surface at a distance of 2–3 mm. The signal reflected from the water surface prior to the deposition of the monolayer was used as a baseline.

The fluorescence spectra of Langmuir monolayers were recorded using a fiber optic spectrophotometer AvaSpec-2048. The monolayer was irradiated with the light of a diode placed above the monolayer to provide a 45° angle of incidence of the excitation light with respect to the subphase

surface. The wavelength of the exciting light (λ = 420 nm) was close to λ_{max} of the Soret absorption bands of the studied compounds.

The UV–vis spectra of solutions and LS films transferred onto PVC substrates were recorded on a SHIMADZU-2450 spectrophotometer in the wavelength range of 300–700 nm. The fluorescence spectra of these films were recorded using a Shimadzu RF-5301 spectrofluorometer in the wavelength range of 600–800 nm. The UV–vis and fluorescence spectra of solutions were recorded by using a rectangular quartz cell (Hellma, 100-QS, 45 × 12.5 × 12.5 mm, light path 10 mm, chamber volume 3.5 mL).

Atomic force microscopy (AFM) studies

AFM measurements were performed on MultiMode Nanoscope V (Veeco, USA) operating in tapping mode and scratching experiments were achieved in the contact mode. Noncontact polysilicon cantilevers with a high aspect ratio were used (Resonant frequency 120 kHz, Q-factor 350), produced by TipsNano, Estonia. Gwyddion software was used for image processing.

Photostability studies

Photostability studies of single-layer LS films were carried out in two ways. The first method: the films were placed at the same distance from 365 nm LED (3W) and irradiated for 1200 min monitoring by UV–vis and fluorescence spectroscopies every 60 min. The absorbance changes over time were recorded at the maximum of the Soret band (428 and 430 nm for H_2TF_4PPP and H_2TPPP, respectively). The fluorescence changes over time were monitored at the maximum of Q(0,1) band (710 and 720 nm for H_2TF_4PPP and H_2TPPP, respectively). The second method: LS films were placed in a spectrofluorometer, the slits were opened and fluorescence intensity was continuously recorded during 60 min at the maximum of Q(0,1) band (710 nm and 720 nm for H_2TF_4PPP and H_2TPPP, respectively). The light source was a xenon lamp of the spectrofluorometer (150 W, excitation slits opening of 5 nm, λ_{ex} = 430 nm).

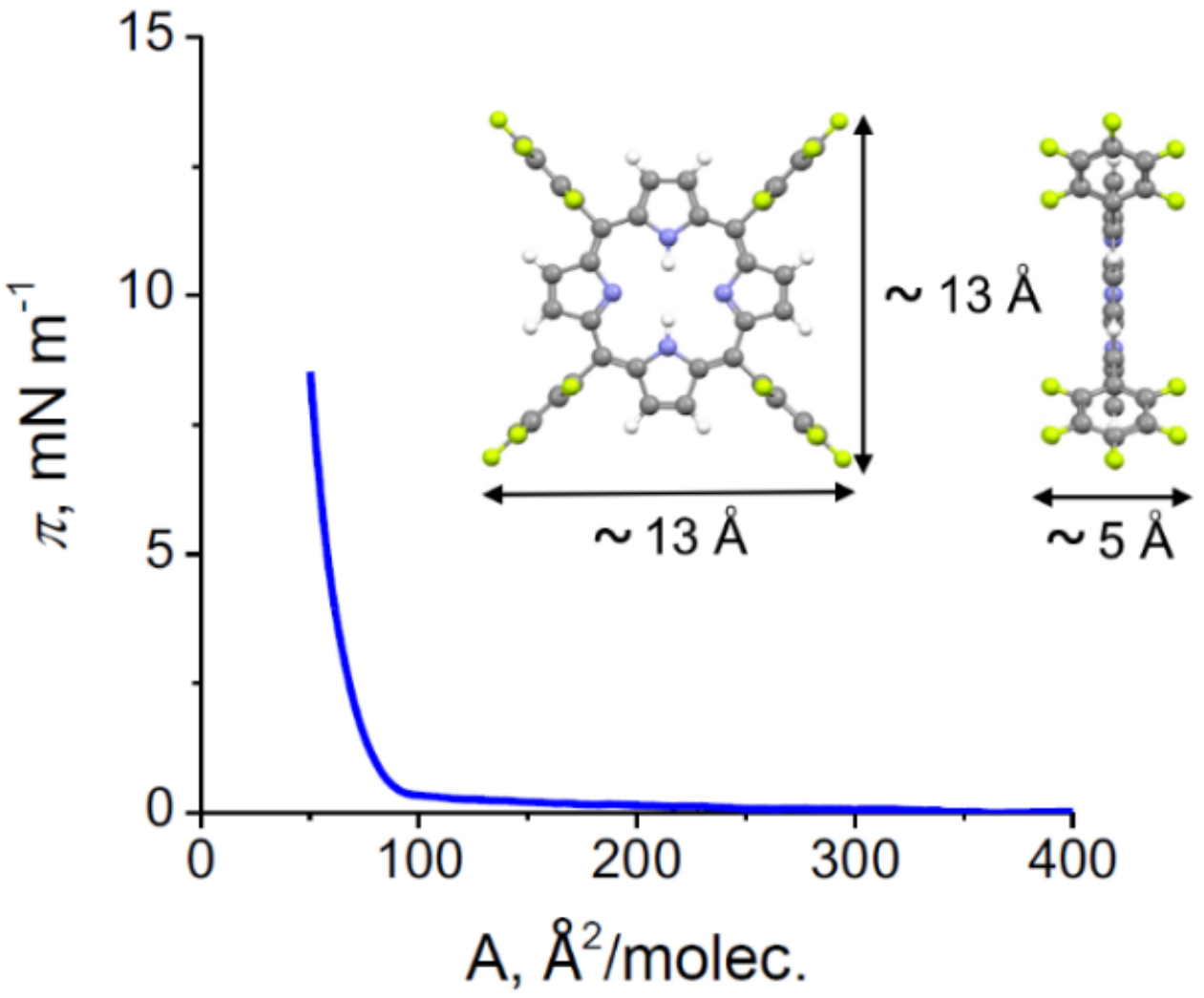

Figure S22. Surface pressure–area isotherm of H_2TF_5PP monolayer on the deionized water surface. Insert: Space fill model of H_2TF_5PP molecule optimized by the semi-empirical PM6 method [138].

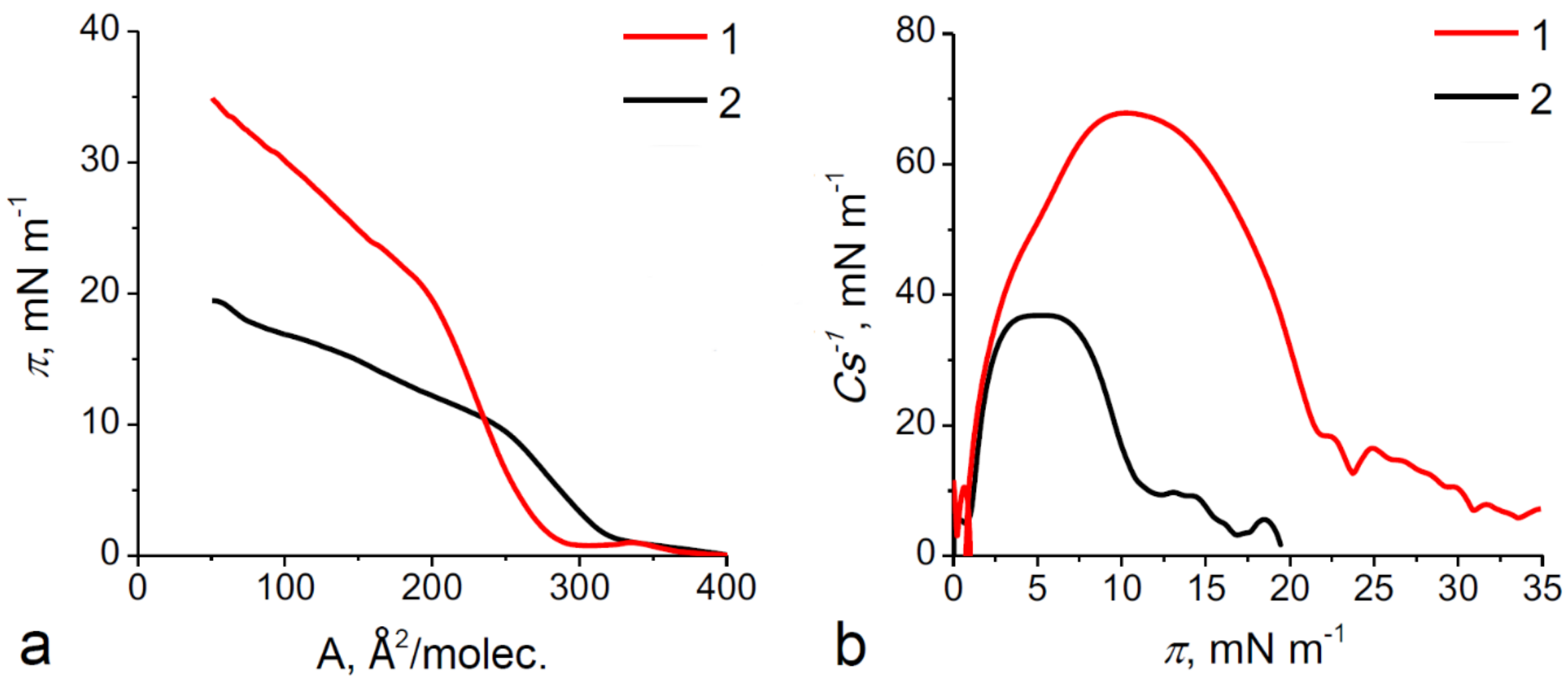

Figure S23. Surface pressure–area isotherms (a) and compressibility modulus C_s^{-1} vs. surface pressure plots (b) for H_2TF_4PPP (1) and H_2TPPP (2) monolayers on the deionized water surface.

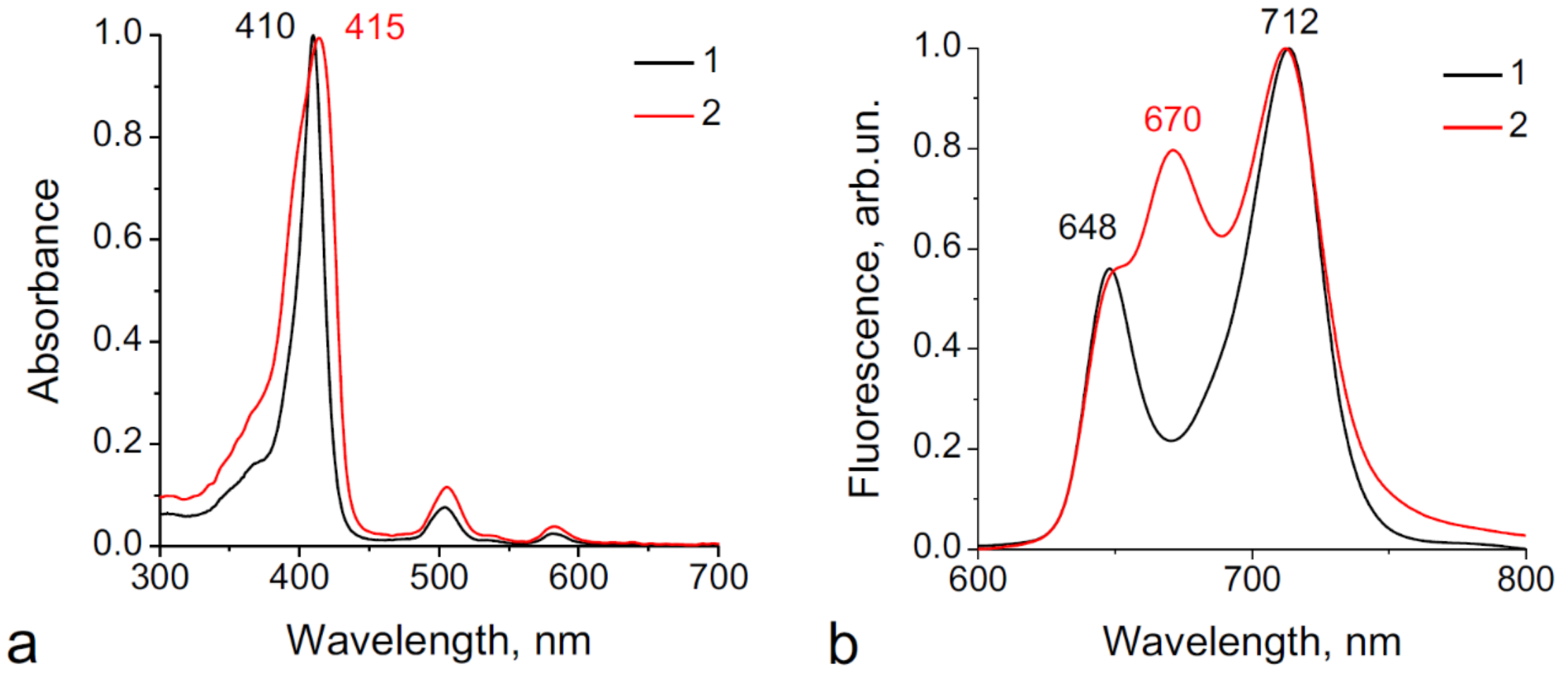

Figure S24. Normalized [0; 1] UV–vis (a) and fluorescence (b) spectra (λ_{ex} = 410 nm) of H_2TF_4PPP solution in ethanol (1) and ethanol/water (1:9 v/v) mixture (2).

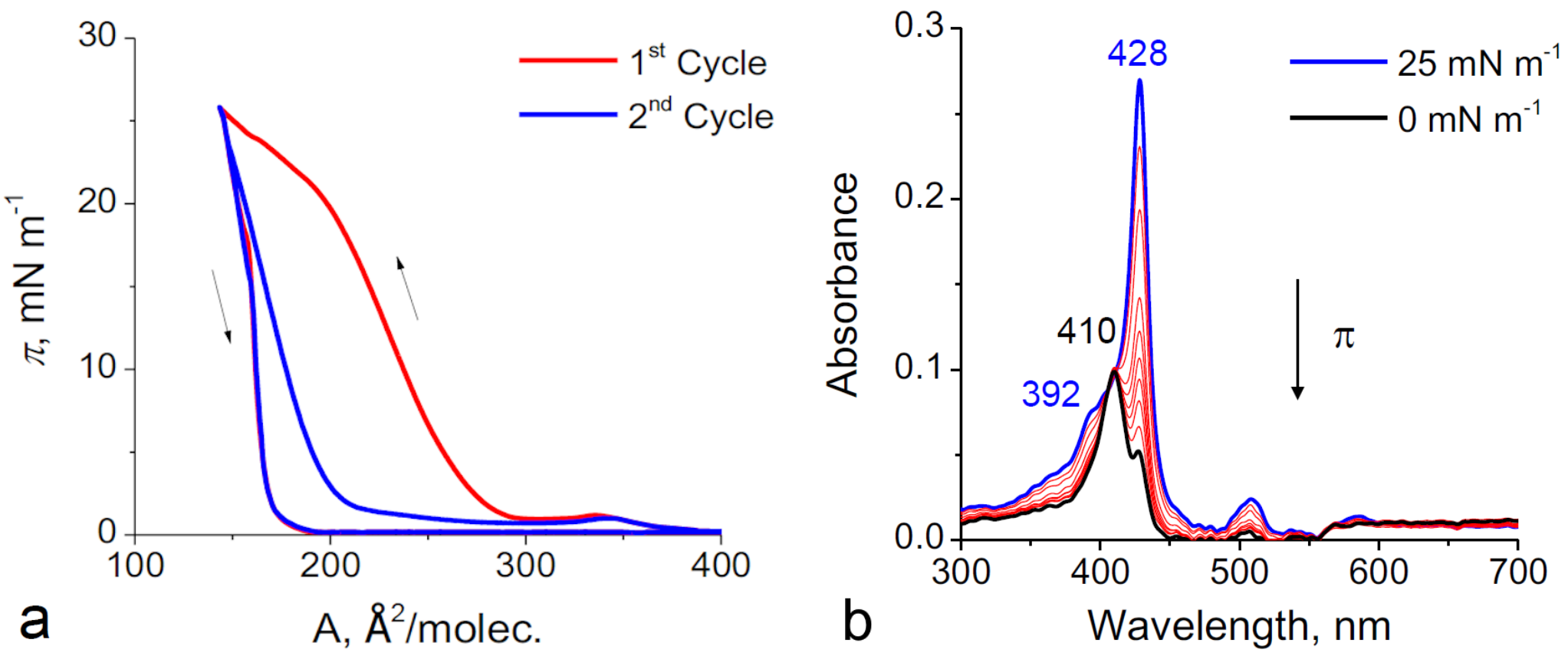

Figure S25. Compression–expansion cycles for the H_2TF_4PPP monolayer (a). *In situ* UV–vis spectra of the H_2TF_4PPP monolayer recorded during the monolayer expansion at 1st cycle (b).

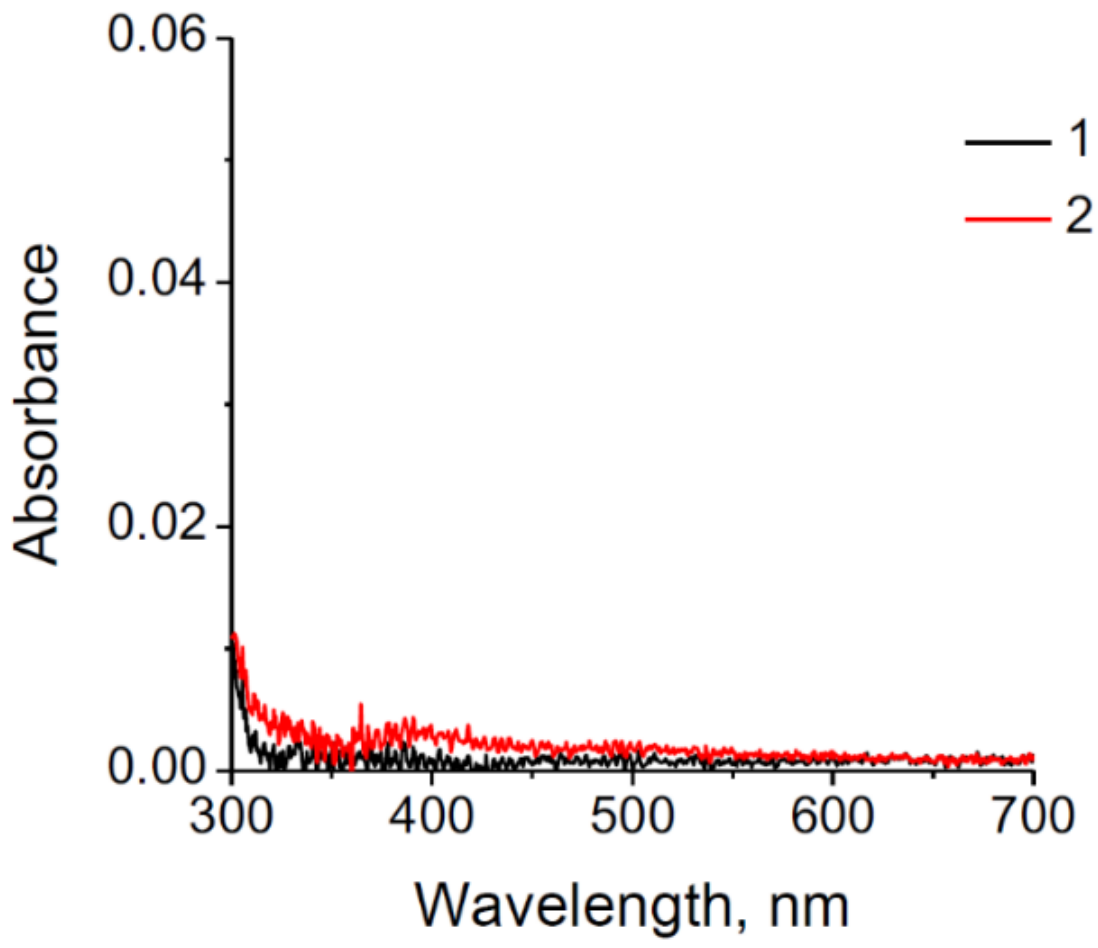

Figure S26. UV–vis spectra of aqueous subphase after compression–expansion cycle of H_2TF_4PPP (1) and H_2TPPP (2) monolayers.

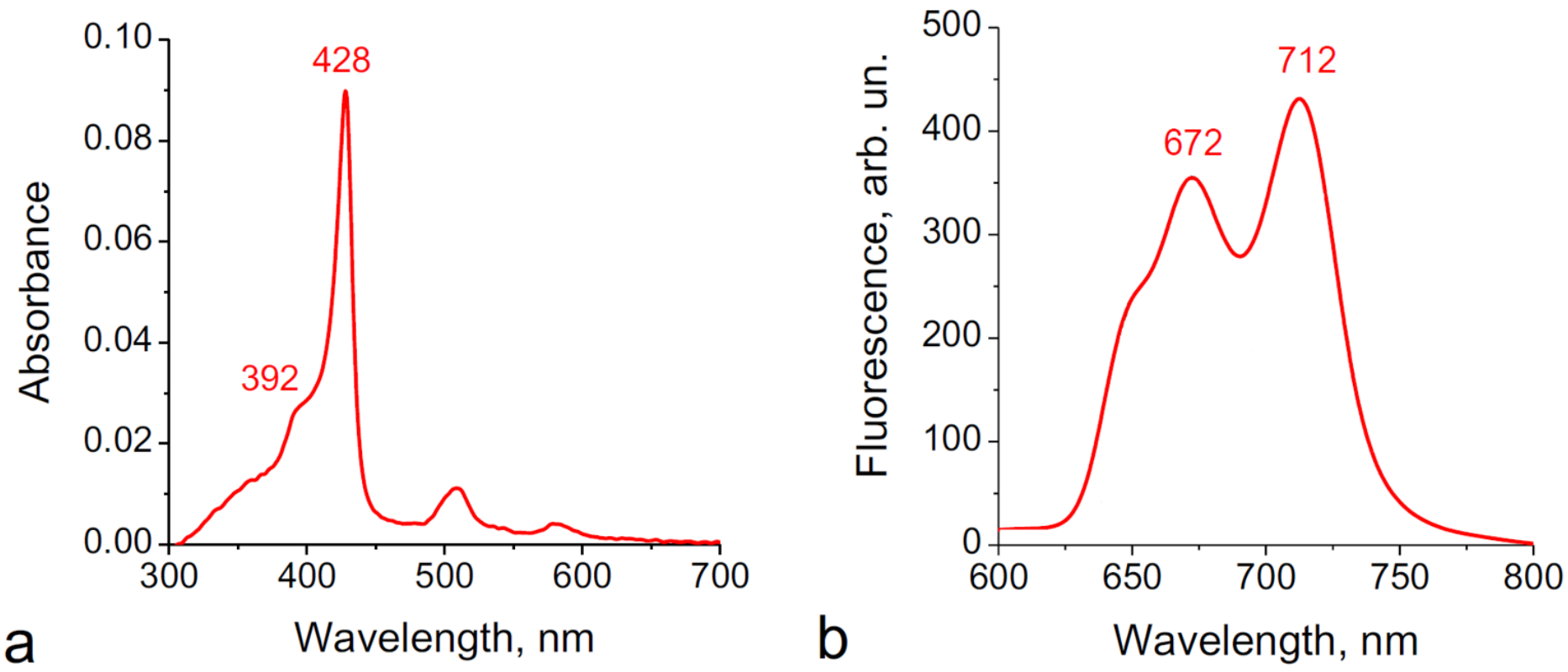

Figure S27. UV–vis (a) and fluorescence (b) spectra (λ_{ex} = 430 nm) of one-layer LS film of H_2TF_4PPP deposited onto a PVC substrate at 18 mN m^{-1}.

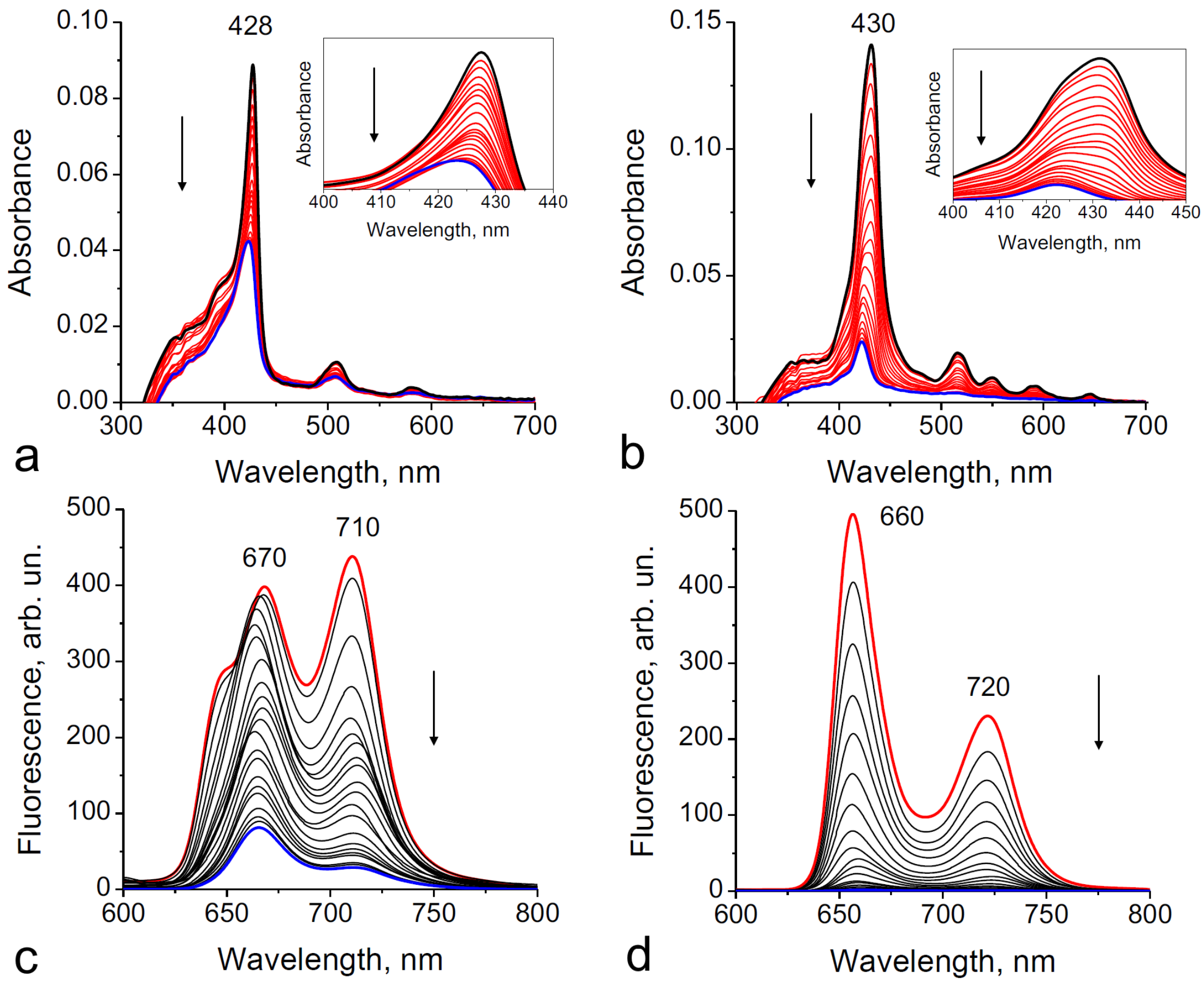

Figure S28. UV–vis (a, b) and fluorescence (c, d) spectra (λ_{ex} = 430 nm) of one-layer LS films of H_2TF_4PPP (a, c) and H_2TPPP (b, d) deposited onto a PVC substrate at 18 mN m^{-1} upon irradiation by LED (365 nm, 3 W).

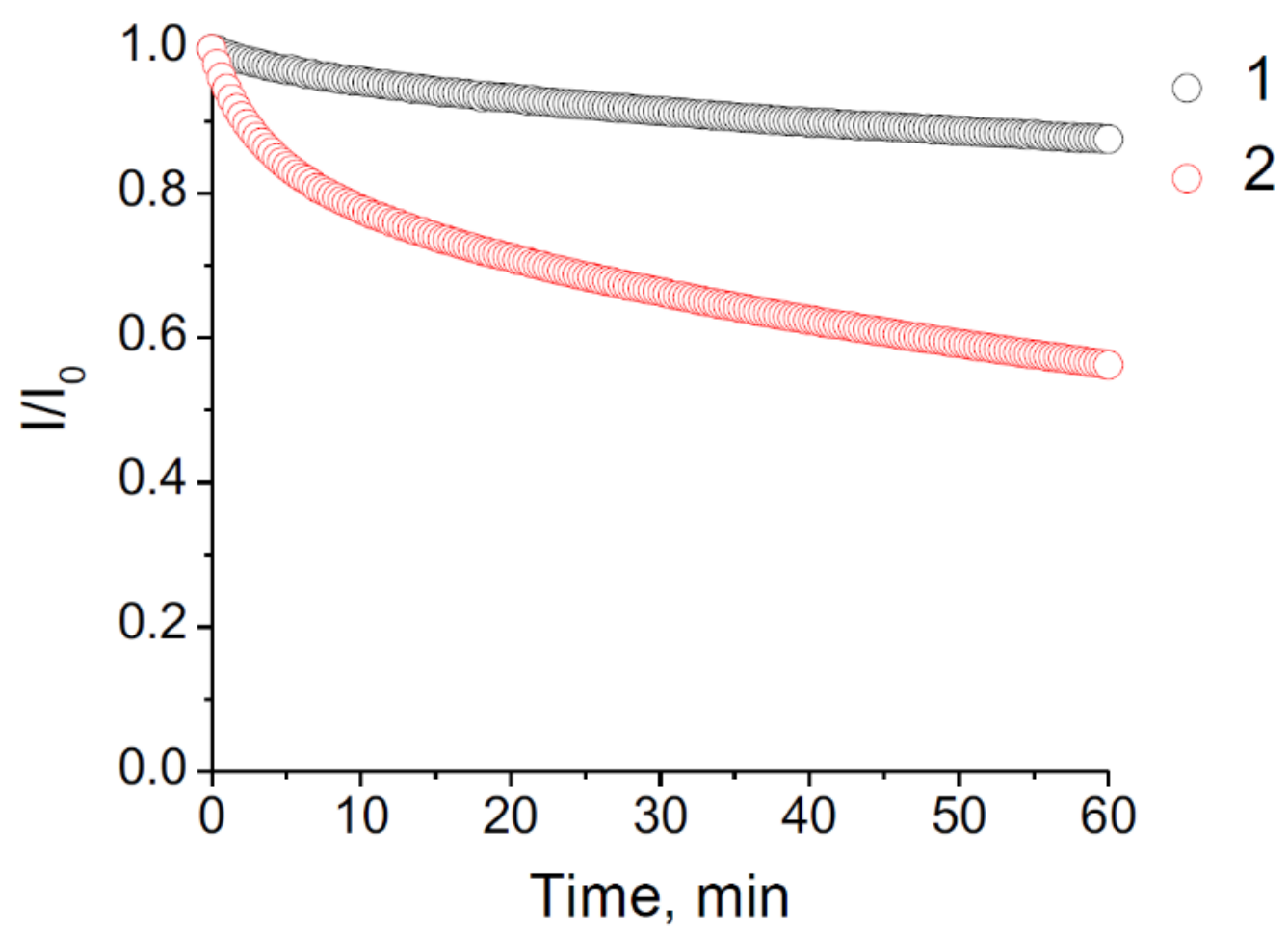

Figure S29. Photodegradation of single-layer LS films of H_2TF_4PPP (1) and H_2TPPP (2) deposited onto a PVC substrate at 18 mN m^{-1} under continuous irradiation at 430 nm during 60 min. The fluorescence intensity at the maximum of Q(0,1) band (H_2TF_4PPP (710 nm) and H_2TPPP (720 nm)) were recorded periodically.

12. Spectral data of newly synthesized compounds

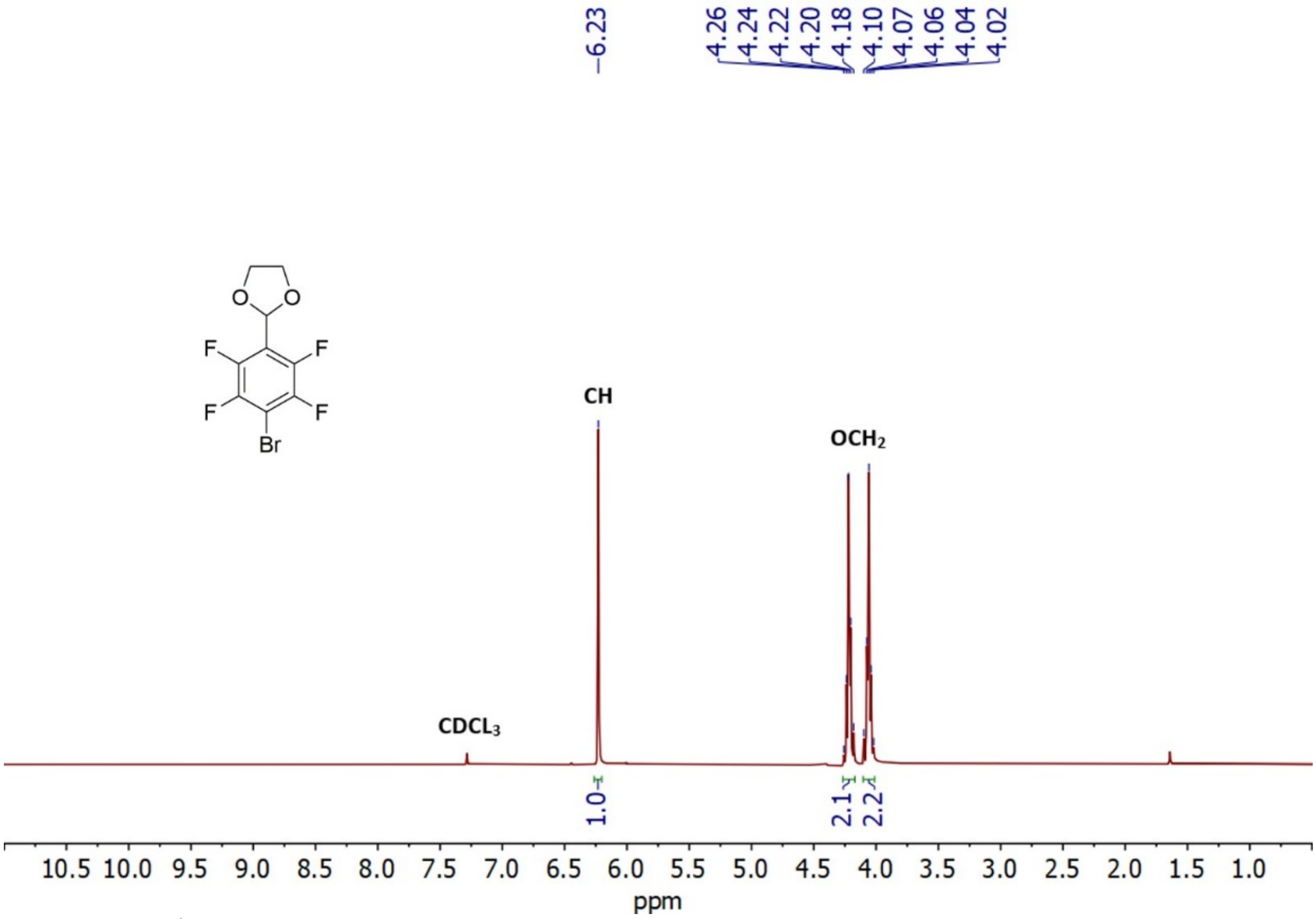

Figure S30. ^{1}H NMR spectrum of compound **2** (300 MHz, $CDCl_3$, 298 K).

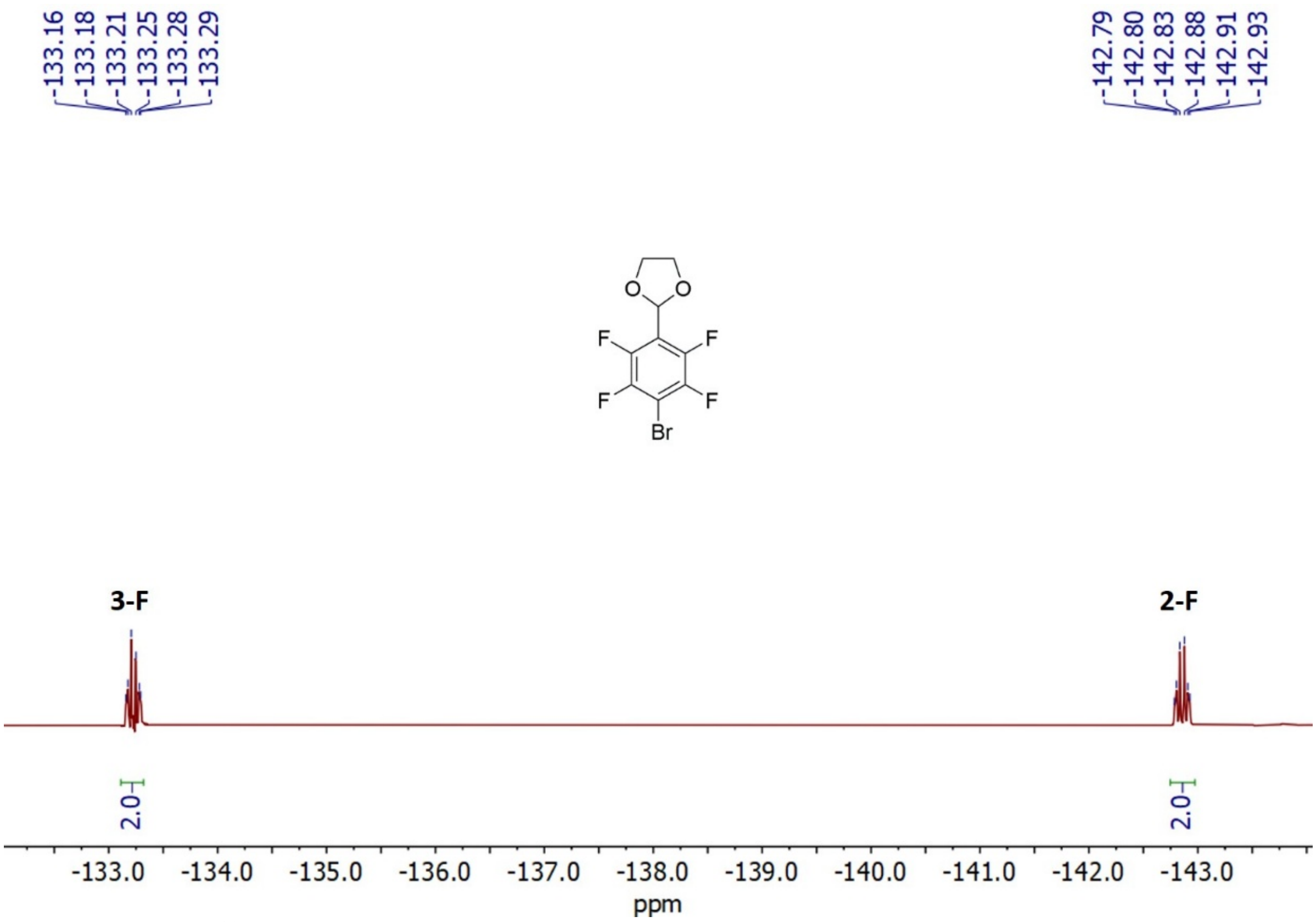

Figure S31. ^{19}F NMR spectrum of compound **2** (282 MHz, $CDCl_3$, 298 K).

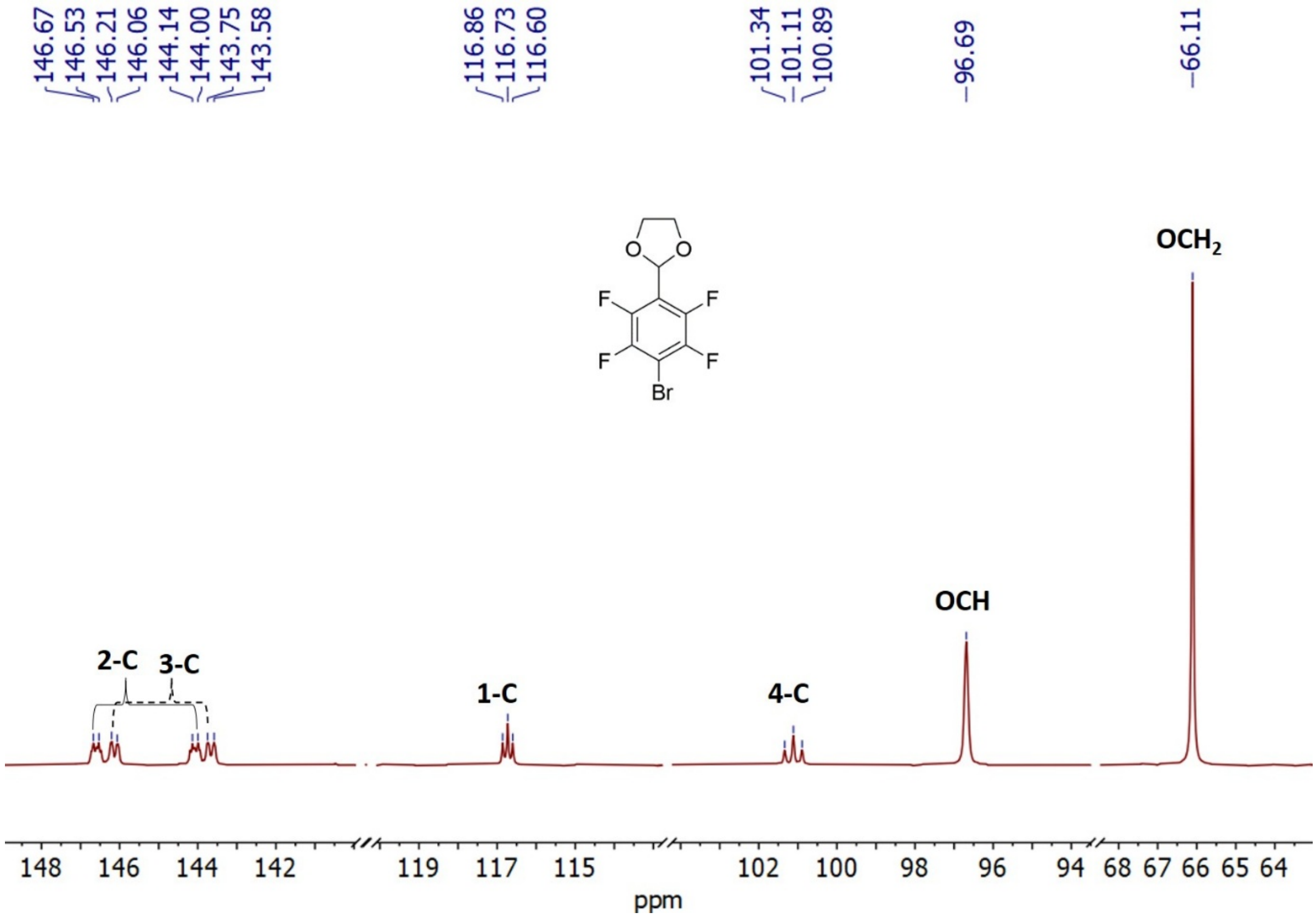

Figure S32. $^{13}C\{^{1}H\}$ NMR spectrum of compound **2** (75 MHz, $CDCl_3$, 298 K).

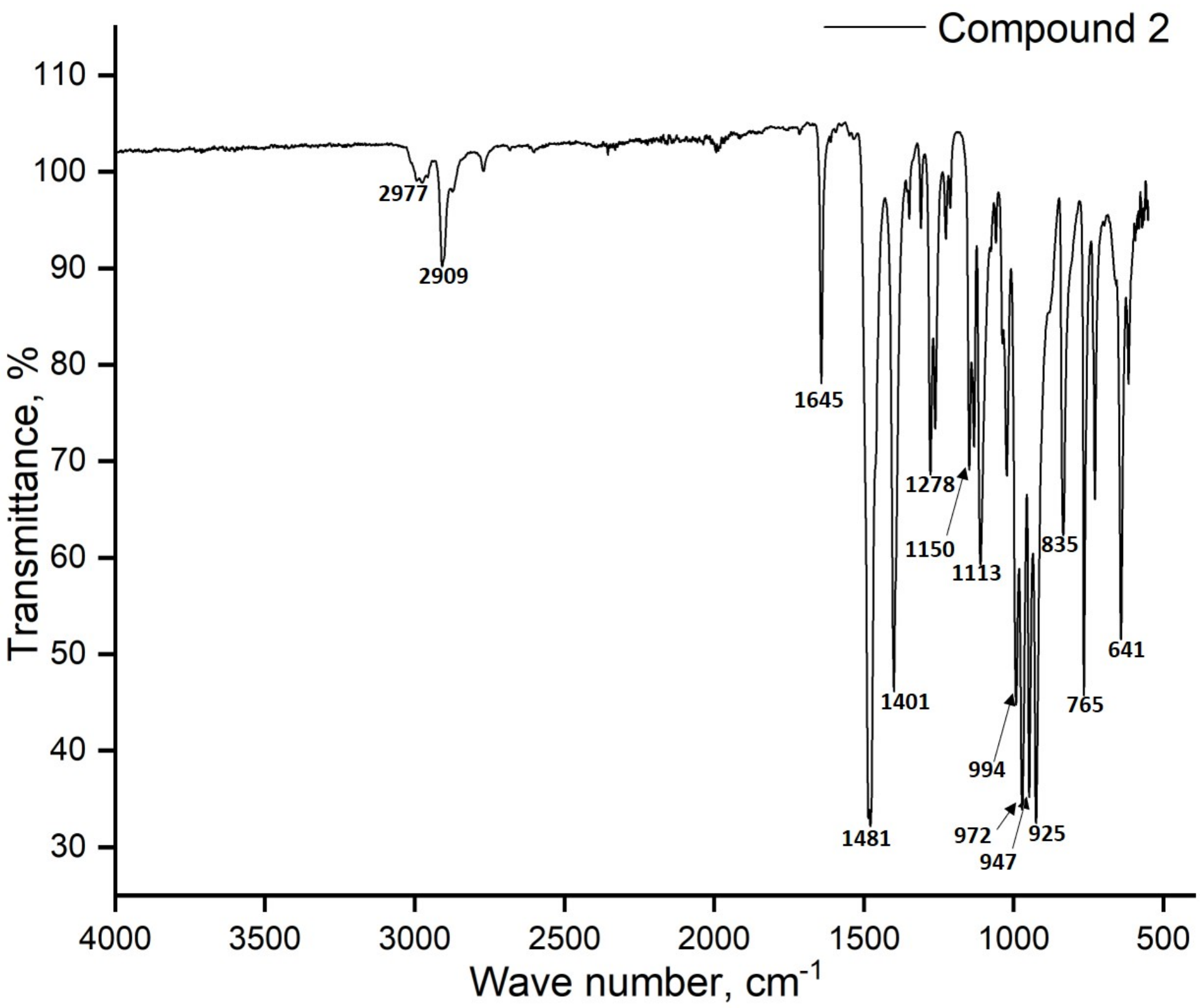

Figure S33. FT-IR spectrum of compound **2** (neat).

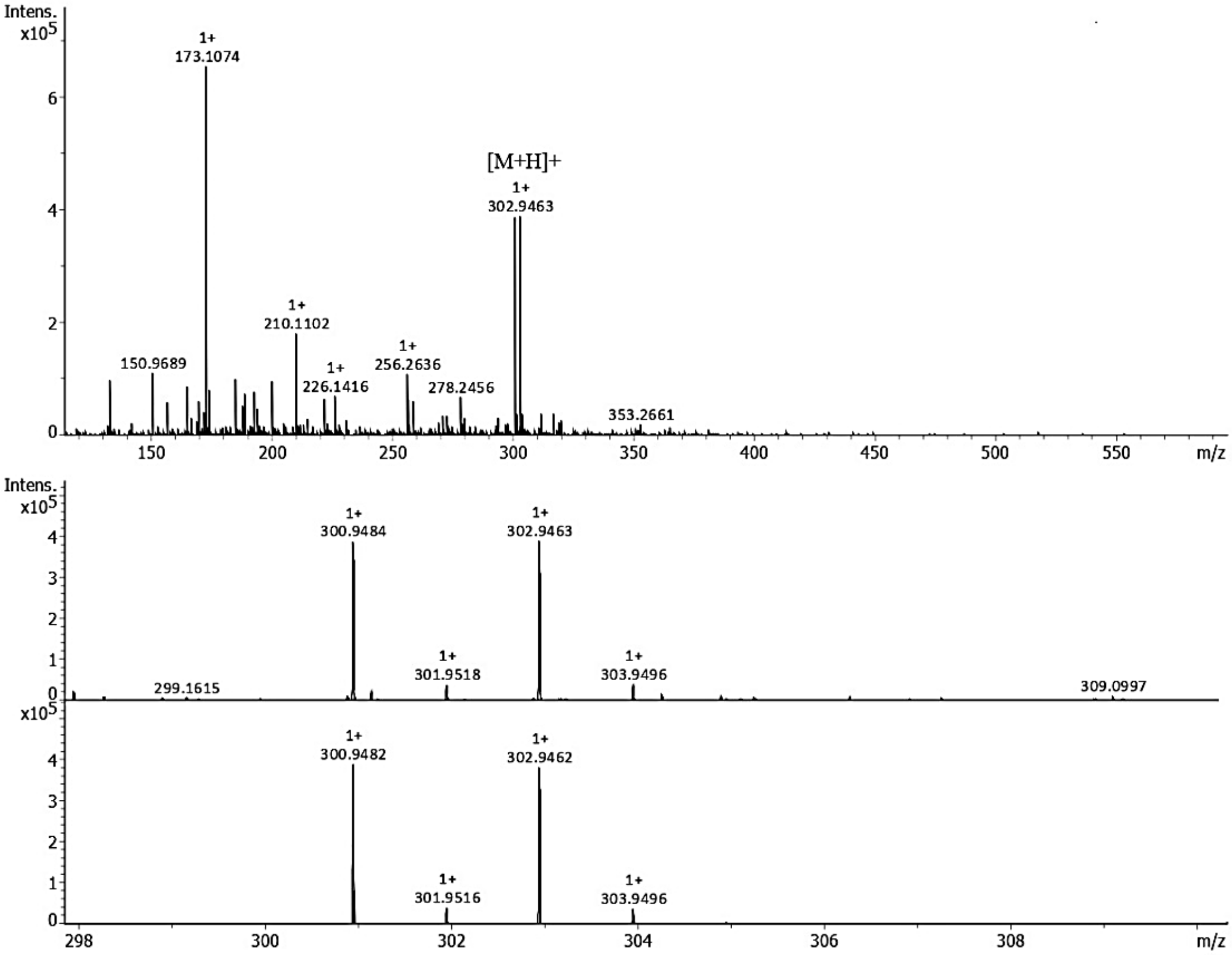

Meas. m/z	Ion Formula	m/z	Sum Formula	err [ppm]	mSigma	Adduct	z
300.9484	C9H6BrF4O2	300.9482	C9H5BrF4O2	-0.6	9.5	M+H	1+

Figure S34. HRMS (ESI^+) spectrum of compound **2**.

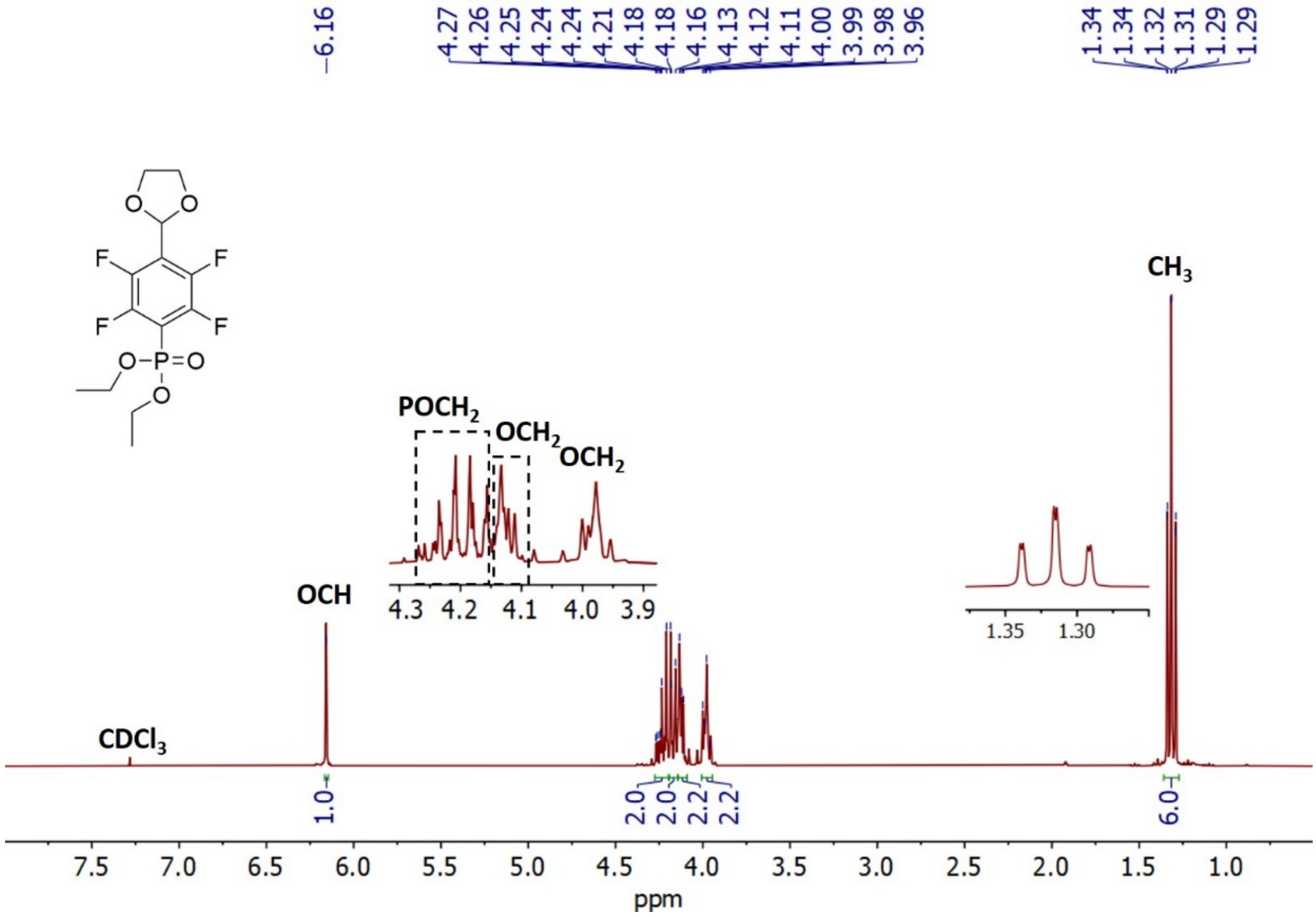

Figure S35. ^{1}H NMR spectrum of compound **3** (300 MHz, $CDCl_3$, 298 K).

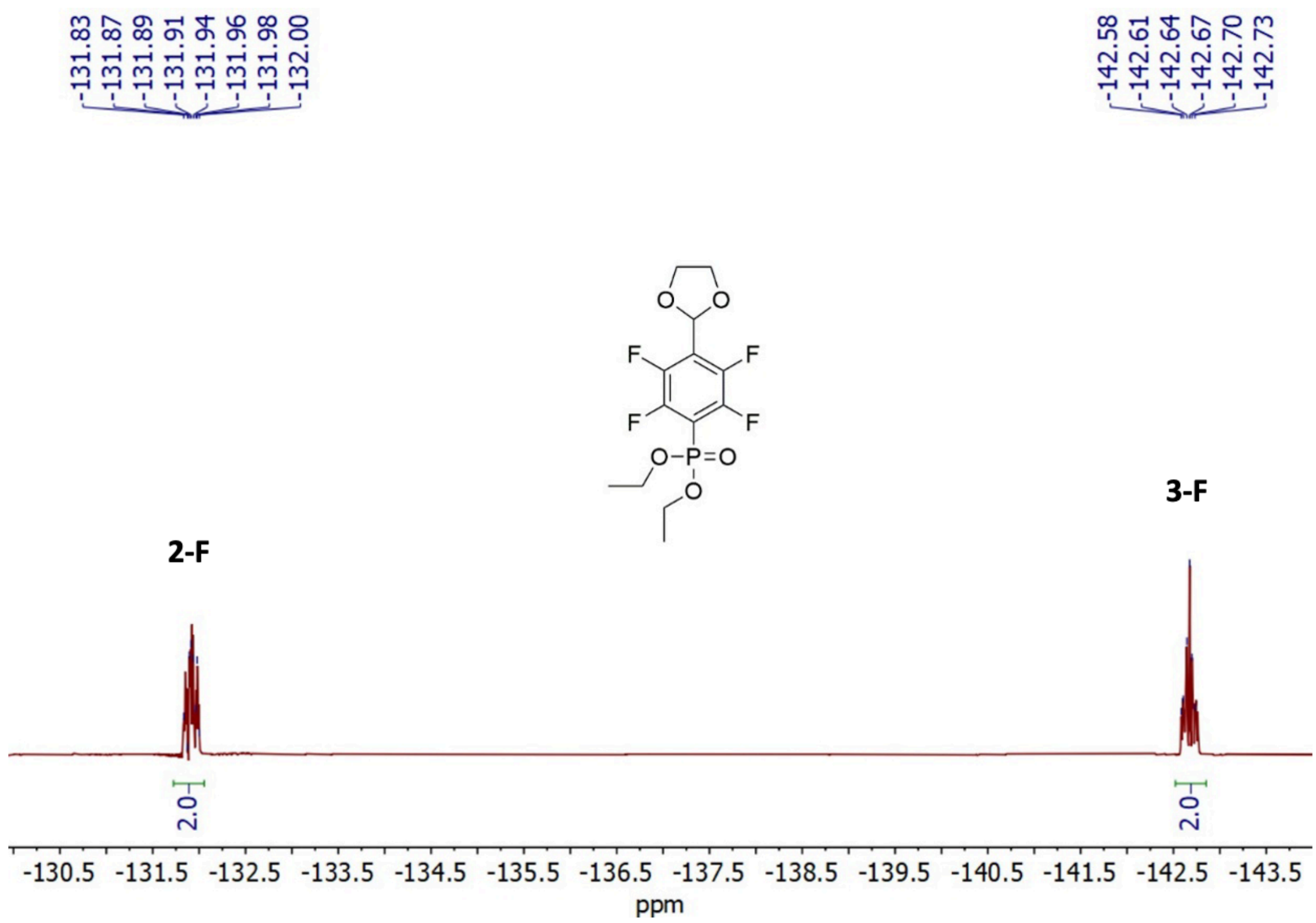

Figure S36. ^{19}F NMR spectrum of compound **3** (282 MHz, $CDCl_3$, 298 K).

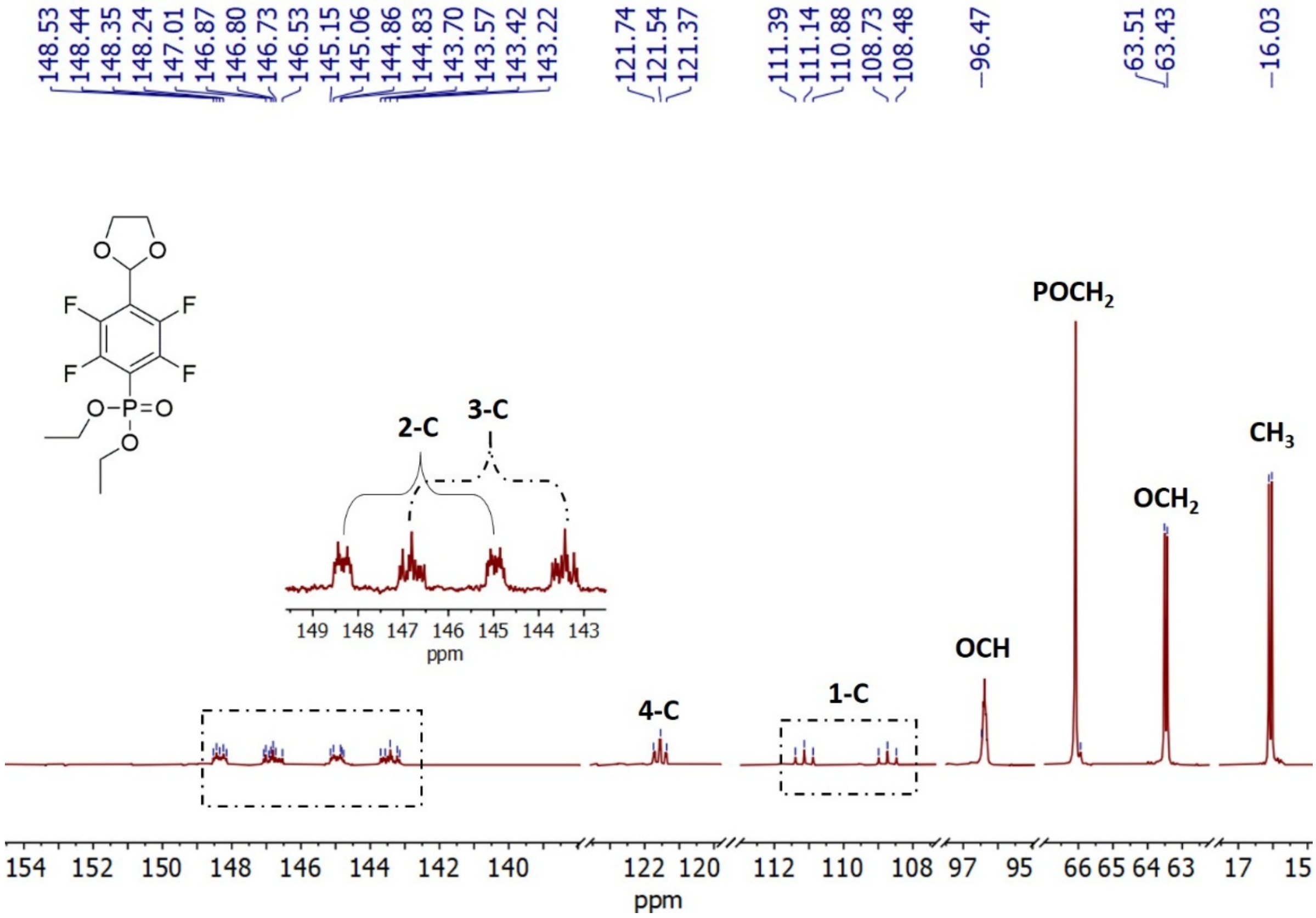

Figure S37. $^{13}C\{^{1}H\}$ NMR spectrum of compound **3** (75 MHz, $CDCl_3$, 298 K).

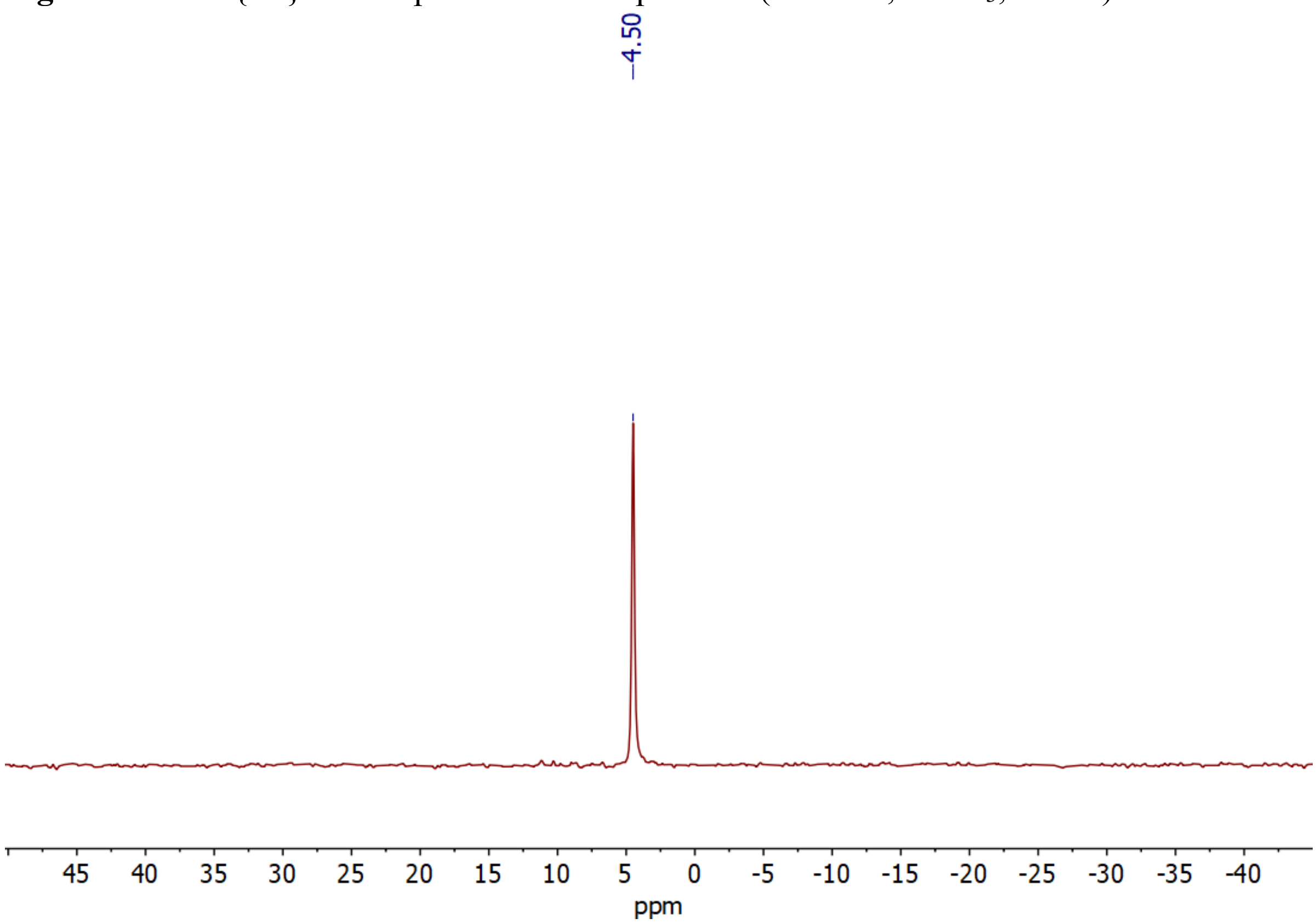

Figure S38. $^{31}P\{^{1}H\}$ NMR spectrum of compound **3** (121 MHz, $CDCl_3$, 298 K).

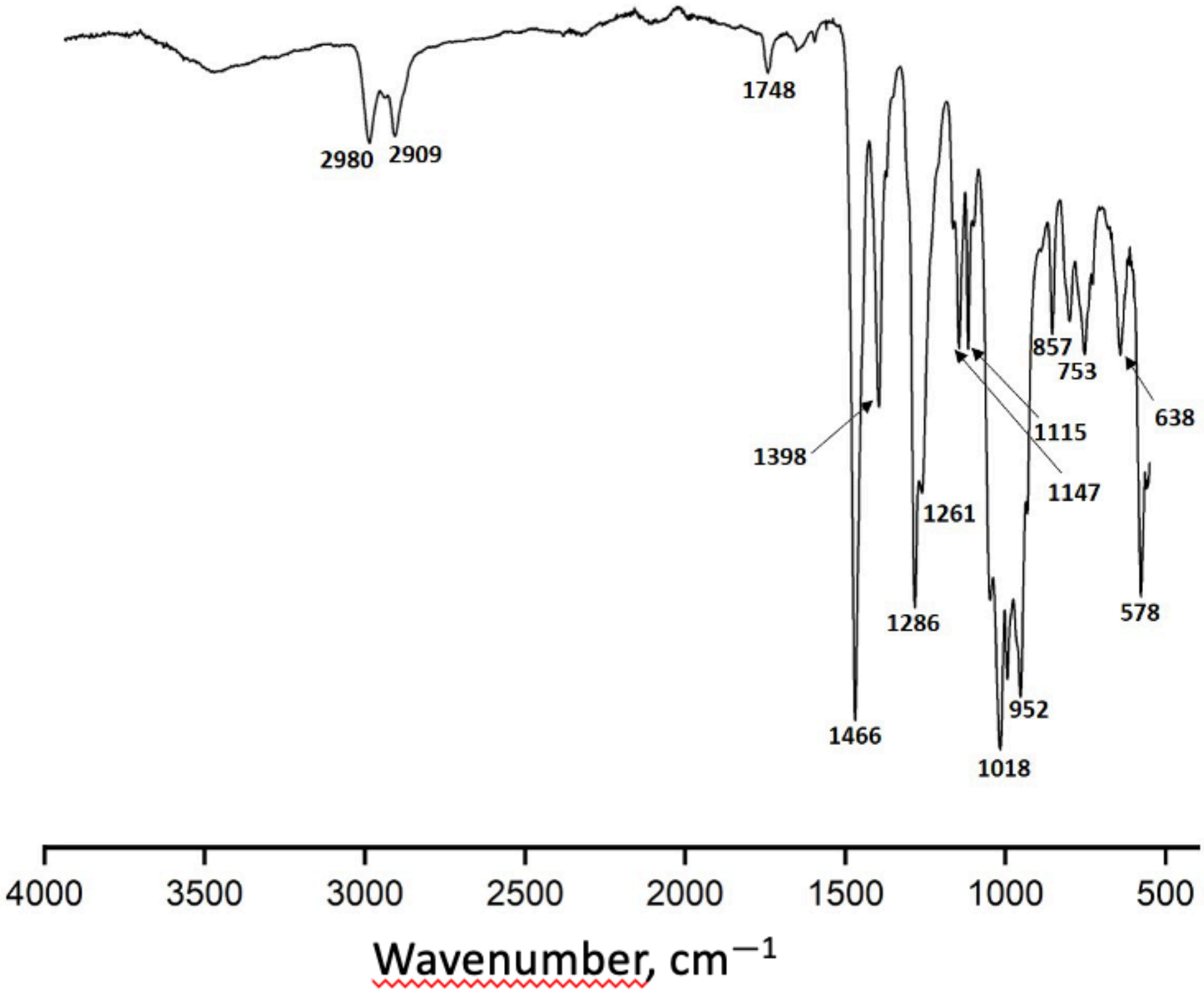

Figure S39. FT-IR spectrum of Compound **3** (neat).

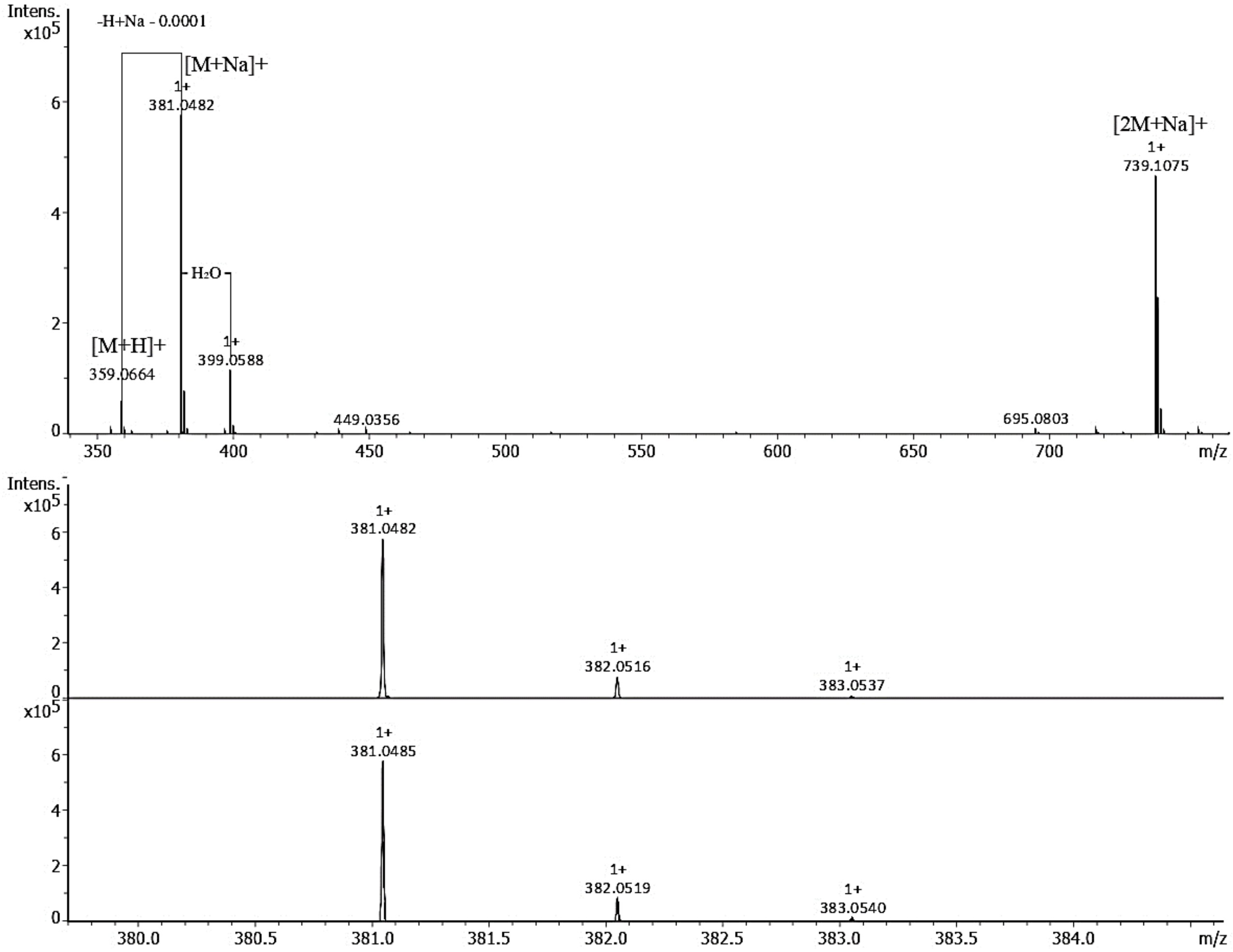

Meas. m/z	Ion Formula	m/z	Sum Formula	err [ppm]	mSigma	Adduct	z
359.0664	C13H16F4O5P	359.0666	C13H15F4O5P	0.6	2.2	M+H	1+
381.0482	C13H15F4NaO5P	381.0485		0.8	4.9	M+Na	1+
739.1075	C26H30F8NaO10P2	739.1079		0.5	124.1	2M+Na	1+

Figure S40. HRMS (ESI^+) spectrum of compound **3**.

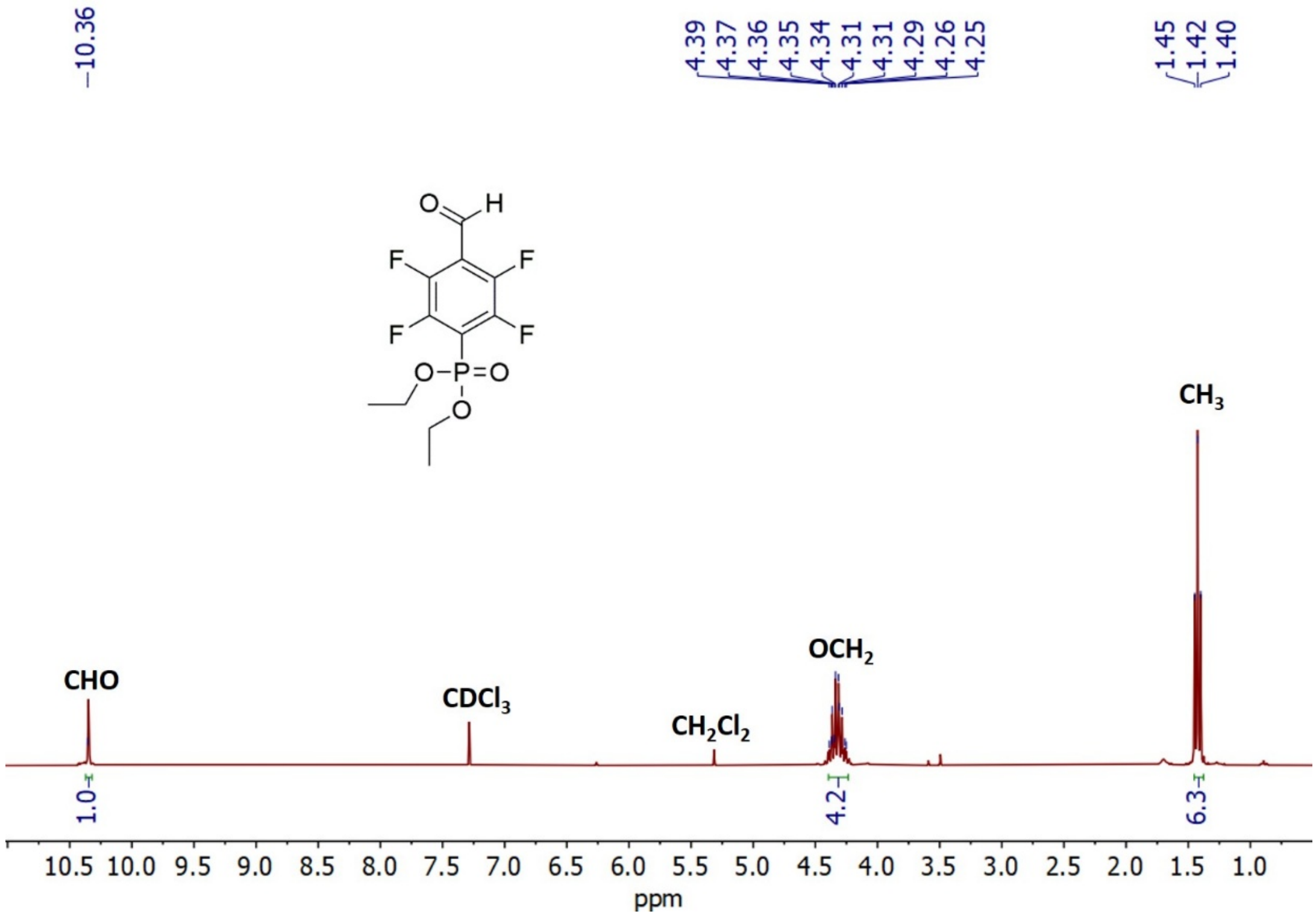

Figure S41. ^{1}H NMR spectrum of compound **6** (300 MHz, $CDCl_3$, 298 K).

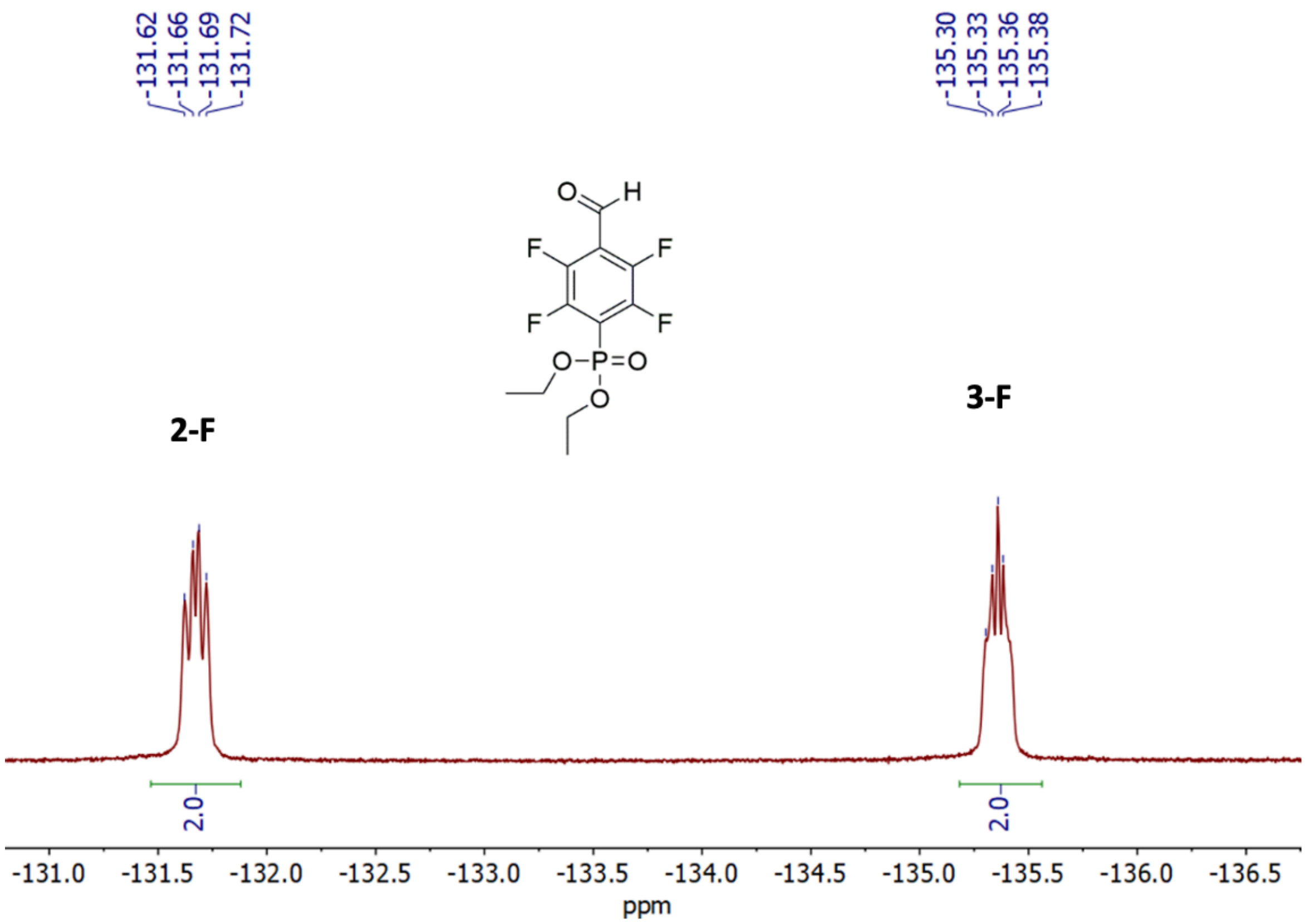

Figure S42. ^{19}F NMR spectrum of compound **6** (282 MHz, $CDCl_3$, 298 K).

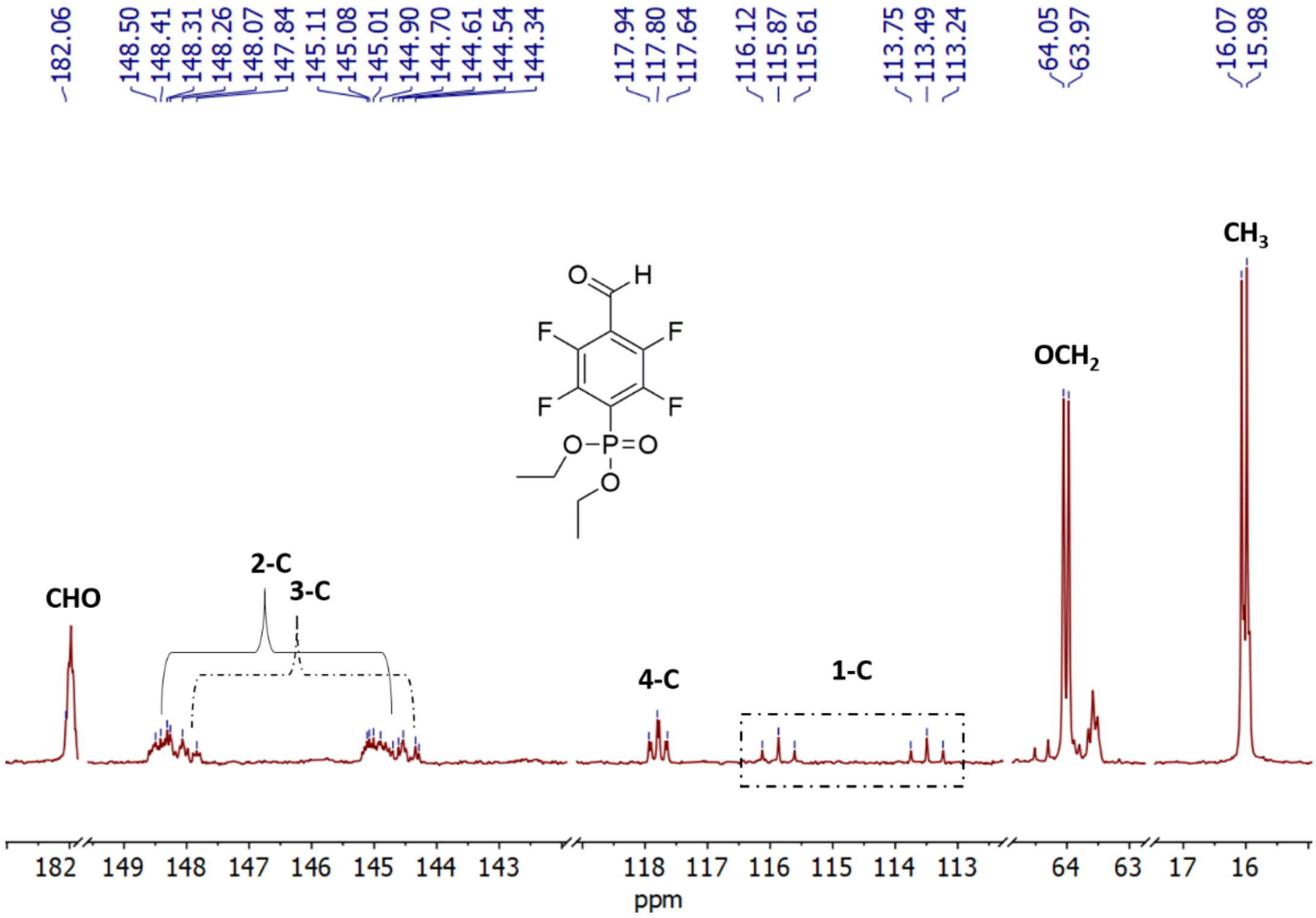

Figure S43. $^{13}C\{^1H\}$ NMR spectrum of compound **6** (75 MHz, $CDCl_3$, 298 K).

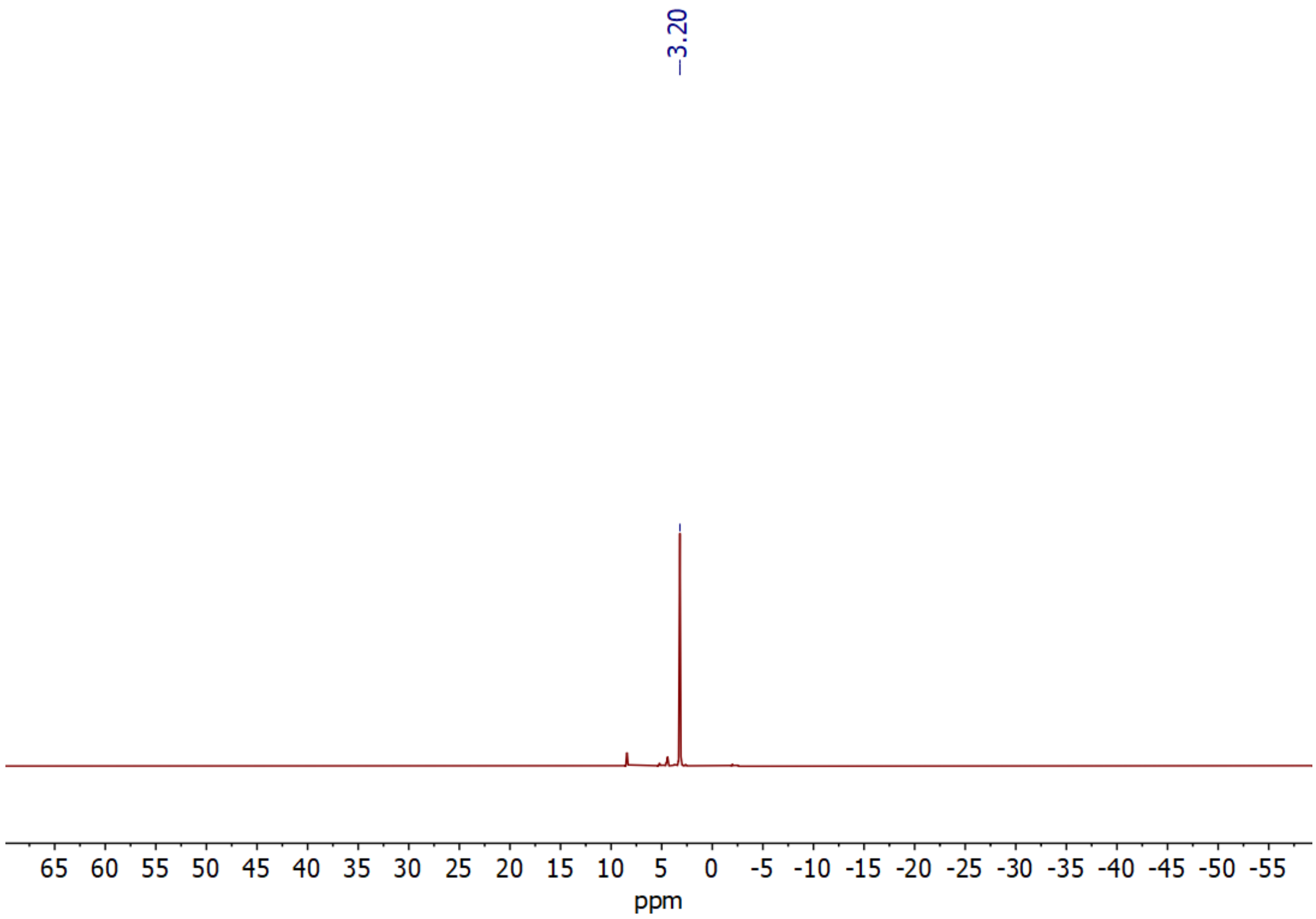

Figure S44. $^{31}P\{^1H\}$ NMR spectrum of compound **6** (121 MHz, $CDCl_3$, 298 K).

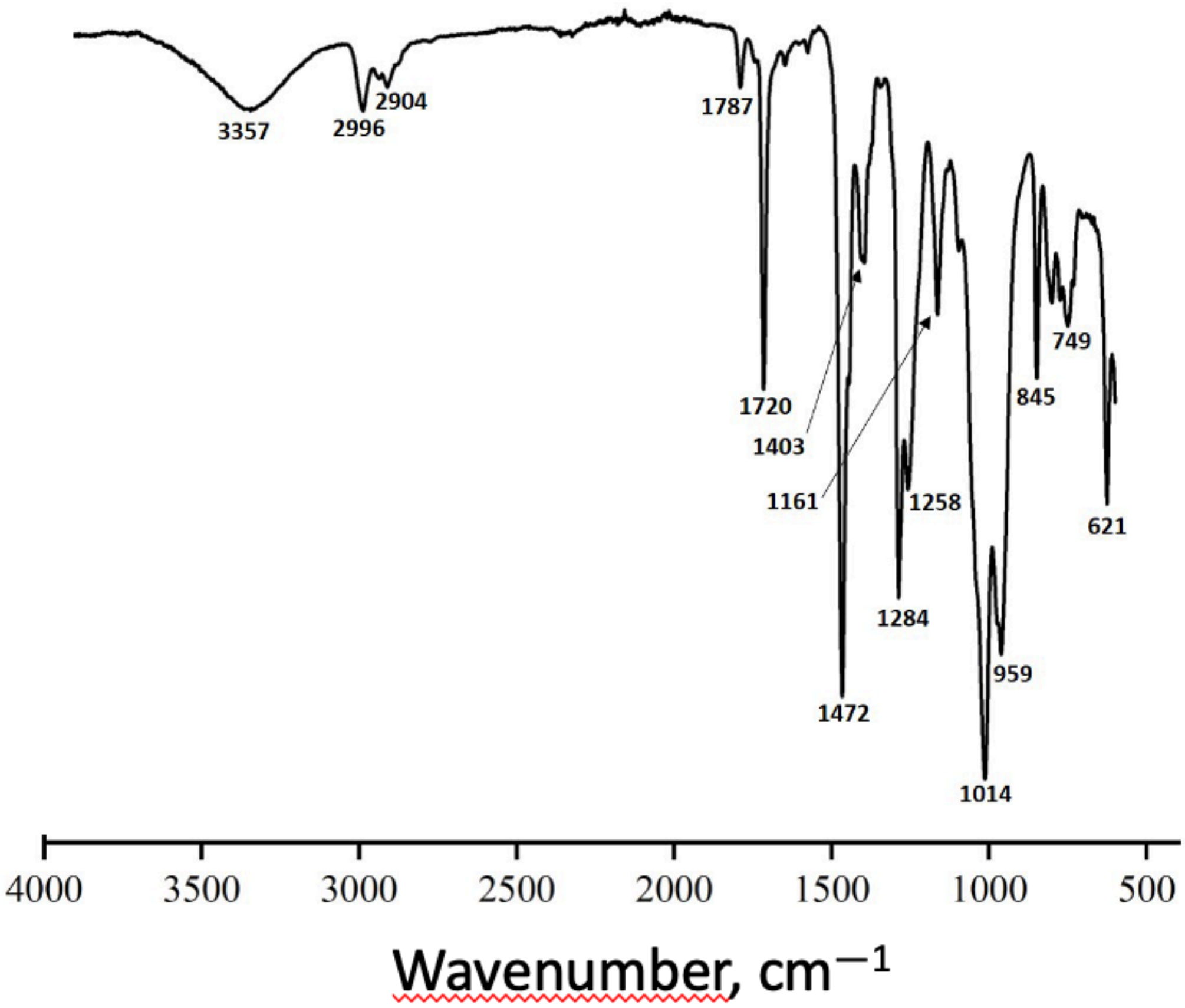

Figure S45. FT-IR spectrum of compound **6** (neat).

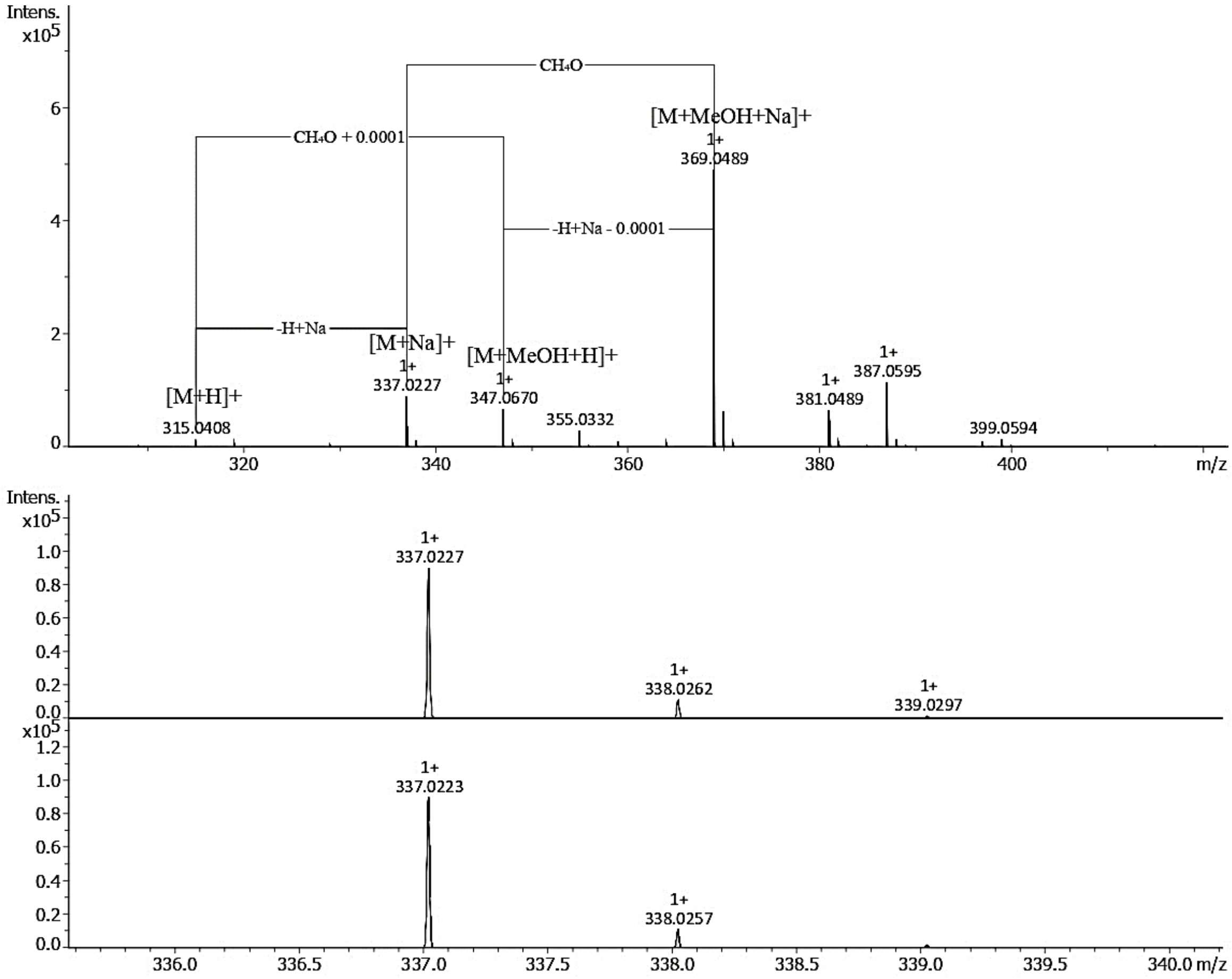

Meas. m/z	Ion Formula	m/z	Sum Formula	err [ppm]	mSigma	Adduct	z
315.0408	C11H12F4O4P	315.0404	C11H11F4O4P	-1.2	10.7	M+H	1+
337.0227	C11H11F4NaO4P	337.0223		-1.1	3.4	M+Na	1+
347.0670	C12H16F4O5P	347.0666	C12H15F4O5P	-1.3	3.4	M+H	1+
369.0489	C12H15F4NaO5P	369.0485		-1.0	1.2	M+Na	1+

Figure S46. HRMS (ESI^+) spectrum of compound **6**.

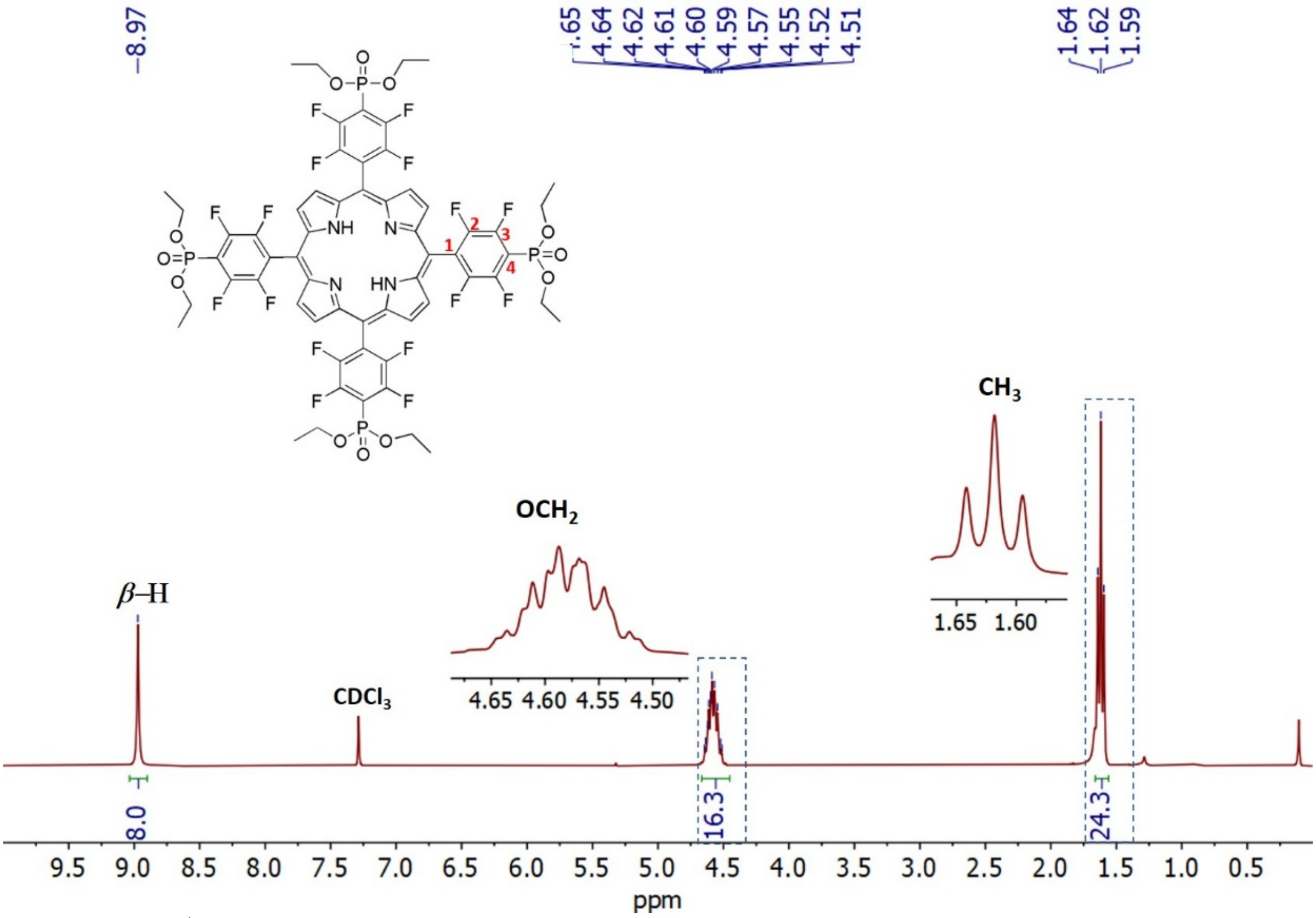

Figure S47. ^{1}H NMR spectrum of H_2TF_4PPP (300 MHz, $CDCl_3$, 298 K).

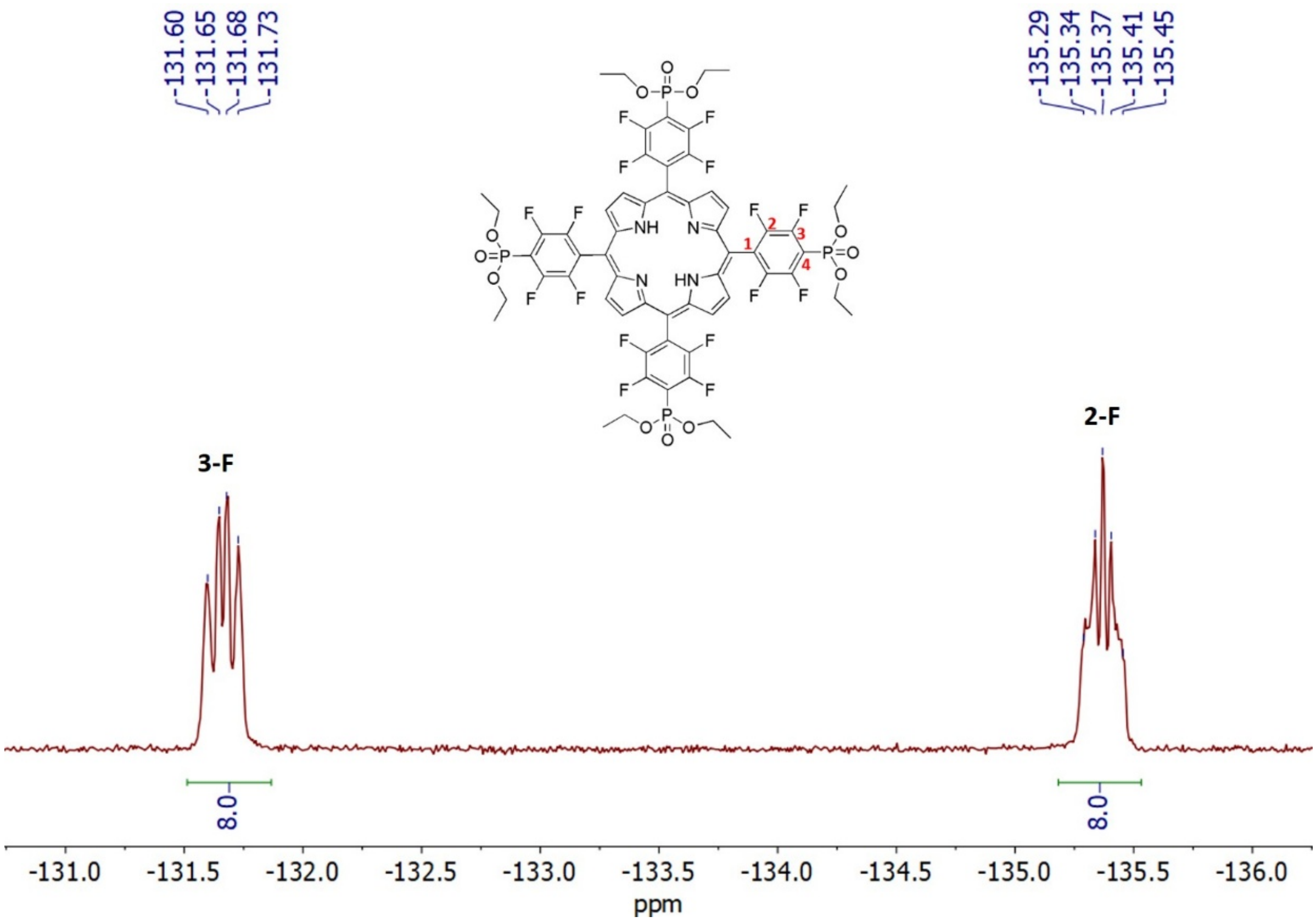

Figure S48. ^{19}F NMR spectrum of H_2TF_4PPP (282 MHz, $CDCl_3$, 298 K).

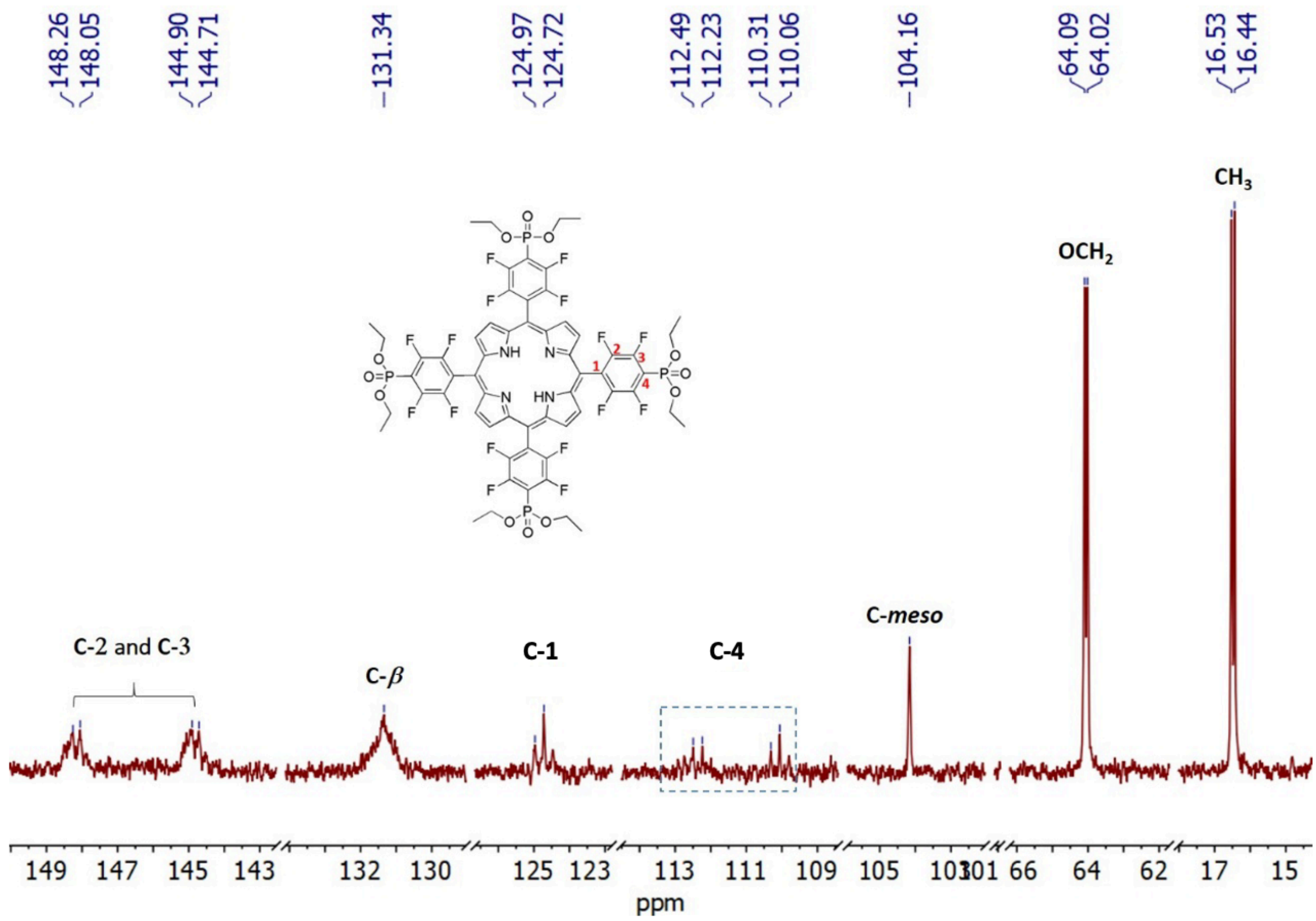

Figure S49. $^{13}C\{^{1}H\}$ NMR spectrum of H_2TF_4PPP (75 MHz, $CDCl_3$, 298 K).

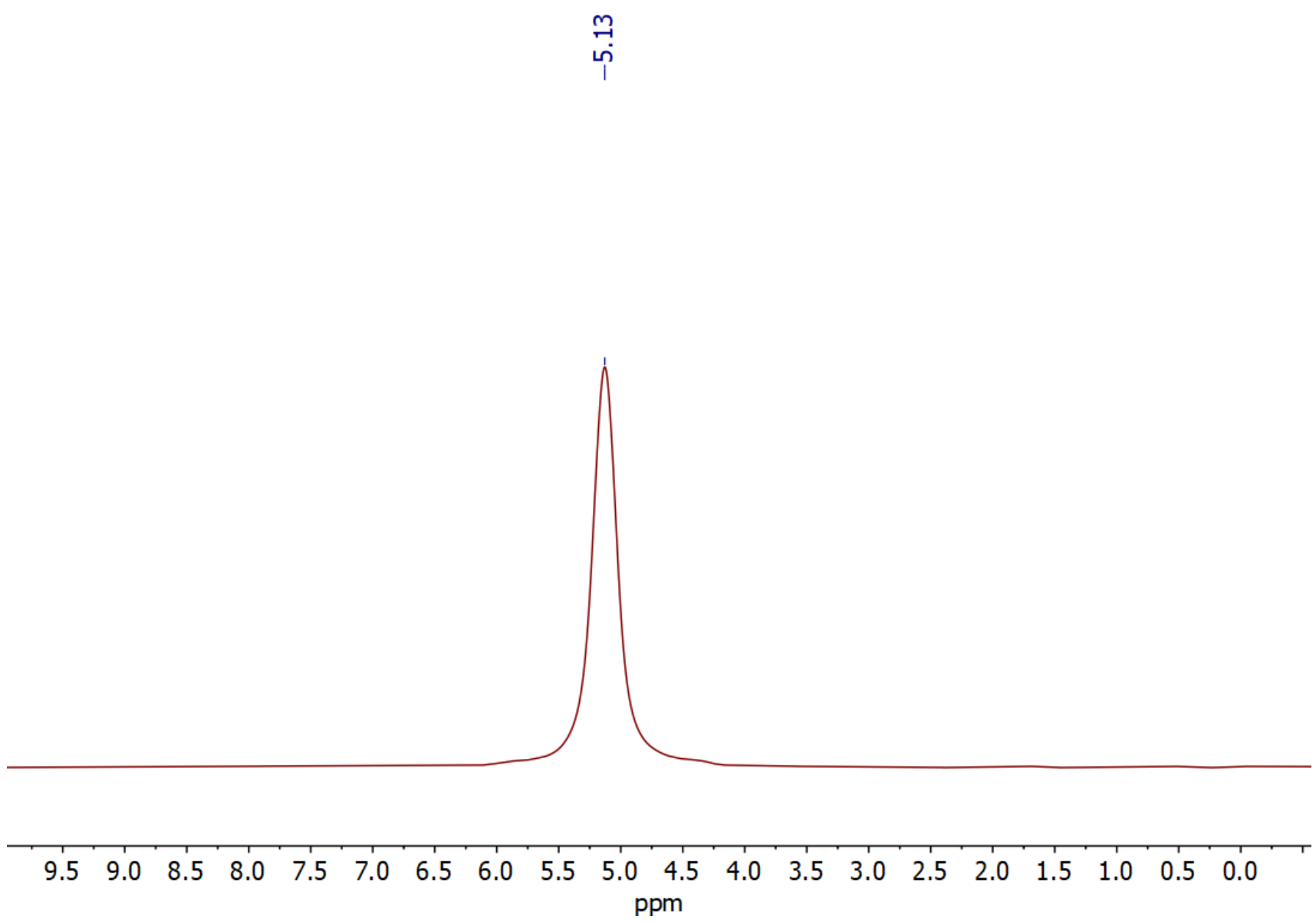

Figure S50. $^{31}P\{^{1}H\}$ NMR spectrum of H_2TF_4PPP (121 MHz, $CDCl_3$, 298 K).

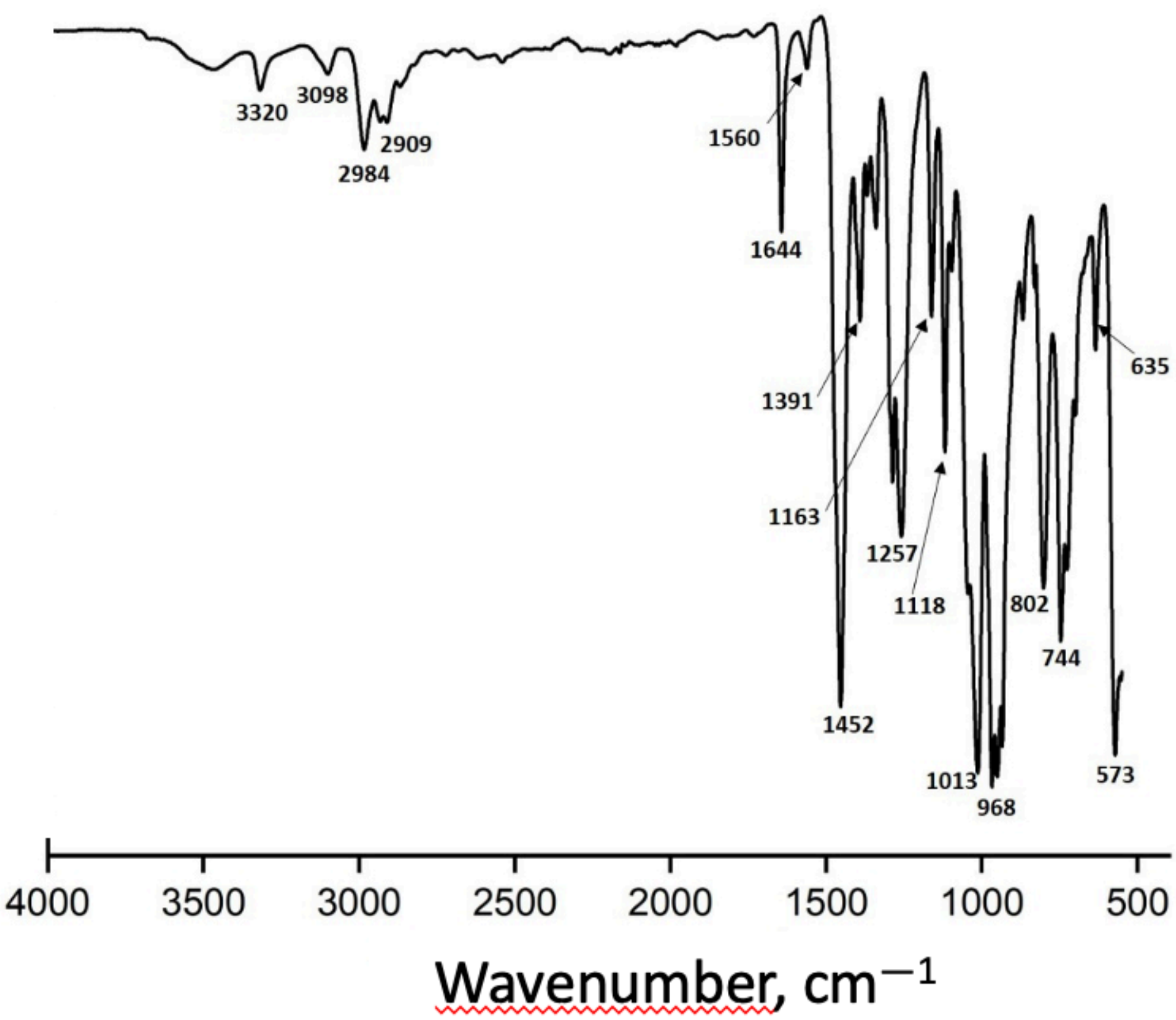

Figure S51. FT-IR spectrum of H_2TF_4PPP (neat).

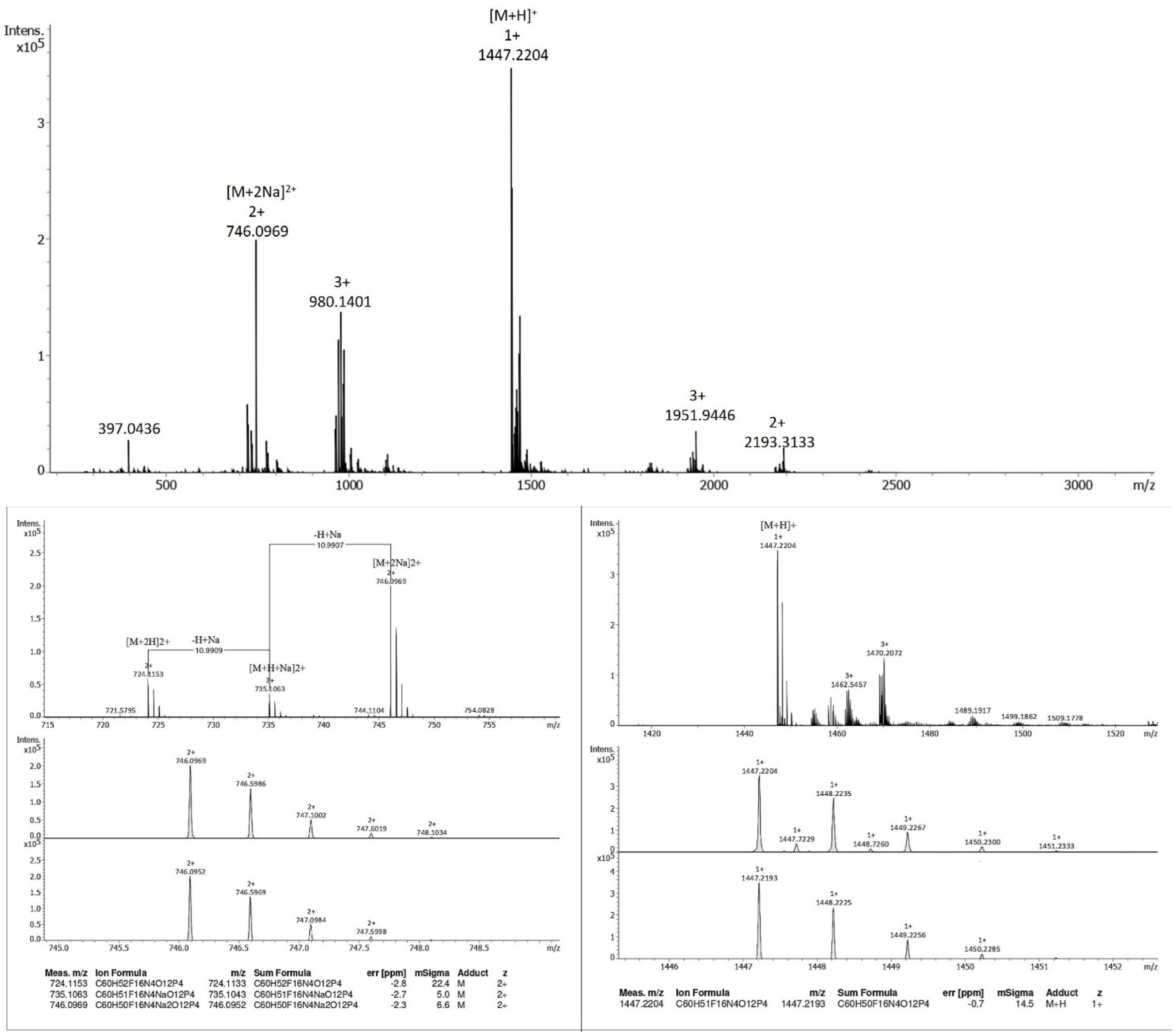

Figure S52. HRMS (ESI^+) spectrum of H_2TF_4PPP.

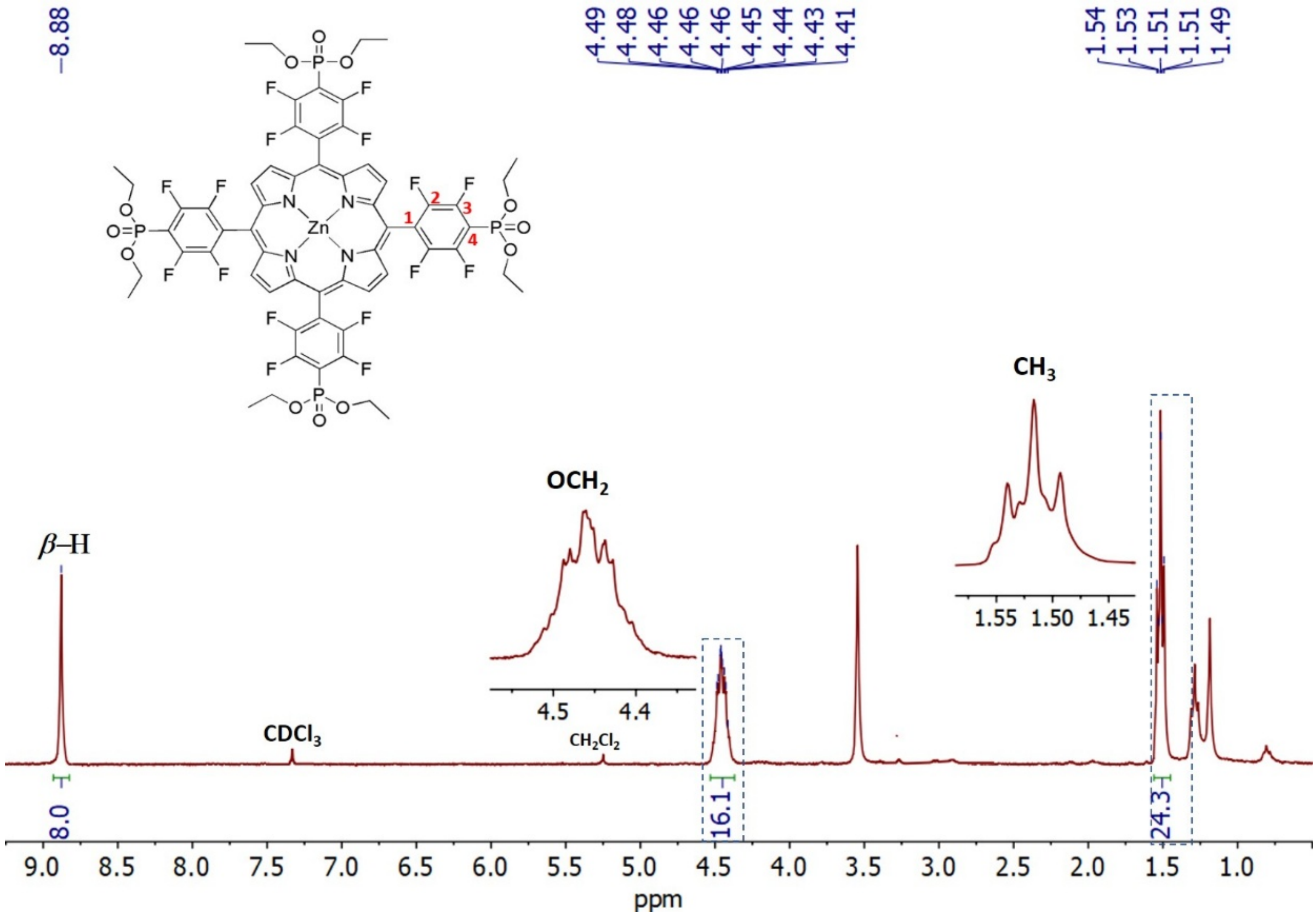

Figure 53. ^{1}H NMR spectrum of ZnTF$_4$PPP (300 MHz, $CDCl_3$, 298 K).

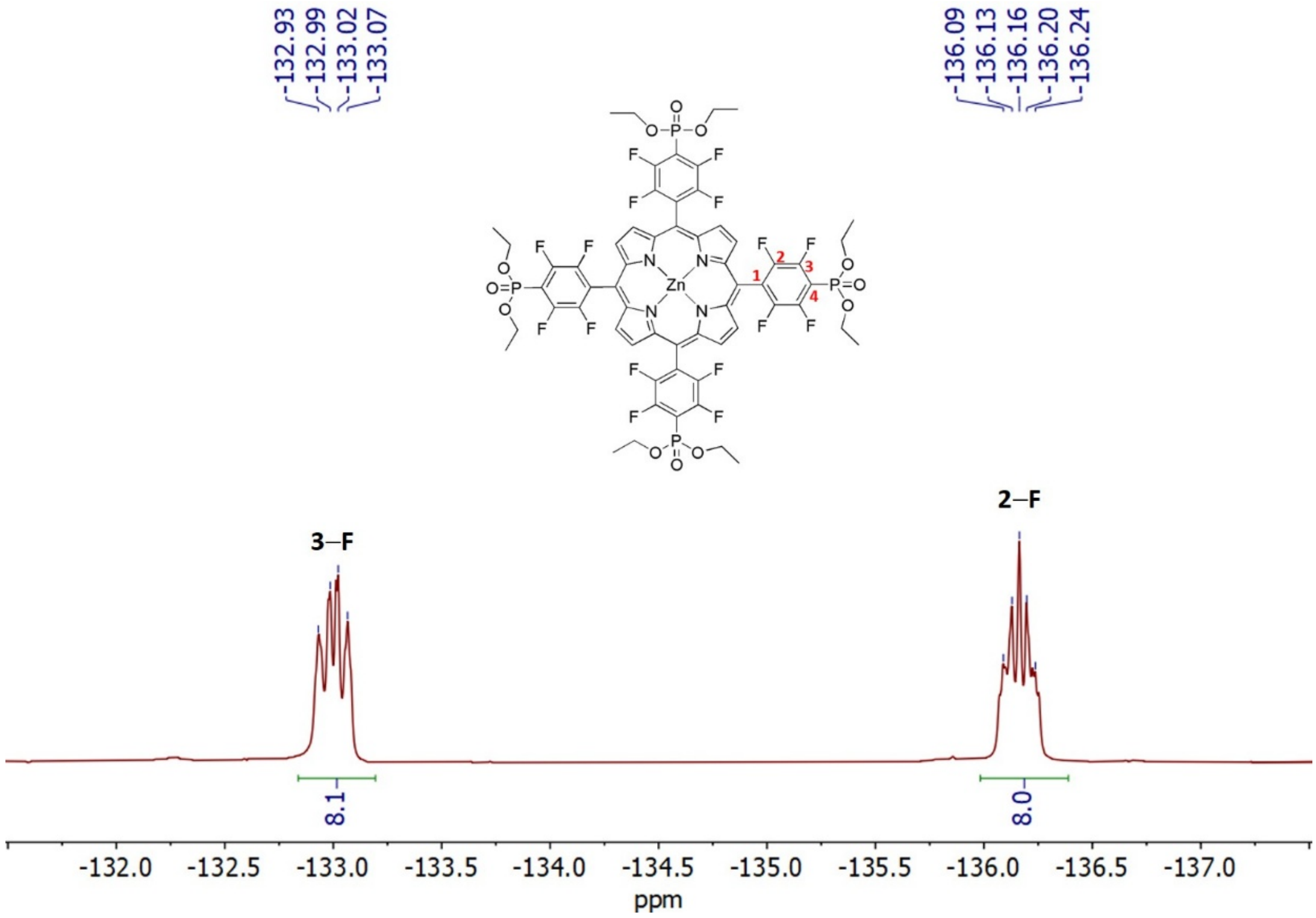

Figure S54. ^{19}F NMR spectrum of ZnTF$_4$PPP (282 MHz, $CDCl_3$, 298 K).

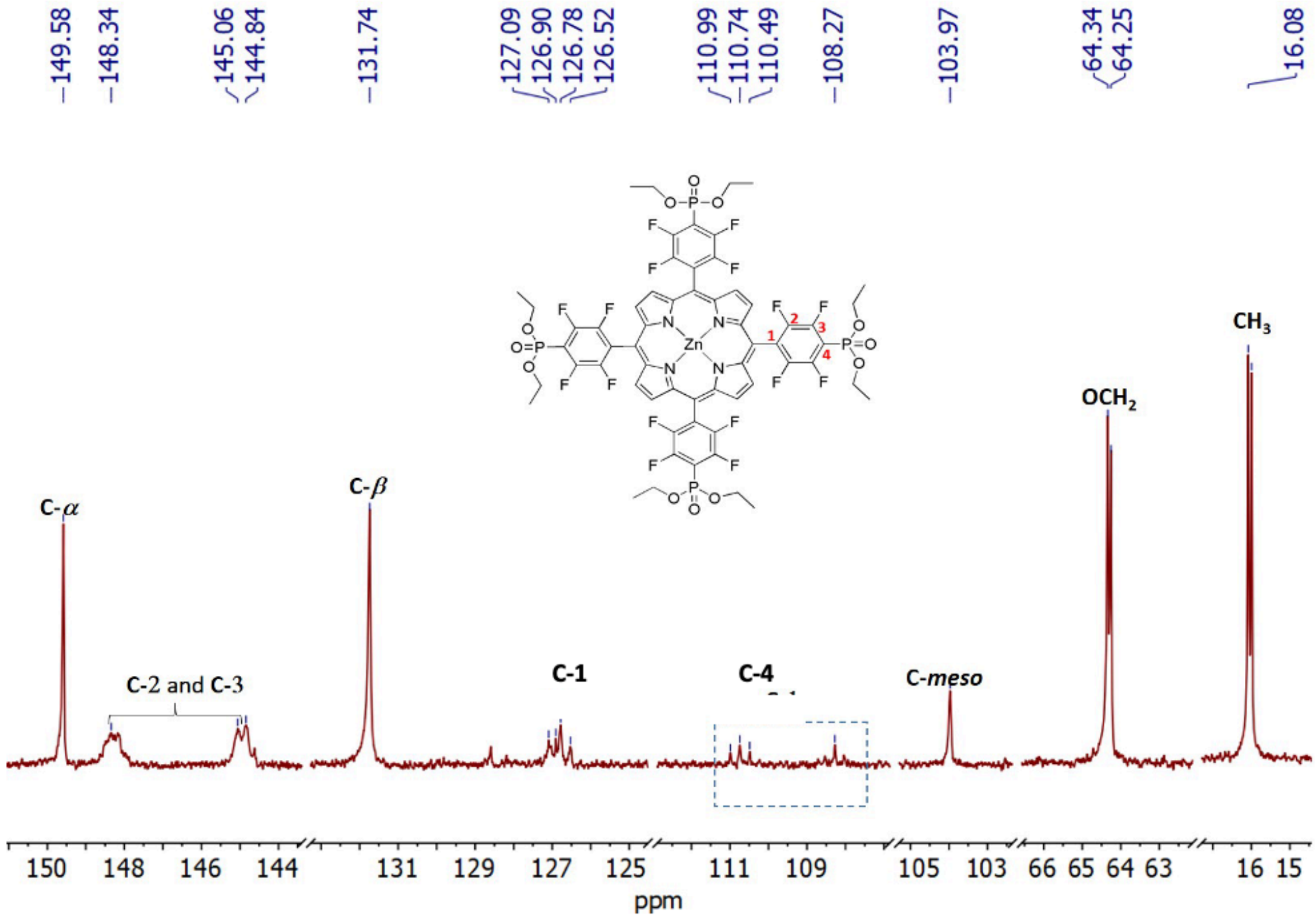

Figure S55. $^{13}C\{^{1}H\}$ NMR spectrum of $ZnTF_4PPP$ (75 MHz, $CDCl_3$, 298 K).

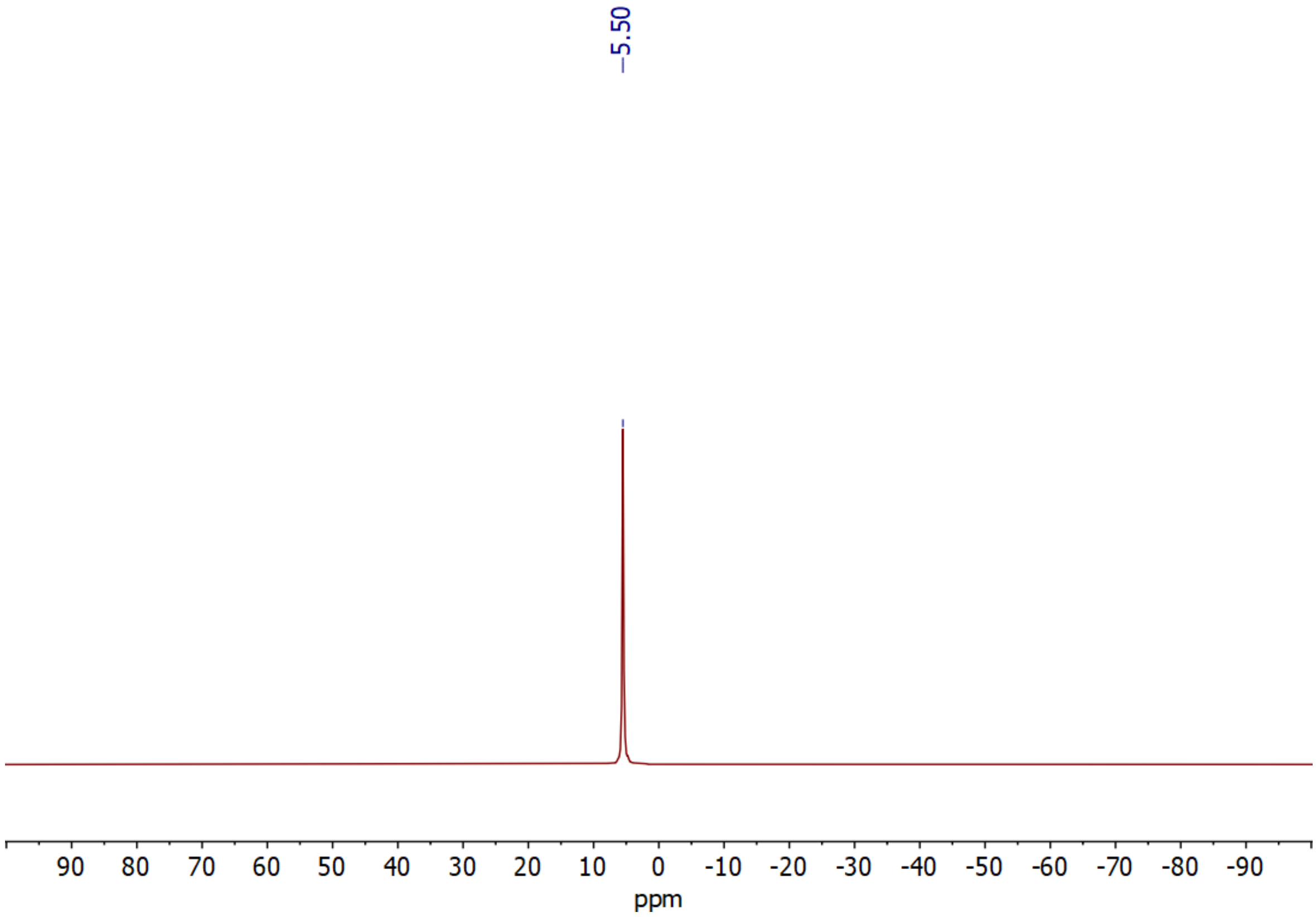

Figure S56. $^{31}P\{^{1}H\}$ NMR spectrum of $ZnTF_4PPP$ (121 MHz, $CDCl_3$, 298 K).

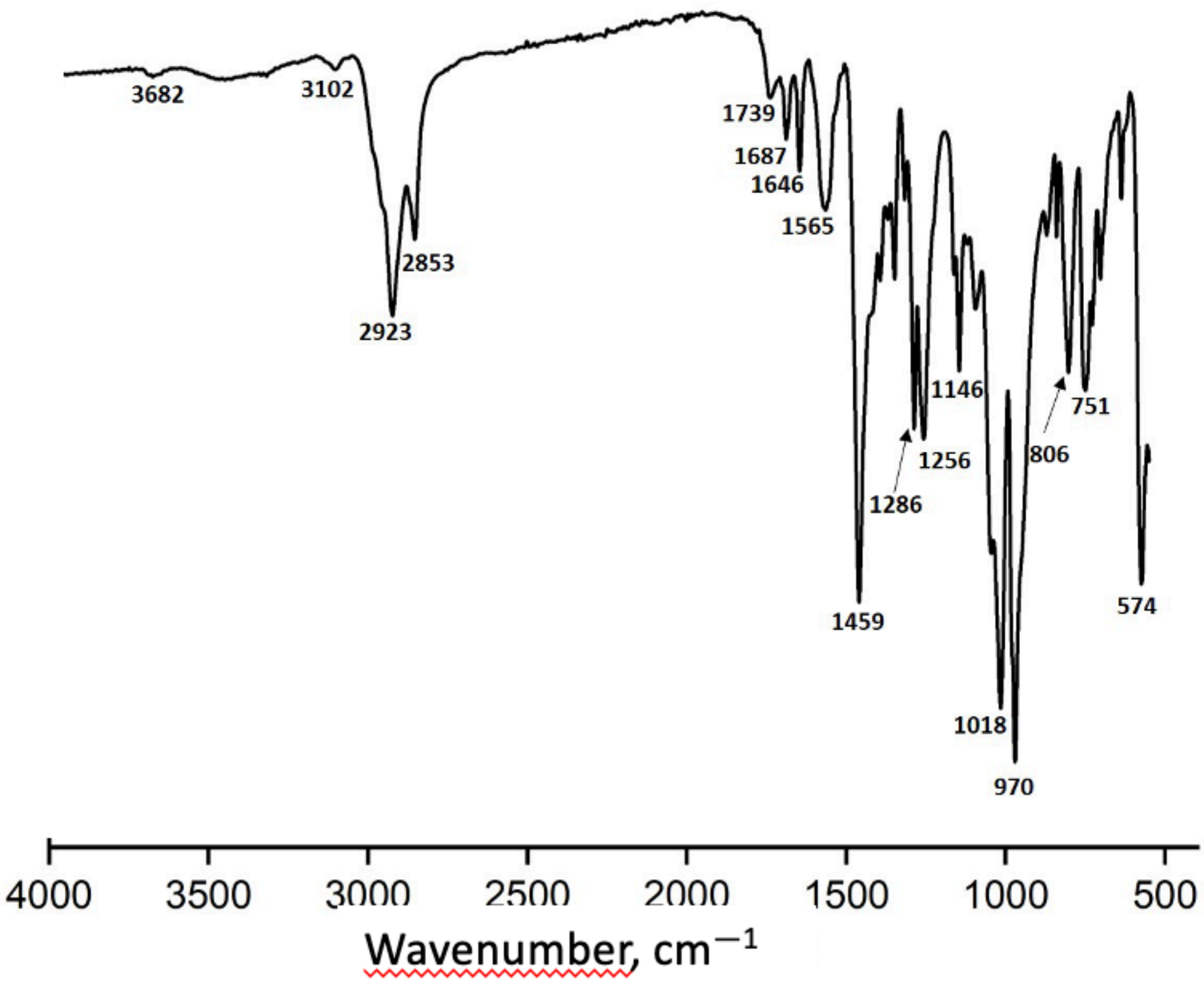

Figure S57. FT-IR spectrum of $ZnTF_4PPP$ (neat).

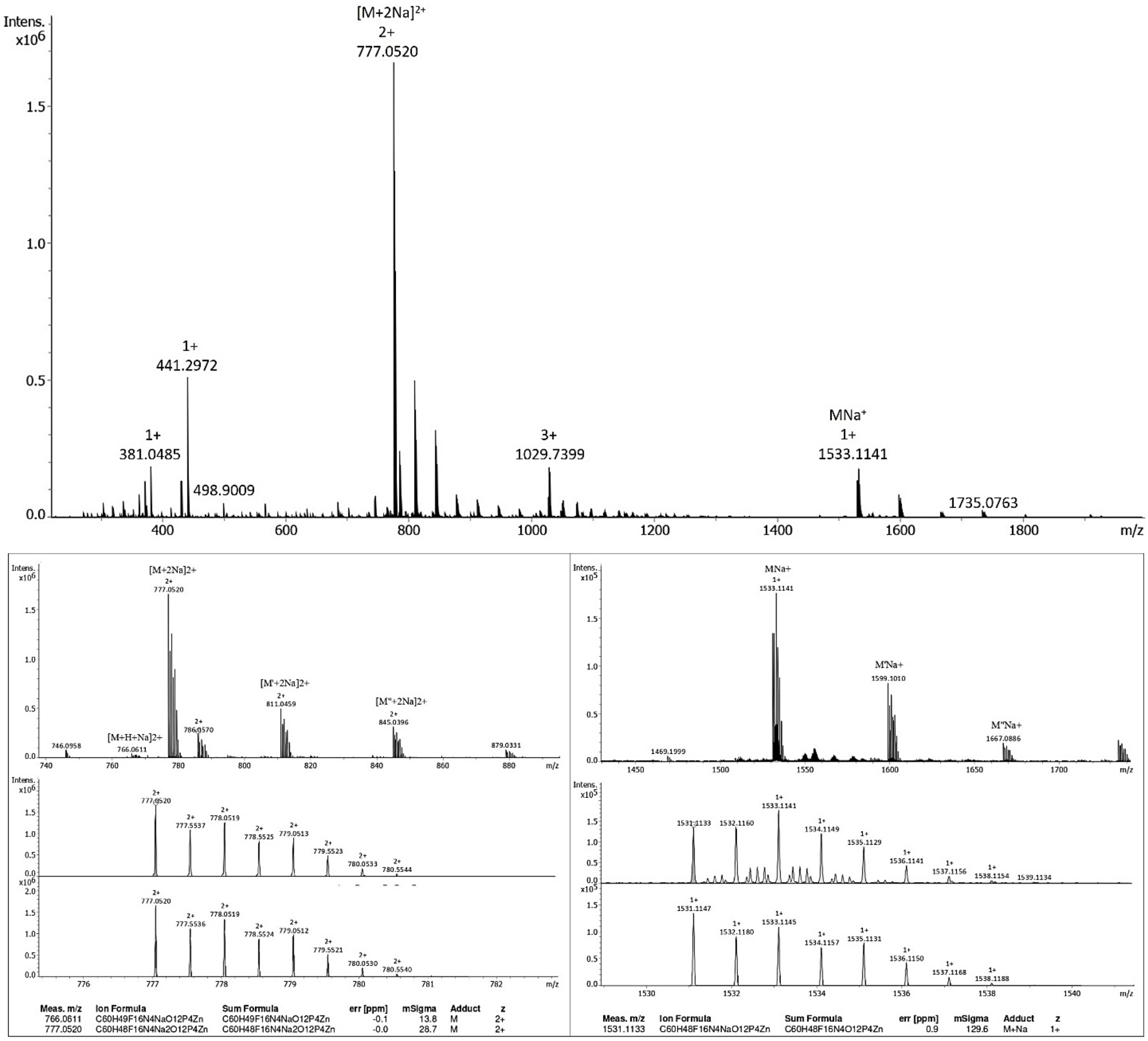

Figure S58. HRMS (ESI^+) spectrum of $ZnTF_4PPP$.

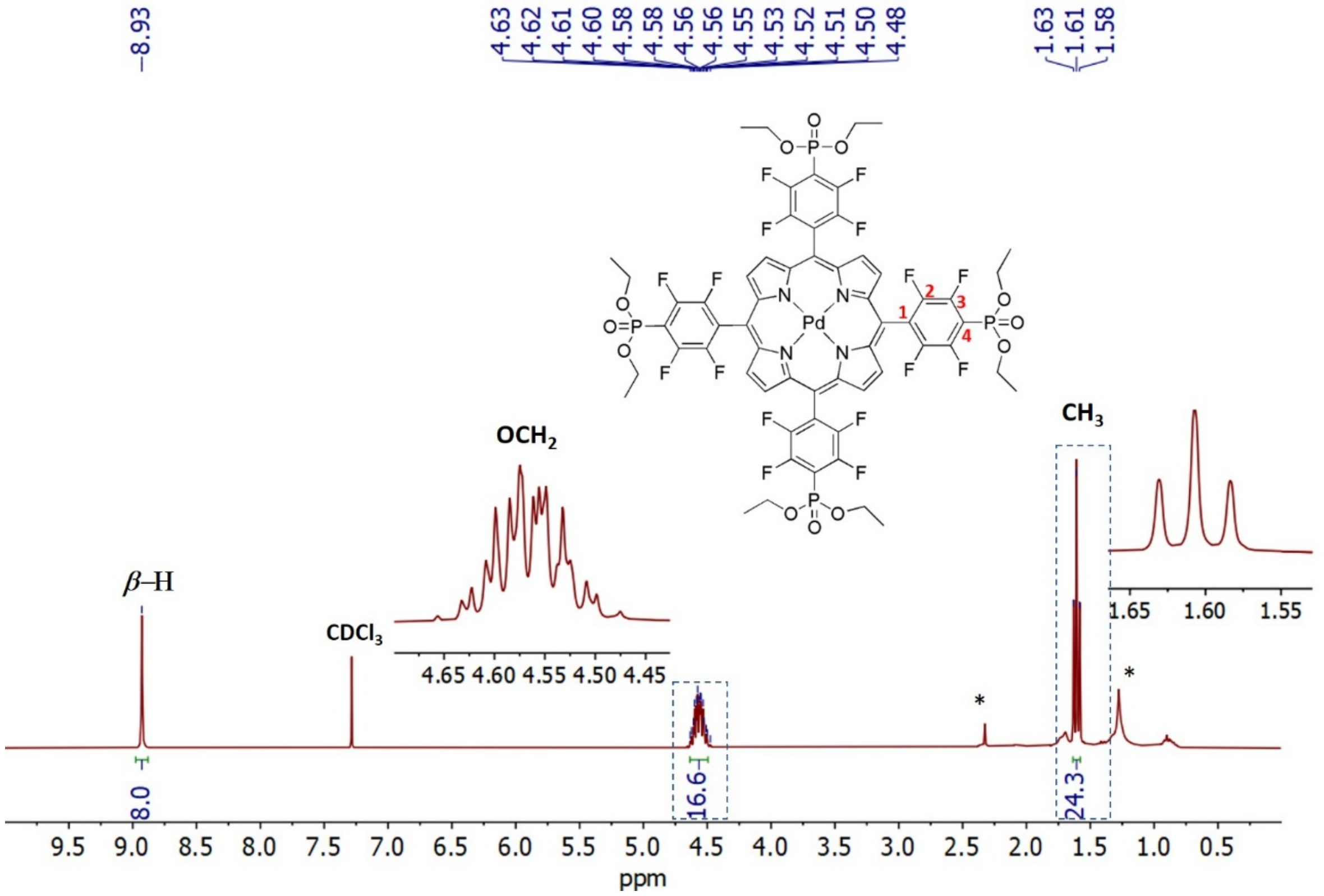

Figure S59. ^{1}H NMR spectrum of $PdTF_4PPP$ (300 MHz, $CDCl_3$, 298 K).

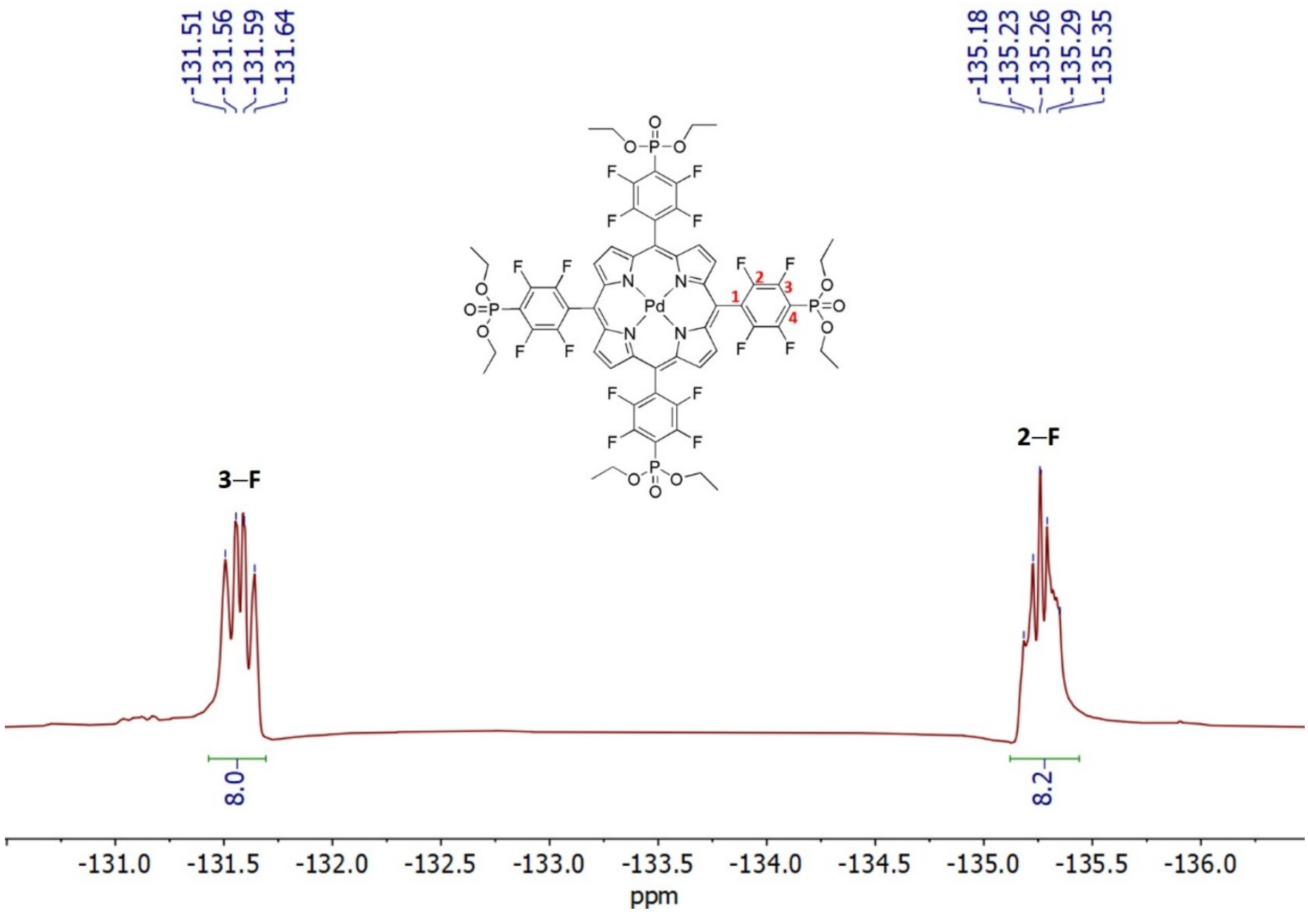

Figure S60. ^{19}F NMR spectrum of $PdTF_4PPP$ (282 MHz, $CDCl_3$, 298 K).

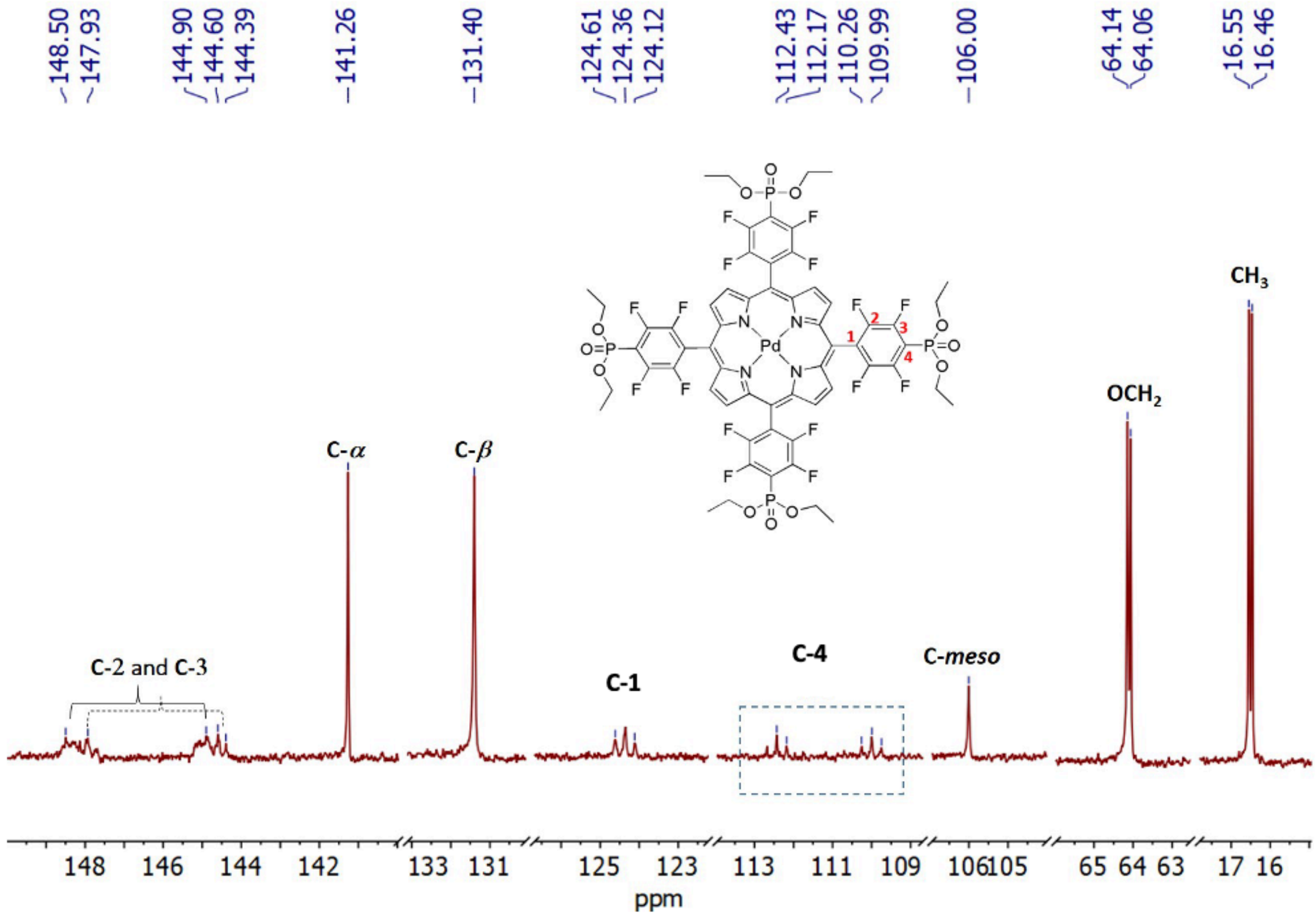

Figure S61. $^{13}C\{^{1}H\}$ NMR spectrum of PdTF$_4$PPP (75 MHz, $CDCl_3$, 298 K).

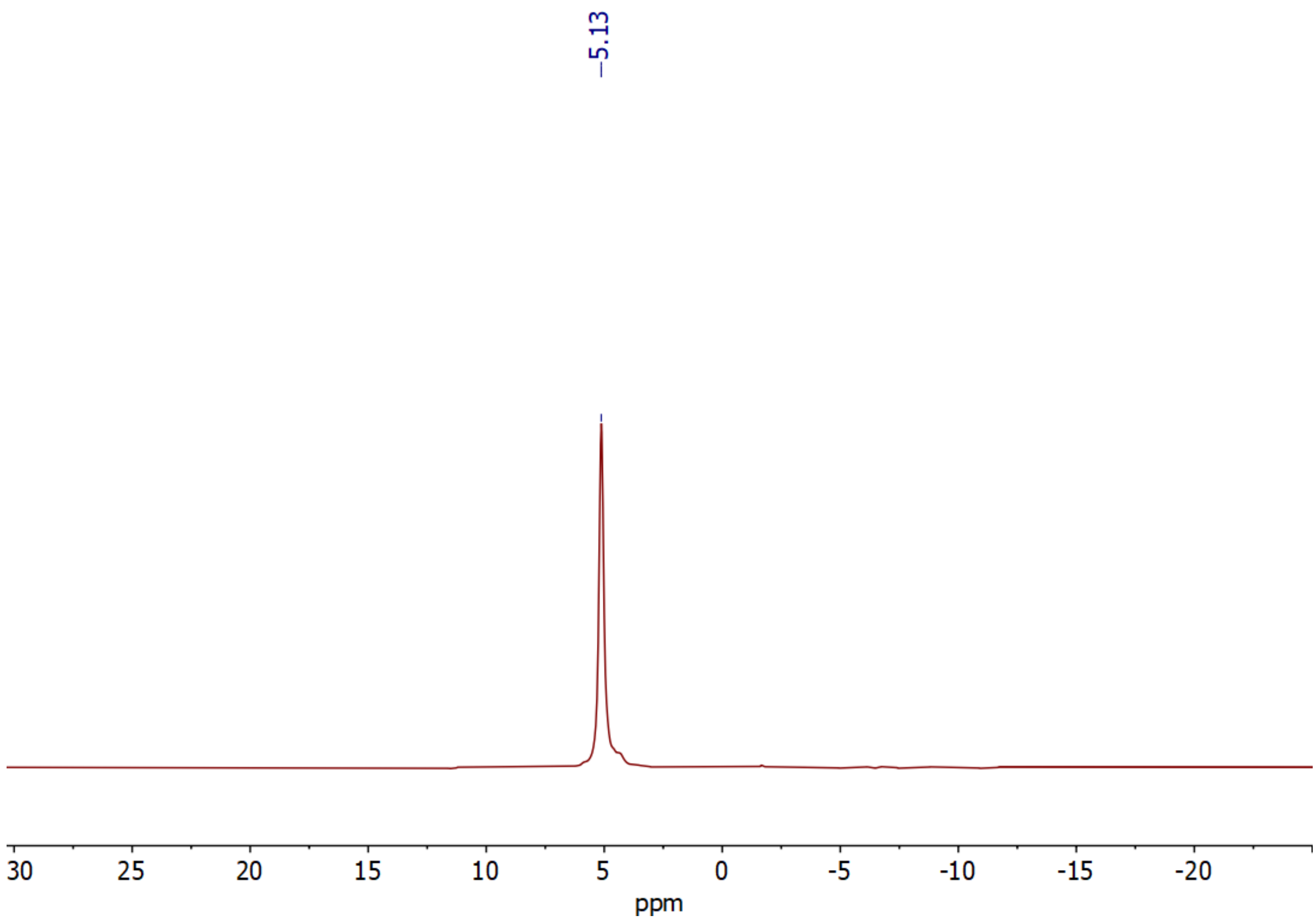

Figure S62. $^{31}P\{^{1}H\}$ NMR spectrum of PdTF$_4$PPP (121 MHz, $CDCl_3$, 298 K).

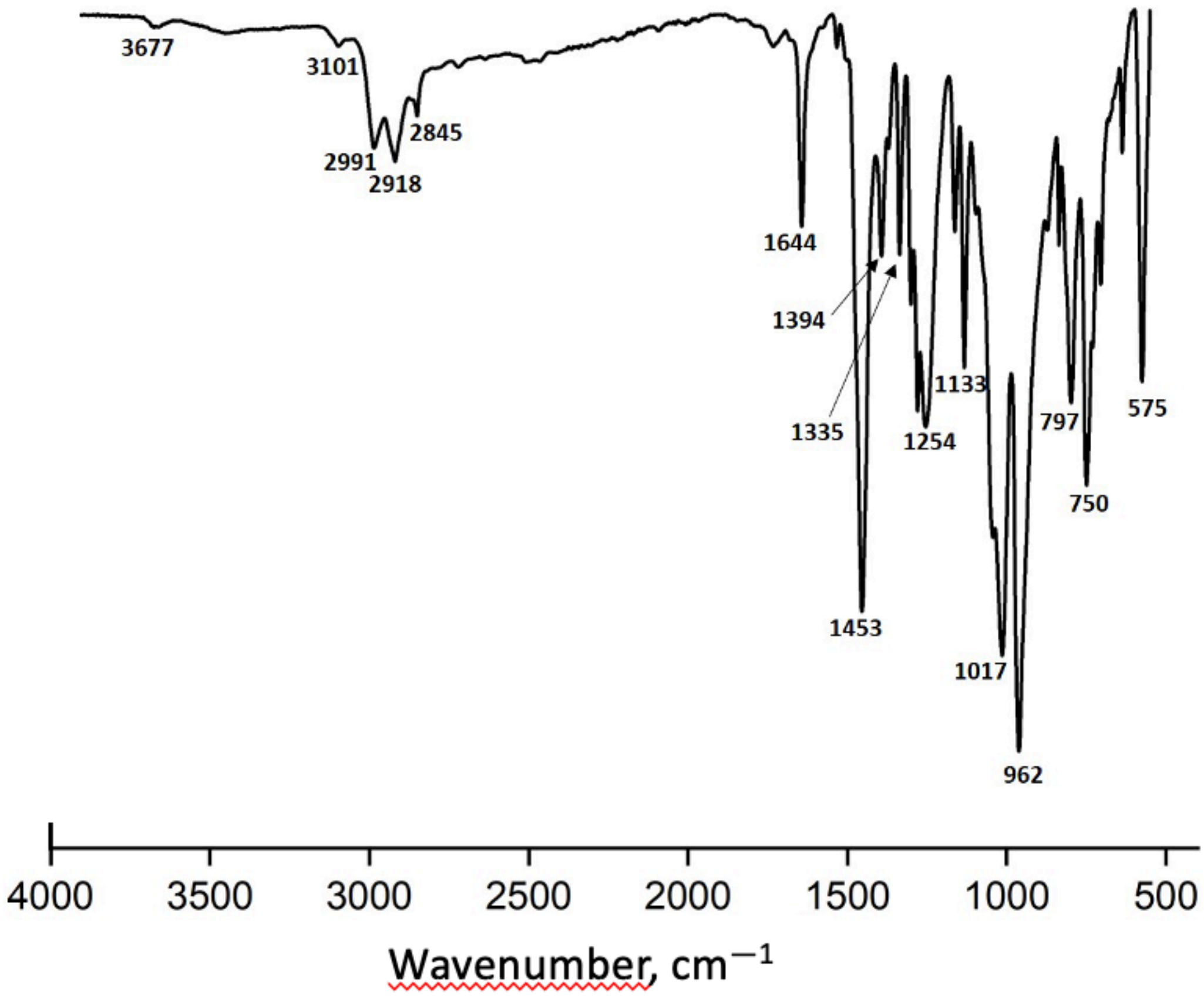

Figure S63. FT-IR spectrum of $PdTF_4PPP$ (neat).

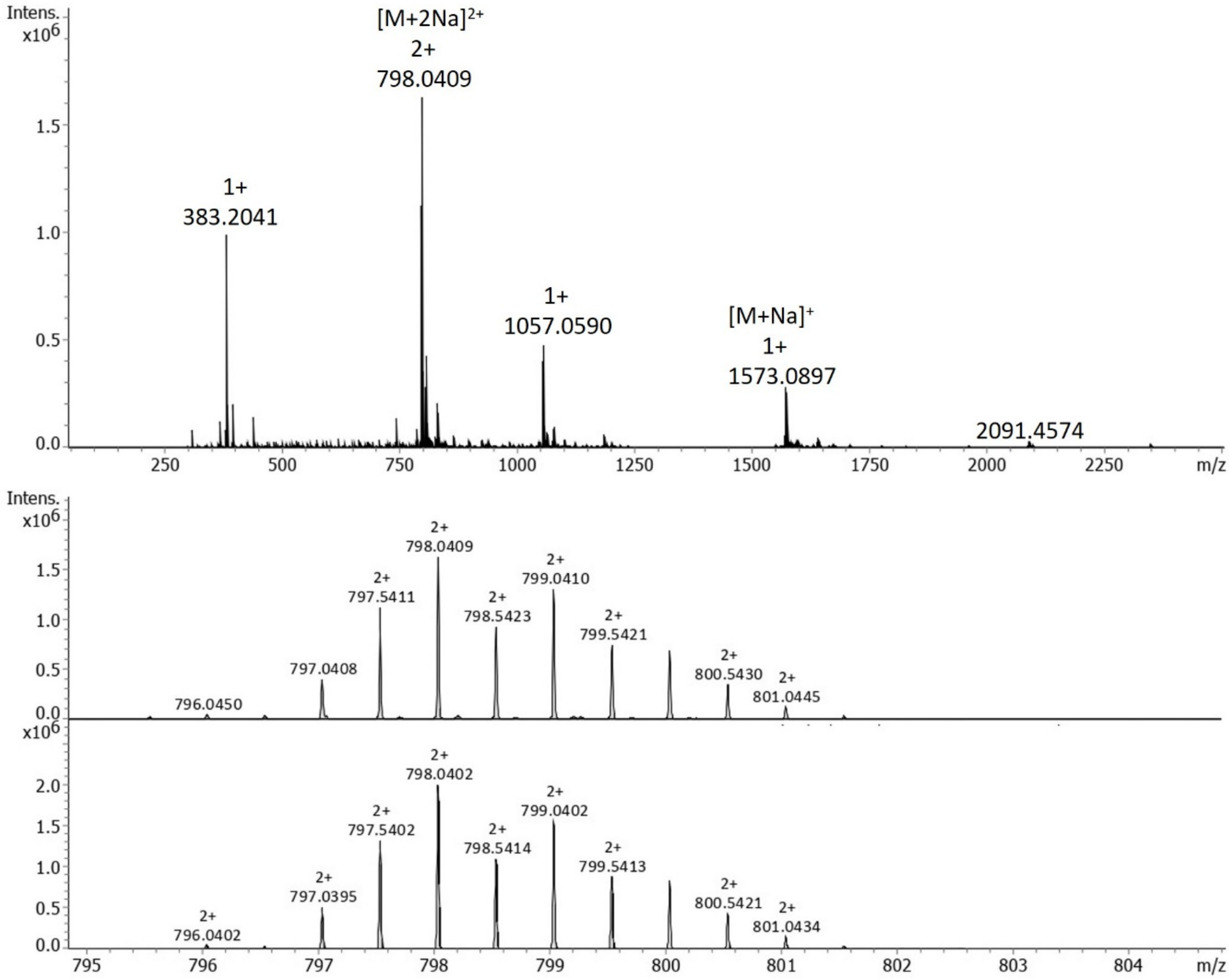

Meas. m/z	Ion Formula	Sum Formula	err [ppm]	mSigma	Adduct	z
798.0409	C60H48F16N4Na2O12P4Pd	C60H48F16N4Na2O12P4Pd	-0.9	15.7	M	1+

Figure S64. HRMS (ESI$^+$) spectrum of PdTF$_4$PPP.

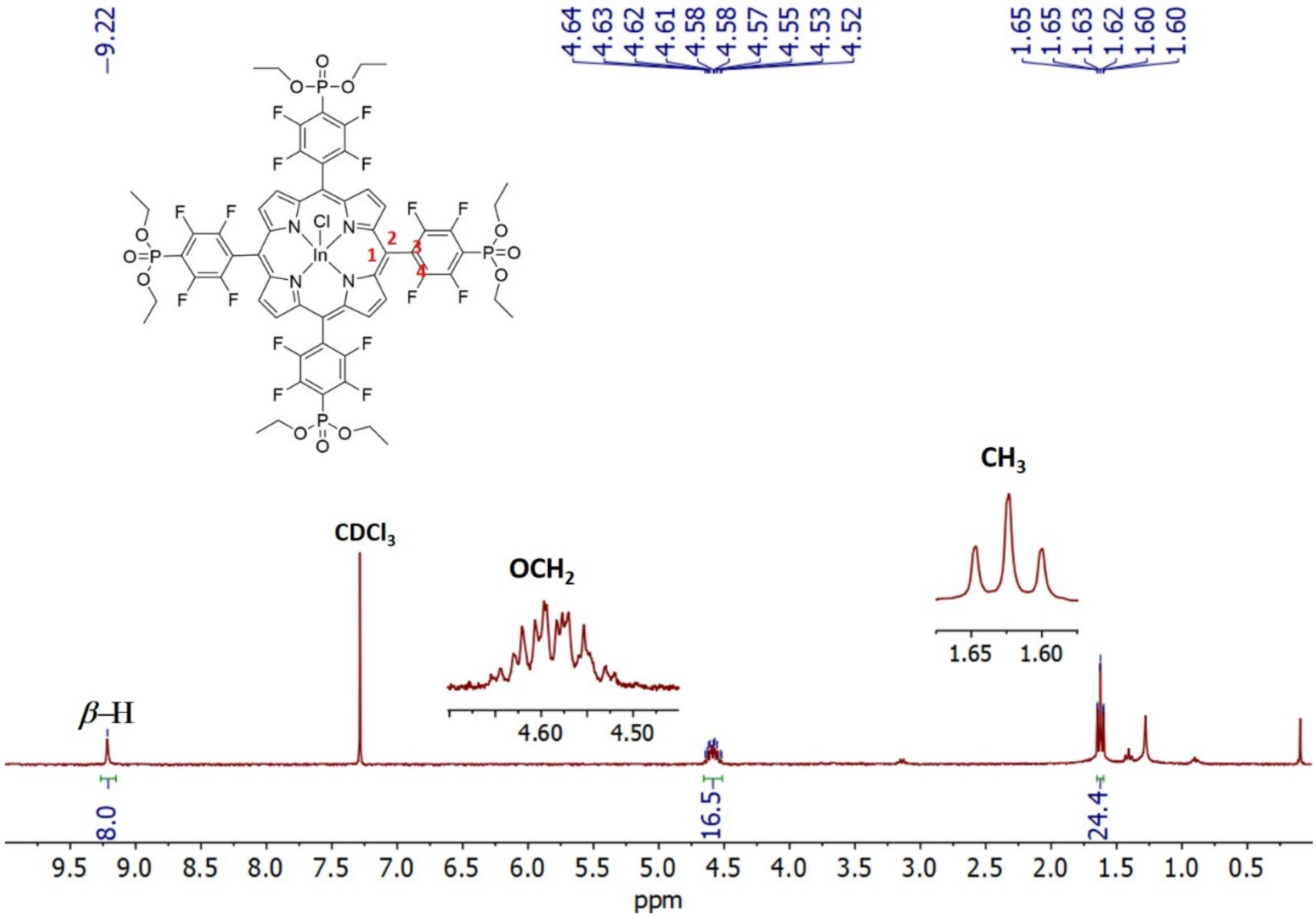

Figure S65. ^{1}H NMR spectrum of $InTF_4PPP$ (300 MHz, $CDCl_3$, 298 K).

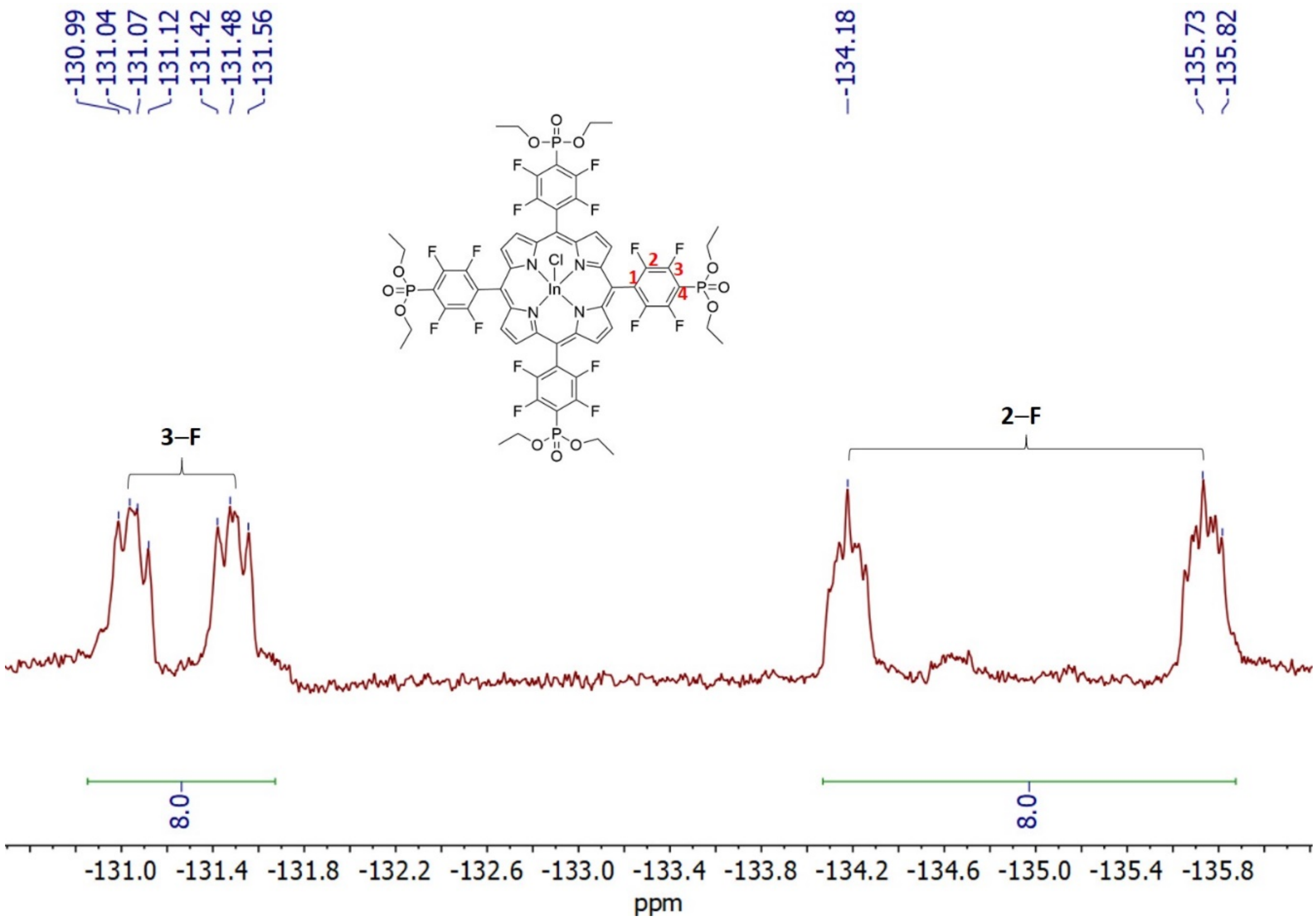

Figure S66. ^{19}F NMR spectrum of $InTF_4PPP$ (282 MHz, $CDCl_3$, 298 K).

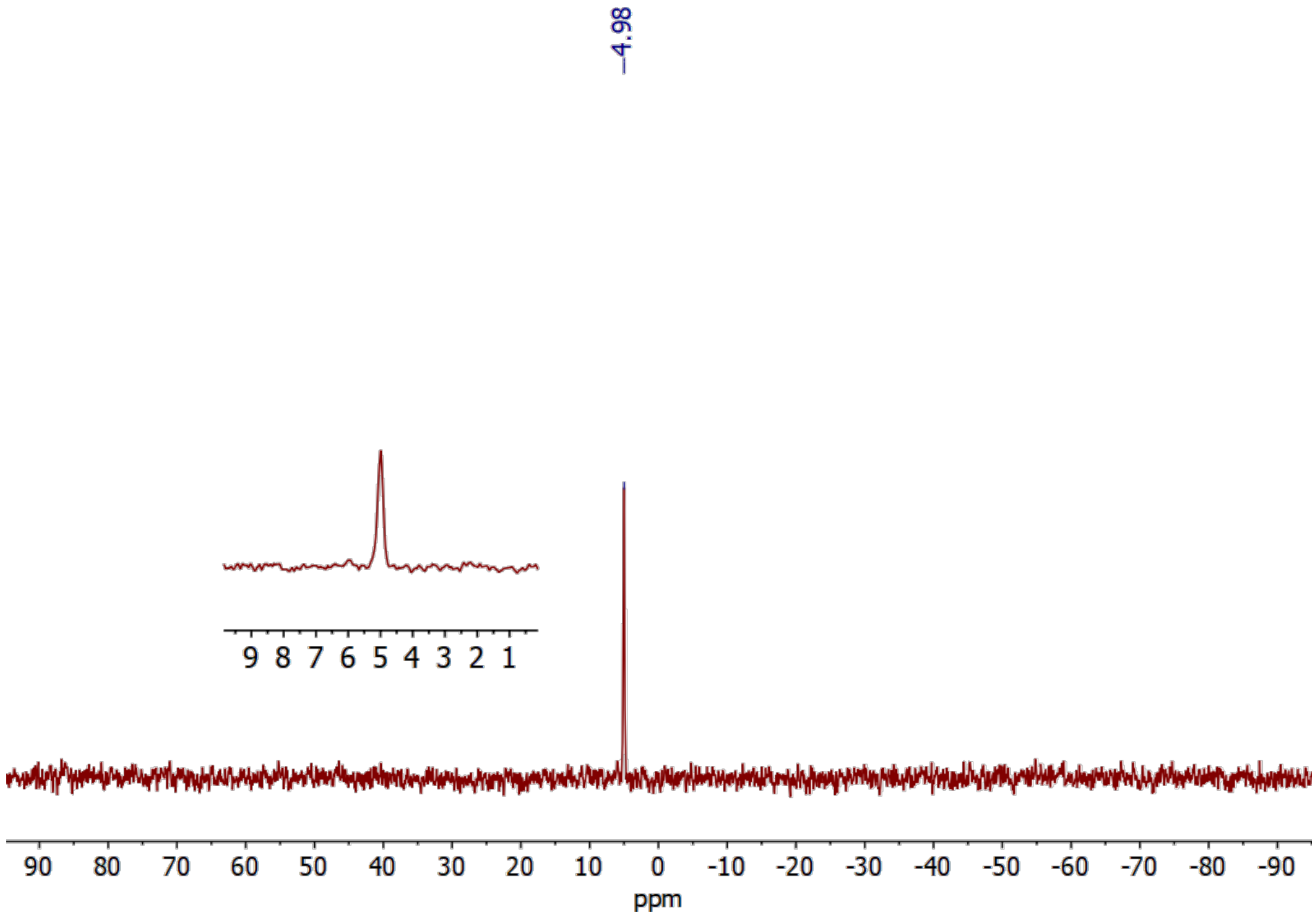

Figure S67. $^{31}P\{^{1}H\}$ NMR spectrum of $InTF_4PPP$ (121 MHz, $CDCl_3$, 298 K).

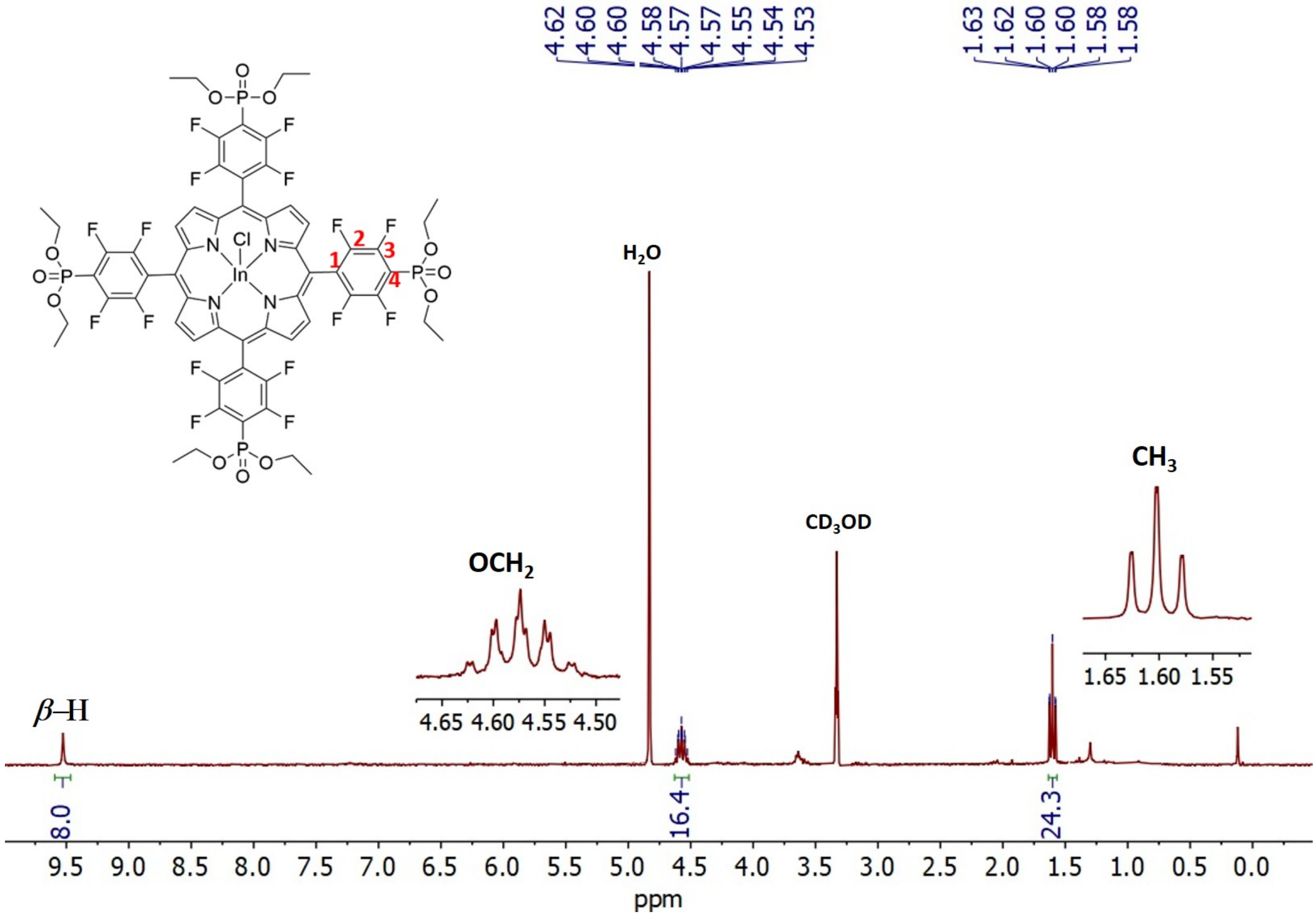

Figure S68. ^{1}H NMR spectrum of $InTF_4PPP$ (300 MHz, CD_3OD, 298 K).

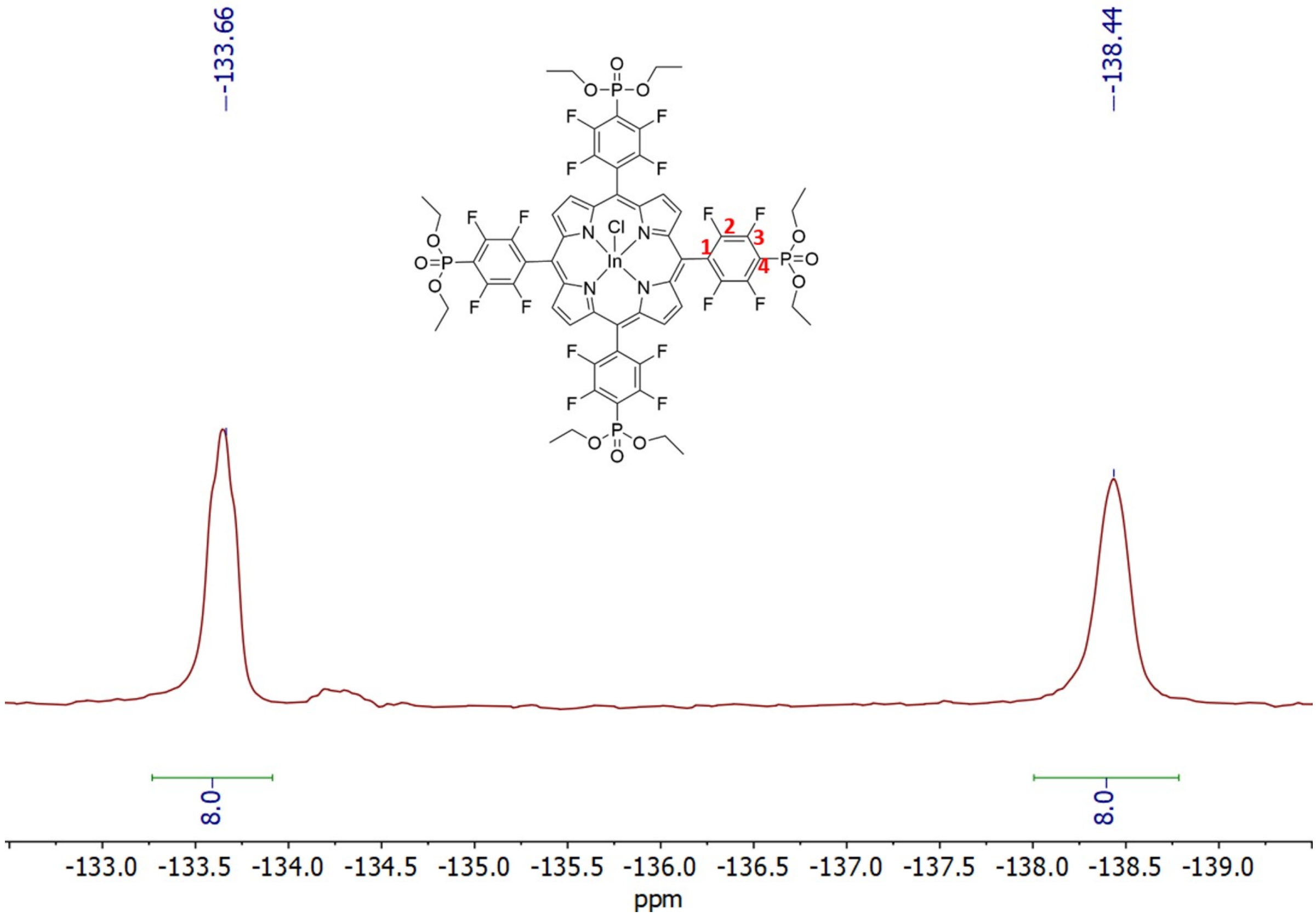

Figure S69. ^{19}F NMR spectrum of InTF$_4$PPP (282 MHz, CD_3OD, 298 K).

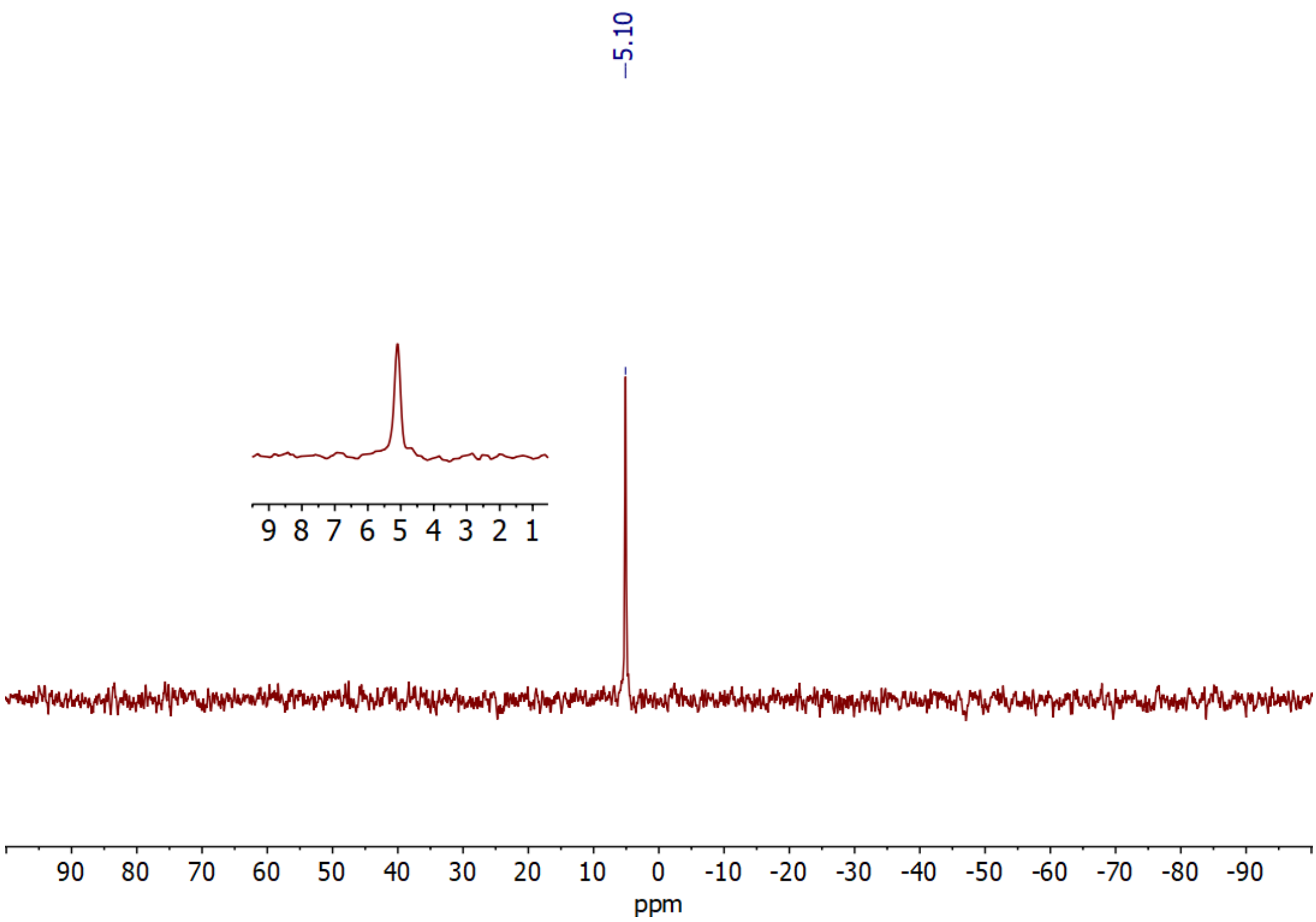

Figure S70. $^{31}P\{^1H\}$ NMR spectrum of InTF$_4$PPP (121 MHz, CD_3OD, 298 K)

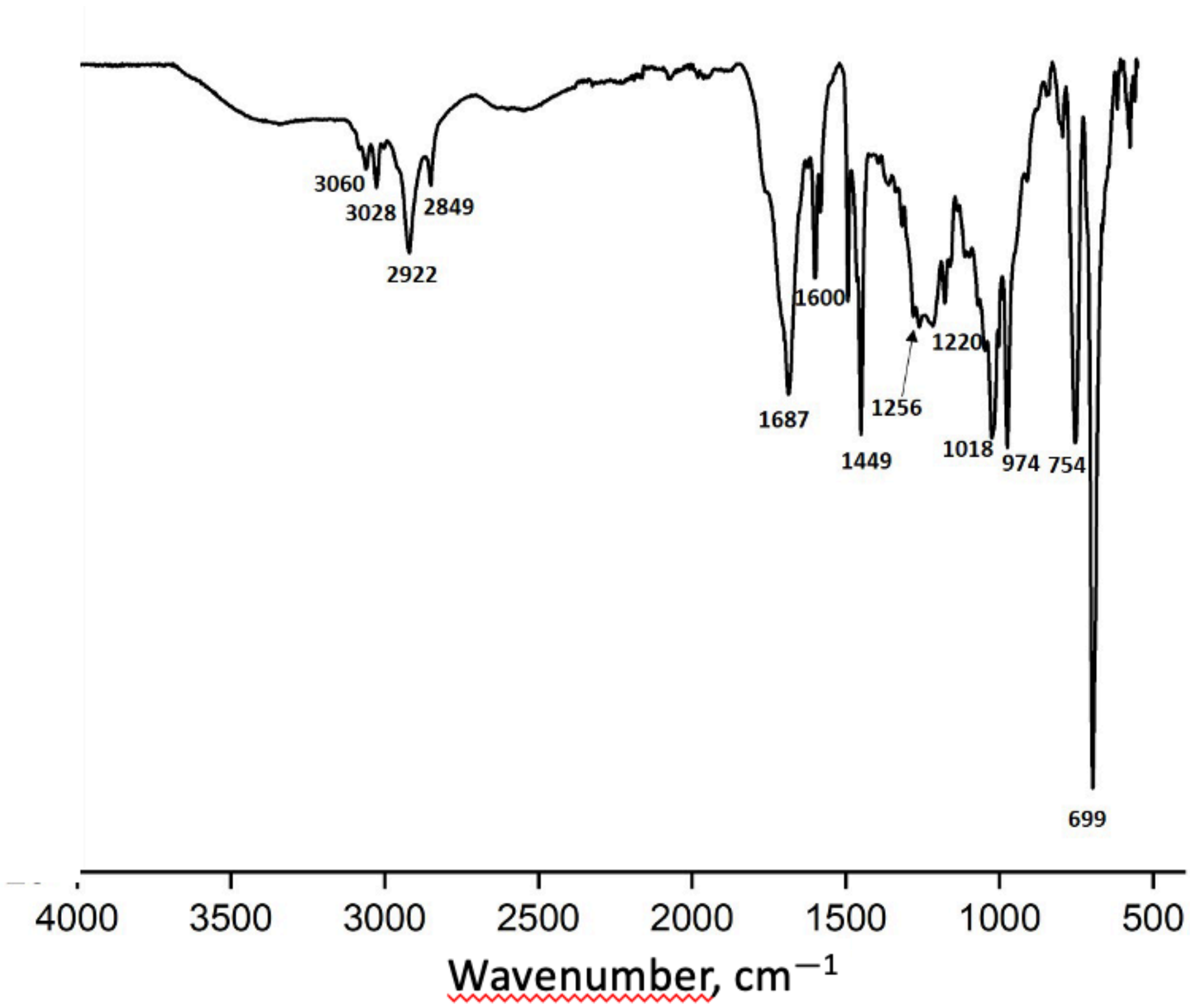

Figure S71. FT-IR spectrum of $InTF_4PPP$ (neat).

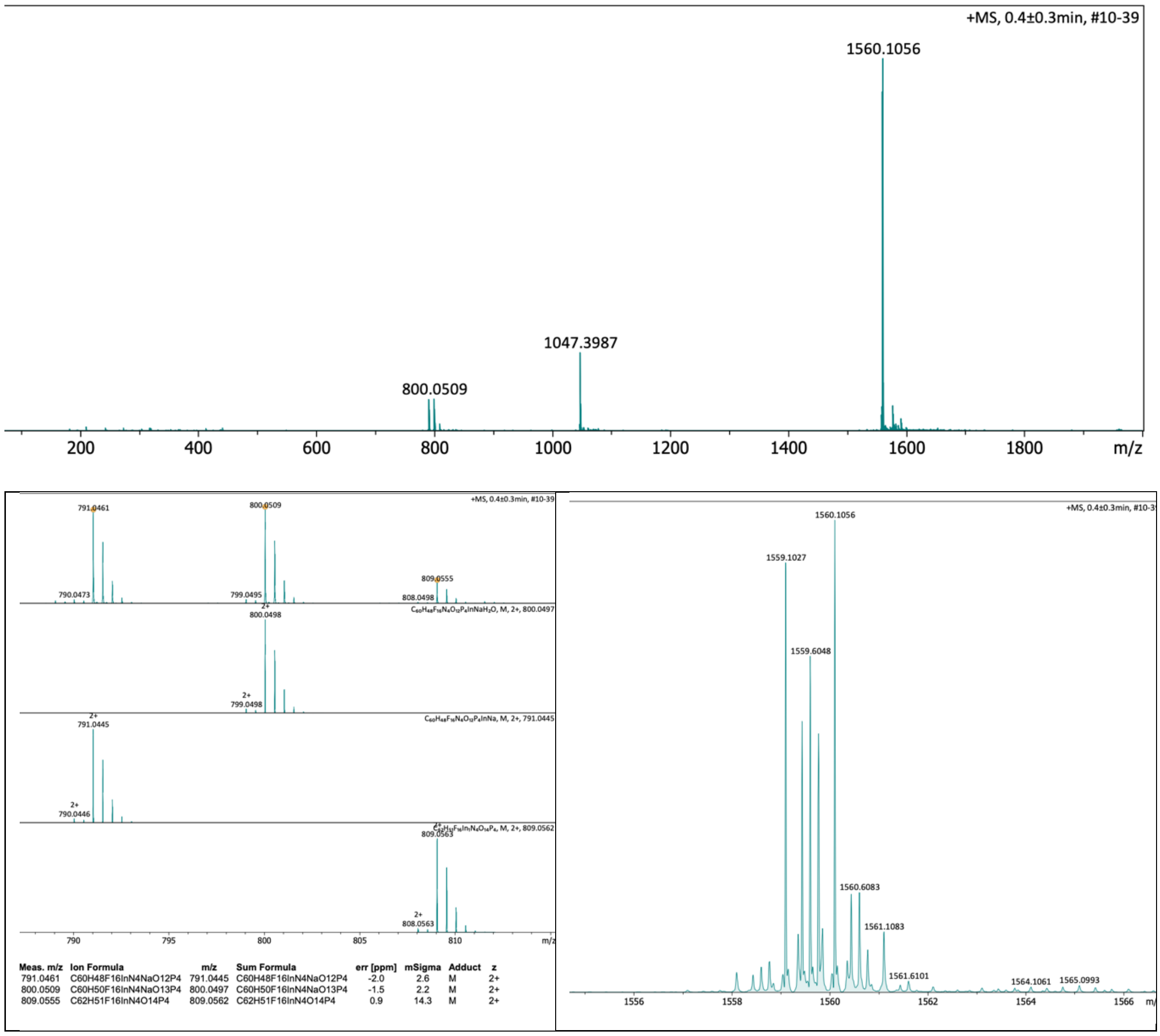

Figure S72. HRMS (ESI^+) spectrum of $InTF_4PPP$.

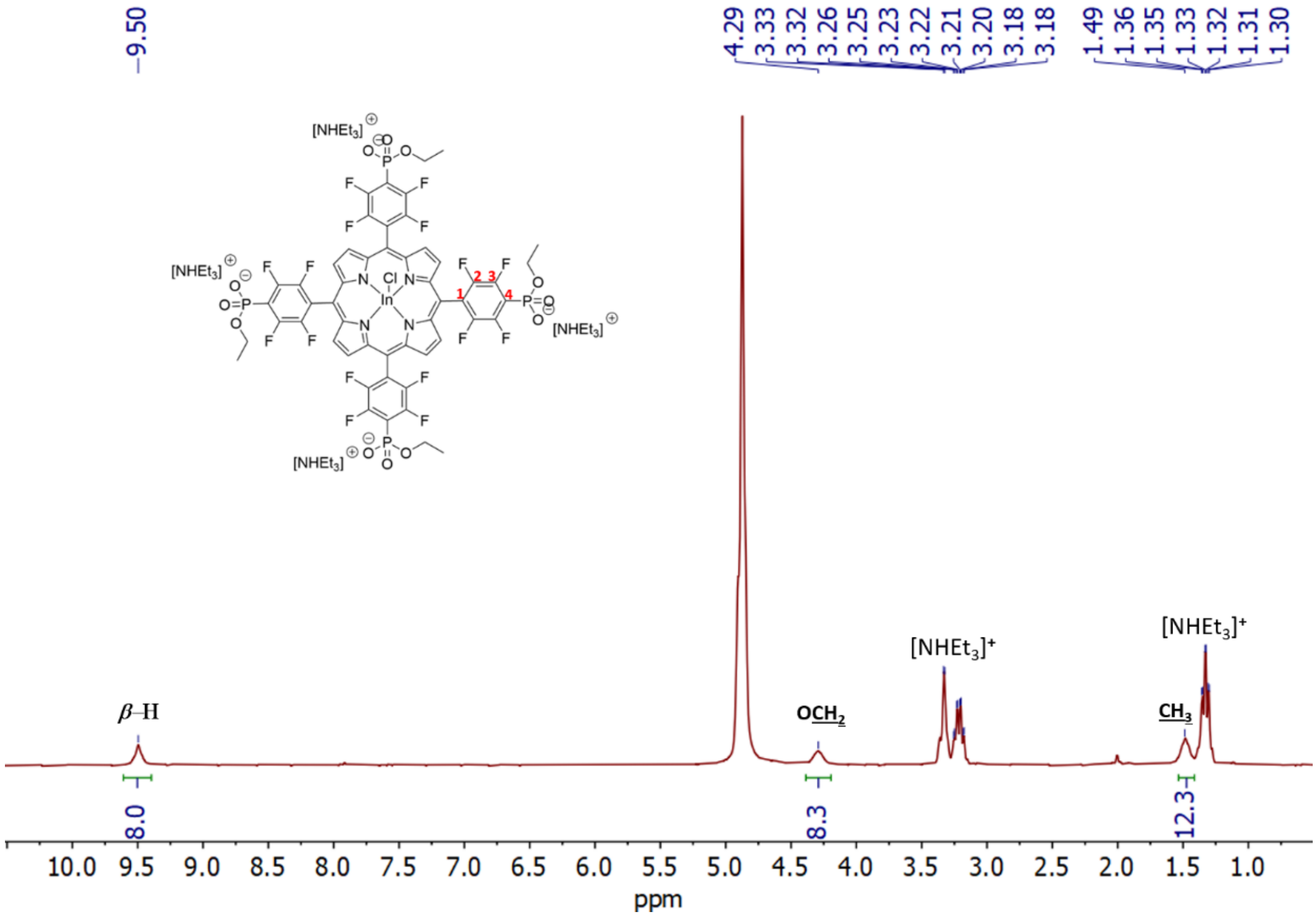

Figure S73. ^{1}H NMR spectrum of InTF$_4$PPP-M (300 MHz, CD_3OD, 298 K).

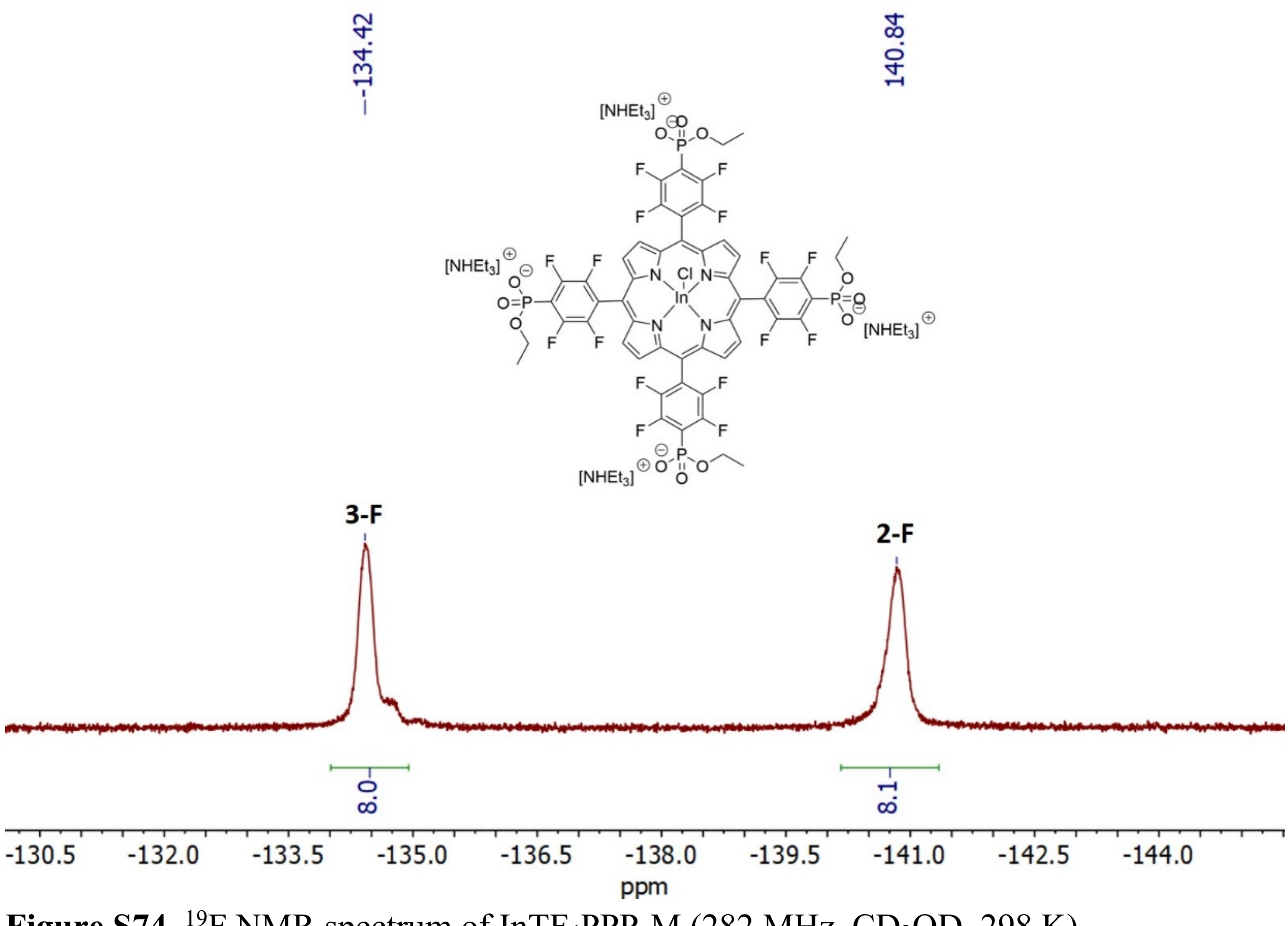

Figure S74. ^{19}F NMR spectrum of InTF$_4$PPP-M (282 MHz, CD_3OD, 298 K).

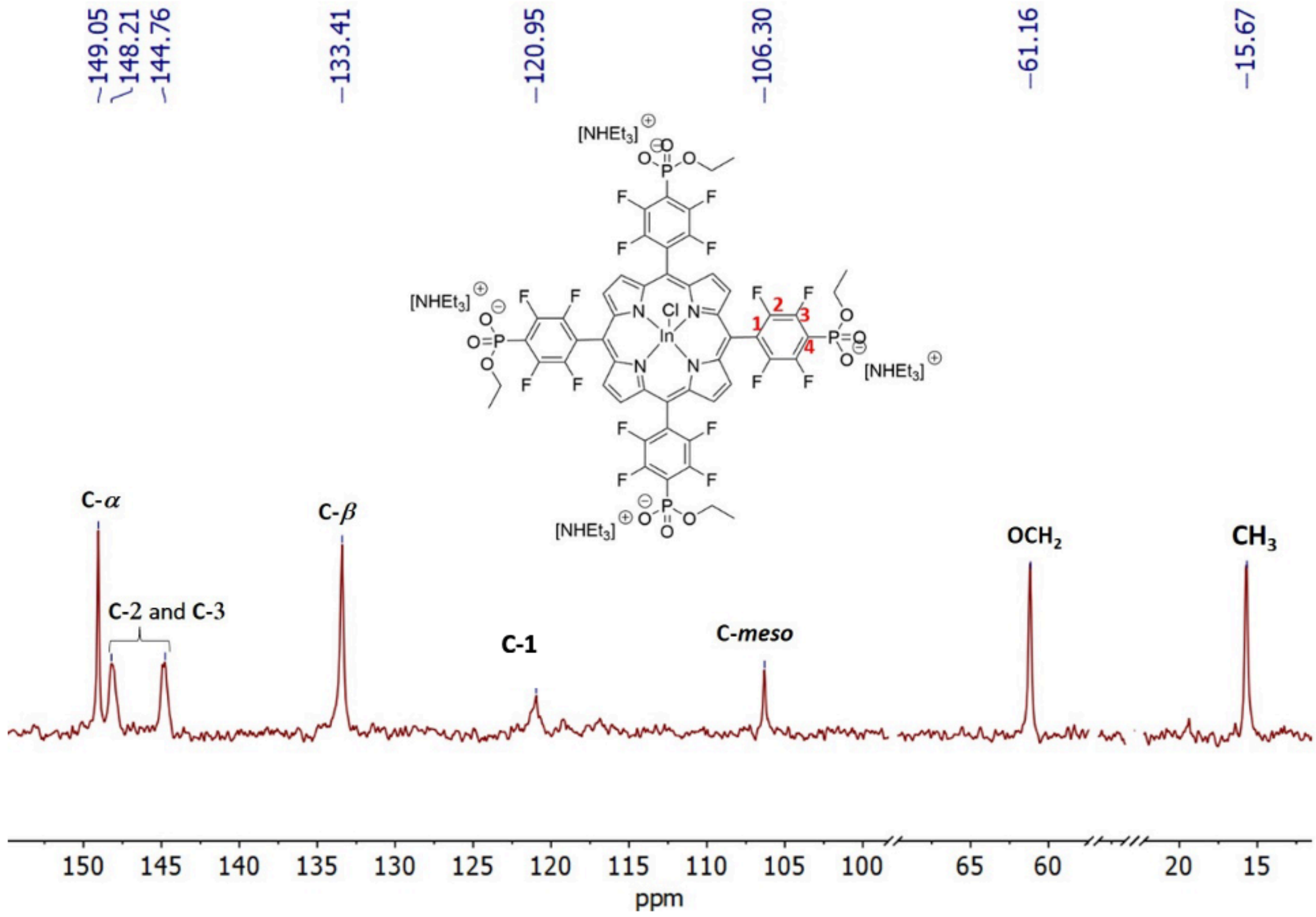

Figure S75. $^{13}C\{^{1}H\}$ NMR spectrum of $InTF_4PPP$-M (75 MHz, CD_3OD, 298 K).

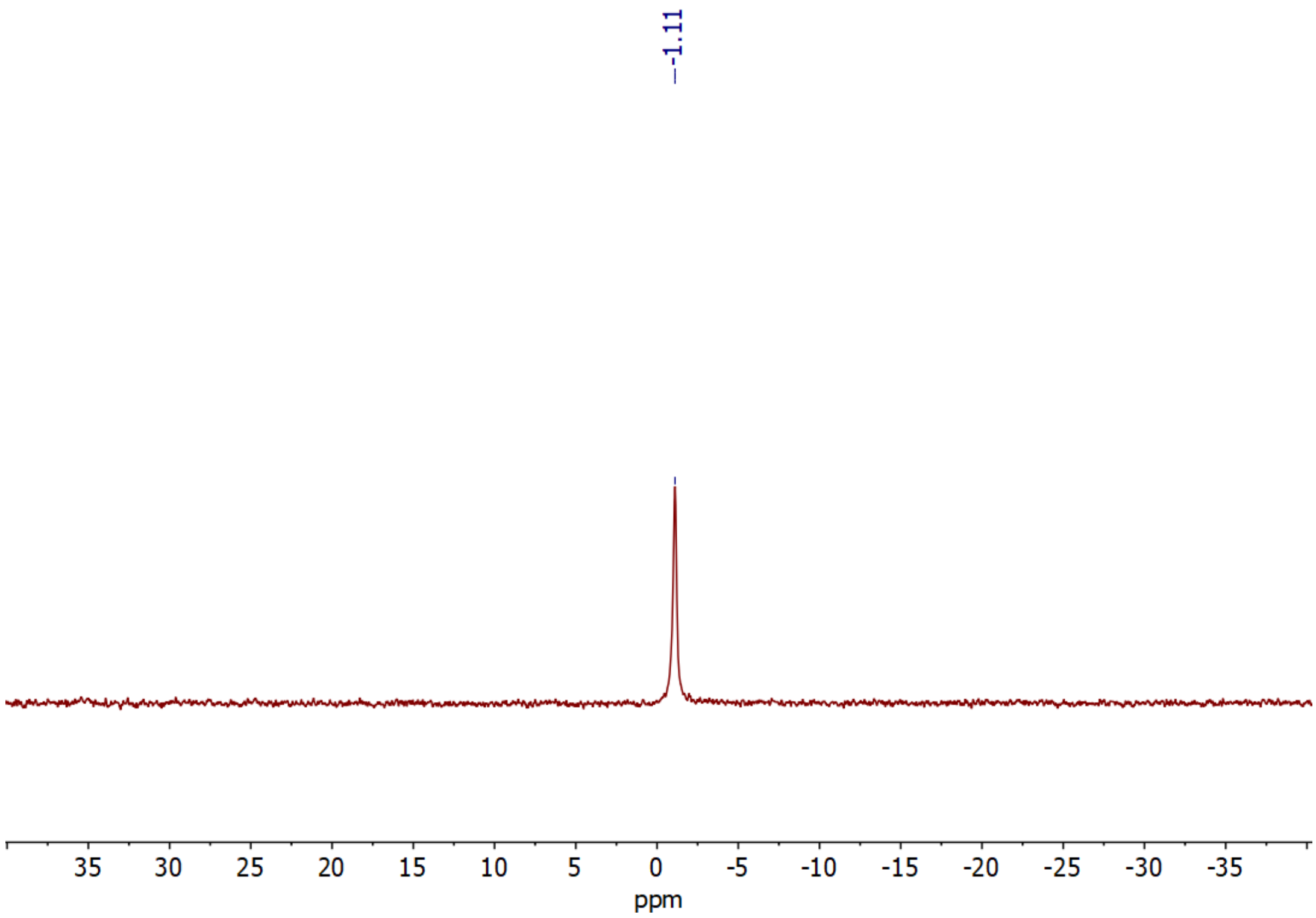

Figure S76. $^{31}P\{^{1}H\}$ NMR spectrum of $InTF_4PPP$-M (121 MHz, CD_3OD, 298 K).

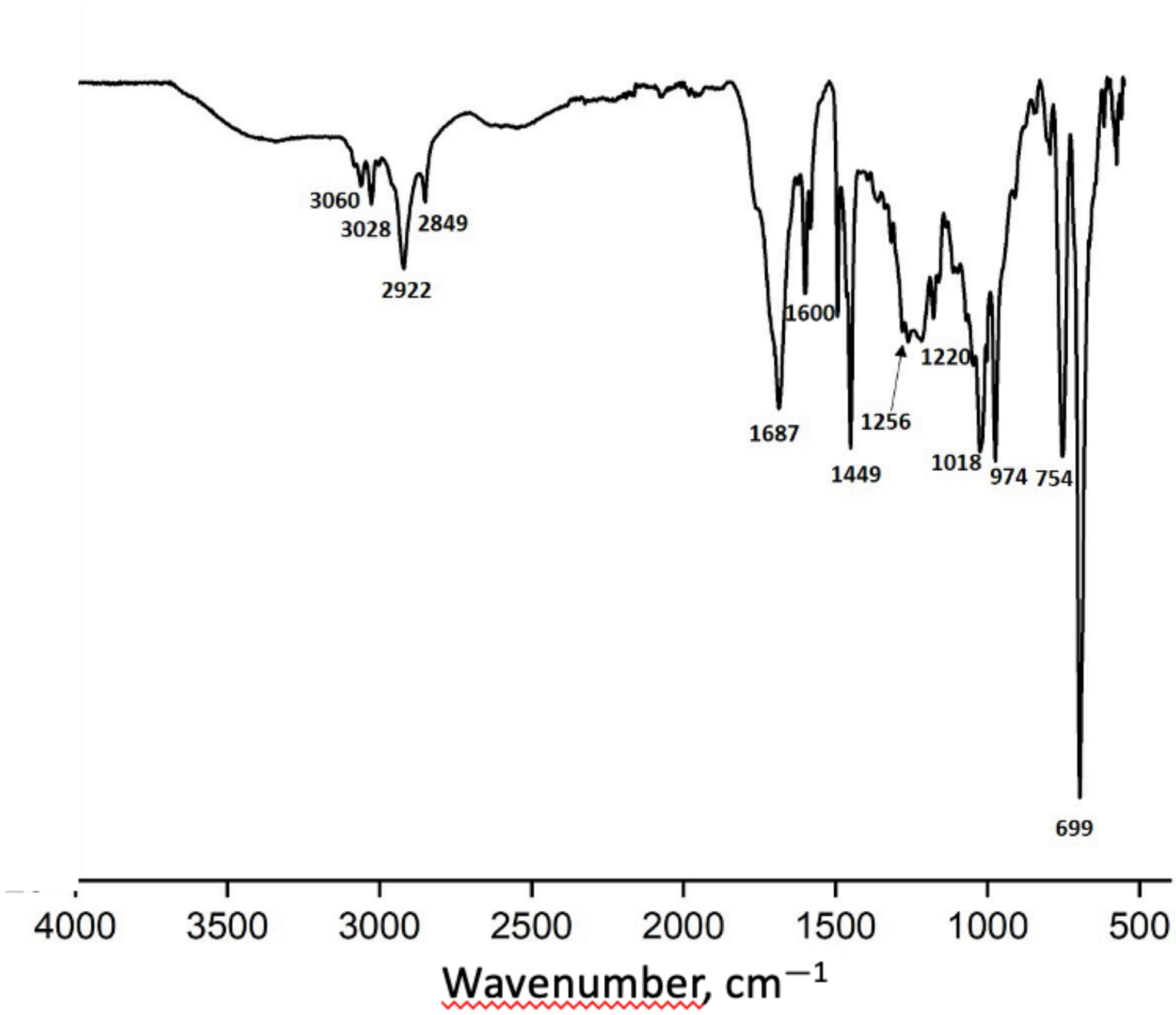

Figure S77. FT-IR spectrum of $InTF_4PPP$-M (neat).

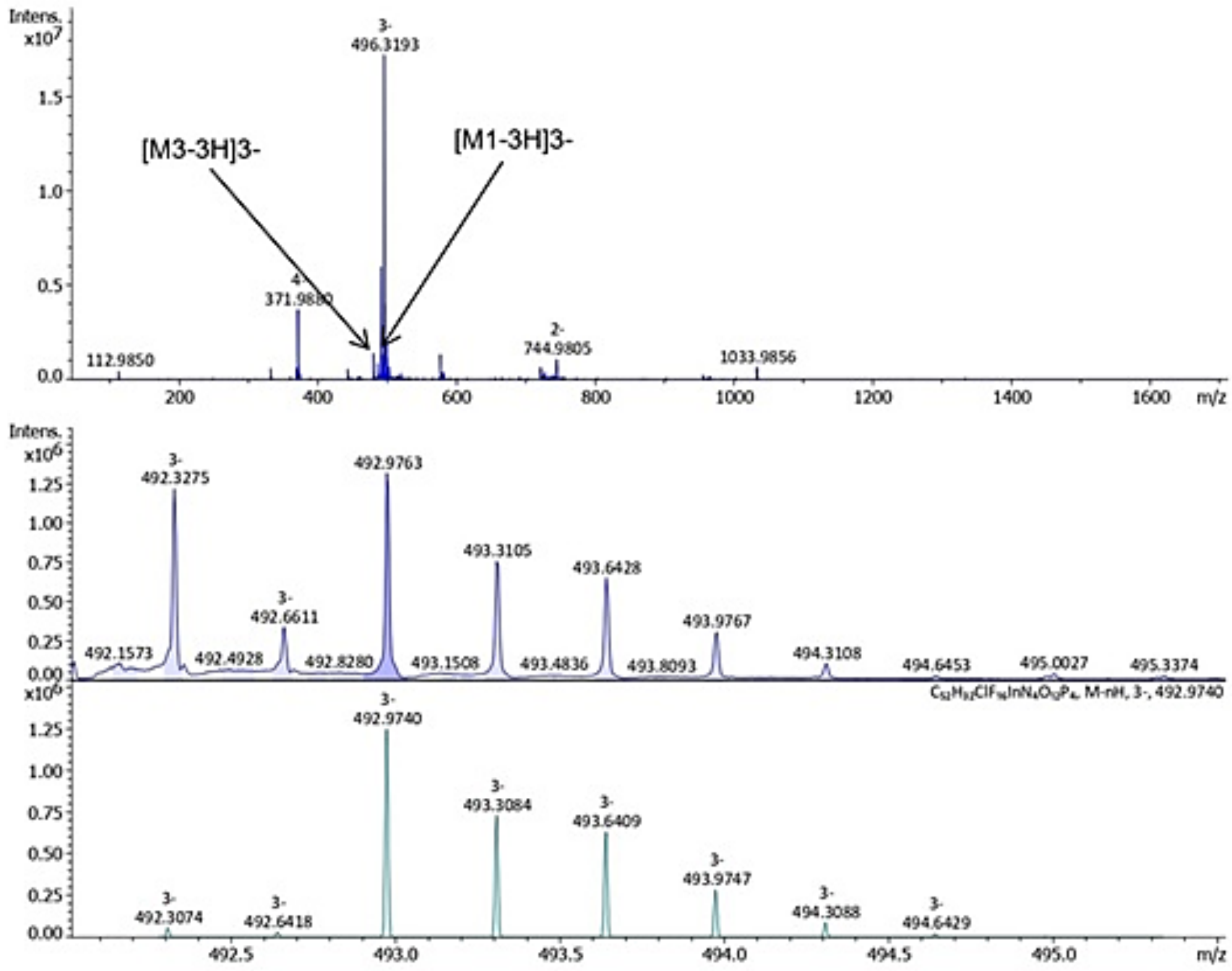

Meas. m/z	Ion Formula	m/z	Sum Formula	err [ppm]	mSigma	Adduct	z
492.9763	C52H29ClF16InN4O12P4	492.9740	C52H32ClF16InN4O12P4	-4.5	19.3	M-H	3-

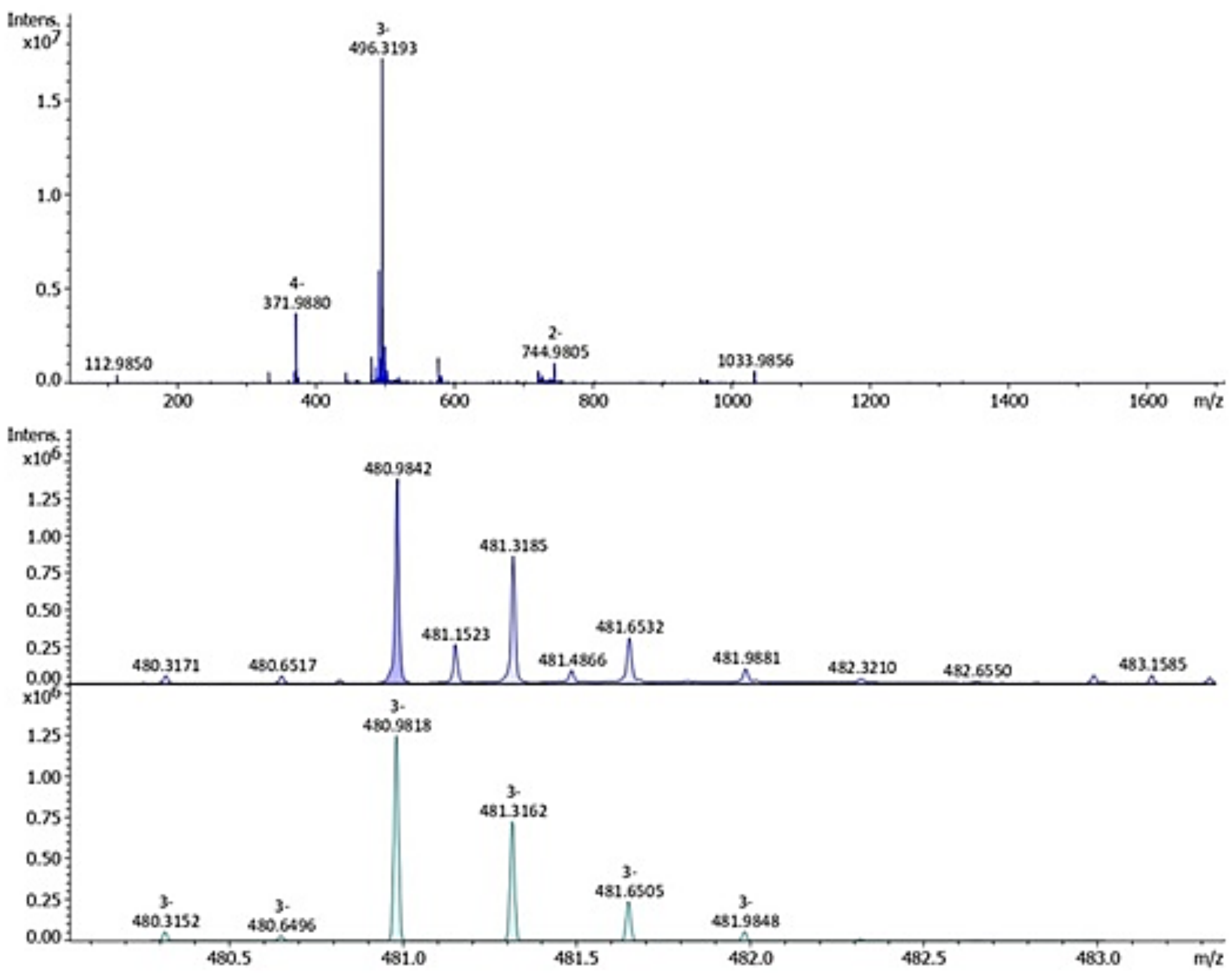

Meas. m/z	Ion Formula	m/z	Sum Formula	err [ppm]	mSigma	Adduct	z
480.9842	C52H28F16InN4O12P4	480.9818	C52H31F16InN4O12P4	-4.9	24.3	M-H	3-

Figure S78. HRMS (ESI⁻) spectrum of $InTF_4PPP$-M.

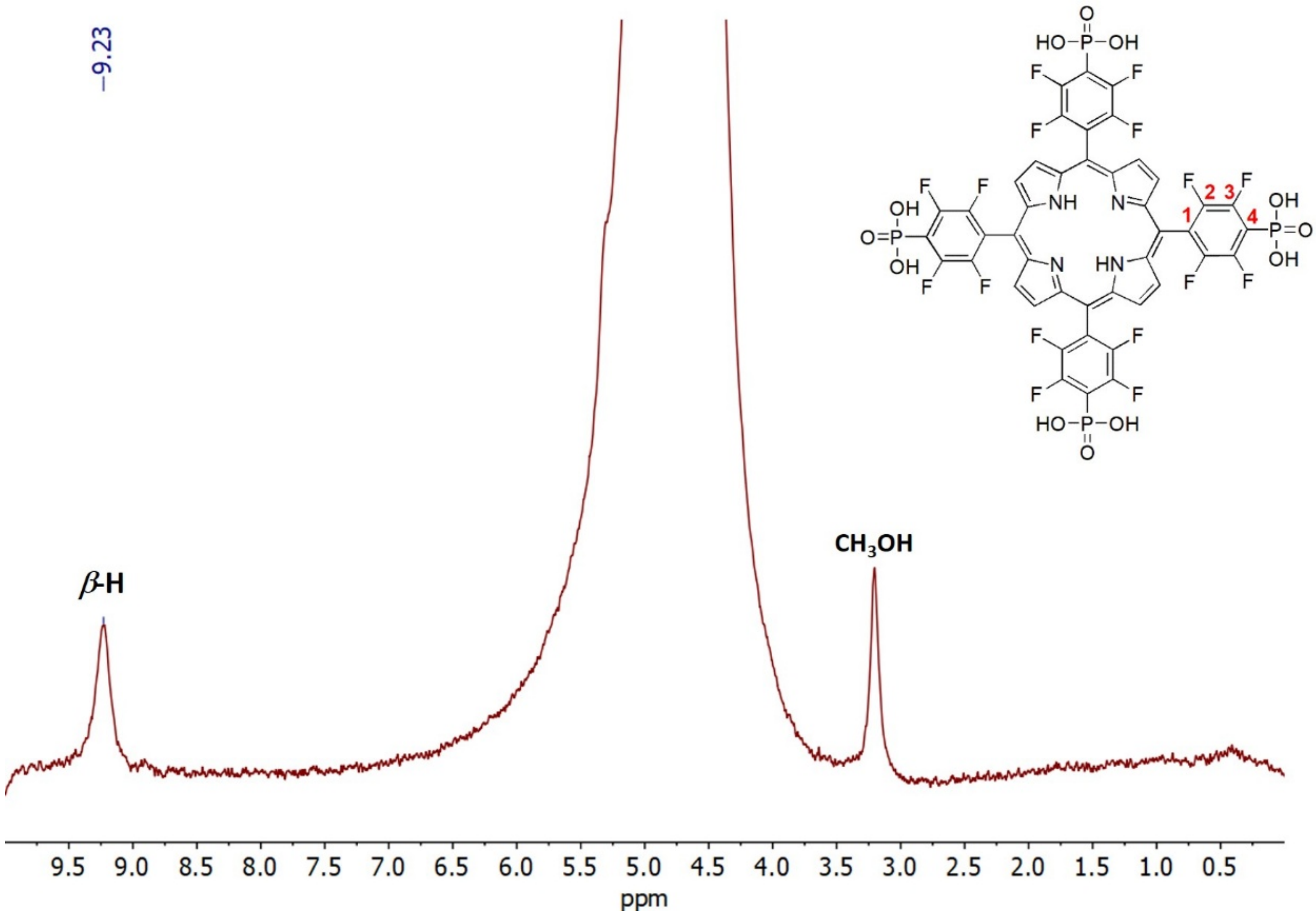

Figure S79. ^{1}H NMR spectrum of H_2TF_4PPP-A (300 MHz, D_2O + 1 drop NaOD (40% in D_2O, 298 K).

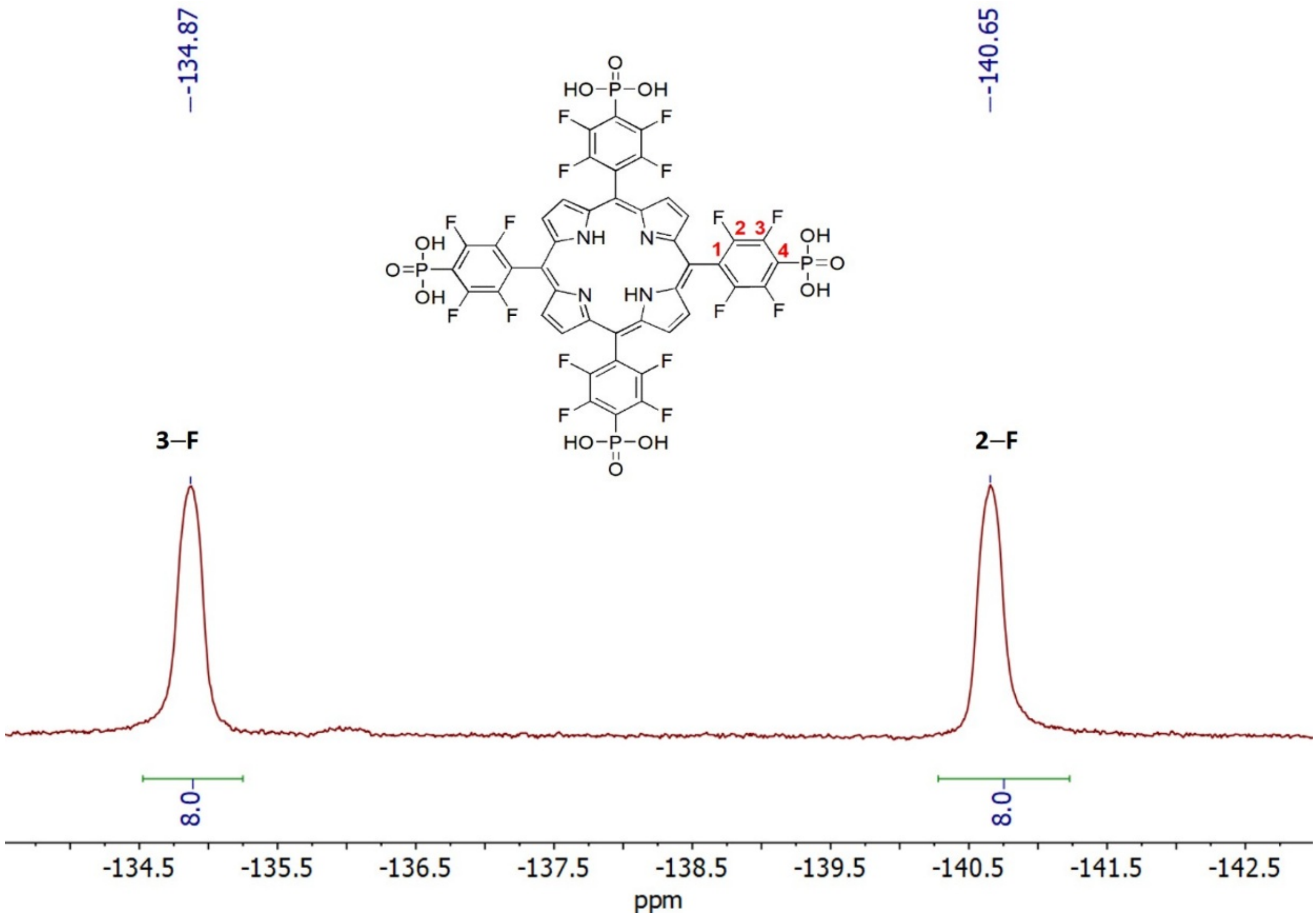

Figure S80. ^{19}F NMR spectrum of H_2TF_4PPP-A (300 MHz, D_2O + 1 drop NaOD (40% in D_2O, 298 K).

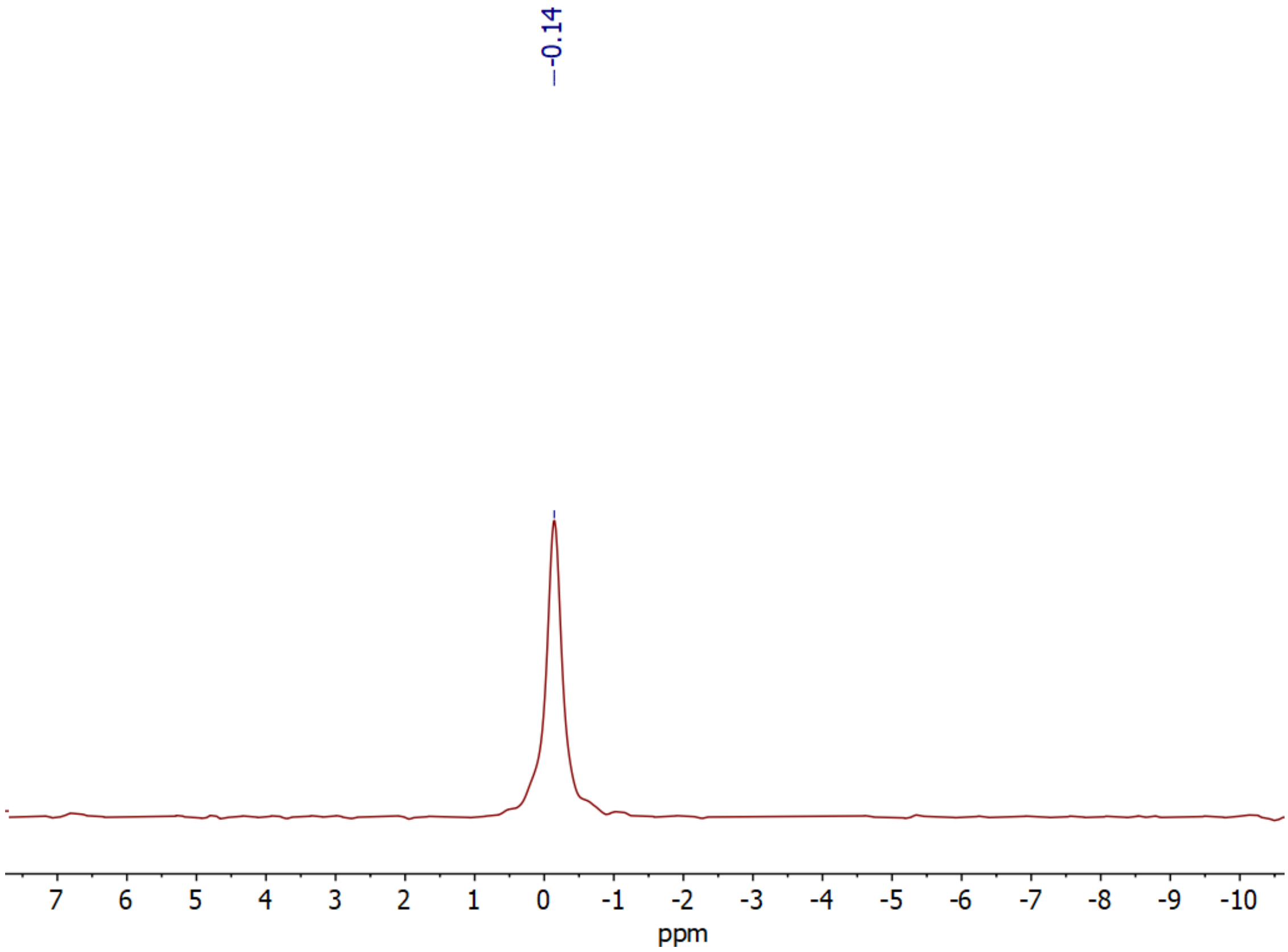

Figure S81. $^{31}P\{^{1}H\}$ NMR spectrum of H_2TF_4PPP-A (300 MHz, D_2O + 1 drop NaOD (40% in D_2O, 298 K).

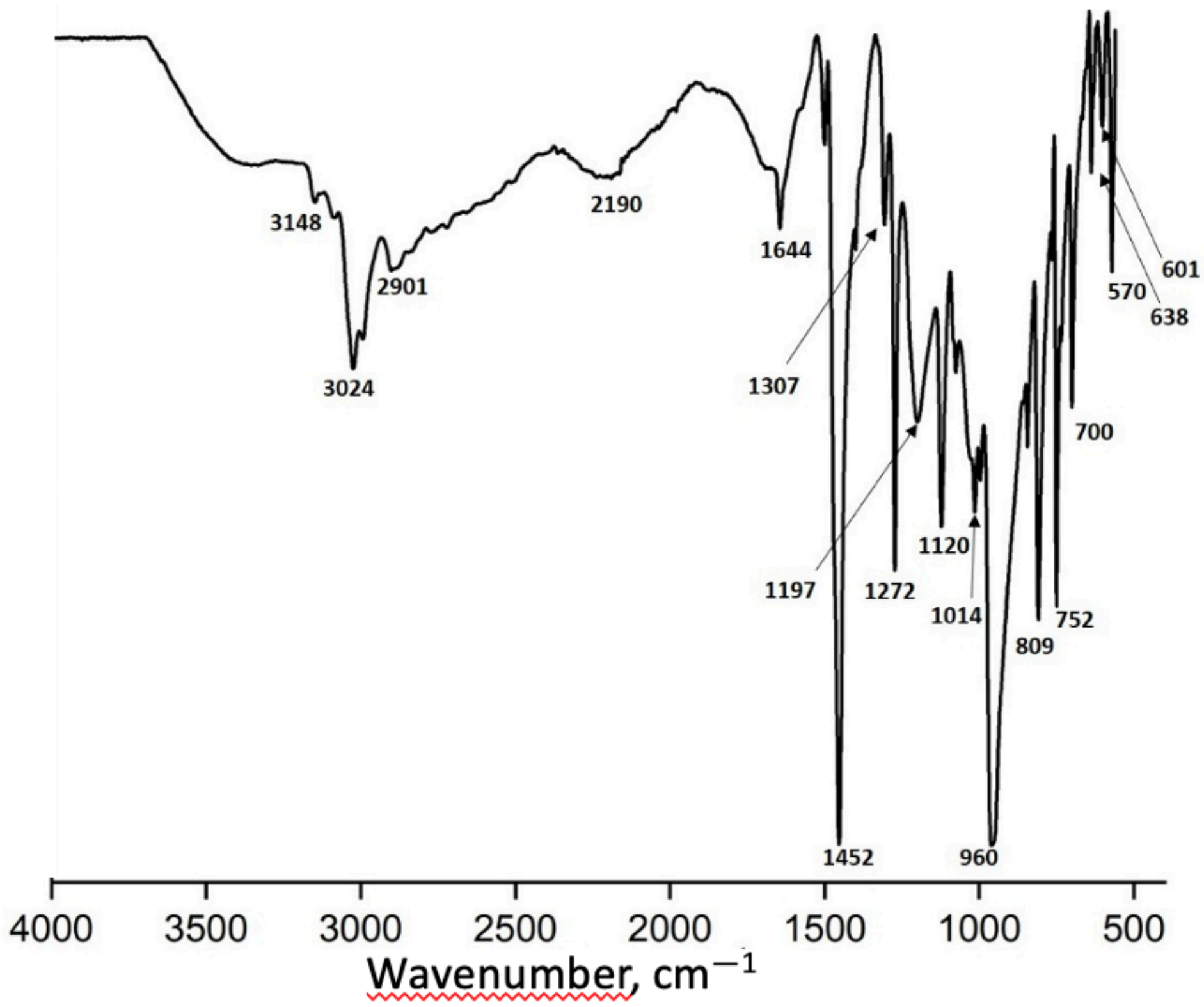

Figure S82. FT-IR spectrum of H_2TF_4PPP-A (neat).

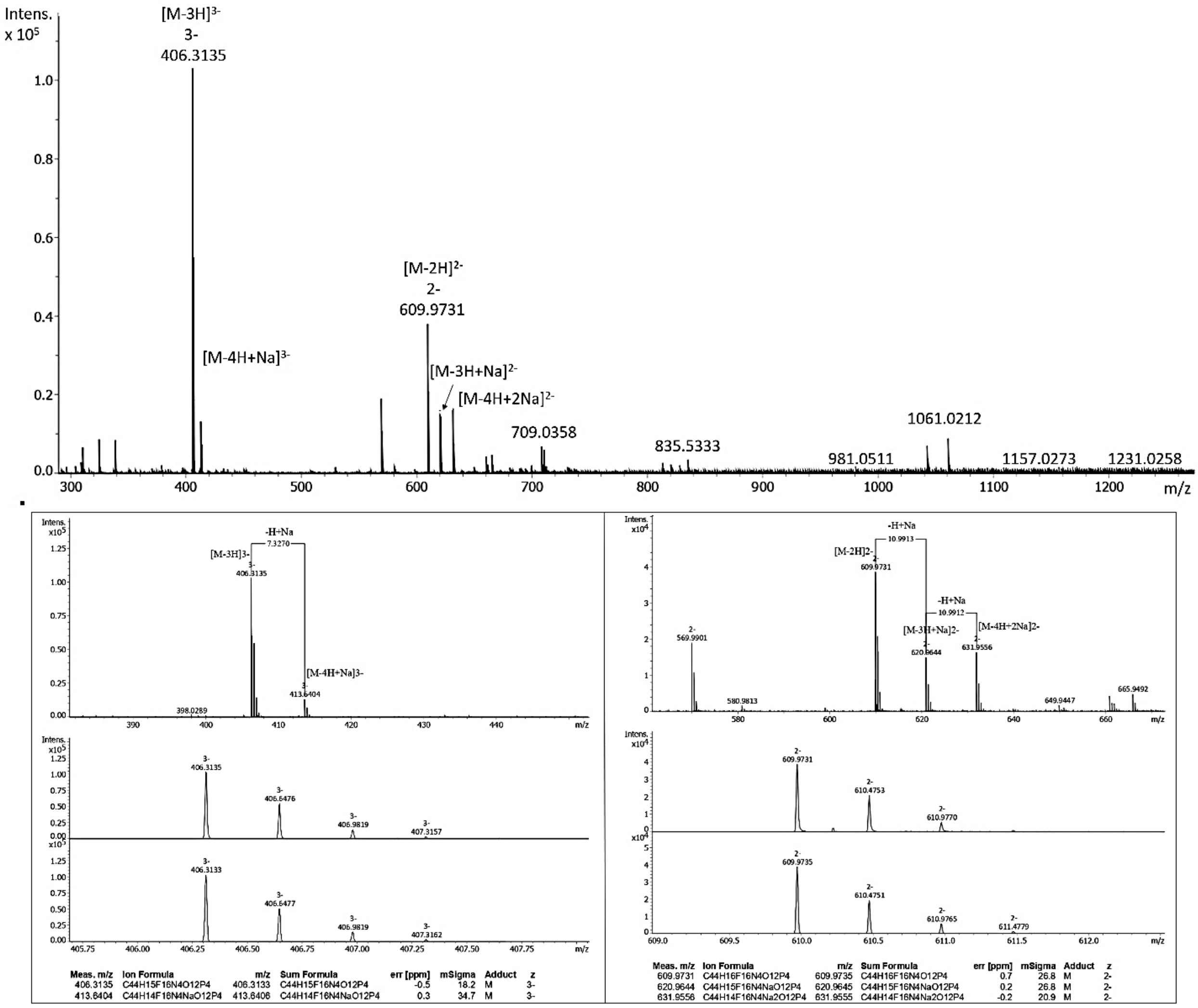

Figure S83. HRMS (ESI^-) spectrum of H_2TF_4PPP-A

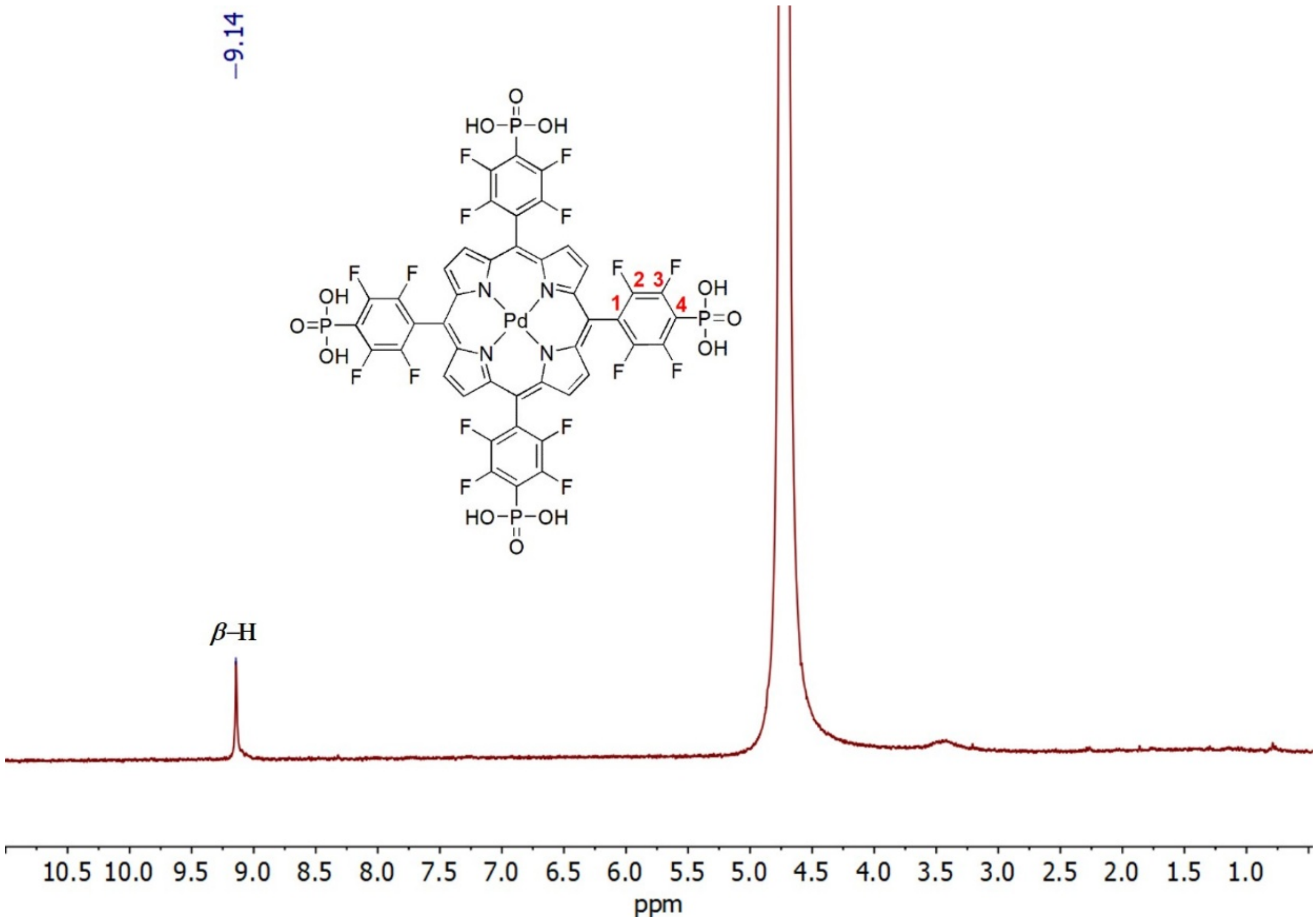

Figure S84. ^{1}H NMR spectrum of $PdTF_4PPP$-A (300 MHz, D_2O + 1 drop NaOD (40% in D_2O), 298 K).

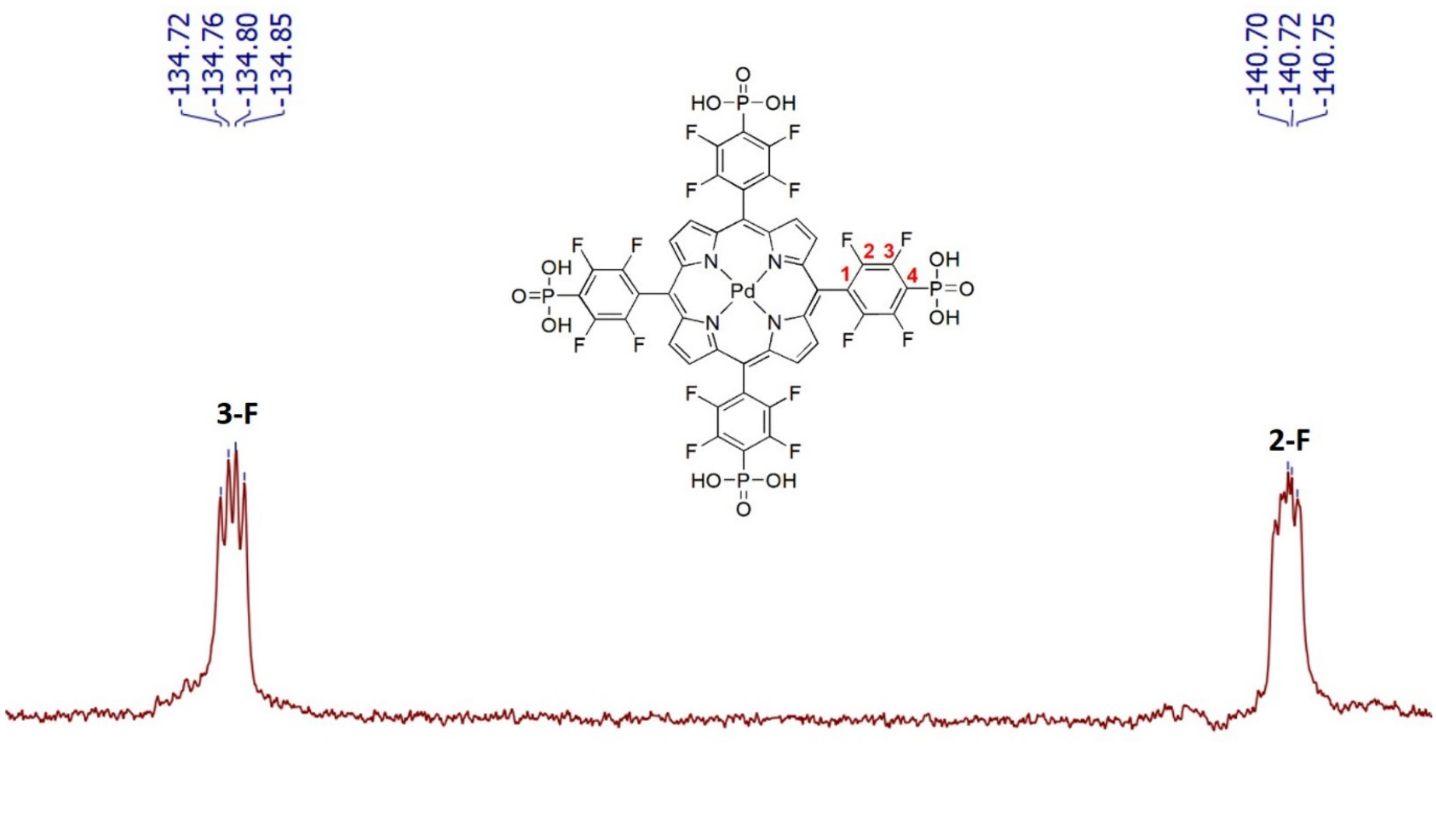

Figure S85. ^{19}F NMR spectrum of $PdTF_4PPP$-A (282 MHz, D_2O + 1 drop NaOD (40% in D_2O), 298 K)

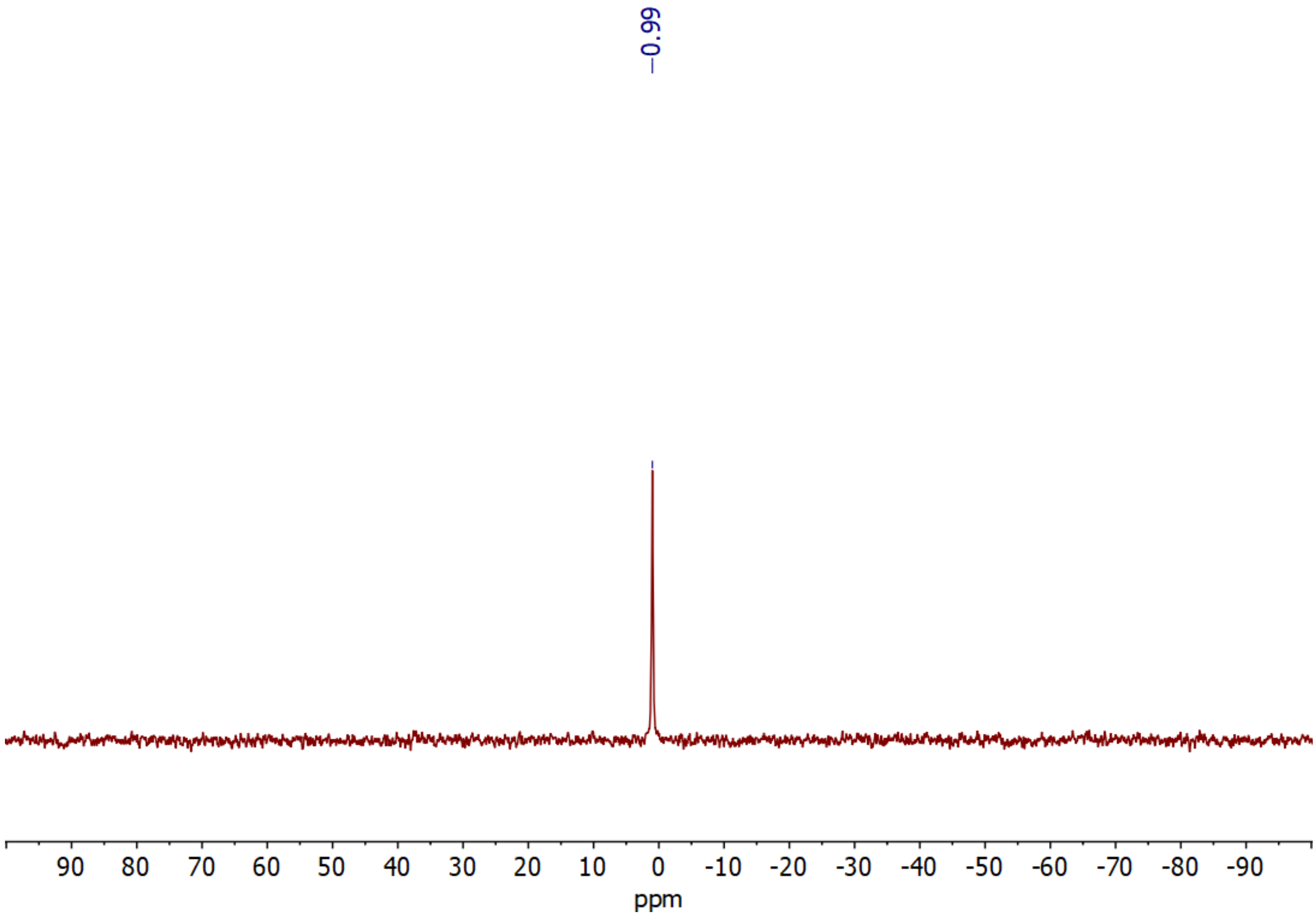

Figure S86. $^{31}P\{^{1}H\}$ NMR spectrum of $PdTF_4PPP$-A (121 MHz, CD_3OD, 298 K).

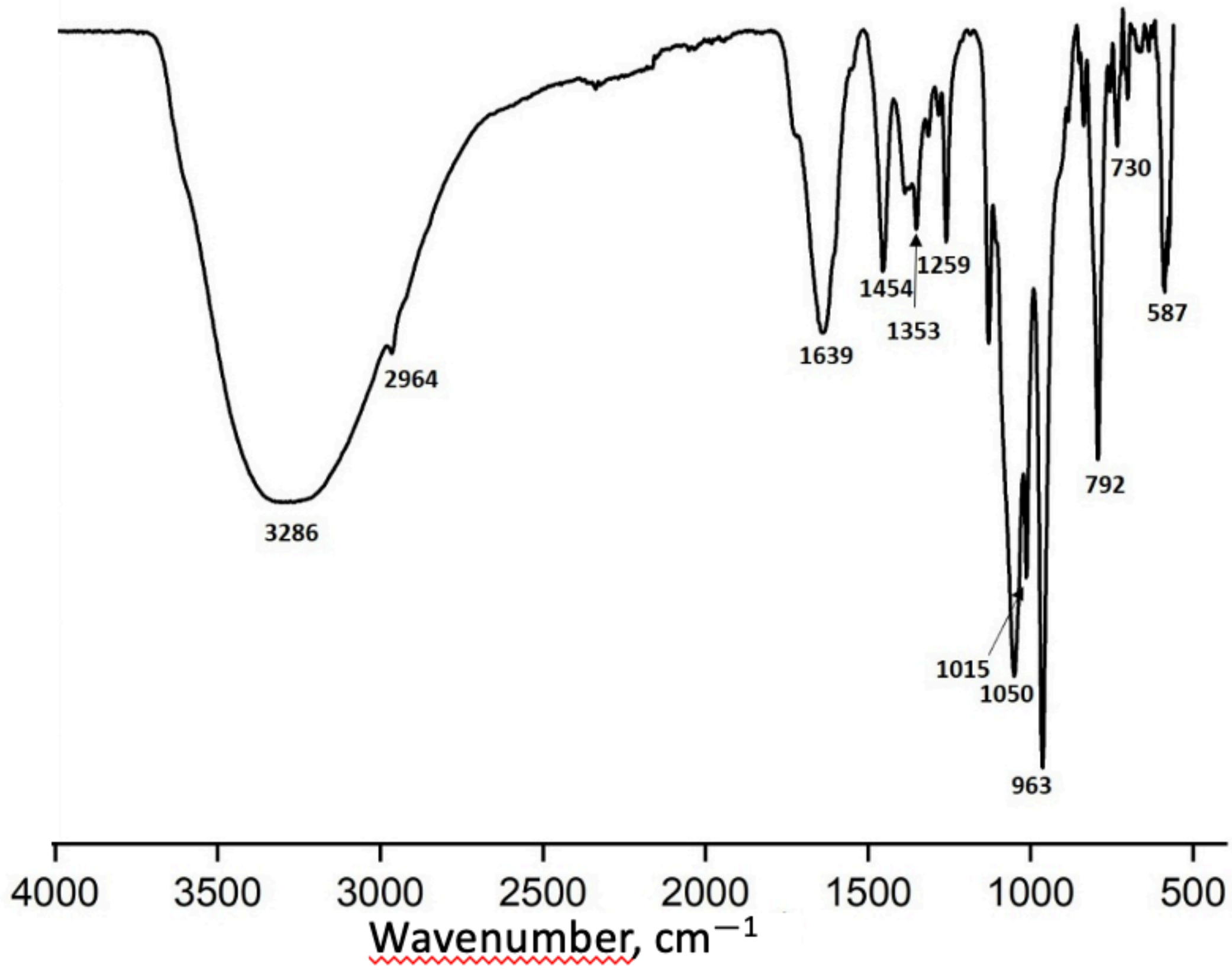

Figure S87. FT-IR spectrum of $PdTF_4PPP$-A (neat).

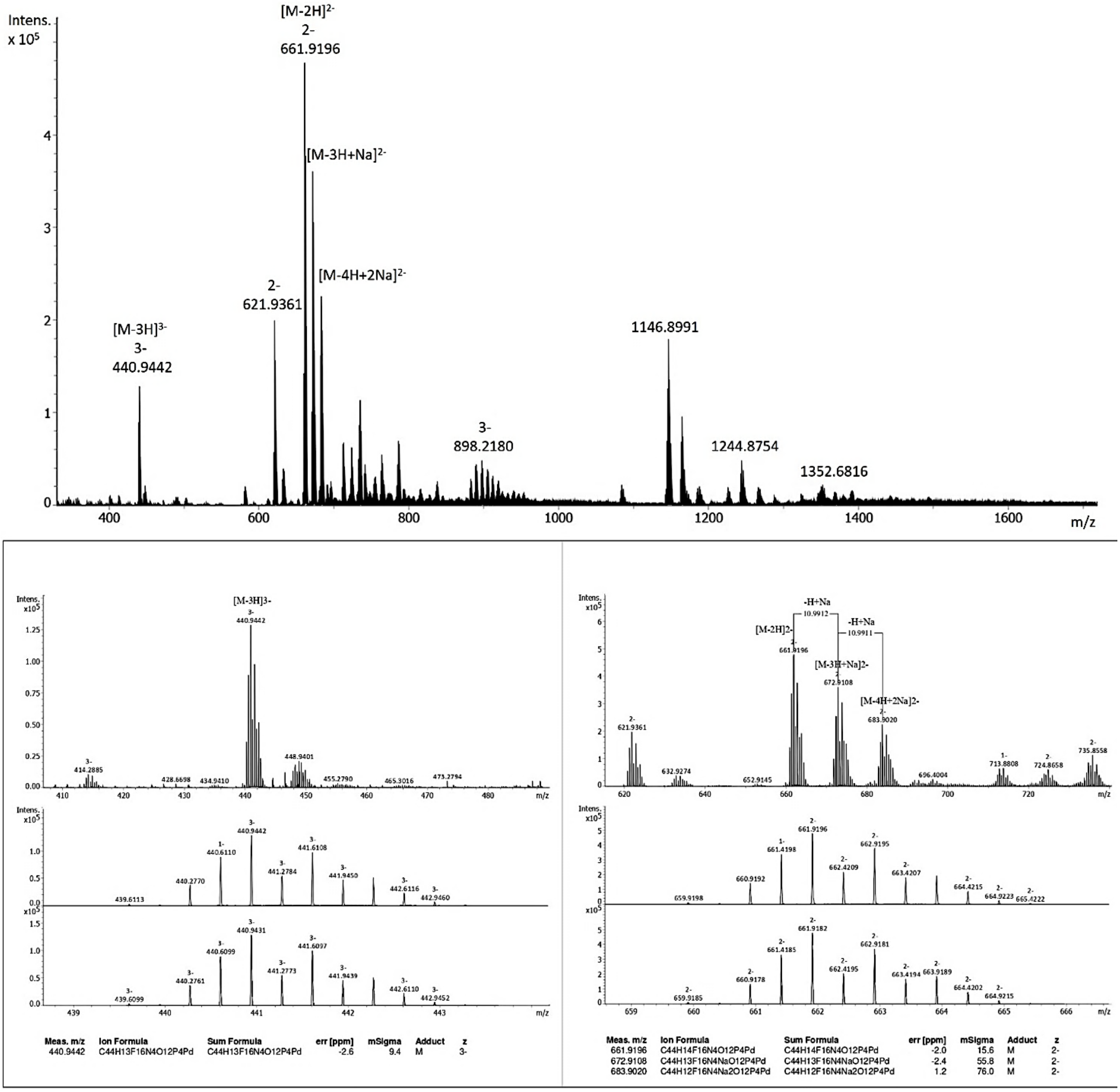

Figure S88. HRMS (ESI$^-$) spectrum of $PdTF_4PPP$-A.
